# Turning Nonselective Inhibitors of Type I Protein Arginine Methyltransferases into Potent and Selective Inhibitors of Protein Arginine Methyltransferase 4 through a Deconstruction–Reconstruction and Fragment-Growing Approach

**DOI:** 10.1021/acs.jmedchem.2c00252

**Published:** 2022-04-28

**Authors:** Giulia Iannelli, Ciro Milite, Nils Marechal, Vincent Cura, Luc Bonnefond, Nathalie Troffer-Charlier, Alessandra Feoli, Donatella Rescigno, Yalong Wang, Alessandra Cipriano, Monica Viviano, Mark T. Bedford, Jean Cavarelli, Sabrina Castellano, Gianluca Sbardella

**Affiliations:** ^†^Department of Pharmacy, Epigenetic Med Chem Lab and ^‡^Ph.D. Program in Drug Discovery and Development, University of Salerno, via Giovanni Paolo II 132, I-84084 Fisciano (SA), Italy; § Department of Integrated Structural Biology, Institut de Génétique et de Biologie Moléculaire et Cellulaire, 67400 Illkirch, France; □ Centre National de la Recherche Scientifique, UMR7104 Illkirch, France; ■ Institut National de la Santé et de la Recherche Médicale, U1258 Illkirch, France; △ Université de Strasbourg, 67400 Illkirch, France; ⊥ Department of Epigenetics and Molecular Carcinogenesis, The University of Texas MD Anderson Cancer Center, Houston, Texas 77030, United States

## Abstract

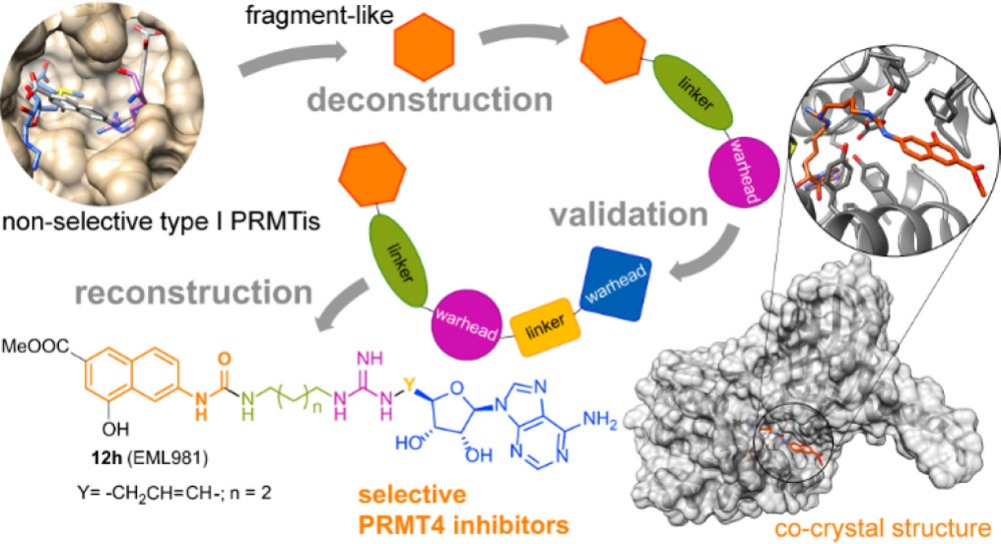

Protein arginine methyltransferases (PRMTs) are important therapeutic targets, playing a crucial role in the regulation of many cellular processes and being linked to many diseases. Yet, there is still much to be understood regarding their functions and the biological pathways in which they are involved, as well as on the structural requirements that could drive the development of selective modulators of PRMT activity. Here we report a deconstruction–reconstruction approach that, starting from a series of type I PRMT inhibitors previously identified by us, allowed for the identification of potent and selective inhibitors of PRMT4, which regardless of the low cell permeability show an evident reduction of arginine methylation levels in MCF7 cells and a marked reduction of proliferation. We also report crystal structures with various PRMTs supporting the observed specificity and selectivity.

## Introduction

The post-translational methylation of the guanidinium group of arginine residues of histone and nonhistone proteins by protein arginine methyltransferases (PRMTs) plays a fundamental role in many key cellular functions, including gene regulation, signal transduction, RNA processing, and DNA repair.^1−7^ On the other hand, the aberrant expression of PRMTs or the dysregulation of PRMT activity is associated with several diseases, including many types of cancer.^1,3,5,8−11^

On the basis of their methylation products, monomethylarginine (Rme1),^12^ asymmetric dimethylarginine (Rme2a), or symmetric dimethylarginine (Rme2s),^1,8^ the nine PRMT isoforms identified in human genome to date are classified into three subfamilies:^13^ type I PRMTs (PRMT1, PRMT2, PRMT3, PRMT4, PRMT6, and PRMT8), catalyzing mono- and asymmetric dimethylation, type II PRMTs (PRMT5 and PRMT9), catalyzing mono- and symmetric dimethylation, and PRMT7, the sole member of type III, which only catalyzes the formation of Rme1.^14^ Arginine methylation and PRMTs have been associated with a variety of diseases, including cancer and neurological and inflammatory diseases.^13^

Also, viral proteins from several viruses are methylated by PRMTs,^15−19^ including SARS-CoV-2 nucleocapsid (N) protein, the methylation of which at residues R95 and R177 is crucial for viral replication.^20^ Indeed, over the past 15 years the medicinal chemistry community has paid a growing attention to PRMTs,^11,13,21−26^ in particular to PRMT5 (with a few inhibitors in clinical trials)^21,27−33^ and to type I enzymes (both pan-type I^22,34−36^ and selective^24,37−45^).

**Chart 1:**
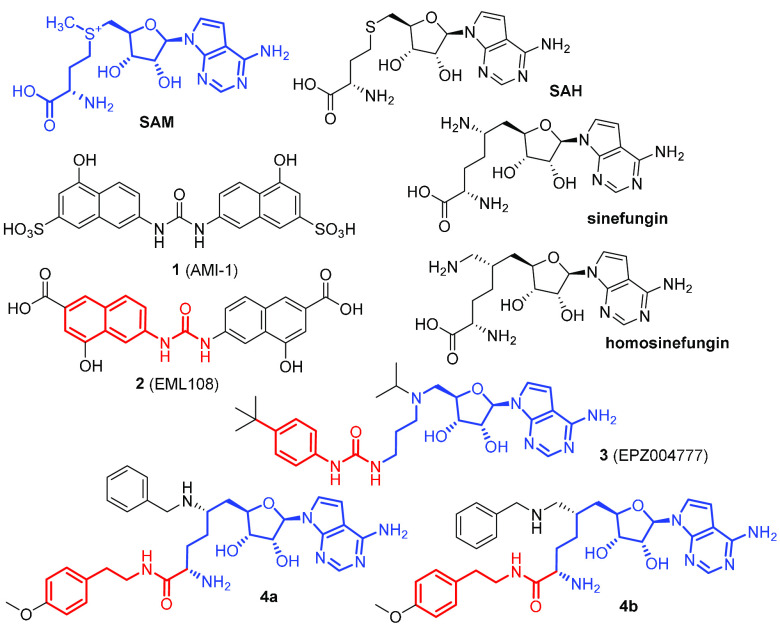
Structure of the Cofactor SAM, the Byproduct SAH, and Representative Inhibitors of PRMTsa

In our early studies in the field,^46^ we developed a series of type I PRMT inhibitors starting from 7,7′-(carbonylbis(azanediyl))bis(4-hydroxynaphthalene-2-sulfonic acid) **1** (AMI-1, Chart 1). In particular, the isosteric bis-4-hydroxy-2-naphthoic acid **2** (EML108, Chart 1) was able to prevent arginine methylation of cellular proteins in whole-cell assays, with activities comparable to AMI-1 (or even better than it). Moreover, compound **2** and its derivatives were found to be selective for arginine methyltransferases and essentially inactive against the lysine methyltransferase SET7/9.^47^

a Similar moieties (see text) are depicted in the same blue or red color.

**Figure 1:**
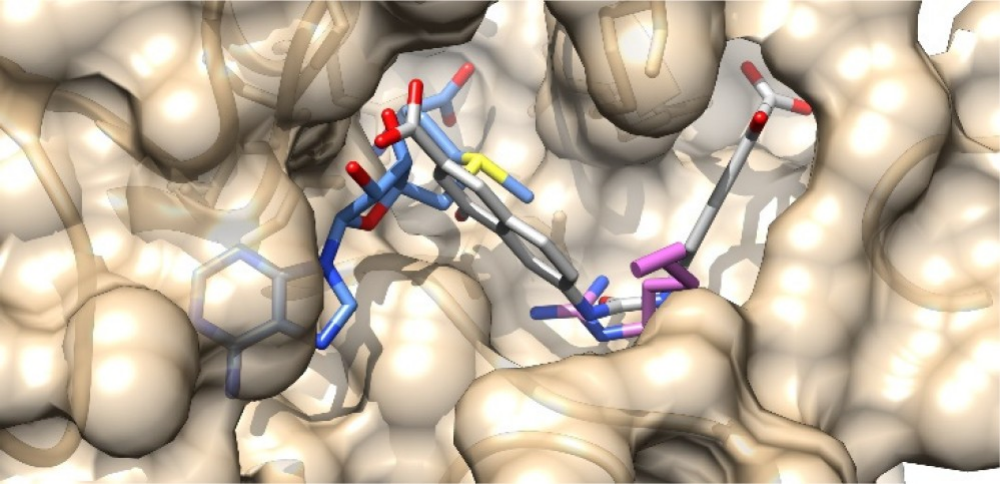
Predicted binding mode of **2** (EML108, in sticks, carbon atoms in gray) into PRMT1 (tan) catalytic site (PDB ID: 1OR8).^46−48^ SAM is depicted in cornflower blue, histone Arg in orchid (for clarity, only the side chain is shown).

On the basis of molecular modeling studies (docking and binding mode analysis, confirmed by structure-based 3-D QSAR models),^46,48^ we found that such inhibitors, as well AMI-1, bind PRMT1 (at that time chosen as representative of type I enzymes) between the *S*-adenosine-*l*-methionine (SAM) cofactor and substrate arginine binding sites without entirely occupying them. In particular, the binding site of the Arg guanidine group as well as both the adenosine and the methionine ends of the SAM binding pocket seems to be largely unoccupied (Figure 1). This is consistent with previously reported kinetics experiments.^49^ Therefore, a further decoration of the scaffold of compound **2** aimed to better occupy these pockets could, in principle, result in a gain of affinity.

Soon after our studies, Epizyme reported on the development of compound **3** (EPZ004777, Chart 1), a potent and selective inhibitor of lysine methyltransferase DOT1L based on the chemical structures of SAM and the corresponding product (*S*-adenosylhomocysteine, SAH) of DOT1L catalysis reaction as well as on its mechanism.^50^ We were intrigued by the structural similarity between the 4′-alkylphenylurea in this compound and the *N*-naphthylurea moiety of compound **2**. Although showing a remarkable selectivity against other histone methyltransferases, compound **3** was confirmed to inhibit also PRMT5 and PRMT7 (with IC_50_ values of 0.52 and 7.5 μM, respectively).^51^ Similarly, the amidic derivatives **4a** and **4b** (Chart 1) of the nonspecific SAM-dependent enzyme inhibitors sinefungin and 6′-homosinefungin were recently reported as PRMT inhibitors, with IC_50_ values against PRMT4 of 43 nM and 1.9 μM, respectively.^52^ Again, just like the 4′-alkylphenylurea in compound **3**, the *N*-phenethylamide portion in compounds **4a** and **4b** resembles the naphthylurea (in red in Chart 1) and mimics the lysine substrate covalently linked to a SAM-like moiety (in blue in Chart 1). It is noteworthy that **4b** inhibits modestly (IC_50_ = 1.9 μM) but selectively PRMT4 with no appreciable activity on other PRMTs. On the other hand, **4a**, which differs from **4b** only by the methylene group bridging the C5 position of the hexanamide with the benzylamine nitrogen, shows a dramatic increase of potency against PRMT4 (IC_50_ of 43 nM) but maintains a definite activity against type II and III PRMTs. This supports the hypothesis that the distance between the pharmacophoric moieties that are correlated with PRMTs inhibition plays a key role in potency and selectivity.

**Figure 2:**
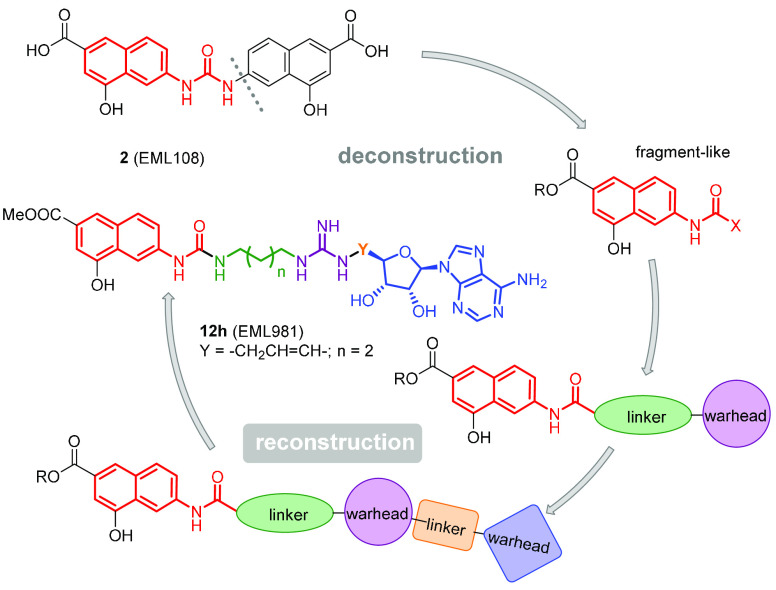
Flowchart of our design strategy.

Based on these considerations and pursuing our interest in the identification of potent and selective PRMT inhibitors,^7,44,46,47,53−59^ we resolved to reduce the structure of **2** to a single *N*-naphthylurea moiety and to grow it into a more complex structure incorporating warheads able to better bind the above-mentioned available pockets. The derivatives resulting from this design concept (Figure 2) were screened using in vitro biochemical assays to study their potency and selectivity in the inhibition of the various PRMTs. Herein, we report the design and synthesis of such compounds and the identification of **12h** (EML981) as a potent and selective inhibitor of PRMT4/CARM1. We also report cocrystallization studies supporting the observed specificity and selectivity of compound **12h**.

## Results and Discussion

### Design Strategy

Our previous molecular modeling studies^46,48^ performed on compound **2** and its derivatives had suggested a binding mode between the cofactor and substrate binding pockets, without fully occupying them. In particular, one of the two 4-hydroxy-2-naphthoic moieties partially occupies the substrate binding site without establishing interactions with the two conserved glutamate residues of the so-called “double-E loop” critical for chelating and orienting the Arg guanidine group.^60^ On the other hand, the binding of the second 4-hydroxy-2-naphthoic moiety seems to leave the SAM binding pocket largely unoccupied (Figure 1). Therefore, we decided to base our design strategy, schematically depicted in Figure 2, on a “deconstruction–reconstruction” approach that has gained traction in recent years.^61−63^ The concept underlying this approach is simple: since traditional fragment-based drug discovery (FBDD) combines fragments into a final molecule,^64^ it is typically possible to deconstruct a known ligand to obtain a relatively smaller fragment library.^65,66^ Therefore, we deconstructed our previously identified PRMTs inhibitors and decided to extend the resulting naphthylamide fragment using a growing approach.^67^

**Figure 3:**
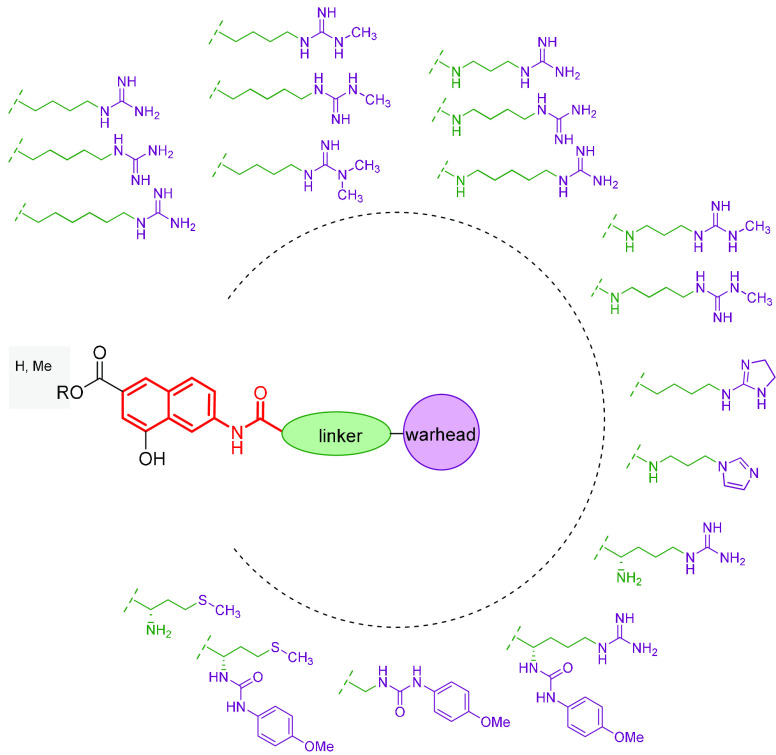
Compounds designed for the first reconstruction step.

We then designed a series of derivatives (Figure 3) incorporating the 4-hydroxy-2-naphthoic group bridged by an amide or urea group with an arginine surrogate (arginine mimetic moiety). The effect of the introduction of a methionine was also explored. The compounds (**5**–**11**, Table 1) were synthesized and, at first, analyzed for known classes of assay interference compounds.^68^ All derivatives were not recognized as PAINS according to the SwissADME web tool (http://www.swissadme.ch),^69^ the Free ADME-Tox Filtering Tool (FAF-Drugs4) program (http://fafdrugs4.mti.univ-paris-diderot.fr/),^70^ and the “False Positive Remover” software (http://www.cbligand.org/PAINS/)^71^ nor as aggregators according to the software “Aggregator Advisor” (http://advisor.bkslab.org/).^72^

**Table 1:**
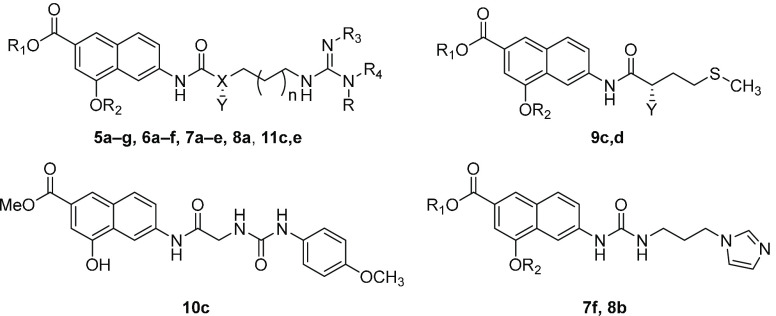

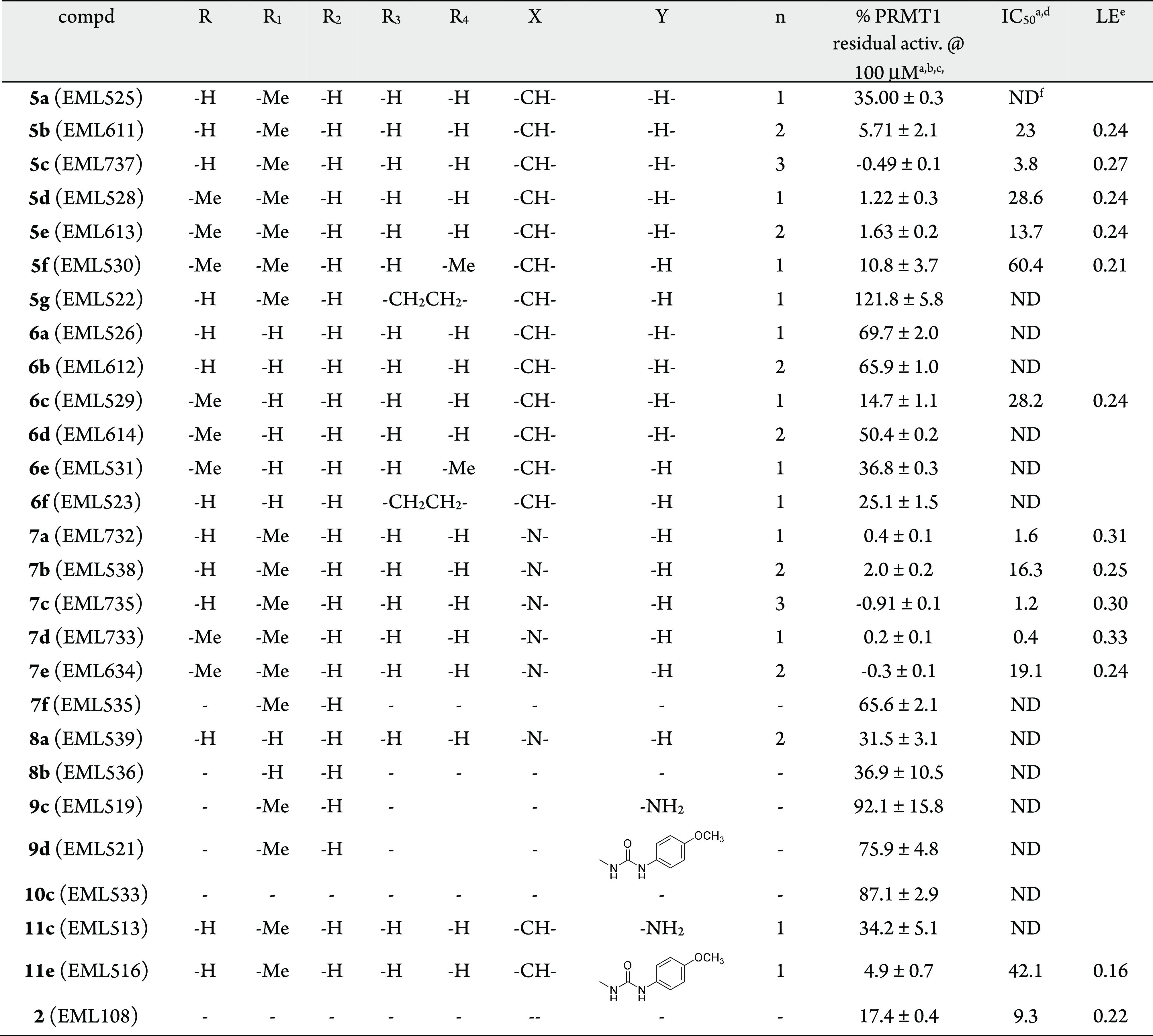
Inhibitory Activities of Compounds **5**–**11** against *h*PRMT1

a AlphaLISA was used for both fixed dose and IC_50_ determinations against human recombinant PRMT1 (0.9 nM, final concentration). Histone H4 (1–21) peptide, biotinylated (100 nM, final concentration), and SAM (2 μM, final concentration) were used as substrate and cofactor, respectively.

b Compounds were tested at a 100 μM fixed concentration.

c Enzyme residual activity percentage calculated with respect to DMSO.

d Compounds were tested in 10-concentration IC_50_ mode with threefold serial dilutions starting at 100 μM. Data were analyzed with GraphPad Prism software (version 6.0) for IC_50_ curve fitting.

e Ligand efficiency (LE) calculated from IC_50_ as a surrogate for *K*_D_.

f ND, not determined.

Then, we resolved to determine their effect on the catalytic activity of human recombinant PRMT1, chosen as representative of class I PRMTs. To this aim, we used an in-house peptide-based AlphaLISA assay measuring the levels of H4R3me, developed by us (see the Experimental Section) because the commercially available homogeneous assay kit (BPS, #52054) did not work properly in our hands and gave an unacceptably narrow assay window. All the compounds were tested at a fixed concentration of 100 μM using **2** (EML108) as the reference compound. Then, the compounds that displayed a greater than 85% inhibition (residual enzyme activity <15%) were selected, and the corresponding IC_50_ values were determined (Table 1).

Overall, the results show that one of the two 4-hydroxy-2-naphthoic groups could be efficiently replaced by an arginine-mimetic group moiety. This result is consistent with our initial assumption that our PRMTs inhibitors bind PRMT1 between the SAM and arginine binding sites without fully occupying them.

Structure–activity relationship (SAR) analysis of tested compounds suggested that, in general, ester derivatives are better than the corresponding acids (for example, compare inhibiting activities of compounds **5a**, **5b**, **5d**, and **5e** with those of **6a**–**6d**, respectively) and urea derivatives are more active (compare **7a**–**7d** with **5a**–**5d**, respectively) or comparable (**7e** vs **5e**) to their amide counterparts. The presence of a single methyl on one of the terminal nitrogen atoms of the guanidine group also improves the activity in the amide series (compare **5d** and **5e** with **5a** and **5b**, respectively), whereas this is less evident in the urea series (compare **7d** and 7**e** with **7a** and **7b**, respectively) and in carboxylic acids with respect to ester derivatives. On the contrary, the introduction of a second methyl group on the same nitrogen atom leads to a decrease of inhibiting activity (compare **5f** with **5d**). When both terminal nitrogens are substituted and blocked into an imidazoline ring (as in the case of compounds **5g** and **6f**), the inhibiting activity is even lower. Similarly, in the urea series the replacement of the guanidine group with an imidazole leads to the loss of activity (compare **7f** with **7a**). Regarding the effect of the methylene linker length, in the amide series the activity seems to increase with the number of carbon atoms of the linker (see, for example, the activities of compounds **5a**–**5c** and **7a**–**7c**) at least for ester derivatives, whereas for the corresponding acids the effect is less evident (**6a** vs **6b**) or opposite (**6c** vs **6d**). On the other hand, an “odd/even effect”^73−75^ seems to occur in the urea series, where an even number of carbon atoms in the alkyl spacer (as in the case of **7b** and **7e**, *n* = 2, total carbon atoms in the linker = 4) is less favorable than an odd number (as in the case of **7a** and **7d**—*n* = 1, total carbon atoms in the linker = 3—or **7c**—*n* = 3, total carbon atoms in the linker = 5).

The introduction of an α-amino group (as in derivative **11c**) does not improve the activity of the compound. On the contrary, the introduction at the same position of a *p*-methoxyphenylurea resulted in compounds with improved inhibiting capability, even if compared to the α-unsubstituted compounds (compare **11e** with **11c** and **5a**). Last, the removal of the guanidine group is detrimental for the inhibiting activity and cannot be compensated by the sole presence of a *p*-methoxyphenylurea in the α position relative to the amide carbonyl group. In fact, both the methionine and glycine derivatives **9c**, **9d**, and **10c** are inactive or scarcely active. Noteworthy, the effects appear to be additive. In fact, the ester derivative of the urea series, with three carbon atoms in the linker and a methyl group on the terminal nitrogen of the guanidine, namely compound **7d**, is the most active and the most efficient ligand among the tested compounds, showing a submicromolar IC_50_ value (0.4 μM) and a ligand efficiency^76^ value (LE) of 0.33.

**Figure 4:**
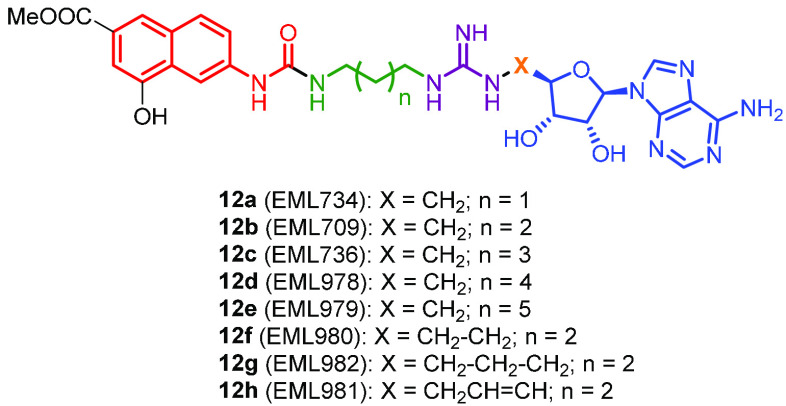
Compounds designed for the second fragment-growing step.

Therefore, on the basis of the structure–activity relationships, we resolved to select the scaffold of the latter compound for the second step of the construction of our multisubstrate ligands. Considering the effect of the linker length on the activity against PRMT1 and also with the aim to explore a possible difference among various PRMTs, first we designed and synthesized three derivatives in which the linker between the urea and the guanidine groups included three, four, or five carbon atoms and the adenosine moiety was connected to a guanidine ω-nitrogen through a methylene group (Figure 4, derivatives **12a**, **12b**, and **12c**, respectively).

**Figure 5:**
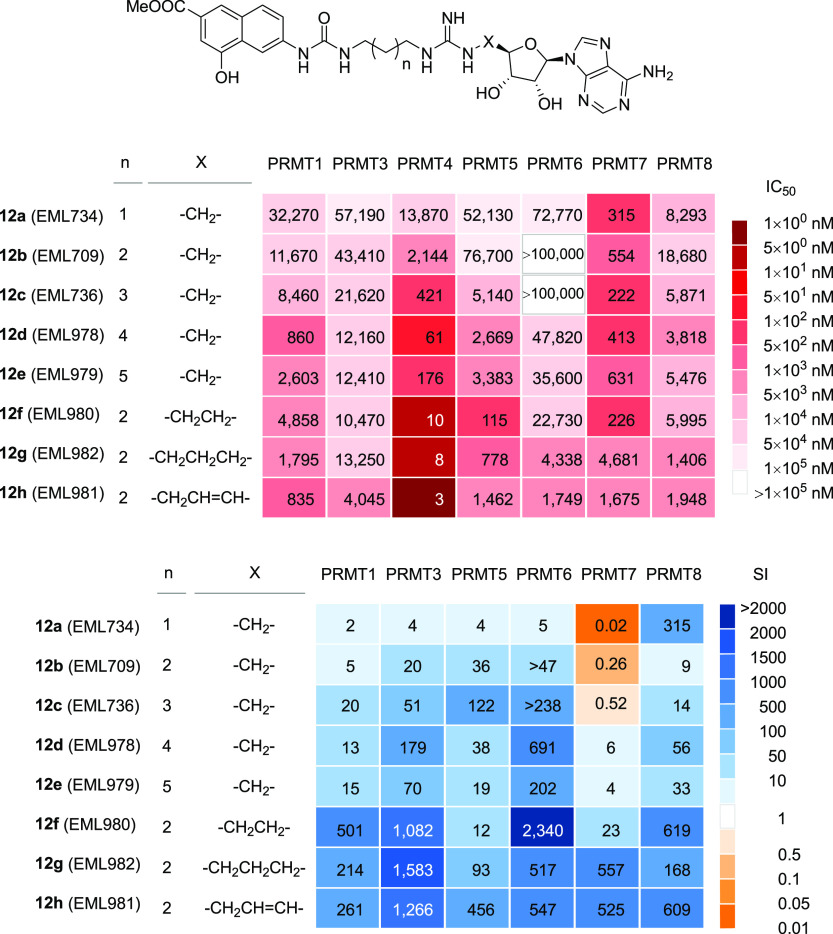
Inhibitory activities of compounds **12a**–**12h**: the heatmaps depict the IC_50_ values (nM) for compounds **12a**–**12h** (top panel) and the selectivity index (fold) for PRMT4 over the specified PRMT (bottom).

As in the case of derivatives **5**–**11**, we first determined the effect of compounds **12a**–**12c** on the catalytic activity of human recombinant PRMT1, using our AlphaScreen assay. As expected, the introduction of the adenosine moiety increased the inhibiting activity against PRMT1 as all the derivatives showed IC_50_ values in the submicromolar range (Table 2, values in parentheses). Prompted by these results, we next used a secondary screening approach to profile the activity of the compounds against a panel of four PRMTs (PRMT1, PRMT3, PRMT4, and PRMT5) using a radioisotope-based filter-binding assay. If compared to the results obtained from the AlphaScreen assay, in the more sensitive radioisotope-based assay compounds **12a**–**12c** were found to be less potent inhibitors of PRMT1 activity, but the IC_50_ values were still in the micromolar range (Table 2). Noteworthy, they were appreciably more active against PRMT4. We also noticed that the distance between the methyl 4-hydroxy-2-naphthoate moiety and the arginine-mimetic group significantly affects the inhibition of PRMT4 activity, whereas it has no significant effect on the activity of PRMT5 and only a moderate effect on PRMT1 and PRMT3. In fact, compound **12c** exhibits a submicromolar activity (IC_50_ = 0.42 μM) only against PRMT4, with a selectivity for the latter ranging from 20-fold (over PRMT1; Table 2) to 122-fold (over PRMT5; Table 2). Based on these outcomes, we speculated that more potent and selective PRMT4 inhibitors could be generated by modulating the distance between the three pharmacophoric moieties. Therefore, we synthesized a second set of derivatives (compounds **12d**–**12h**, Figure 4) in which the distance between the 4-hydroxy-2-naphthoate moiety and the guanidine group (compound **12d** and **12e**) or the distance between the guanidine group and the adenosine moiety (compounds **12f**–**12h**) was further increased. Again, all compounds **12a**–**12h** were analyzed for known classes of assay interference compounds^68^ and were not recognized as PAINS nor as aggregators. The compounds were then tested against a wider panel of PRMTs (including also PRMT6, PRMT7, and PRMT8) using the same radioisotope-based filter-binding assay. The results are reported in Table 2 and summarized as heatmaps in Figure 5. As expected, increasing the linker length between the 4-hydroxy-2-naphthoate and the guanidine group up to a total of four or five carbon atoms yielded a gain in the inhibitory activity against PRMT4, although moderate (6.2-fold for **12d** and 2.4-fold for **12e**). However, a similar gain was observed also against PRMT1, PRMT5, and PRMT6 (e.g., 9.8-fold and 3.2-fold against PRMT1 and 19.8-fold and 15.2-fold against PRMT5, for **12d** and **12e**, respectively), with a consequent reduction of selectivity. A lesser effect was observed against PRMT8, and a slight decrease of inhibiting activity was observed against PRMT7. On the other hand, we were pleased to find out that compounds **12f**–**12h**, featuring an increased distance between the guanidine group and the adenosine moiety, showed a remarkable and selective increase in potency against PRMT4, with a consistent gain in selectivity over six other human PRMTs. In particular, compound **12h** (EML981) showed a 668-fold gain in potency over its shorter counterpart **12b** and 261- to 1266-fold selectivity over the other PRMTs.

**Table 2:** Inhibitory Activities of Compounds **12a**–**12h** against Various PRMTs

	IC_50_ (μM)^a^^,^^b^							selectivity index for PRMT4 vs other PRMTs^c^						
no.	PRMT1	PRMT3	PRMT4	PRMT5	PRMT6	PRMT7	PRMT8	PRMT1	PRMT3	PRMT5	PRMT6	PRMT7	PRMT8	LE^d^
**12a**	32.27 (0.5)^e^	57.19	13.84	52.13	72.77	0.32	8.29	2	4	4	5	0.02	1	0.14
**12b**	11.67 (0.43)^e^	43.41	2.14	76.7	>100	0.55	18.68	5	20	36	>47	0.26	9	0.18
**12c**	8.46 (0.3)^e^	21.26	0.42	51.41	>100	0.22	5.87	20	51	122	>238	0.52	14	0.19
**12d**	0.86	12.2	0.068	2.6	47	0.41	3.82	13	179	38	691	6	56	0.21
**12e**	2.6	12.4	0.176	3.38	35.6	0.631	5.84	15	70	19	202	4	33	0.20
**12f**	4.86	10.5	0.0097	0.115	22.7	0.226	6	501	1082	12	2340	23	619	0.24
**12g**	1.80	13.3	0.0084	0.778	4.34	4.68	1.41	214	1583	93	517	557	168	0.24
**12h**	0.835	4.05	0.0032	1.46	1.75	1.68	1.95	261	1266	456	547	525	609	0.25

a Compounds were tested in 10-concentration IC_50_ mode with threefold serial dilutions starting at 100 μM. Data were analyzed with GraphPad Prism software (version 6.0) for IC_50_ curve fitting.

b Unless differently indicated, the values were obtained in a radioisotope-based filter assay, using 5 μM histone H4 (for PRMT1, PRMT3, and PRMT8), histone H3 (for PRMT4), histone H2A (for PRMT5), or GST-GAR (for PRMT6 and PRMT7) as the substrate and *S*-adenosyl-*l*-[methyl-^3^H]methionine (1 μM) as methyl donor.

c Selectivity index for PRMT4 over the specified PRMT, calculated as the ratio between the IC_50_ against the specified PRMT and the IC_50_ against PRMT4 and rounded to the nearest integer.

d Ligand efficiency (LE) for PRMT4 calculated from IC_50_ as a surrogate for *K*_D_.

e Obtained in the AlphaLISA assay, using human recombinant PRMT1 (0.9 nM, final concentration). Histone H4 (1–21) peptide, biotinylated (100 nM, final concentration), and SAM (2 μM, final concentration) were used as the substrate and cofactor, respectively.

The selectivity of compound **12h** was further assessed against a panel of eight lysine methyltransferases (KMTs), including the SET-domain-containing proteins ASH1L/KMT2H, EZH2/KMT6, MLL1/KMT2A, SET7/9/KMT7, SETD8/KMT5A, SUV39H2/KMT1B, and SUV420H1/KMT5B and the non-SET-domain-containing DOT1L/KMT4.^77^ To this aim, the inhibition of **12h** toward these selected enzymes was assessed at two different concentrations (1 and 10 μM, respectively, >300 and >3000 fold higher than the IC_50_ value against PRMT4) using SAH,^78−80^ chaetocin (for ASH1L),^81^ or ryuvidine (for SETD8)^82^ as reference compounds. Noteworthy, we found that none of the enzymes was inhibited by **12h** even at the higher tested concentration (Figure S2 and Table S1, Supporting Information).

### SPR-Based Studies of Binding to PRMT4

First identified as a transcriptional regulator,^83^ PRMT4, also known as coactivator-associated arginine methyltransferase 1 (CARM1), is a type I enzyme that regulates gene expression by numerous mechanisms. It positively regulates transcription by methylating H3R17 and H3R26,^84,85^ methylates steroid receptor coactivators including SRC3 and CBP/p300, and can directly act as a transcriptional coactivator of nuclear receptors.^86,87^ PRMT4 also methylates a variety of other targets, including splicing factors such as CA150,^91^ to regulate the exon skipping, and RNA-binding proteins (e.g., PABP1, HuR, and HuD),^88−90^ to modify their ability to bind to the transcription-related proteins. PRMT4 also regulates the looping of enhancers and promoters by methylating MED12, which is a component of the mediator.^92^

It has been demonstrated that PRMT4 is overexpressed in various cell lines of hematologic cancers and solid tumors, such as leukemia,^93,94^ breast,^95^ prostate,^96^ liver,^97^ and colorectal cancers.^98,99^ Moreover, the enzyme is overexpressed in ischemic hearts and hypoxic cardiomyocytes, and it has been suggested that PRMT4 has an essential role in myocardial infarction and cardiomyocyte apoptosis.^100^ Other emerging functions of PRMT4 include autophagy, metabolism, early development, pre-mRNA splicing and export, and localization to paraspeckles.^101^ Also, it was recently found that in lymphomas that carry mutation in p300/CBP, PRMT4 loss or inhibition is a vulnerability.^102^ Therefore, PRMT4 is considered an appealing therapeutic target for anticancer drug development, and in fact a few inhibitors have been developed with different degrees of potency and selectivity.^24,41,52,103^

**Figure 6:**
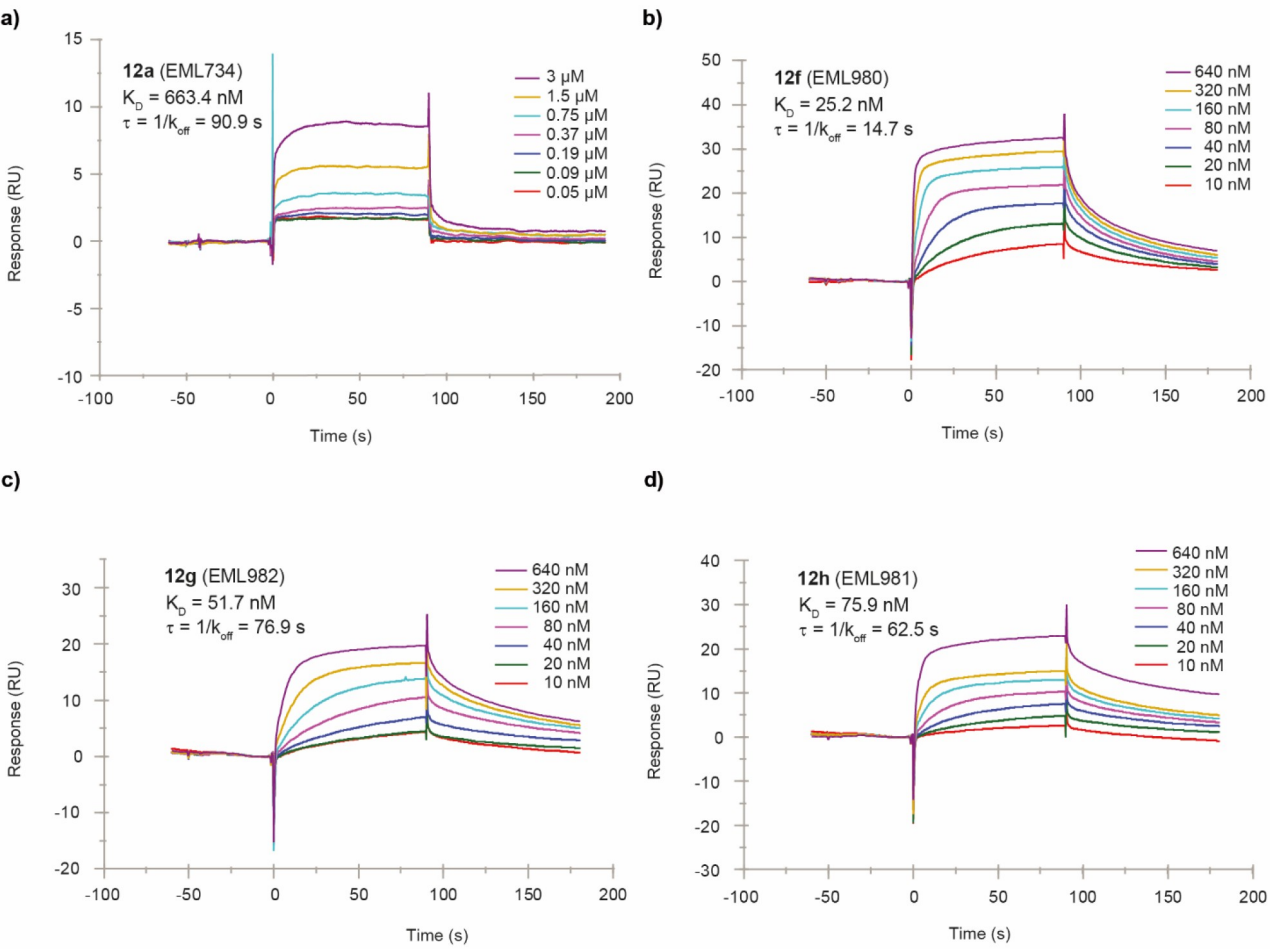
Sensorgrams obtained from the SPR interaction analysis of compounds **12a** and **12f**–**12h** (panels a–d, respectively) binding to immobilized PRMT4. Each compound was injected at different concentrations (from 3 to 0.05 μM for **12a** and from 640 to 10 nM for **12f**–**12h**) with an association and a dissociation time of 90 s, with a flow rate of 30 μL/min. The equilibrium dissociation constants (*K*_D_) were derived from the ratio between kinetic dissociation (*k*_off_) and association (*k*_on_) constants.

To further characterize the effect of compounds **12a**–**12h** on PRMT4, we resolved to evaluate their direct binding to the target protein using Surface Plasmon Resonance (SPR). To this aim, human PRMT4 (full length) was covalently immobilized on a sensor chip surface using an amine-coupling approach, and the three compounds were injected at different concentrations over the protein surface. To reduce false positives from detergent-sensitive, nonspecific aggregation-based binding, detergents (0.05% Tween-20) were added to the running buffer in all experiments. A specific and strong binding interaction was demonstrated between PRMT4 and each compound, with equilibrium dissociation constant (*K*_D_) values in the nanomolar range for the most active derivatives **12f**–**12h** (Figure 6 and Table 3). Compound **12f** interacts with PRMT4 with higher affinity (*K*_D_ = 25.2 nM; Table 3) compared to **12g** (*K*_D_ = 51.7 nM) and **12h** (*K*_D_ = 75.9 nM). As shown by the sensorgrams depicted in Figure 6 and Figure S1 (Supporting Information), compounds **12f**–**12h** dissociate from the protein slower than compounds **12a**–**12e**. Nonetheless, the in vitro residence time (τ_R_)^104^ values are quite similar (Table 3), in particular for compounds **12b**, **12c**, **12e**, **12g**, and **12h**, and cannot account alone for the higher potency of compounds **12g** and **12h**. On the contrary, the association rate constants (*K*_on_) certainly contribute to the affinity for the target, being significantly higher for compounds **12f**–**12h** than for compounds **12a**–**12e** (Table 3).

**Table 3:** Affinity and Kinetic Parameters Derived from SPR Experiments

compound	*K*_D_ (nM)	*k*_on_ (1/Ms)	*k*_off_ (1/s)	τ_R_ (s)
**12a**	663.4	1.67 × 10^4^	0.011	90.9
**12b**	2300	6.52 × 10^3^	0.015	66.7
**12c**	359.2	3.49 × 10^4^	0.013	76.9
**12d**	2800	2.70 × 10^5^	0.760	1.31
**12e**	603.4	2.58 × 10^4^	0.015	66.7
**12f**	25.2	2.70 × 10^6^	0.068	14.7
**12g**	51.7	0.25 × 10^6^	0.013	76.9
**12h**	75.9	0.21 × 10^6^	0.016	62.5
SAM	9.6	2.90 × 10^5^	0.003	333.3

### Structural Studies

Then we resolved to study and compare the binding modes of compounds **12a**–**12h** with different PRMTs. In particular, we resolved to compare compounds **12a**–**12c** (less potent and selective PRMT4 inhibitors in the series **12**, upper part of the heatmaps in Figure 5) and compounds **12f**–**12h** (most potent and selective PRMT4 inhibitors of the series, lower part of the heatmaps in Figure 5) in cocrystallization studies performed using five PRMTs, namely isolated domains of *mm*PRMT4 (*Mus musculus* PRMT4, residues 130–487 or 140–497), full length and truncated *Rattus norvegicus* PRMT1, full length *Mus musculus* PRMT2, *mm*PRMT6 (*Mus musculus* PRMT6, residues 34–378), and full length *Mus musculus* PRMT7.

Unfortunately, no cocrystals were obtained with PRMT1, PRMT2, or PRMT7. On the contrary, we were able to cocrystallize the compounds in the complex with both PRMT4 and PRMT6. Crystallization, data collection, and structure refinement are fully described in the Experimental Section.

**Figure 7:**
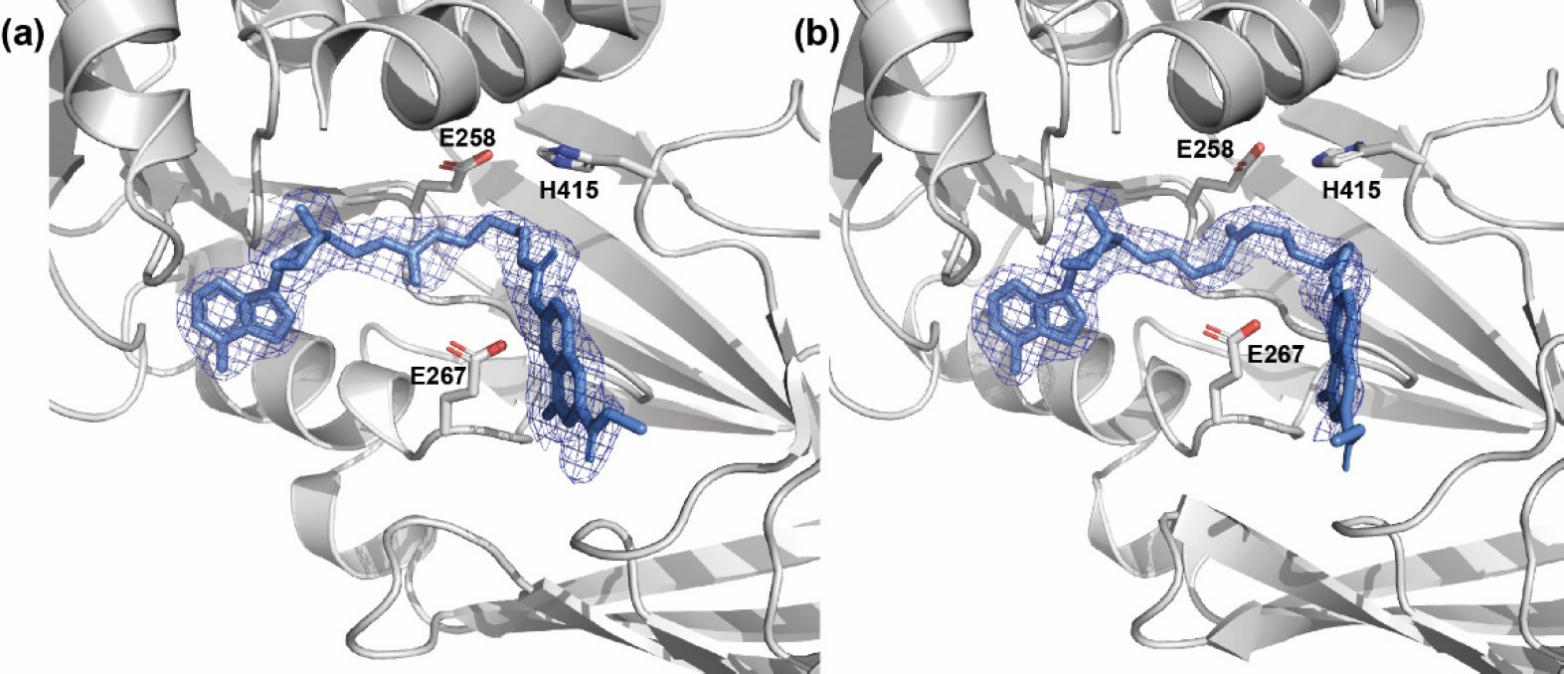
Electron density (2*F*_obs_ – *F*_calc_) weighted maps. Compound **12b** (a) and compound **12h** (b) bound to subunit B of *mm*PRMT4 (PDB IDs: 7PV6 and 7PUC). PRMT4 is represented as a gray cartoon, and compounds are represented as cornflower blue sticks. Maps are represented as a mesh, with the contouring level set to 1σ. For clarity, N-terminal helices (residues 135–155) of PRMT4 are not shown. E258 and E267 belonging to the double-E loop and H415 of the THW loop are also displayed as sticks.

**Figure 8:**
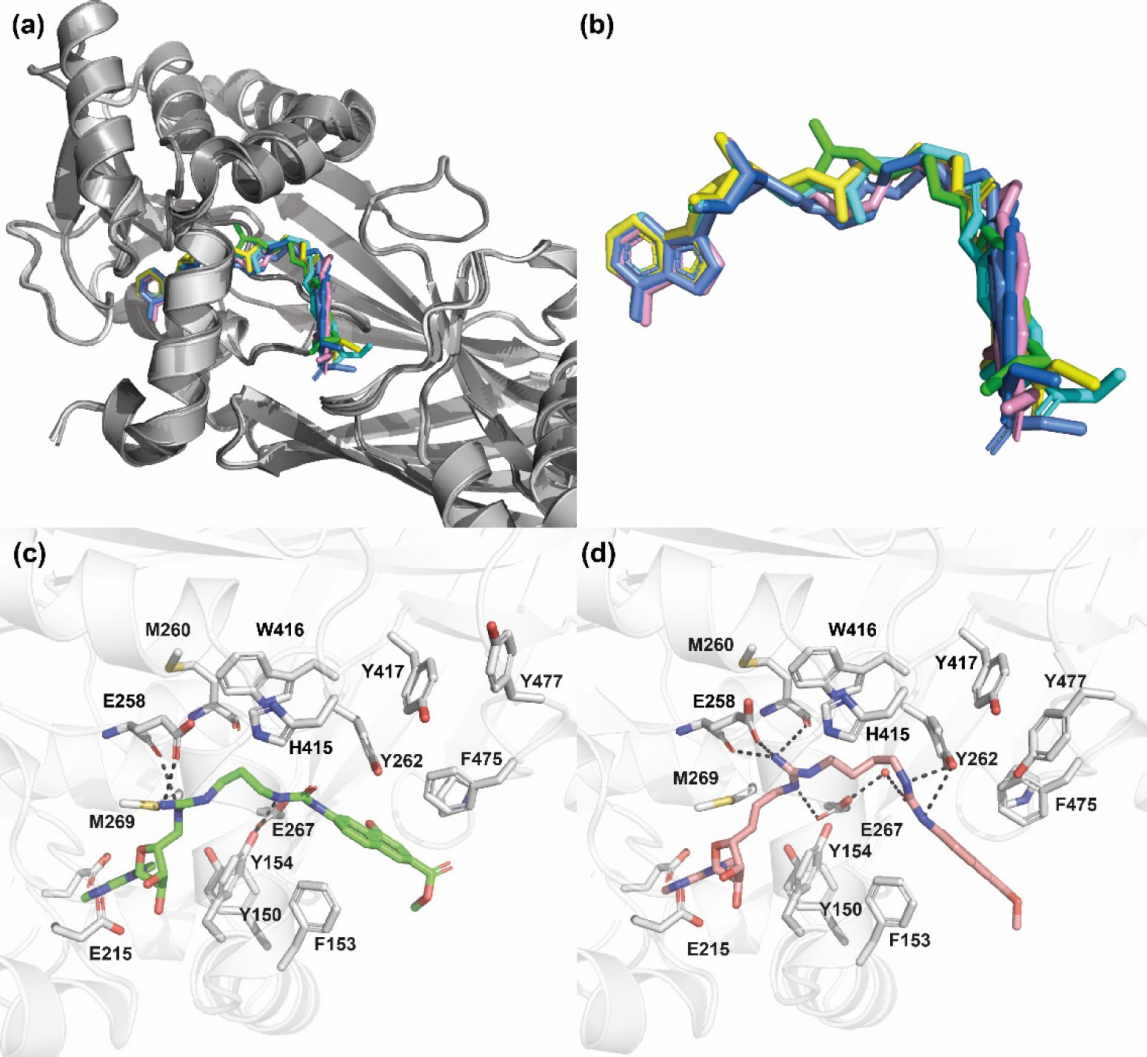
Structures of *mm*PRMT4 in complex with compounds **12a**–**12c** and **12f**–**12h** (PDB IDs: 7PV6, 7PPY, 7PPQ, 7PU8, 7PUQ, and 7PUC, respectively). (a) Superimposition (done on protein backbones) of compounds (**12a**, **12b**, **12c**, **12f**, **12g**, and **12h**) bound to subunit B of *mm*PRMT4. Each PRMT4 subunit is represented as a cartoon (shades of gray, lime, cyan, marine, yellow, gray, and pink ribbons), and compounds are represented as sticks (in lime, yellow, cyan, cornflower blue, sea blue, and pink, respectively). (b) Close-up view of bound compound conformations. (c) Binding interactions of compound **12a** (lime sticks) with *mm*PRMT4 monomer B (ribbon). (d) Binding interactions of compound **12h** (pink sticks) with *mm*PRMT4 monomer B (ribbon). Hydrogen bonds are shown as dashed lines. For clarity, *N*-terminal helices (residues 135–165) of PRMT4 are not shown.

In the case of the complexes with PRMT4, all structures were solved and refined (depending on crystals, resolution ranged from 2.1 to 2.4 Å at ESRF or SOLEIL synchrotron beamlines) in the space group *P*2_1_2_1_2 and contain one copy of the PRMT4 tetramer in the asymmetric unit, as previously described.^106^ In all cases, each PRMT4 monomer binds one molecule of the ligand (Figure S2, Supporting Information). Crystallographic statistics are summarized in Table 4. The electron density maps obtained in the cocrystallization studies with compounds **12a**–**12c** and **12f**–**12h** reveal the conformation of each compound on all four monomers of the asymmetric unit (Figure 7). All six cocrystallized compounds (**12a**–**12c** and **12f**–**12h**) adopt an overall similar conformation in the complex with PRMT4, revealing two common anchoring platforms, the methyl 4-hydroxy-2-naphthoate and the adenosine moieties, each one occupying the same binding site on PRMT4 regardless of the different linker lengths (Figure 8a and b). On the contrary, distinctive conformations are observed for the linker in each compound (see below for details).

**Table 4:** X-ray Data Collection and Refinement Statistics for PRMT4 Complexes with Compounds **12a**–**12c** and **12f**–**12h**

ligand	**12a** (EML734)	**12b** (EML709)	**12c** (EML736)	**12f** (EML980)	**12g** (EML982)	**12h** (EML981)
PDB ID	7PV6	7PPY	7PPQ	7PU8	7PUQ	7PUC
data processing						
wavelength (Å)	0.968	0.979	0.979	0.980	0.980	0.980
resolution range (Å)^a^	48.40–2.40 (2.46–2.40)	42.53–2.42 (2.49–2.42)	45.80–2.10 (2.13–2.10)	48.07–2.19 (2.23–2.19)	48.01–2.09 (2.12–2.09)	46.06–2.19 (2.23–2.19)
space group	*P*2_1_2_1_2	*P*2_1_2_1_2	*P*2_1_2_1_2	*P*2_1_2_1_2	*P*2_1_2_1_2	*P*2_1_2_1_2
unit cell	75.0 99.5 208.5 90 90 90	74.7 98.8 207.0 90 90 90	74.8 98.5 206.9 90 90 90	75.2 98.8 208.4 90 90 90	75.1 98.7 207.8 90 90 90	75.3 98.9 208.2 90 90 90
total reflections	424165 (30752)	249037 (11755)	382899 (10013)	1073363 (40161)	1237125 (48566)	1072170 (46596)
unique reflections	61974 (4383)	58136 (3623)	89784 (3974)	80189 (3738)	92014 (4053)	80457 (3971)
multiplicity	6.8 (7.0)	4.3 (3.2)	4.3 (2.5)	13.4 (10.7)	13.4 (12.0)	13.3 (11.7)
completeness (%)	99.7 (96.8)	98.3 (80.0)	99.1 (86.7)	98.7 (81.5)	99.5 (90.2)	99.2 (87.0)
mean ⟨*I*/σI⟩^b^	11.3 (1.1)	8.8 (0.9)	9.2 (0.9)	14.4 (1.3)	17.8 (1.8)	11.1 (1.1)
resolution limit for ⟨*I*/σ*I*⟩ > 2^c^	2.63	2.69	2.33	2.24	2.13	2.39
Wilson B-factor	50.5	46.1	38.4	46.2	42.5	45.3
*R*_meas_	0.140 (2.177)	0.131 (1.441)	0.101 (1.282)	0.104 (1.678)	0.089 (1.387)	0.164 (2.950)
CC_1/2_	0.999 (0.547)	0.998 (0.441)	0.998 (0.416)	0.999 (0.543)	1.000 (0.831)	0.999 (0.511)
refinement						
resolution range	46.17–2.40 (2.49–2.40)	42.53–2.42 (2.51–2.42)	45.80–2.10 (2.17–2.10)	46.09–2.19 (2.27–2.19)	48.01–2.09 (2.16–2.09)	46.06–2.19 (2.27–2.19)
*R*_work_	0.191 (0.318)	0.205 (0.339)	0.194 (0.323)	0.194 (0.322)	0.181 (0.264)	0.202 (0.351)
*R*_free_	0.237 (0.338)	0.235 (0.359)	0.228 (0.351)	0.229 (0.344)	0.217 (0.304)	0.230 (0.364)
number of non-hydrogen atoms	11684	11303	11827	11483	11659	11408
macromolecules	11282	10971	11001	11000	11001	10993
ligands	455	436	398	368	410	342
solvent	165	104	614	283	442	223
validation						
RMS(bonds)	0.006	0.010	0.004	0.006	0.005	0.009
RMS(angles)	0.75	1.32	0.60	0.79	0.78	1.21
Ramachandran favored (%)	97.08	96.47	96.70	97.14	96.77	97.58
Ramachandran outliers (%)	0.00	0.00	0.00	0.00	0.00	0.00
rotamer outliers (%)	0.41	0.84	0.08	0.67	0.42	0.42
average B-factor	55.96	49.51	45.06	52.70	48.59	52.36
macromolecules	55.77	49.31	44.75	52.68	48.00	51.59
ligands	69.79	60.51	57.20	58.28	63.01	85.17
solvent	49.58	46.85	46.45	49.52	48.59	61.75
Clashscore	4.24	3.72	2.95	3.22	6.35	2.64

a Values in parentheses correspond to the highest-resolution shell.

b See ref (105) for crystallographic definitions.

c The resolution limits for ⟨*I*/σ*I*⟩ > 2 are reported.

As expected, the adenosine moiety occupies the SAM binding site, and as previously described,^106^ the main interactions are with Y150, E215, E244, and M269 (Figure 8c and d).

On the other hand, the methyl 4-hydroxy-2-naphthoate moiety partially occupies the peptide substrate binding site on PRMT4 and mainly interacts with Y262, P473, F475, and Y477 on one side and F153 on the other side (Figure 8c and d). Interestingly, superimposition of the conformation of **12h** cocrystallized with PRMT4 with the conformations of the PABP1 peptide transition state mimics recently developed by us^44^ allowed us to assess that the moiety occupies position −1 to −4 of the peptide substrate binding site, −1 being the position of the amino acid located at the N-terminal side of the arginine to be methylated (Figure S3, Supporting Information). Despite slight modifications observed among monomers inside a given tetramer, the crystal structures of compounds **12a**–**12c** and **12f**–**12h** revealed that the conformations of the protein side chains are identical for all compounds.

Surrounded by such a “frozen” binding site, the linker of each compound adopts a unique and distinctive conformation stabilized by interactions with the protein platform in a region mapped on one side by catalytic residues M260, E258, H415, and W416 and on the other side by F153, Y154, and E267 (Figure 8c and d and Figures S4 and S5, Supporting Information). Depending on the length and the nature of the linker, strong hydrogens bonds are established with such frozen sites, and the number and the strength of the binding interactions of each compound appear to be correlated with the affinity and, consequently, with the IC_50_ value. In fact, longer compounds **12f**–**12h** showed IC_50_ values in the low nM ranges compared to the μM range observed for shorter compounds **12a**–**12c**.

In the case of compound **12h**, the best inhibitor herein reported, the guanidine group lies between the catalytic glutamate residues (E258 and E267) of the double-E loop in the binding site of the guanidine moiety of the peptide transition state, and the N5 atom establishes two hydrogen bonds with the oxygen atoms of the main chain of E258 and M260 (Figure 8d and Figure S10, Supporting Information).

As revealed by the crystal structures, at least a two-carbon atom long linker between the adenosine moiety and the guanidine group is required to bring the latter within the catalytic clamp formed by E258 and E267, with a three-carbon atom linker (as in compounds **12g** and **12h**) being even better. If the linker is shorter (as in the case of compounds **12a**–**12c**), the two guanidine-stabilizing hydrogen bonds established with the PRMT4 main chain are lost, and this may account for a weaker affinity compared to derivatives **12f**–**12h**. This observation is in agreement with an improvement in inhibition capability observed for compounds **12f**–**12h** compared to compounds **12a**–**12c** (see Table 2 above). In addition, the trans conformation imposed by the double bond in **12h** is less constrained than the one adopted by **12g** (also featuring a three-carbon atom linker between the adenosine moiety and the guanidine group) and, therefore, more favorable.

Regarding the linker between the naphthylurea moiety and the guanidine group, a length increase from three carbon atoms (*n* = 1, **12a**) to four or five (*n* = 2, **12b**, and *n* = 3, **12c**, respectively) brings additional van der Waals interaction with W416 and Y262, thus substantiating an increase in affinity and a corresponding decrease in IC_50_ values.

In the case of the complexes with PRMT6, all structures were solved and refined (depending on crystals, resolution ranging from 1.65 to 2.3 Å) in the space group *P*2_1_ with one copy of the PRMT6 dimer in the asymmetric unit as previously described.^107^ Structure determinations and refinements revealed that only compounds **12a**, **12c**, and **12f** are visible in the active site of each monomer of the PRMT6 dimer.

**Figure 9:**
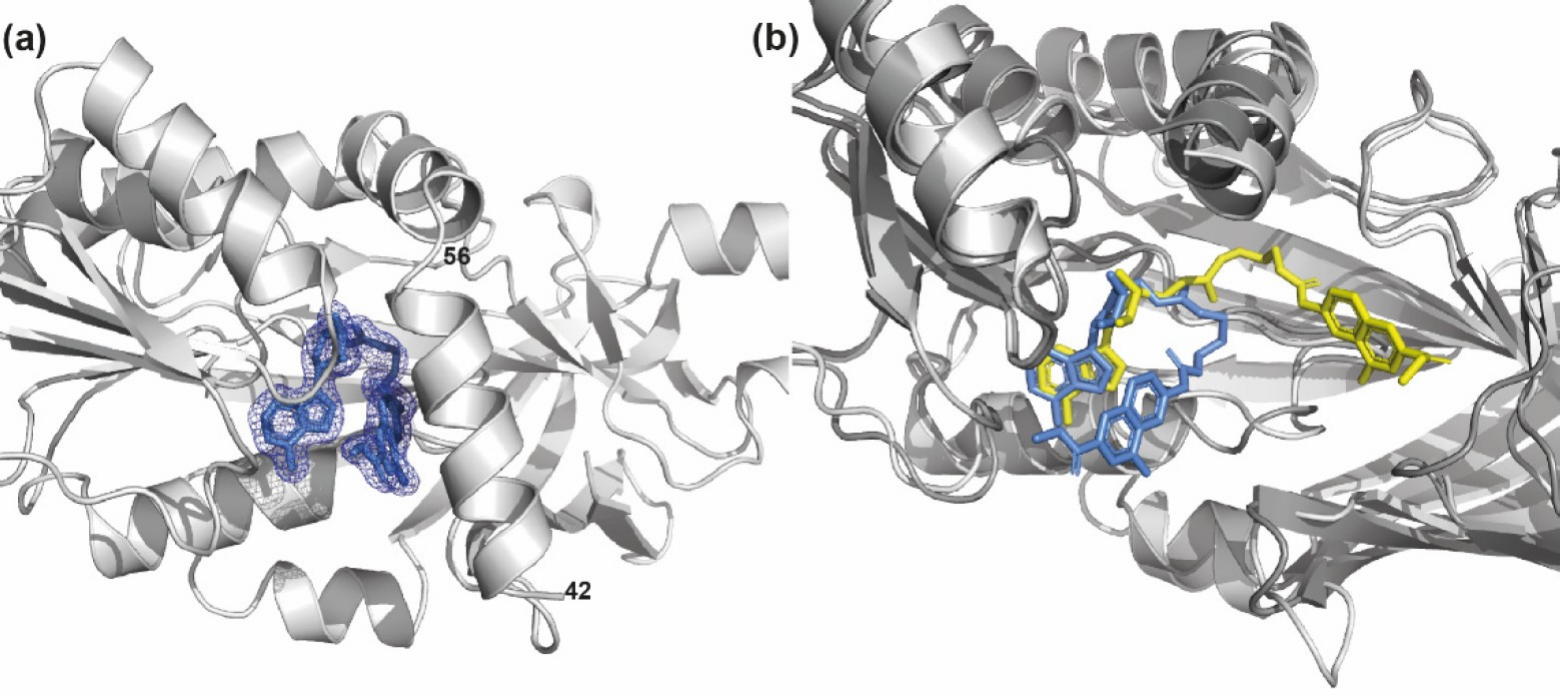
(a) Electron density (2*F*_obs_ – *F*_calc_) weighted maps of compound **12a** (represented as cornflower blue sticks) bound to subunit B of *mm*PRMT6 (represented as a gray cartoon; PDB ID: 7NUD). Maps are represented as a mesh, with the contouring level set to 1σ. For clarity, N-terminal helices (residues 42–56) of *mm*PRMT6 are not shown. (b) Superimposition (done on protein backbones) of the conformations of compound **12a** (yellow or cornflower blue sticks, respectively) when bound to subunit B of *mm*PRMT4 (represented as a dark gray cartoon) and when bound to *mm*PRMT6 (represented as a light gray cartoon). For clarity, N-terminal helices of PRMT4 and PRMT6 are not shown.

For the complexes with the other compounds, a molecule of SAH (constitutively contained in the purified *Mus musculus* PRMT6) was observed in the active site. Crystallographic statistics are summarized in Table 5. In all the structures of the complexes with PRMT6, the electron density for the compound is always better in one monomer. Compound **12a** is the only one for which a complete electron density is visible in one monomer of the PRMT6 structure (Figure 9), whereas for **12c** and **12f** the electron density becomes fragmented after the guanidine group and the density for the methyl-4-hydroxy-2-naphthoate moiety is weak. Moreover, for compound **12f**, the active site of one monomer is occupied by both the compound and SAH.

**Table 5:** X-ray Data Collection and Refinement Statistics for PRMT6 Complexes with Compounds **12a, 12b**, and **12f**

ligand	**12a** (EML734)	**12c** (EML736)	**12f** (EML980)
PDB ID	7NUD	7NUE	7P2R
data processing			
wavelength (Å)	1.54178	1.54178	1.54178
resolution range (Å)^a^	45.08–1.65 (1.68–1.65)	45.26–2.00 (2.05–2.00)	45.34–2.30 (2.39–2.30)
space group	*P*2_1_	*P*2_1_	*P*2_1_
unit cell (Å, deg)	41.8 118.1 72.0 90 104.3 90	41.8 118.5 71.9 90 103.1 90	41.8 118.7 72.1 90 102.8 90
total reflections	1494775 (29889)	174449 (10045)	119380 (8711)
unique reflections	80845 (4071)	45652 (3074)	30045 (2880)
multiplicity	18.5 (7.3)	3.8 (3.3)	4.0 (3.0)
completeness (%)	99.6 (99.8)	98.7 (91.4)	98.7 (90.6)
⟨*I*/σ*I*⟩^b^	14.9 (1.1)	14.4 (2.0)	7.2 (2.0)
resolution limit for ⟨*I*/σ*I*⟩ > 2^c^	1.72	2.00	2.30
Wilson B-factor (Å^2^)	21.3	27.9	35.2
*R*_meas_	0.123 (2.215)	0.112 (0.737)	0.130 (0.597)
CC_1/2_	0.999 (0.479)	0.996 (0.679)	0.989 (0.780)
refinement			
resolution range (Å)	37.46–1.65 (1.71–1.65)	38.53–2.0 (2–2.0)	45.34–2.3 (2–2.3)
% *R*_work_	0.1961 (0.2903)	0.1957 (0.2659)	0.1990 (0.2649)
% *R*_free_	0.2135 (0.3114)	0.2434 (0.3227)	0.2685 (0.3706)
number of non H atoms	5375	5341	5396
protein	5148	5126	5266
ligands	152	92	92
water	139	123	38
validation			
RMS(bonds)	0.008	0.008	0.008
RMS(angles)	1.04	0.99	1.03
Ramachandran favored (%)	98.61	97.83	98.18
Ramachandran outliers (%)	0.00	0.15	0.00
rotamer outliers (%)	0.55	0.55	0.90
average B-factor (Å^2^)	30.87	33.39	40.92
protein	30.69	33.02	40.59
ligands	43.30	58.06	62.86
water	29.82	30.33	32.21
Clashscore	3.49	4.08	3.32

a Values in parentheses correspond to the highest-resolution shell.

b See ref (105) for crystallographic definitions.

c The resolution limits for ⟨*I*/σ*I*⟩ > 2 are reported.

The crystal structure revealed an unusual distorted U-shaped conformation adopted by compound **12a** (and probably also **12c** and **12f**) in the binding to PRMT6, with a folding at the level of the guanidine group (Figure 9 and Figure S11, Supporting Information). Whereas the adenosine moiety lies into its canonic binding pocket, the guanidine group of the compounds is unable to reach the PRMT6 double-E loop clamp because the linker with the sugar is too short, even for the longer compound **12f**. Instead of binding in the arginine substrate pocket as observed with PRMT4 (Figures S2 and S8–S10, Supporting Information), the methyl-4 hydroxy-2-naphthoate moiety is sandwiched between the adenosine and the side chains of Y50 and Y51 residues of the α helix motif I (Figure S12, Supporting Information). Hence, the binding of compounds **12a**, **12c**, and **12f** affects the proper folding of the PRMT6 alpha-X helix containing the motif I (Y50-Y51-X-X-Y54).

If compared to the structures obtained in the presence of SAH, the binding site of the complex of PRMT6 with compound **12a** is larger due to a displacement of the α helix and the flipping of the side chains of Y50 and Y51 residues to give room for the compound naphthoate moiety. As a consequence, the E267 residue of the double-E loop is not coordinated anymore by residues Y50 and Y54 of motif 1 and adopts a different conformation (Figure S11, Supporting Information).

### Assessment of Functional Potency and Cell Toxicity in Various Cell Lines

**Figure 10:**
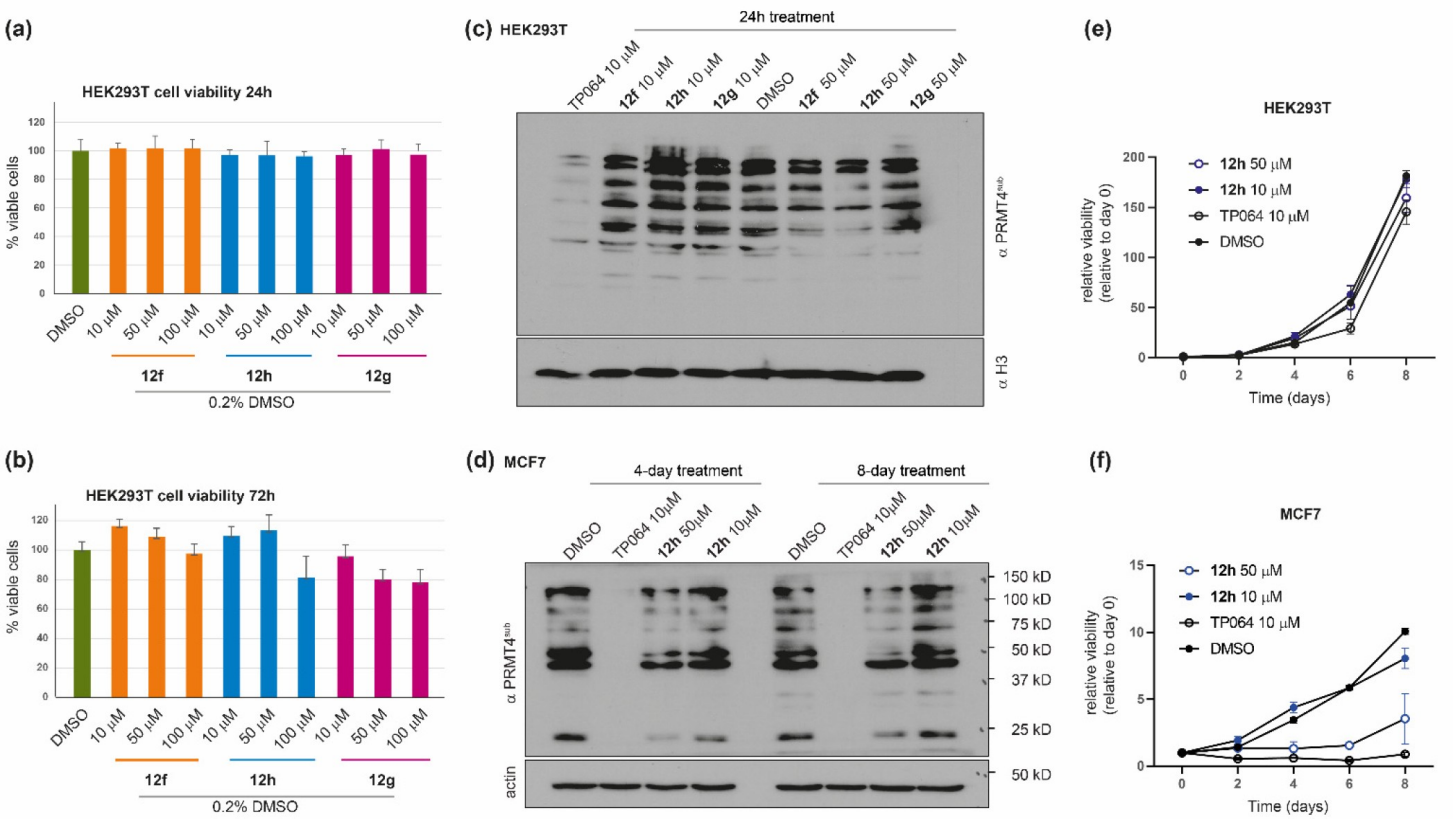
Cellular effects of compounds **12f**–**12h**. (a, b) The viability of HEK293T cells was assessed by measuring the mitochondrial-dependent reduction of MTT to formazan, with respect to DMSO, after treatment with compounds **12f**–**12h** at three different concentrations (10, 50, and 100 μM) for (a) 24 h and (b) 72 h. Data are reported as the mean ± SD of four independent experiments. (c, d) Western blot analyses were performed (a) on lysates from HEK293T cells after treatment with compounds **12f**–**12h** at 10 and 50 μM for 24 h and (d) on lysates from MCF7 cells after treatment with compound **12h** at 10 and 50 μM for 4 and 8 days. Methylation was detected by immunoblotting with a pan-PRMT4 substrate antibody (PRMT4^sub^; see the main text).^102^ Total histone H3 (c) or actin (d) was used to check for equal loading. The cell-permeable PRMT4 inhibitor TP064 (10 μM) was used as a reference compound. (e, f) Relative proliferation of (e) HEK293T and (f) MCF7 cells with different concentration of **12h** for different time points. The medium was changed at day 4. All the data points represent the relative viability normalized to day 0. The error bars represent the standard deviation of three biological replicates performed at each time point.

As mentioned above, this study was designed as a proof of concept of our approach to probe the structural differences among the various PRMTs and to develop potent and selective inhibitors. Therefore, we were not surprised to find that, at this stage, compounds **12f**–**12h** showed poor apparent permeability in a parallel artificial membrane permeability assay (PAMPA), using the reference drugs propranolol (highly permeable) and the furosemide (poorly permeable) as positive and negative controls, respectively (see the Supporting Information). Nonetheless, we resolve to investigate if, regardless of the low cell permeability, the compounds were able to affect the activity of PRMT4 in a cellular context. First, we assessed cell toxicity in human embryonic kidney HEK293T cells. To this aim, we incubated the cell line with different concentrations (10, 50, and 100 μM) of each compound and assessed cell viability after 24 and 72 h using the MTT assay. We observed that none among the tested compound was able to reduce the number of metabolically active cells in comparison with the vehicle, at all the tested concentrations (up to 100 μM, Figure 10a). Even after 72 h of treatment cell viability remained above 80% (Figure 10b).

We next investigated the functional potency of compounds **12f**–**12h** in reducing the cellular level of arginine methylation catalyzed by PRMT4. To this aim, HEK293T cells were incubated for 24 h with the three compounds (at 10 and 50 μM) or with compound TP-064 (10 μM)^24^ and used as a positive control, and total cell lysates were then immunoblotted with a pan-PRMT4 substrate antibody (PRMT4^sub^), originally raised against the R388 site of Nuclear Factor 1 B-type (NFIB-Me) but also capable of recognizing many PRMT4 substrates.^102,108^ As shown in Figure 10c, the results confirmed the proof of concept concerning the design of these derivatives. In fact, although with a lower effect compared to the cytopermeable TP-064, the compounds, in particular **12h**, are able to reduce the activity of PRMT4 in a concentration-dependent way. Noteworthy, TP-064 concentrations higher than 10 μM led to cell death. Based on these findings, we resolved to investigate the effects of compound **12h** also on the MCF7 breast cancer cell line, the proliferation of which was previously found to be dependent on PRMT4.^109,110^ MCF7 cells were incubated with **12h** (at 10 and 50 μM) or with the control TP-064 (10 μM) for 4 and 8 days used as positive control, and total cell lysates were then immunoblotted with the pan-PRMT4 substrate antibody PRMT4^sub^. As shown in Figure 10d, the arginine methylation levels in MCF7 cells are profoundly affected by TP064 but also by the significantly less permeable **12h**, in a concentration- and time-dependent way. More importantly, both 10 μM TP064 and 50 μM **12h** markedly decreased the proliferation of MCF7 cells, whereas no effect was observed in HEK293T cells (Figure 10e and f).

## Conclusions

The pivotal role played by PRMT-mediated arginine methylation in the regulation of many cellular processes and the implications in the genesis of various diseases have attracted growing interest toward PRMTs as potential therapeutic targets. Yet, even if a few clinical-grade small-molecule inhibitors have been identified for these proteins,^11^ there is still much to be understood about their functions and the biological pathways in which they are involved, as well as on the structural requirements that could drive the development of selective modulators of PRMT activity.^11^

In this work, starting from a series of type I PRMT inhibitors previously identified by us and with molecular modeling studies of their binding mode,^46,47^ we deconstructed such ligands into a 4-hydroxy-2-naphthoic fragment and then applied a step-by-step growing approach, in which the fragment was bridged by an amide or urea group with an arginine mimetic or methionine moiety. As a primary screening, we gauged the effect of the synthesized compounds (derivatives **5**–**11**) on the catalytic activity of human recombinant PRMT1, chosen as representative of class I PRMTs, using a purpose-developed AlphaLISA assay. The compounds showing an inhibition greater than 85% were selected, and the corresponding IC_50_ values were determined. The structure–activity relationships identified a scaffold (namely compound **7d**) featuring both naphthylurea and methylguanidine groups as the best candidate for the further growing step. Then, another series of compounds (**12a**–**12h**) were designed and synthesized, introducing also an adenosine moiety and exploring the distance between the three groups.

A radioisotope-based filter-binding assay was used as a secondary screening to profile the activity of the compounds against type I PRMT1, PRMT3, PRMT4, PRMT6, and PRMT8, type II PRMT5, and type III PRMT7. The overall length of the compounds and, even more, the length of the two linkers resulted to be crucial for the inhibitory activity especially against PRMT4 and, at a minor extent, against PRMT1. In fact, derivative **12h** featuring a four-carbon atom linker between the 4-hydroxy-2-naphthoate and the guanidine group and a three-carbon atom linker between the latter and the adenosine resulted to be the most potent inhibitor against PRMT4 (IC_50_ = 3 nM), with a 261- to 1266-fold selectivity over the other PRMTs. Noteworthy, **12h** was found to be selective also and further assessed against a panel of eight KMTs, including the SET-domain-containing proteins ASH1L/KMT2H, EZH2/KMT6, MLL1/KMT2A, SET7/9/KMT7, SETD8/KMT5A, SUV39H2/KMT1B, and SUV420H1/KMT5B and the non-SET-domain-containing DOT1L/KMT4, showing no inhibiting effect against these enzymes even at the higher tested concentration (>3000 fold higher than the IC_50_ value against PRMT4). SPR studies confirmed a specific and strong binding interaction between PRMT4 and **12h** with a *K*_D_ value in the nanomolar range (*K*_D_ = 75.9 nM; τ_R_ = 62.5 s), and crystallographic studies showed that the three-carbon atom long linker between the adenosine moiety and the guanidine group brings the latter within the catalytic clamp formed by E258 and E267 of the double-E loop, allowing the establishing of stabilizing hydrogen bonds between the guanidine N5 atom and the main chain oxygen atoms of E258 and M260. The trans conformation imposed by the double bond in **12h** impedes the formation of more constrained rotamers. On the other hand, the methyl 4-hydroxy-2-naphthoate moiety partially occupies the peptide substrate binding site on PRMT4 and mainly interacts with Y262, P473, F475, and Y477 on one side and F153 on the other side.

Whereas a similar trend was observed also against PRMT1 and PRMT5, the effect of the linker length on the inhibiting activity was less significant (if any) in the case of PRMT3, PRMT8, and, particularly, PRMT6. In the case of the latter, cocrystallization studies revealed that the compounds adopt an odd distorted U-shaped conformation, where the guanidine group is unable to reach the PRMT6 double-E loop clamp and the methyl-4 hydroxy-2-naphthoate moiety does not bind to the arginine substrate pocket, but it is sandwiched between the adenosine and the side chains of Y50 and Y51 residues of α helix motif I. Interestingly, in the case of PRMT7 the shorter compound **12a** shows a certain selective inhibition (IC_50_ = 0.3 μM; selectivity from 26-fold to 231-fold) compared to other tested PRMTs, in agreement with the restrictive and narrow active site for PRMT7.^111^

Surprisingly, regardless of the low cell permeability, **12h** is able to affect the activity of PRMT4 in a cellular context, showing an evident reduction of arginine methylation levels in MCF7 cells and a marked reduction of proliferation.

In conclusion, this study confirmed the feasibility of our deconstruction–reconstruction approach to achieve potency and selectivity against a specific PRMT isoform starting from nonselective PRMT inhibitors. Although nonoptimized for cell permeability, the identified PRMT4 inhibitor **12h** (EML981) is able to reduce the activity of PRMT4 in a concentration-dependent way and can be used for further development of cell-active PRMT4 inhibitors. Also, the approach is versatile and can be applied to identify selective inhibitors of other PRMTs.

## Chemistry

**Scheme 1:**
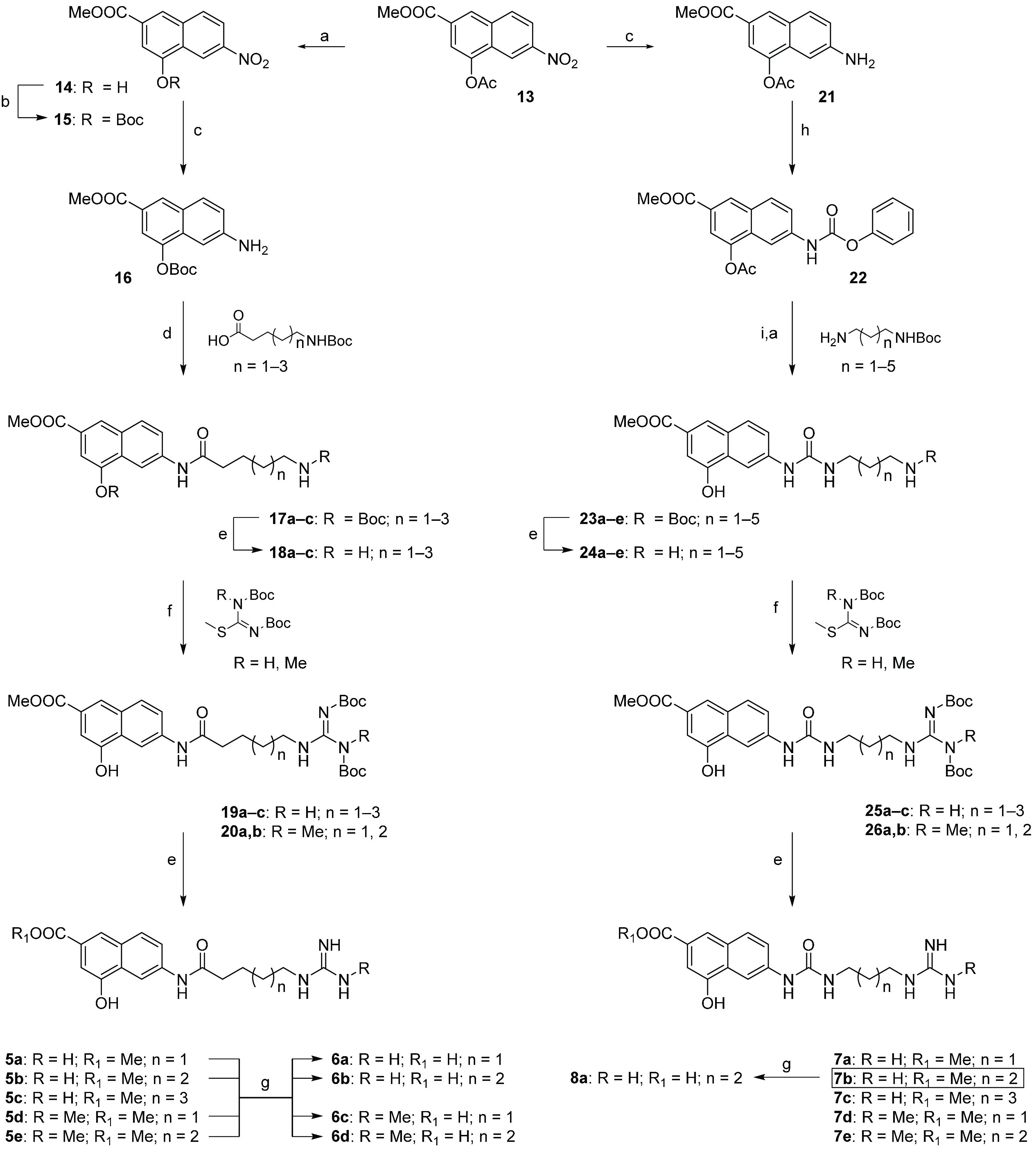
Synthesis of Derivatives **5a–5e, 6a–6d, 7a–7e**, and **8a** Reagents and conditions: (a) piperidine, DCM, room temperature (r.t.), 30 min. (93–99%); (b) Boc_2_O, TEA, DMAP, DCM, r.t., 12 h (70%); (c) Zn, AcOH, r.t., 1 h (98–99%; (d) DCC, DMAP, DCM, r.t., 8–12 h (80–88%); (e) DCM/TFA, 9:1, r.t., 1 h (60–99%); (f) TEA, DMAP, DMF, r.t., 24 h (30–85%); (g) LiOH, MeOH/H_2_O, r.t., 48 h (80–92%); (h) phenyl chloroformate, TEA, EtOAc, r.t., 12 h (70%); (i) TEA, DMF, r.t., 24 h (80–87%).

**Scheme 2:**
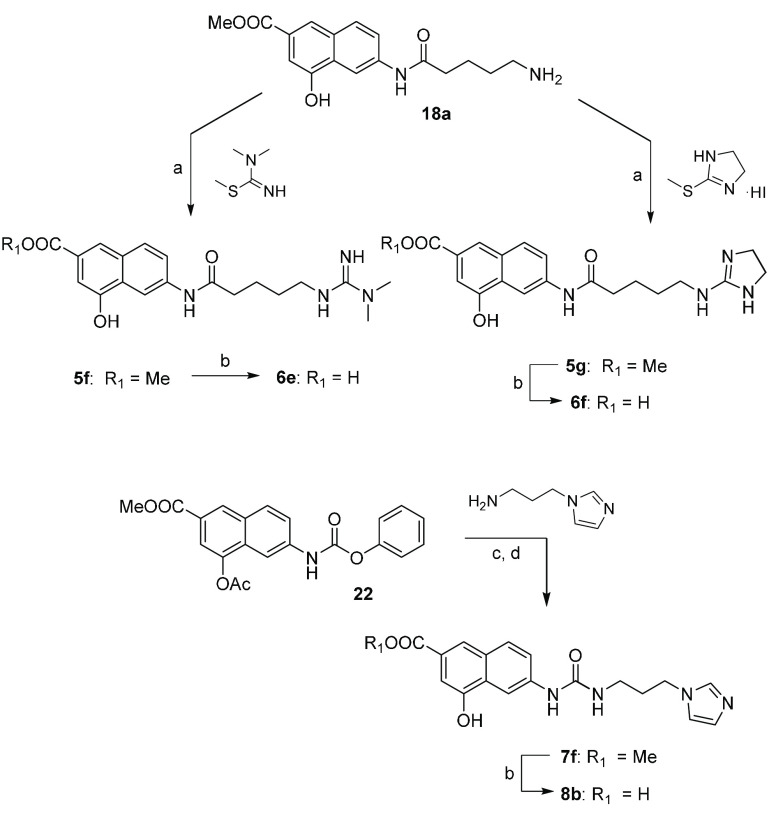
Synthesis of Derivatives **5f, 5g, 6e, 6f, 7f**, and **8b** Reagents and conditions: (a) TEA, DMAP, DMF, r.t., 24 h (57–67%); (b) LiOH, MeOH/H_2_O, r.t., 48 h (62–89%); (c) TEA, DMAP, DMF, r.t., 24 h; (d) piperidine, DCM, room temperature (r.t.), 30 min (71%, over two steps).

The synthetic protocol for the preparation of compounds **5**–**8** is depicted in Schemes 1 and 2. The 4-acetoxy-6-nitro-2-naphthoate **13**, prepared as previously reported by us,^47^ was straightforwardly transformed in the 4-hydroxy-6-nitro-2-naphthoate **14**,^112^ through treatment with piperidine in dichloromethane (DCM; Scheme 1). Protection of the hydroxyl group with di-*tert*-butyl dicarbonate (Boc_2_O) in the presence of triethylamine (TEA) and *N,N*-dimethyl-4-aminopyridine (DMAP) yielded the intermediate **15** which was then reduced with zinc dust in acetic acid to give the corresponding arylamine **16**. The reaction of the latter with the proper Boc-protected aminoalkanoic acid, in the presence of *N,N*′-dicyclohexylcarbodiimide (DCC) and DMAP, furnished amides **17a**–**17c**. After deprotection with trifluoroacetic acid (TFA) in DCM, the resulting amines **18a**–**18c** were reacted with Boc-protected *S*-methylisothiourea or *N,S*-dimethylisothiourea to yield the protected guanidines **19a**–**19c** and **20a** and **20b**. Removal of the *tert*-butoxycarbonyl group under acidic conditions gave ester derivatives **5a**–**5e**, from which the corresponding carboxylic acids **6a**–**6d** were obtained by hydrolysis with lithium hydroxide aqueous solution. A similar synthetic pathway was followed to prepare ureido derivatives **7a**–**7e** and **8a** (Scheme 1). Briefly, naphthylamine **21**, prepared from the same key building block **13** as previously reported by us,^47^ was reacted with phenyl chloroformate to yield phenyl carbamate **22**. The reaction of the latter with the proper mono-Boc-protected alkyldiamine, followed by treatment with piperidine in DCM, furnished derivatives **23a**–**23e**. After trifluoroacetic acid deprotection, the corresponding amines **24a**–**24e** were reacted with Boc-protected *S*-methylisothiourea or *N,S*-dimethylisothiourea to yield compounds **25a**–**25c**, **26a**, and **26b**. Finally, deprotection gave esters **7a**–**7e**. The carboxylic acid derivative **8a** was obtained by hydrolysis of ester **7b**. As depicted in Scheme 2, the amine **18a** was also reacted with 1,1,2-trimethylisothiourea to give the substituted guanidine derivative **5f** or with 2-methylthio-2-imidazoline hydroiodide to yield the imidazoline derivative **5g**. The carboxylic acids **6e** and **6f** were obtained from **5f** and **5g**, respectively, by hydrolysis with lithium hydroxide aqueous solution. Reaction of phenyl carbamate **22** with 3-imidazolylpropan-1-amine followed by treatment with piperidine in DCM furnished ester derivative **7f**. Finally, the carboxylic acid **8b** was obtained by basic hydrolysis.

**Scheme 3:**
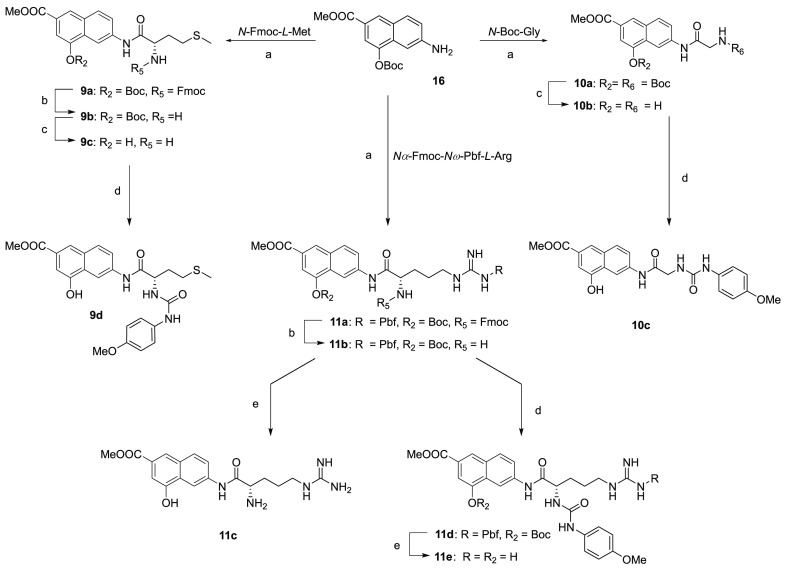
Synthesis of Derivatives **9**–**11** Reagents and conditions: (a) proper N-protected amino acid, DCC, DMAP, DCM, r.t., 8–12 h (79–81%); (b) piperidine, DCM, 30 min (72–99%); (c) DCM/TFA, 9:1, r.t., 1 h (68–74%); (d) 4-methoxyphenyl isocyanate, TEA, THF, r.t., 4 h (79–82%); (e) DCM/TFA, 1:9, r.t., 24 h (68–77%).

Derivatives **9**–**11** were in turn prepared (Scheme 3) starting from the *O*-Boc-aminohydroxynaphthoate **16** that was reacted with the orthogonally protected *N*-Fmoc-*l*-methionine, *N*-Boc-glycine, or *N*α-Fmoc-*N*ω-Pbf-*l*-arginine in the presence of DCC and DMAP to give the corresponding amides **9a**, **10a**, or **11a**. After deprotection with piperidine and/or TFA in DCM, the corresponding compounds **9c**, **10b**, and **11b** were reacted with 4-methoxyphenyl isocyanate in the presence of TEA to obtain ureas **9d**, **10c**, and **11d**. Derivatives **11c** and **11e** were obtained from **11b** and **11d**, respectively, after the removal of Pbf protection.

**Scheme 4:**
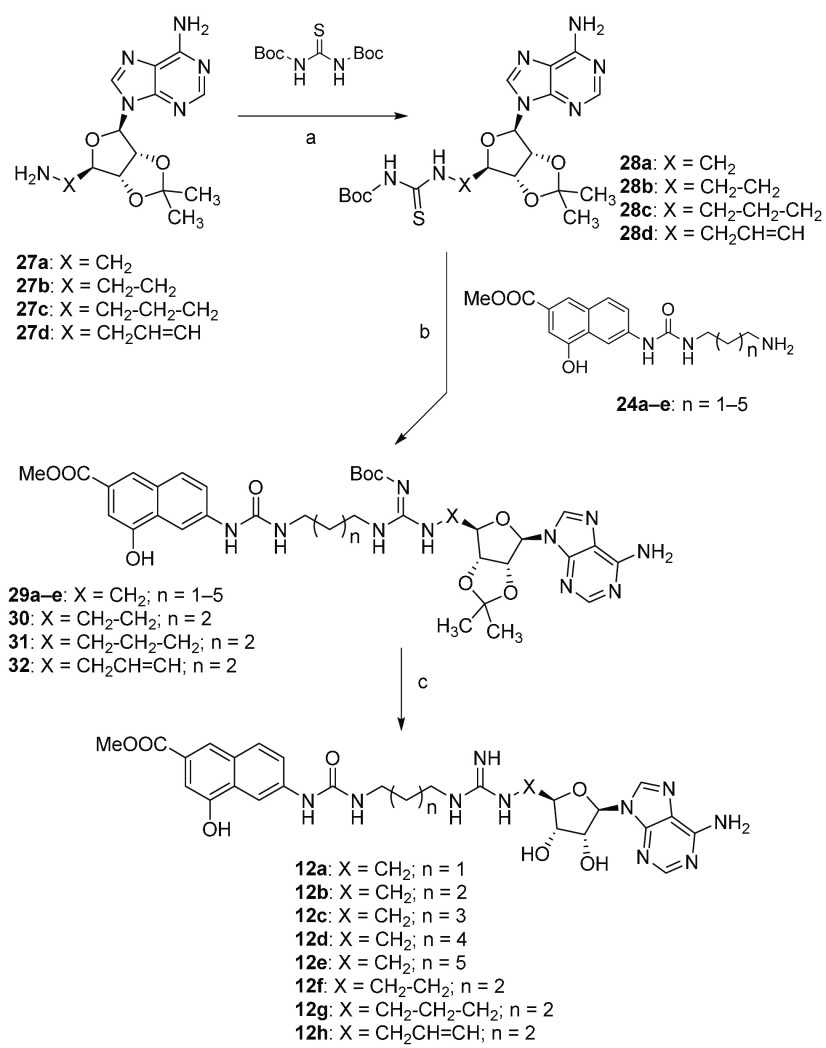
Synthesis of Derivatives **12a**–**12h** Reagents and conditions: (a) NaH 60% mineral oil, TFAA, dry THF, r.t., 16 h (33–53%); (b) EDC hydrochloride, TEA, DCM, r.t., 18 h (72–81%); (c) DCM/TFA, 1:1, r.t., 2 h (74–80%).

**Scheme 5:**
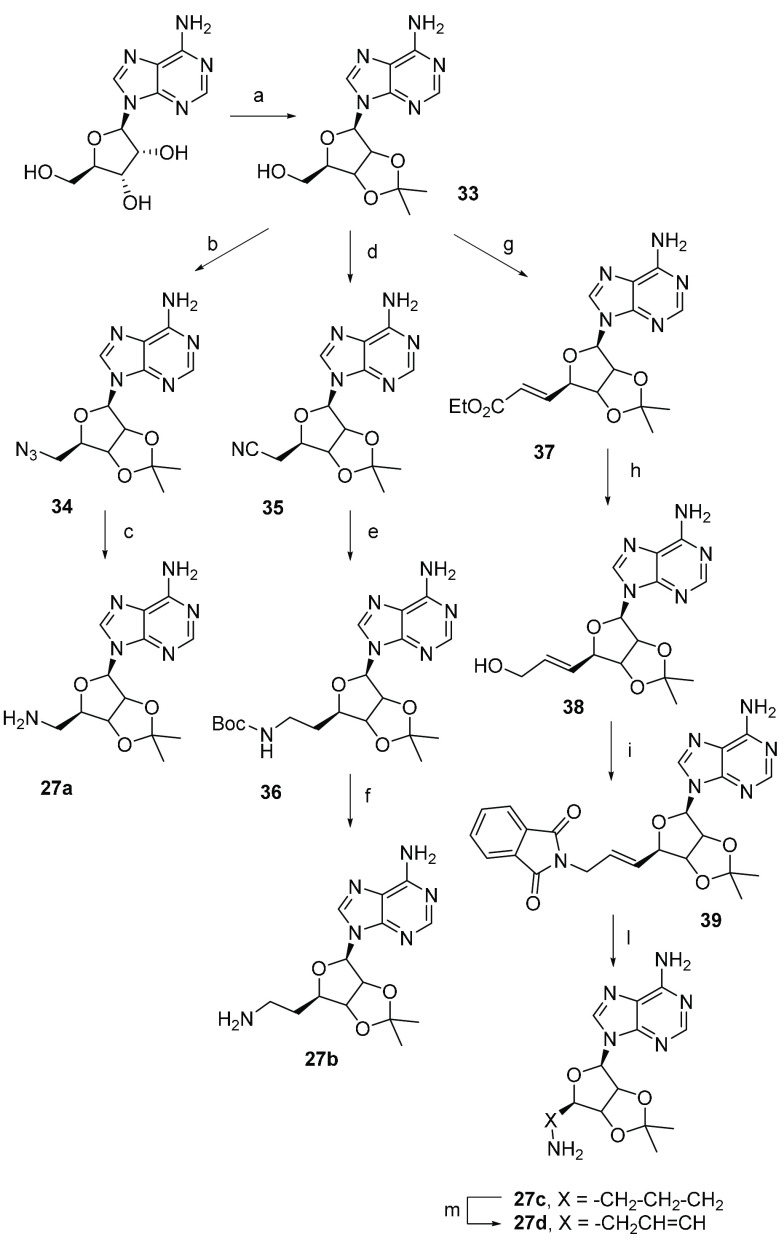
Synthesis of Derivatives **27a**–**27d** Reagents and conditions: (a) acetone, HClO_4_ 70%, r.t., 5 h (81%); (b) NaN_3_, DPPA, DBU, 15-crown-5, dioxane, 2 h (86%); (c) H_2_, Pd/C 10%, MeOH, r.t., 5 h (99%); (d) α-hydroxyisobutyronitrile, DEAD, PPh_3_, dry THF, 0–20 °C, 24 h, (89%); (e) Boc_2_O, NaBH_4_, NiCl_2_·6H_2_O, dry MeOH, 0 °C, 2 h (80%); (f) DCM/TFA 95:5, t.a, 8 h, (65%); (g) *o*-iodoxybenzoic acid (IBX), CH_3_CH_2_O_2_CCH=P(C_6_H_5_)_3_, DMSO, 20 °C, 72 h, (70%); (h) DIBAL-H, DCM, −78 °C, 2 h, (98%); (i) phthalimide, DEAD, PPH_3_, dry THF, 20 °C, 16 h, (70%); (l) NH_2_NH_2_·H_2_O, MeOH, 0–25 °C, 16 h, (90%); (m) H_2_, Pd/C 10%, AcOEt, 20 °C, 18 h (99%).

Compounds **12a**–**12h** were prepared as outlined in Scheme 4. Adenosine derivatives **27a**–**27d**,^44,113−115^ obtained according to a slight modification of previously reported procedures^116,117^ (Scheme 5), were reacted with *N,N*′-di-Boc-thiourea in the presence of trifluoroacetic anhydride (TFAA) and sodium hydride^118^ to give thioureas **28a**–**28d**. A coupling reaction with amino derivatives **24a**–**24e** in the presence of EDC hydrochloride as an activating agent yielded derivatives **29**–**32** which were deprotected with TFA to obtain the target compounds **12a**–**12h**.

## Experimental Section

### Chemistry

#### General Directions

All chemicals, purchased from Merck KGaA and Fluorochem Ltd., were of the highest purity. All solvents were reagent grade and, when necessary, were purified and dried by standard methods. All reactions requiring anhydrous conditions were conducted under a positive atmosphere of nitrogen in oven-dried glassware. Standard syringe techniques were used for anhydrous addition of liquids. Reactions were routinely monitored by TLC performed on aluminum-backed silica gel plates (Merck KGaA, Alufolien Kieselgel 60 F254) with spots visualized by UV light (λ = 254, 365 nm) or using a KMnO_4_ alkaline solution. Solvents were removed using a rotary evaporator operating at a reduced pressure of ∼10 Torr. Organic solutions were dried over anhydrous Na_2_SO_4_. Chromatographic purification was done on an automated flash-chromatography system (Isolera Dalton 2000, Biotage) using cartridges packed with KP-SIL, 60 Å (40–63 μm particle size). All microwave-assisted reaction were conducted in a CEM Discover SP microwave synthesizer equipped with a vertically focused IR temperature sensor.

Analytical high-performance liquid chromatography (HPLC) was performed on a Shimadzu SPD 20A UV/vis detector (λ = 220 and 254 nm) using a C-18 column Phenomenex Synergi Fusion-RP 80A (75 × 4.60 mm; 4 μm) at 25 °C using a mobile phase A (water + 0.1% TFA) and B (ACN + 0.1% TFA) at a flow rate of 1 mL/min. Preparative HPLC was performed using a Shimadzu Prominence LC-20AP with the UV detector set to 220 and 254 nm. Samples were injected onto a Phenomenex Synergi Fusion-RP 80A (150 × 21 mm; 4 mm) C-18 column at room temperature. Mobile phases of A (water + 0.1% TFA) and B (ACN + 0.1% TFA) were used with a flow rate of 20 mL/min.

^1^H spectra were recorded at 400 MHz on a Bruker Ascend 400 spectrometer while ^13^C NMR spectra were obtained by distortionless enhancement by polarization transfer quaternary (DEPTQ) spectroscopy on the same spectrometer. Chemical shifts are reported in δ (ppm) relative to the internal reference tetramethylsilane (TMS). Low-resolution mass spectra were recorded on a Finnigan LCQ DECA TermoQuest mass spectrometer in electrospray positive and negative ionization modes (ESI-MS). High-resolution mass spectra were recorded on a ThermoFisher Scientific Orbitrap XL mass spectrometer in electrospray positive ionization modes (ESI-MS). All tested compounds possessed a purity of at least 95% established by HPLC unless otherwise noted.

#### Methyl 6-(5-Guanidinopentanamido)-4-hydroxy-2-naphthoate (**5a**)

Compound **19a** (280 mg, 0.501 mmol) was dissolved in 10 mL of a solution of DCM/TFA (9:1), and the mixture was stirred for 48 h. The solvent was evaporated, and the resulting solid was washed with CHCl_3_ to give the TFA salt of compound **5a** as a brown solid (201 mg, 85%). ^1^H NMR (400 MHz, DMSO-*d*_6_) δ 10.45 (s, 1H, exchangeable with D_2_O), 10.24 (s, 1H, exchangeable with D_2_O), 8.54 (s, 1H), 8.01–7.95 (m, 2H), 7.72 (d, *J* = 8.7 Hz, 1H), 7.49 (br t, 1H, exchangeable with D_2_O), 7.34 (s, 1H), 7.21–6.75 (m, 3H, exchangeable with D_2_O), 3.88 (s, 3H), 3.18–3.12 (m, 2H), 2.42 (t, *J* = 7.2 Hz, 2H), 1.68–1.53 (m, 4H); ^13^C NMR (101 MHz, DMSO-*d*_6_) δ 171.96, 167.02, 156.91, 153.20, 138.43, 130.18, 130.14, 127.79, 126.06, 121.49, 121.09, 110.05, 106.93, 40.90, 40.42, 36.21, 28.35, 22.48. HRMS (ESI): *m*/*z* [M + H]^+^ calcd for C_18_H_22_N_4_O_4_ + H^+^: 359.1714. Found: 359.1705.

#### Methyl 6-(6-Guanidinohexanamido)-4-hydroxy-2-naphthoate (**5b**)

The TFA salt of compound **5b** was obtained as a pale brown solid (254 mg, 81%) starting from compound **19b** (370 mg, 0.646 mmol) following the procedure described for **5a**. ^1^H NMR (400 MHz, DMSO-*d*_6_) δ 10.43 (s, 1H, exchangeable with D_2_O), 10.20 (s, 1H, exchangeable with D_2_O), 8.54 (s, 1H), 8.00 (s, 1H), 7.97 (d, *J* = 8.5 Hz, 1H), 7.72 (d, *J* = 8.5 Hz, 1H), 7.50 (br t, 1H, exchangeable with D_2_O), 7.33 (s, 1H), 7.22–6.83 (m, 3H, exchangeable with D_2_O), 3.87 (s, 3H), 3.12–3.10 (m, 2H), 2.39 (t, *J* = 6.9 Hz, 2H), 1.67–1.63 (m, 2H), 1.53–1.49 (m, 2H), 1.37–1.33 (m, 2H); ^13^C NMR (101 MHz, DMSO-*d*_6_) δ 171.94, 167.05, 157.18, 153.45, 138.77, 130.27, 130.19, 128.00, 126.10, 121.55, 121.13, 110.05, 107.10, 52.51, 41.13, 36.82, 28.84, 26.23, 25.13. HRMS (ESI): *m*/*z* [M + H]^+^ calcd for C_19_H_24_N_4_O_4_ + H^+^: 373.1870. Found: 373.1863.

#### Methyl 6-(7-Guanidinoheptanamido)-4-hydroxy-2-naphthoate (**5c**)

The TFA salt of compound **5c** was obtained as a pale brown solid (76 mg, 90%) starting from **19c** (100 mg, 0.170 mmol) following the procedure described for **5a**. ^1^H NMR (400 MHz, DMSO-*d*_6_) δ 10.45 (s, 1H, exchangeable with D_2_O), 10.20 (s, 1H, exchangeable with D_2_O), 8.54 (s, 1H), 8.01 (s, 1H), 7.95 (d, *J* = 8.9 Hz, 1H) 7.71 (d, *J* = 8.9 Hz, 1H), 7.55 (br t, 1H, exchangeable with D_2_O), 7.33 (s, 1H), 7.23–6.77 (m, 3H, exchangeable with D_2_O), 3.87 (s, 3H), 3.12–3.07 (m, 2H), 2.38 (t, *J* = 7.3 Hz, 2H), 1.65–1.62 (m, 2H), 1.51–1.47 (m, 2H), 1.35–1.33 (m, 4H); ^13^C NMR (101 MHz, DMSO-*d*_6_) δ 172.04, 167.05, 157.22, 153.45, 138.79, 130.26, 130.19, 128.01, 126.09, 121.55, 121.15, 110.05, 107.09, 52.50, 41.21, 36.88, 28.81, 28.75, 26.39, 25.44. HRMS (ESI): *m*/*z* [M + H]^+^ calcd for C_20_H_26_N_4_O_4_ + H^+^: 387.2027. Found: 387.2017.

#### Methyl 4-Hydroxy-6-(5-(3-methylguanidino)pentanamido)-2-naphthoate (**5d**)

The TFA salt of compound **5d** was obtained as a brown solid (204 mg, 86%) starting from **20a** (280 mg, 0.489 mmol) following the procedure described for **5a**. ^1^H NMR (400 MHz, DMSO-*d*_6_) δ 10.47 (s, 1H, exchangeable with D_2_O), 10.28 (s, 1H, exchangeable with D_2_O), 8.55 (s, 1H), 8.00 (s, 1H) 7.96 (d, *J* = 8.9 Hz, 1H), 7.72 (d, *J* = 8.7 Hz, 1H), 7.54 (br t, 1H, exchangeable with D_2_O), 7.45 (s, 1H, exchangeable with D_2_O), 7.36–7.34 (m, 3H, 2H exchangeable with D_2_O), 3.87 (s, 3H), 3.18–3.14 (m, 2H), 2.75–2.74 (m, 3H), 2.44–2.40 (m, 2H), 1.68–1.53 (m, 4H); ^13^C NMR (101 MHz, DMSO-*d*_6_) δ 171.85, 167.09, 156.80, 153.47, 138.73, 130.29, 130.20, 128.01, 126.12, 121.53, 121.15, 110.10, 107.11, 52.51, 41.13, 36.42, 28.64, 28.39, 22.67. HRMS (ESI): *m*/*z* [M + H]^+^ calcd for C_19_H_24_N_4_O_4_ + H^+^: 373.1870. Found: 373.1873.

#### Methyl 4-Hydroxy-6-(6-(3-methylguanidino)hexanamido)-2-naphthoate (**5e**)

The TFA salt of compound **5e** was obtained as a pale brown solid (279 mg, 80%) starting from **20b** (410 g, 0.699 mmol) following the procedure described for **5a**. ^1^H NMR (400 MHz, DMSO-*d*_6_) δ 10.48 (s, 1H, exchangeable with D_2_O), 10.24 (s, 1H, exchangeable with D_2_O), 8.55 (s, 1H), 8.01 (s, 1H), 7.96 (d, *J* = 8.5 Hz, 1H), 7.72 (d, *J* = 8.5 Hz, 1H), 7.47 (t, *J* = 5.4 Hz, 1H, exchangeable with D_2_O), 7.40 (s, 1H, exchangeable with D_2_O), 7.34–7.32 (m, 3H, 2H exchangeable with D_2_O), 3.87 (s, 3H), 3.13–3.09 (m, 2H), 2.73 (s, 3H), 2.39 (t, *J* = 7.3 Hz, 2H), 1.66–1.63 (m, 2H), 1.53–1.50 (m, 2H), 1.36–1.33 (m, 2H); ^13^C NMR (101 MHz, DMSO-*d*_6_) δ 171.96, 167.05, 156.73, 153.45, 138.77, 130.25, 130.20, 128.00, 126.10, 121.55, 121.12, 110.05, 107.10, 52.51, 41.23, 36.82, 28.86, 28.40, 26.22, 25.14. HRMS (ESI): *m*/*z* [M + H]^+^ calcd for C_20_H_26_N_4_O_4_ + H^+^: 387.2027. Found: 387.2020.

#### Methyl 6-(5-(3,3-Dimethylguanidino)pentanamido)-4-hydroxy-2-naphthoate (**5f**)

To a stirred solution of **18a** (200 mg, 0.464 mmol) and DMAP (6 mg, 0.046 mmol) in dry DMF (2.5 mL), TEA (65 μL, 0.464 mmol) and 1,3-bis(*tert*-butoxycarbonyl)-2-methyl-2-thiopseudourea (228 mg, 0.929 mmol) were added. The resulting mixture was stirred at room temperature for 24 h. The solution was diluted with AcOEt (30 mL) and brine (10 mL), dried over Na_2_SO_4_, filtered, and concentrated under reduced pressure. The crude product was purified by flash chromatography (gradient Hex/AcOEt 80:20 to 20:80) to give **5f** as a white solid (268 mg, 67%). ^1^H NMR (400 MHz, DMSO-*d*_6_) δ 10.46 (s, 1H, exchangeable with D_2_O), 10.24 (s, 1H, exchangeable with D_2_O), 8.55 (d, *J* = 2.1 Hz, 1H), 8.01 (s, 1H), 7.96 (d, *J* = 8.9 Hz, 1H), 7.71 (dd, *J* = 8.9, 2.1 Hz, 1H), 7.42 (s, 1H, exchangeable with D_2_O), 7.37 (t, 1H, exchangeable with D_2_O), 7.36–7.34 (m, 1H), 3.87 (s, 3H), 3.23–3.18 (m, 2H), 2.96 (s, 6H), 2.42 (t, *J* = 7.0 Hz, 2H), 1.68–1.60 (m, 2H), 1.60–1.57 (m, 2H). ^13^C NMR (101 MHz, DMSO-*d*_6_) δ 171.87, 167.05, 156.20, 153.46, 138.72, 130.30, 130.22, 127.99, 126.14, 121.55, 121.13, 110.09, 107.11, 52.51, 42.13, 38.60, 36.47, 28.67, 22.63. HRMS (ESI): *m*/*z* [M + H]^+^ calcd for C_20_H_26_N_4_O_4_ + H^+^: 387.2027. Found: 387.2019.

#### Methyl 6-(5-((4,5-Dihydro-1*H*-imidazol-2-yl)amino)pentanamido)-4-hydroxy-2-naphthoate (**5g**)

Compound **5g** was obtained as a white product (136 mg, 61%) by the reaction of compound **18a** (250 mg, 0.58 mmol) with 2-methylthio-2-imidazoline hydroiodide (283 mg, 1.16 mmol) following the procedure described for **5f**. ^1^H NMR (400 MHz, DMSO-*d*_6_) δ 10.45 (s, 1H, exchangeable with D_2_O), 10.23 (s, 1H, exchangeable with D_2_O), 8.54 (d, *J* = 2.1 Hz, 1H), 8.30 (br t, 1H, exchangeable with D_2_O), 8.00 (s, 1H), 7.95 (d, *J* = 8.9 Hz, 1H), 7.70 (dd, *J* = 8.9, 2.1 Hz, 1H), 7.33 (s, 1H), 4.27–3.97 (m, 4H), 3.87 (s, 3H), 3.19–3.14 (m, 2H), 2.41 (t, *J* = 7.1 Hz, 2H), 1.66–1.60 (m, 2H), 1.60–1.51 (m, 2H); ^13^C NMR (101 MHz, DMSO-*d*_6_) δ 171.79, 167.04, 159.87, 153.45, 138.70, 130.30, 130.22, 127.99, 126.14, 121.55, 121.13, 110.09, 107.11, 52.51, 42.92, 42.49, 36.39, 28.85, 22.62. HRMS (ESI): *m*/*z* [M + H]^+^ calcd for C_20_H_24_N_4_O_4_ + H^+^: 385.1870. Found: 385.1863.

#### 6-(5-Guanidinopentanamido)-4-hydroxy-2-naphthoic Acid (**6a**)

To a stirred solution of **5a** (100 mg, 0.211 mmol) in 4 mL of MeOH, an aqueous solution (1 mL) of LiOH (15 mg, 0.633 mmol) was added. The reaction mixture was stirred at room temperature for 48–72 h (TLC analysis). The resulting mixture was acidified with 1 N HCl and then concentrated under vacuum. The crude product was purified by reversed-phase high-performance liquid chromatography (RP-HPLC) to afford **6a** as the TFA salt (89 mg, 92%). ^1^H NMR (400 MHz, DMSO-*d*_6_) δ 12.81 (s, 1H, exchangeable with D_2_O), 10.38 (s, 1H, exchangeable with D_2_O), 10.23 (s, 1H, exchangeable with D_2_O), 8.54 (s, 1H), 7.99–7.93 (m, 2H), 7.70 (d, *J* = 8.6 Hz, 1H), 7.53 (br t, 1H, exchangeable with D_2_O), 7.34 (s, 1H), 7.22–6.75 (m, 3H, exchangeable with D_2_O), 3.19–3.10 (m, 2H), 2.43 (t, *J* = 7.4 Hz, 2H), 1.69–1.53 (m, 4H); ^13^C NMR (101 MHz, DMSO-*d*_6_) δ 171.26, 167.64, 156.71, 152.78, 137.95, 129.86, 129.61, 127.30, 121.07, 120.48, 109.65, 107.07, 40.54, 35.91, 28.13, 22.17. HRMS (ESI): *m*/*z* [M + H]^+^ calcd for C_17_H_20_N_4_O_4_ + H^+^: 345.1557. Found: 345.1549.

#### 6-(6-Guanidinohexanamido)-4-hydroxy-2-naphthoic Acid (**6b**)

The TFA salt of compound **6b** was obtained as a white solid (149 mg, 77%) starting from **5b** (200 mg, 0.411 mmol) following the procedure described for **6a**. ^1^H NMR (400 MHz, DMSO-*d*_6_) δ 12.79 (s, 1H, exchangeable with D_2_O), 10.38 (s, 1H, exchangeable with D_2_O), 10.21 (s, 1H, exchangeable with D_2_O), 8.54 (s, 1H), 7.97–7.92 (m, 2H), 7.69 (d, *J* = 8.0 Hz, 1H), 7.59 (br t 1H, exchangeable with D_2_O), 7.33 (s, 1H), 7.17–6.77 (m, 3H, exchangeable with D_2_O), 3.12–3.09 (m, 2H), 2.39 (t, *J* = 7.1 Hz, 2H),1.67–1.64 (m, 2H), 1.53–1.50 (m, 2H), 1.37–1.34 (m, 2H); ^13^C NMR (101 MHz, DMSO-*d*_6_) δ 172.08, 168.13, 157.06, 153.24, 138.35, 130.33, 130.13, 127.72, 127.26, 121.61, 120.95, 110.14, 107.51, 41.13, 36.74, 28.76, 26.18, 25.13. HRMS (ESI): *m*/*z* [M + H]^+^ calcd for C_18_H_22_N_4_O_4_ + H^+^: 359.1714. Found: 359.1707.

#### 4-Hydroxy-6-(5-(3-methylguanidino)pentanamido)-2-naphthoic Acid (**6c**)

The TFA salt of compound **6c** was obtained as a pale brown solid (84 mg, 88%) starting from **5d** (100 mg, 0.204 mmol) following the procedure described for **6a**. ^1^H NMR (400 MHz, DMSO-*d*_6_) δ 12.79 (s, 1H, exchangeable with D_2_O), 10.37 (s, 1H, exchangeable with D_2_O), 10.22 (s, 1H, exchangeable with D_2_O), 8.54 (s, 1H), 7.99–7.93 (m, 2H), 7.70 (d, *J* = 8.5 Hz, 1H), 7.39–7.30 (m, 5H, 4H exchangeable with D_2_O), 3.19–3.14 (m, 2H), 2.76 (s, 3H), 2.45–2.40 (m 2H), 1.70–1.53 (m, 4H); ^13^C NMR (101 MHz, DMSO-*d*_6_) δ 171.87, 168.10, 138.28, 130.35, 130.16, 127.75, 127.27, 121.63, 121.05, 110.17, 107.46, 41.02, 36.34, 28.55, 28.23, 22.63. HRMS (ESI): *m*/*z* [M + H]^+^ calcd for C_18_H_22_N_4_O_4_ + H^+^: 359.1714. Found: 359.1709.

#### 4-Hydroxy-6-(6-(3-methylguanidino)hexanamido)-2-naphthoic Acid (**6d**)

The TFA salt of compound **6d** was obtained as a white solid (155 mg, 80%) starting from **5e** (200 mg, 0.399 mmol) following the procedure described for **6a**. ^1^H NMR (400 MHz, DMSO-*d*_6_) δ 12.75 (s, 1H, exchangeable with D_2_O), 10.37 (s, 1H, exchangeable with D_2_O), 10.26 (s, 1H, exchangeable with D_2_O), 8.55 (s, 1H), 7.97–7.92 (m, 2H), 7.72 (d, *J* = 7.9 Hz, 1H), 7.50 (br t, 1H,, exchangeable with D_2_O) 7.50–7.34 (m, 4H, 3H exchangeable with D_2_O), 3.15–3.10 (m, 2H), 2.74 (s, 3H), 2.40 (t, *J* = 7.4 Hz, 2H), 1.68–1.64 (m, 2H), 1.55–1.51 (m, 2H), 1.39–1.36 (m, 2H); ^13^C NMR (101 MHz, DMSO-*d*_6_) δ 171.97, 168.15, 156.89, 153.29, 138.55, 130.31, 130.03, 127.82, 127.29, 121.54, 121.01, 110.11, 107.56, 41.20, 36.82, 28.84, 28.38, 26.21, 25.17. HRMS (ESI): *m*/*z* [M + H]^+^ calcd for C_19_H_24_N_4_O_4_ + H^+^: 373.1870. Found: 373.1862.

#### 6-(5-(3,3-Dimethylguanidino)pentanamido)-4-hydroxy-2-naphthoic Acid (**6e**)

Compound **6e** was obtained as a pale brown solid (74 mg, 62%) starting from **5f** (95 mg, 0.245 mmol) following the procedure described for **6a**. ^1^H NMR (400 MHz, DMSO-*d*_6_) δ 10.37 (s, 1H, exchangeable with D_2_O), 10.21 (s, 1H, exchangeable with D_2_O), 8.53 (d, *J* = 2.1 Hz, 1H), 7.97 (s, 1H), 7.93 (d, *J* = 8.9 Hz, 1H), 7.70 (dd, *J* = 8.9, 2.2 Hz, 1H), 7.41 (s, 1H, exchangeable with D_2_O), 7.37 (br t, 1H, exchangeable with D_2_O), 7.33 (s, 1H), 3.23–3.18 (m, 2H), 2.42 (t, *J* = 7.0 Hz, 2H), 1.70–1.63 (m, 2H), 1.61–1.53 (m, 2H). ^13^C NMR (101 MHz, DMSO-*d*_6_) δ 171.81, 168.13, 156.20, 153.27, 138.45, 130.35, 130.10, 127.80, 127.35, 121.57, 120.97, 110.14, 107.56, 42.13, 38.60, 36.47, 28.67, 22.65. HRMS (ESI): *m*/*z* [M + H]^+^ calcd for C_19_H_24_N_4_O_4_ + H^+^: 373.1870. Found: 373.1865.

#### 6-(5-((4,5-Dihydro-1*H*-imidazol-2-yl)amino)pentanamido)-4-hydroxy-2-naphthoic Acid (**6f**)

Compound **6f** was obtained as a pale brown solid (84 mg, 79%) starting from **5g** (85 mg, 0.221 mmol) following the procedure described for **6a**. ^1^H NMR (400 MHz, DMSO-*d*_6_) δ 12.78 (s, 1H, exchangeable with D_2_O), 10.37 (s, 1H, exchangeable with D_2_O), 10.21 (s, 1H, exchangeable with D_2_O), 8.53 (d, *J* = 2.1 Hz, 1H), 8.31 (br t, 1H, exchangeable with D_2_O), 7.97 (s, 1H), 7.93 (d, *J* = 8.9 Hz, 1H), 7.70 (dd, *J* = 8.9, 2.1 Hz, 1H), 7.33 (s, 1H), 3.67–3.54 (m, 4H), 3.20–3.15 (m, 2H), 2.41 (t, *J* = 7.1 Hz, 2H), 1.69–1.59 (m, 2H), 1.59–1.52 (m, 2H); ^13^C NMR (101 MHz, DMSO-*d*_6_) δ 171.74, 168.13, 159.87, 153.27, 138.44, 130.35, 130.10, 127.80, 127.35, 121.56, 120.97, 110.15, 107.56, 42.92, 42.49, 36.39, 28.85, 22.63. HRMS (ESI): *m*/*z* [M + H]^+^ calcd for C_19_H_22_N_4_O_4_ + H^+^: 371.1714. Found: 371.1706.

#### Methyl 6-(3-(3-Guanidinopropyl)ureido)-4-hydroxy-2-naphthoate (**7a**)

The TFA salt of compound **7a** (36 mg, 99%) was obtained starting from **25a** (43 mg, 0.077 mmol) following the procedure described for **5a**. ^1^H NMR (400 MHz, DMSO-*d*_6_) δ 10.26 (s, 1H, exchangeable with D_2_O), 8.94 (s, 1H, exchangeable with D_2_O), 8.24 (d, *J* = 2.2 Hz, 1H), 7.93 (s, 1H), 7.85 (d, *J* = 9.0 Hz, 1H), 7.54–7.47 (m, 2H, 1H exchangeable with D_2_O), 7.26 (s, 1H), 7.07 (br s, 3H, exchangeable with D_2_O), 6.40 (br t, 1H, exchangeable with D_2_O), 3.82 (s, 3H), 3.16–3.11 (m, 4H), 1.70–1.58 (m, 2H); ^13^C NMR (101 MHz, DMSO-*d*_6_) δ 166.62, 156.75, 155.32, 152.54, 139.72, 129.70, 128.77, 127.91, 124.63, 121.23, 120.16, 107.22, 106.46, 51.92, 38.44, 36.45, 29.39. HRMS (ESI): *m*/*z* [M + H]^+^ calcd for C_17_H_21_N_5_O_4_ + H^+^: 360.1666. Found: 360.1659.

#### Methyl 6-(3-(4-Guanidinobutyl)ureido)-4-hydroxy-2-naphthoate (**7b**)

The TFA salt of compound **7b** was obtained as a yellow solid (39 mg, 97%) starting from compound **25b** (49 mg, 0.082 mmol) following the procedure described for **5a**. ^1^H NMR (400 MHz, DMSO-*d*_6_) δ 10.30 (s, 1H, exchangeable with D_2_O), 8.96 (s, 1H, exchangeable with D_2_O), 8.28 (d, *J* = 2.2 Hz, 1H), 7.96 (s, 1H), 7.88 (d, *J* = 8.9 Hz, 1H), 7.59–7.52 (m, 2H, 1H exchangeable with D_2_O), 7.29 (s, 1H), 7.06 (br s, 3H, exchangeable with D_2_O), 6.44 (br t, 1H, exchangeable with D_2_O), 3.85 (s, 3H), 3.23–3.03 (m, 4H), 1.60–1.39 (m, 4H). ^13^C NMR (101 MHz, DMSO-*d*_6_) δ 166.63, 156.67, 155.18, 152.52, 139.79, 129.70, 128.72, 127.93, 124.58, 121.23, 120.10, 107.08, 106.45, 51.92, 27.04, 26.00. HRMS (ESI): *m*/*z* [M + H]^+^ calcd for C_18_H_23_N_5_O_4_ + H^+^: 374.1823. Found: 374.1816.

#### Methyl 6-(3-(5-Guanidinopentyl)ureido)-4-hydroxy-2-naphthoate (**7c**)

The TFA salt of compound **7c** was obtained as a yellow solid (77 mg, 90%) starting from compound **25c** (100 mg, 0.170 mmol) following the procedure described for **5a**. ^1^H NMR (400 MHz, DMSO-*d*_6_) δ 10.29 (s, 1H, exchangeable with D_2_O), 8.91 (s, 1H, exchangeable with D_2_O), 8.28 (d, *J* = 2.2 Hz, 1H), 7.97 (s, 1H), 7.88 (d, *J* = 8.9 Hz, 1H), 7.56–7.52 (m, 2H, 1H, exchangeable with D_2_O), 7.30 (s, 1H), 7.08 (br s, 3H, exchangeable with D_2_O), 6.36 (br t, 1H, exchangeable with D_2_O), 3.86 (s, 3H), 3.15–3.08 (m, 4H), 1.53–1.45 (m, 4H), 1.36–1.31 (m, 2H); ^13^C NMR (101 MHz, DMSO-*d*_6_) δ 167.13, 157.19, 155.61, 153.02, 140.33, 130.19, 129.20, 128.44, 125.05, 121.74, 120.58, 107.52, 106.95, 52.41, 41.21, 29.88, 28.67, 23.94. HRMS (ESI): *m*/*z* [M + H]^+^ calcd for C_19_H_25_N_5_O_4_ + H^+^: 388.1979. Found: 388.1969.

#### Methyl 4-Hydroxy-6-(3-(3-(3-methylguanidino)propyl)ureido)-2-naphthoate (**7d**)

The TFA salt of compound **7d** was obtained as a yellow solid (39 mg, 97%) starting from compound **26a** (47 mg, 0.082 mmol) following the procedure described for **5a**. ^1^H NMR (400 MHz, DMSO-*d*_6_) δ 10.29 (s, 1H, exchangeable with D_2_O), 8.95 (s, 1H, exchangeable with D_2_O), 8.27 (d, *J* = 2.2 Hz, 1H), 7.97 (s, 1H), 7.89 (d, *J* = 8.9 Hz, 1H), 7.57 (dd, *J* = 8.9, 2.2 Hz, 1H), 7.42–7.30 (m, 4H, 3H exchangeable with D_2_O), 6.39 (br t, 1H, exchangeable with D_2_O), 3.86 (s, 3H), 3.20–3.15 (m, 4H), 2.76 (d, *J* = 4.7 Hz, 3H), 1.72–1.65 (m, 2H). ^13^C NMR (101 MHz, DMSO-*d*_6_) δ 167.12, 156.76, 155.82, 153.03, 140.19, 130.21, 129.28, 128.40, 125.15, 121.73, 120.66, 107.74, 106.96, 52.43, 36.97, 29.89, 28.44. HRMS (ESI): *m*/*z* [M + H]^+^ calcd for C_18_H_23_N_5_O_4_ + H^+^: 374.1823. Found: 374.1816.

#### Methyl 4-Hydroxy-6-(3-(4-(3-methylguanidino)butyl)ureido)-2-naphthoate (**7e**)

The TFA salt of compound **7e** was obtained as a yellow solid (136 mg, 92%) starting from compound **26b** (125 mg, 0.250 mmol) following the procedure described for **5a**. ^1^H NMR (400 MHz, DMSO-*d*_6_) δ 10.30 (s, 1H, exchangeable with D_2_O), 8.94 (s, 1H, exchangeable with D_2_O), 8.28 (d, *J* = 2.1 Hz, 1H), 7.97 (s, 1H), 7.89 (d, *J* = 8.8 Hz, 1H), 7.55 (dd, *J* = 8.9, 2.2 Hz, 1H), 7.44–7.21 (m, 4H, 3H exchangeable with D_2_O), 6.41 (br t, 1H, exchangeable with D_2_O), 3.86 (s, 3H), 3.26–3.08 (m, 4H), 2.74 (d, *J* = 4.5 Hz, 3H), 1.60–1.42 (m, 4H); ^13^C NMR (101 MHz, DMSO-*d*_6_) δ 166.63, 156.23, 155.21, 152.53, 139.81, 129.69, 128.72, 127.94, 124.58, 121.23, 120.11, 107.09, 106.45, 51.91, 40.60, 38.59, 27.91, 27.04, 26.01. HRMS (ESI): *m*/*z* [M + H]^+^ calcd for C_19_H_25_N_5_O_4_ + H^+^: 388.1979. Found: 388.1972.

#### Methyl 6-(3-(3-(1*H*-Imidazol-1-yl)propyl)ureido)-4-hydroxy-2-naphthoate (**7f**)

To a stirring solution of methyl 4-acetoxy-6-((phenoxycarbonyl)amino)-2-naphthoate **22** (400 mg, 1.05 mmol) in dry DMF (4 mL), a solution of 3-(1*H*-imidazol-1-yl)propan-1-amine (263 mg, 2.11 mmol) and TEA (293 μL, 2.10 mmol) in dry DMF (4 mL) was added, and the reaction was stirred at room temperature for 2 h. NaHCO_3_ saturated solution was added (25 mL), and the resulting mixture was extracted with AcOEt (3 × 25 mL). The combined organic layers were washed with NaHCO_3_ saturated solution (2 × 10 mL) and brine (10 mL), dried over Na_2_SO_4_, filtered, and concentrated under reduced pressure to give a light brown solid that was purified by flash chromatography (gradient AcOEt/MeOH 100:0 to 90:10) yielding a mixture of acetylated and deacetylated compounds. Therefore, the solid was suspended in DCM (15 mL), and piperidine (290 μL) was added. After 1 h, the solvent was removed, and the crude product was diluted with AcOEt (50 mL). The organic layer was washed with 1 N HCl (3× 20 mL) and brine (30 mL), dried over Na_2_SO_4_, filtered, and concentrated under reduced pressure, yielding pure **7f** as a white solid (274 mg, 71%). ^1^H NMR (400 MHz, DMSO-*d*_6_) δ 10.32 (s, 1H, exchangeable with D_2_O), 8.87 (s, 1H, exchangeable with D_2_O), 8.27 (d, *J* = 2.2 Hz, 1H), 8.00–7.97 (m, 1H), 7.89 (d, *J* = 8.9 Hz, 1H), 7.67 (s, 1H), 7.56 (dd, *J* = 8.9, 2.2 Hz, 1H), 7.30 (d, *J* = 1.6 Hz, 1H), 7.22 (s, 1H), 6.91 (s, 1H), 6.36 (br t, 1H, exchangeable with D_2_O), 4.02 (t, *J* = 6.9 Hz, 2H), 3.86 (s, 3H), 3.12–3.07 (m, 2H), 1.94–1.87 (m, 2H). ^13^C NMR (101 MHz, DMSO-*d*_6_) δ 167.12, 155.65, 153.03, 140.20, 137.75, 130.19, 129.25, 128.87, 128.40, 125.11, 121.72, 120.65, 119.80, 107.70, 106.95, 52.41, 44.16, 36.92, 31.85. HRMS (ESI): *m*/*z* [M + H]^+^ calcd for C_19_H_20_N_4_O_4_ + H^+^: 369.1557. Found: 369.1550.

#### 6-(3-(4-Guanidinobutyl)ureido)-4-hydroxy-2-naphthoic Acid (**8a**)

The TFA salt of compound **8a** was obtained as a yellow solid (45 mg, 92%) starting from compound **7b** (50 mg, 0.102 mmol) following the procedure described for **6a**. ^1^H NMR (400 MHz, DMSO-*d*_6_) δ 10.23 (s, 1H, exchangeable with D_2_O), 8.94 (s, 1H, exchangeable with D_2_O), 8.27 (d, *J* = 2.2 Hz, 1H), 7.93 (s, 1H), 7.86 (d, *J* = 8.9 Hz, 1H), 7.61–7.52 (m, 2H, 1H exchangeable with D_2_O), 7.29 (s, 1H), 7.25–6.63 (br s, 3H, exchangeable with D_2_O), 6.44 (br t, 1H, exchangeable with D_2_O), 3.23–3.05 (m, 4H), 1.68–1.39 (m, 4H). ^13^C NMR (101 MHz, DMSO-*d*_6_) δ 167.73, 156.66, 155.21, 152.35, 139.50, 129.59, 128.79, 127.73, 125.80, 121.26, 119.95, 107.16, 106.92, 27.06, 26.00. HRMS (ESI): *m*/*z* [M + H]^+^ calcd for C_17_H_21_N_5_O_4_ + H^+^: 360.1666. Found: 360.1658.

#### 6-(3-(3-(1*H*-Imidazol-1-yl)propyl)ureido)-4-hydroxy-2-naphthoic Acid (**8b**)

Compound **8b** was obtained as a yellow solid (62 mg, 89%) starting from compound **7f** (72 mg, 0.195 mmol) following the procedure described for **6a**. ^1^H NMR (400 MHz, DMSO-*d*_6_) δ 12.69 (s, 1H, exchangeable with D_2_O), 10.22 (s, 1H, exchangeable with D_2_O), 9.13 (s, 1H, exchangeable with D_2_O), 9.02 (s, 1H), 8.27 (d, *J* = 2.2 Hz, 1H), 7.94 (s, 1H), 7.89–7.83 (m, 2H), 7.70 (s, 1H), 7.56 (dd, *J* = 8.9, 2.2 Hz, 1H), 7.30 (s, 1H), 6.53 (br t, 1H, exchangeable with D_2_O), 4.26 (t, *J* = 7.0 Hz, 2H), 3.18–3.13 (m, 2H), 2.05–1.98 (m, 2H). ^13^C NMR (101 MHz, DMSO-*d*_6_) δ 168.21, 155.84, 152.87, 139.90, 135.99, 130.06, 129.35, 128.20, 126.36, 122.47, 121.74, 120.65, 120.54, 107.86, 107.42, 46.92, 36.51, 30.94. HRMS (ESI): *m*/*z* [M + H]^+^ calcd for C_18_H_18_N_4_O_4_ + H^+^: 355.1401. Found: 355.1394.

#### Methyl (*S*)-6-(2-((((9*H*-Fluoren-9-yl)methoxy)carbonyl)amino)-4-(methylthio)butanamido)-4-((*tert*-butoxycarbonyl)oxy)-2-naphthoate (**9a**)

Under nitrogen atmosphere **16** (692 mg, 2.18 mmol), (((9*H*-fluoren-9-yl)methoxy)carbonyl)-*l*-methionine (969 mg, 2.61 mmol), and DMAP (27 mg, 0.218 mmol) were dissolved in dry DCM (8 mL), and then DCC (536 mg, 2.60 mmol) was added. The reaction mixture was stirred until the disappearance of the starting material (TLC analysis, 8–12 h). The resulting suspension was filtered, and the filtrate was evaporated under reduced pressure. The crude material was purified by flash chromatography (gradient Hex/AcOEt 80:20 to 20:80) yielding **9a** as a pale yellow solid (1.18 g, 81%). ^1^H NMR (400 MHz, DMSO-*d*_6_) δ 10.57 (s, 1H, exchangeable with D_2_O), 8.51 (s, 1H, exchangeable with D_2_O), 8.47–8.41 (m, 1H), 8.19 (d, *J* = 9.0 Hz, 1H), 7.92–7.88 (m, 2H), 7.86–7.72 (m, 5H), 7.45–7.39 (m, 2H), 7.36–7.31 (m, 2H), 4.35–4.28 (m, 3H), 4.27–4.22 (m, 1H), 3.92 (s, 3H), 3.32–3.28 (m, 2H), 2.08 (s, 3H), 2.05–1.92 (m, 2H), 1.52 (s, 9H). MS (ESI) *m*/*z*: 671 (M + H)^+^.

#### Methyl (*S*)-6-(2-Amino-4-(methylthio)butanamido)-4-hydroxy-2-naphthoate (**9c**)

To a solution of **9a** (1.07 g, 1.60 mmol) in 20 mL of DCM, piperidine (1 mL) was added. After 30 min, the solvent was removed, and the crude product was diluted with AcOEt (40 mL). The organic layer was washed with 1 N HCl (3 × 10 mL) and brine (30 mL), dried over Na_2_SO_4_, filtered, and concentrated under reduced pressure. The crude material **9b** was dissolved in 10 mL of a solution of DCM/TFA (9:1), and the mixture was stirred for 1 h. The solvent was evaporated, and the resulting solid was washed with CHCl_3_ to give the pure TFA salt of compound **9c** as a light brown solid (503 mg, 68%). ^1^H NMR (400 MHz, DMSO-*d*_6_) δ 10.43 (s, 2H, exchangeable with D_2_O), 8.58 (s, 1H), 8.04–8.01 (m, 1H), 7.97 (d, *J* = 8.8 Hz, 1H), 7.77 (dd, *J* = 8.8, 2.2 Hz, 1H), 7.34 (d, *J* = 2.2 Hz, 1H), 3.87 (s, 3H), 3.49–3.46 (m, 1H), 3.00–2.97 (m, 1H), 2.64–2.55 (m, 2H), 2.06 (s, 3H), 2.00–1.93 (m, 1H). ^13^C NMR (101 MHz, DMSO-*d*_6_) δ 174.11, 166.55, 153.00, 137.81, 129.93, 127.46, 125.73, 121.05, 120.83, 109.84, 106.64, 54.72, 52.02, 34.26, 29.89, 14.62. HRMS (ESI): *m*/*z* [M + H]^+^ calcd for C_17_H_20_N_2_O_4_S + H^+^: 349.1217. Found: 349.1211.

#### Methyl (*S*)-4-Hydroxy-6-(2-(3-(4-methoxyphenyl)ureido)-4-(methylthio)butanamido)-2-naphthoate (**9d**)

A solution of 4-methoxyphenyl isocyanate (77 mg, 0.519 mmol) in dry THF (6 mL) was added dropwise to a solution of **9c** (200 mg, 0.432 mmol) and TEA (126 μL, 0.950 mmol) in dry THF (20 mL) under N_2_ atmosphere. The reaction was stirred at room temperature for 6 h. The precipitate was collected by filtration and rinsed with water and diethyl ether successively. Purification by crystallization (EtOH) gave the title compound as a pale yellow solid (169 mg, 79%). ^1^H NMR (400 MHz, DMSO-*d*_6_) δ 10.55–10.45 (m, 2H, exchangeable with D_2_O), 8.55 (d, *J* = 2.2 Hz, 1H), 8.47 (s, 1H, exchangeable with D_2_O), 8.05–8.01 (m, 1H), 7.99 (d, *J* = 8.9 Hz, 1H), 7.76 (dd, *J* = 8.9, 2.2 Hz, 1H), 7.34 (s, 1H), 7.30 (d, *J* = 9.0 Hz, 2H), 6.82 (d, *J* = 9.0 Hz, 2H), 6.50 (br d, 1H exchangeable with D_2_O), 4.53–4.48 (m, 1H), 3.87 (s, 3H), 3.69 (s, 3H), 2.57–2.53 (m, 2H), 2.08 (s, 3H), 2.08–2.02 (m, 1H), 2.02–1.87 (m, 1H). ^13^C NMR (101 MHz, DMSO-*d*_6_) δ 171.26, 166.53, 155.03, 154.06, 153.01, 137.77, 133.26, 130.01, 129.81, 127.41, 125.85, 121.05, 120.82, 119.33, 113.93, 110.16, 106.68, 55.12, 53.15, 52.03, 32.84, 29.52, 14.74. HRMS (ESI): *m*/*z* [M + H]^+^ calcd for C_25_H_27_N_3_O_6_S + H^+^: 498.1693. Found: 498.1687.

#### Methyl 6-(2-((*tert*-Butoxycarbonyl)amino)acetamido)-4-((*tert*-butoxycarbonyl)oxy)-2-naphthoate (**10a**)

Compound **10a** was obtained as a pale yellow solid (1.58 g, 79%) by the reaction of amine **16** (1.34 g, 4.22 mmol) and (*tert*-butoxycarbonyl)glycine (888 mg, 5.07 mmol) following the procedure described for **9a**. ^1^H NMR (400 MHz, CDCl_3_) δ 8.56 (br s, 1H, exchangeable with D_2_O), 8.47 (s, 1H), 8.27 (s, 1H), 7.94 (d, *J* = 8.9 Hz, 1H), 7.85 (d, *J* = 1.5 Hz, 1H), 7.14–7.09 (m, 1H), 5.27 (br s, 1H, exchangeable with D_2_O), 4.00 (s, 3H), 3.69 (d, *J* = 6.2 Hz, 2H), 1.53–1.48 (m, 18H). MS (ESI) *m*/*z*: 475 (M + H)^+^.

#### Methyl 6-(2-Aminoacetamido)-4-hydroxy-2-naphthoate (**10b**)

Compound **10b** was obtained as a light brown solid (434 mg, 74%) by the reaction of **10a** (716 mg, 1.51 mmol) following the procedure described for **5a**. ^1^H NMR (400 MHz, DMSO-*d*_6_) δ 10.42 (s, 1H, exchangeable with D_2_O), 10.28 (s, 1H, exchangeable with D_2_O), 8.54 (s, 1H), 8.03–7.87 (m, 2H), 7.81–7.61 (m, 4H, 3H exchangeable with D_2_O), 7.32 (s, 1H), 3.92–3.86 (m, 2H), 3.86 (s, 3H). MS (ESI) *m*/*z*: 275 (M + H)^+^.

#### Methyl 4-Hydroxy-6-(2-(3-(4-methoxyphenyl)ureido)acetamido)-2-naphthoate (**10c**)

Compound **10c** was obtained as a white solid (250 mg, 81%) by the reaction of amine **10b** (283 mg, 0.731 mmol) and 4-methoxyphenyl isocyanate (129 mg, 0.871 mmol) following the procedure described for **9d**. ^1^H NMR (400 MHz, DMSO-*d*_6_) δ 10.56 (s, 1H, exchangeable with D_2_O), 10.33 (s, 1H, exchangeable with D_2_O), 8.63 (s, 1H, exchangeable with D_2_O), 8.51 (s, 1H), 8.04–7.95 (m, 2H), 7.70 (dd, *J* = 8.9, 2.2 Hz, 1H), 7.32–7.28 (m, 3H), 6.82 (d, *J* = 9.0 Hz, 2H), 6.36 (br t, 1H, exchangeable with D_2_O), 3.97 (d, *J* = 5.6 Hz, 2H), 3.86 (s, 3H), 3.68 (s, 3H). ^13^C NMR (101 MHz, DMSO-*d*_6_) δ 169.33, 166.84, 155.84, 154.32, 153.16, 138.05, 133.50, 130.12, 130.0986, 129.79, 127.51, 125.71, 121.02, 120.64, 119.32, 113.88, 109.75, 106.67, 55.12, 52.01, 21.66. HRMS (ESI): *m*/*z* [M + H]^+^ calcd for C_22_H_21_N_3_O_6_ + H^+^: 424.1503. Found: 424.1500.

#### Methyl (*S*)-6-(2-((((9*H*-Fluoren-9-yl)methoxy)carbonyl)amino)-5-(3-((2,2,4,6,7-pentamethyl-2,3-dihydrobenzofuran-5-yl)sulfonyl)guanidino)pentanamido)-4-((*tert*-butoxycarbonyl)oxy)-2-naphthoate (**11a**)

Compound **11a** was obtained as a pale yellow solid (1.50 g, 79%) by the reaction of *N*^α^-(((9*H*-fluoren-9-yl)methoxy)carbonyl)-*N*^ω^-((2,2,4,6,7-pentamethyl-2,3-dihydrobenzofuran-5-yl)sulfonyl)-*l*-arginine (1.55 g, 2.40 mmol) with amine **16** (634 mg, 2.00 mmol) following the procedure described for **9a**. ^1^H NMR (400 MHz, DMSO-*d*_6_) δ 10.55 (s, 1H, exchangeable with D_2_O), 8.51 (d, *J* = 1.8 Hz, 1H), 8.50–8.43 (m, 1H), 8.22–8.16 (m, 1H), 7.93–7.87 (m, 2H), 7.87–7.84 (m, 2H), 7.82–7.72 (m, 3H), 7.45–7.39 (m, 1H), 7.39–7.30 (m, 2H), 6.72 (br s, 2H, exchangeable with D_2_O), 6.38 (br s, 2H, exchangeable with D_2_O), 6.28 (s, 1H), 4.37–4.27 (m, 1H), 4.27–4.19 (m, 1H), 3.92 (s, 3H), 3.15–3.05 (m, 2H), 2.95–2.84 (m, 2H), 2.48–2.45 (m, 3H), 2.42–2.38 (m, 4H), 1.98–1.92 (m, 4H), 1.55 (s, 9H), 1.52 (s, 3H), 1.37 (s, 3H), 1.36 (s, 3H). MS (ESI) *m*/*z*: 948 (M + H)^+^.

#### Methyl (*S*)-6-(2-Amino-5-(3-((2,2,4,6,7-pentamethyl-2,3-dihydrobenzofuran-5-yl)sulfonyl)guanidino)pentanamido)-4-((*tert*-butoxycarbonyl)oxy)-2-naphthoate (**11b**)

To a solution of **11a** (948 mg, 1.00 mmol) in 20 mL of DCM, piperidine (1 mL) was added. After 30 min, the solvent was removed, and the crude product was diluted with AcOEt (40 mL). The organic layer was washed with 1 N HCl (3 × 10 mL) and brine (30 mL), dried over Na_2_SO_4_, filtered, and concentrated under reduced pressure yielding pure **11b** as a light yellow solid (522 mg, 72%). ^1^H NMR (400 MHz, DMSO-*d*_6_) δ 10.31 (s, 1H, exchangeable with D_2_O), 8.57 (d, *J* = 2.2 Hz, 1H), 8.01 (s, 1H), 7.97 (d, *J* = 8.9 Hz, 1H), 7.76 (dd, *J* = 8.8, 2.2 Hz, 1H), 7.34 (d, *J* = 1.8 Hz, 1H), 6.75 (br s, 2H, exchangeable with D_2_O), 6.39 (br s, 2H, exchangeable with D_2_O), 3.87 (s, 3H), 3.10–3.03 (m, 3H), 2.90–2.85 (m, 4H), 2.48 (s, 6H), 2.46 (s, 3H), 2.39 (s, 3H), 1.96 (s, 3H), 1.37 (s, 9H). MS (ESI) *m*/*z*: 726 (M + H)^+^.

#### Methyl (*S*)-6-(2-Amino-5-guanidinopentanamido)-4-hydroxy-2-naphthoate (**11c**)

Compound **11b** (100 mg, 0.137 mmol) was dissolved in a solution of DCM/TFA 1:9, and then a drop of water was added. The resulting mixture was stirred at room temperature for 24 h. The solvent was evaporated, and the resulting solid was washed with CHCl_3_ to afford the TFA salt as a white solid (45 mg, 68%). ^1^H NMR (400 MHz, DMSO-*d*_6_) δ 10.95 (s, 1H, exchangeable with D_2_O), 8.55 (d, *J* = 2.0 Hz, 1H), 8.45–8.35 (m, 3H, exchangeable with D_2_O), 8.04–7.99 (m, 1H), 7.92–7.89 (m, 1H), 7.74 (dd, *J* = 8.7, 2.4 Hz, 1H), 7.46–7.22 (m, 4H, 1H, exchangeable with D_2_O), 3.87 (s, 3H), 3.19–3.14 (m, 2H), 1.91–1.86 (m, 2H), 1.63–1.57 (m, 2H). ^13^C NMR (101 MHz, DMSO-*d*_6_) δ 168.05, 166.99, 157.38, 153.63, 137.38, 130.83, 130.55, 127.80, 126.79, 121.45, 121.09, 111.11, 107.36, 53.32, 52.53, 40.61, 28.87, 24.74. HRMS (ESI): *m*/*z* [M + H]^+^ calcd for C_18_H_23_N_5_O_4_ + H^+^: 374.1823. Found: 374.1821.

#### Methyl (*S*)-4-((*tert*-Butoxycarbonyl)oxy)-6-(2-(3-(4-methoxyphenyl)ureido)-5-(3-((2,2,4,6,7-pentamethyl-2,3-dihydrobenzofuran-5-yl)sulfonyl)guanidino)pentanamido)-2-naphthoate (**11d**)

Compound **11d** was obtained as a pale yellow solid (239 mg, 82%) by the reaction of 4-methoxyphenyl isocyanate (61 mg, 0.413 mmol) with amine **11b** (250 mg, 0.334 mmol) following the procedure described for **9d**. ^1^H NMR (400 MHz, DMSO-*d*_6_) δ 10.58–10.41 (m, 2H, exchangeable with D_2_O), 8.55 (d, *J* = 2.1 Hz, 1H), 8.50 (s, 1H, exchangeable with D_2_O), 8.04–8.01 (m, 1H), 7.98 (d, *J* = 8.9 Hz, 1H), 7.75 (dd, *J* = 8.9, 2.2 Hz, 1H), 7.34 (s, 1H), 7.29 (d, *J* = 9.0 Hz, 2H), 6.82 (d, *J* = 9.0 Hz, 2H), 6.74 (brs, 2H, exchangeable with D_2_O), 6.43 (br d, 1H, exchangeable with D_2_O), 6.35 (s, 2H, exchangeable with D_2_O), 4.45–4.41 (m, 1H), 3.87 (s, 3H), 3.69 (s, 3H), 3.15–3.06 (m, 2H), 2.82 (s, 3H), 2.44 (s, 3H), 2.37 (s, 3H), 1.93 (s, 3H), 1.82–1.68 (m, 2H), 1.69–1.58 (m, 2H), 1.49–1.38 (m, 2H), 1.35 (s, 9H). MS (ESI) *m*/*z*: 875 (M + H)^+^.

#### Methyl (*S*)-6-(5-Guanidino-2-(3-(4-methoxyphenyl)ureido)pentanamido)-4-hydroxy-2-naphthoate (**11e**)

Compound **11e** was obtained as a pale yellow solid (74 mg, 77%) starting from **11d** (131 mg, 0.150 mmol) following the procedure described for **11c**. ^1^H NMR (400 MHz, DMSO-*d*_6_) δ 10.50–10.47 (m, 2H, exchangeable with D_2_O), 8.56 (d, *J* = 3.5 Hz, 1H), 8.02 (s, 1H), 8.00 (d, *J* = 8.9 Hz, 1H), 7.76 (dd, *J* = 8.9, 2.2 Hz, 1H), 7.53–7.48 (m, 1H), 7.35–7.34 (m, 1H), 7.30 (d, *J* = 9.1 Hz 2H), 7.05 (br s, 3H, exchangeable with D_2_O), 6.84 (d, *J* = 9.1 Hz, 2H), 6.52–6.49 (m, 1H, exchangeable with D_2_O), 4.52–4.45 (m, 1H), 3.88 (s, 3H), 3.69 (s, 3H), 3.19–3.11 (m, 2H), 1.85–1.65 (m, 2H), 1.59–1.45 (m, 2H). ^13^C NMR (101 MHz, DMSO-*d*_6_) δ 171.44, 166.51, 156.61, 154.98, 154.07, 153.01, 137.67, 133.26, 130.04, 129.88, 127.40, 125.91, 121.06, 120.72, 119.30, 113.95, 110.12, 106.72, 55.13, 53.12, 52.05, 40.44, 30.51, 25.07. HRMS (ESI): *m*/*z* [M + H]^+^ calcd for C_26_H_30_N_6_O_6_ + H^+^: 523.2300. Found: 523.2293.

#### Methyl 6-(3-(3-(3-(((2*R*,3*S*,4*R*,5*R*)-5-(6-Amino-9*H*-purin-9-yl)-3,4-dihydroxytetrahydrofuran-2-yl)methyl)guanidino)propyl)ureido)-4-hydroxy-2-naphthoate (**12a**)

Compound **29a** (100 mg, 0.118 mmol) was dissolved in a solution of DCM/TFA 1:1, and then a drop of water was added. The resulting mixture was stirred at room temperature for 1 h. The solvent was evaporated, and the resulting solid was washed with CHCl_3_ to afford the TFA salt **12a** as a white solid (64 mg, 76%). ^1^H NMR (400 MHz, DMSO-*d*_6_) δ 10.30 (s, 1H, exchangeable with D_2_O), 8.95 (s, 1H, exchangeable with D_2_O), 8.45 (s, 1H), 8.27–8.21 (m, 2H), 7.98 (s, 1H), 7.88 (d, *J* = 8.9 Hz, 1H), 7.86 (br s, 2H, exchangeable with D_2_O), 7.56 (dd, *J* = 9.0, 2.2 Hz, 1H), 7.49–7.42 (m, 4H, exchangeable with D_2_O), 7.30 (s, 1H), 6.39 (br t, 1H, exchangeable with D_2_O), 5.95 (d, *J* = 5.7 Hz, 1H), 4.72 (t, *J* = 5.4 Hz, 1H), 4.18–4.16 (m, 1H), 4.06–4.01 (m, 1H), 3.87 (s, 3H), 3.18–3.12 (m, 4H), 1.70–1.60 (m, 2H). ^13^C NMR (101 MHz, DMSO-*d*_6_) δ 166.63, 155.88, 155.31, 152.53, 149.03, 140.76, 139.69, 129.72, 128.79, 127.90, 124.66, 121.25, 120.17, 119.22, 107.25, 106.47, 87.82, 82.34, 72.71, 71.07, 51.94, 43.17, 36.47, 29.32. HRMS (ESI): *m*/*z* [M + H]^+^ calcd for C_27_H_32_N_10_O_7_ + H^+^: 609.2528. Found: 609.2520.

#### Methyl 6-(3-(4-(3-(((2*R*,3*S*,4*R*,5*R*)-5-(6-Amino-9*H*-purin-9-yl)-3,4-dihydroxytetrahydrofuran-2-yl)methyl)guanidino)butyl)ureido)-4-hydroxy-2-naphthoate (**12b**)

Compound **12b** was obtained as a white solid (33 mg, 75%) starting from compound **29b** (52 mg, 0.060 mmol) following the procedure described for **12a**. ^1^H NMR (400 MHz, DMSO-*d*_6_) δ 10.28 (s, 1H, exchangeable with D_2_O), 8.90 (s, 1H, exchangeable with D_2_O), 8.42 (s, 1H), 8.28–8.21(m, 2H), 7.97 (s, 1H), 7.88 (d, *J* = 8.9 Hz, 1H), 7.78 (br s, 2H, exchangeable with D_2_O), 7.54 (dd, *J* = 8.9, 2.2 Hz, 1H), 7.42–7.33 (m, 4H, exchangeable with D_2_O), 7.30 (s, 1H), 6.34 (br t, 1H, exchangeable with D_2_O), 5.93 (d, *J* = 5.7 Hz, 1H), 4.71 (t, *J* = 5.7 Hz, 1H), 4.17–4.15 (m, 1H), 4.06–3.98 (m, 1H), 3.86 (s, 3H), 3.60–3.48 (m, 2H), 3.17–3.05 (m, 4H), 1.51–1.45 (m, 4H). ^13^C NMR (101 MHz, DMSO-*d*_6_) δ 166.62, 155.80, 155.14, 152.51, 151.14, 149.05, 140.59, 139.77, 129.70, 128.72, 127.91, 121.23, 120.09, 119.22, 107.08, 106.45, 87.79, 82.34, 72.64, 71.05, 51.92, 43.12, 26.99, 25.88. HRMS (ESI): *m*/*z* [M + H]^+^ calcd for C_28_H_34_N_10_O_7_ + H^+^: 623.2685. Found: 623.2689.

#### Methyl 6-(3-(5-(3-(((2*R*,3*S*,4*R*,5*R*)-5-(6-Amino-9*H*-purin-9-yl)-3,4-dihydroxytetrahydrofuran-2-yl)methyl)guanidino)pentyl)ureido)-4-hydroxy-2-naphthoate (**12c**)

Compound **12c** was obtained as a pale yellow solid (63 mg, 78%) starting from compound **29c** (95 mg, 0.108 mmol) following the procedure described for **12a**. ^1^H NMR (400 MHz, DMSO-*d*_6_) δ 10.28 (s, 1H, exchangeable with D_2_O), 8.83 (s, 1H, exchangeable with D_2_O), 8.38 (s, 1H), 8.25 (d, *J* = 2.2 Hz, 1H), 8.20 (s,1H), 7.96 (s, 1H), 7.88 (d, *J* = 8.9 Hz, 1H), 7.59 (s, 2H, exchangeable with D_2_O), 7.53 (dd, *J* = 8.9, 2.2 Hz, 1H), 7.37–7.32 (m, 4H, exchangeable with D_2_O), 7.29 (s, 1H), 6.25 (br t, 1H, exchangeable with D_2_O), 5.92 (d, *J* = 5.7 Hz, 1H), 4.71 (t, *J* = 5.4 Hz, 1H), 4.16–4.14 (m, 1H), 4.03–3.99 (m, 1H), 3.85 (s, 3H), 3.10–3.05 (m, 4H), 1.47–1.40 (m, 4H), 1.28–1.24 (m, 2H). ^13^C NMR (101 MHz, DMSO-*d*_6_) δ 167.13, 156.32, 155.59, 153.01, 149.59, 141.00, 140.29, 130.21, 129.21, 128.42, 125.08, 121.75, 120.59, 119.77, 107.54, 106.95, 88.33, 82.87, 73.10, 71.54, 52.42, 43.62, 41.49, 29.88, 28.58, 23.91. HRMS (ESI): *m*/*z* [M + H]^+^ calcd for C_29_H_36_N_10_O_7_ + H^+^: 637.2841. Found: 637.2843.

#### Methyl 6-(3-(6-(3-(((2*R*,3*S*,4*R*,5*R*)-5-(6-Amino-9*H*-purin-9-yl)-3,4-dihydroxytetrahydrofuran-2-yl)methyl)guanidino)hexyl)ureido)-4-hydroxy-2-naphthoate (**12d**)

Compound **12d** was obtained as a pale yellow solid (0.065 g, 74%) starting from compound **29d** (92 mg, 0.103 mmol) following the procedure described for **12a**. ^1^H NMR (400 MHz, DMSO-*d*_6_) δ 10.27 (s, 1H, exchangeable with D_2_O), 8.86 (s, 1H, exchangeable with D_2_O), 8.41 (s, 1H), 8.25 (d, *J* = 2.2 Hz, 1H), 8.24 (s,1H), 7.46 (s, 1H), 7.87 (d, *J* = 8.9 Hz, 1H), 7.80 (s, 2H, exchangeable with D_2_O), 7.53 (dd, *J* = 8.9, 2.2 Hz, 1H), 7.42–7.35 (m, 4H), 7.29 (s, 1H), 6.30 (br t, 1H, exchangeable with D_2_O), 5.92 (d, *J* = 5.7 Hz, 1H), 4.69 (t, *J* = 5.4 Hz, 1H), 4.18–4.14 (m, 1H), 4.03–3.99 (m, 1H), 3.85 (s, 3H), 3.58–3.45 (m, 2H), 3.12–3.03 (m, 4H), 1.48–1.34 (m, 4H), 1.26–1.18 (m, 4H). ^13^C NMR (101 MHz, DMSO-*d*_6_) δ 166.63, 155.83, 155.08, 152.51, 151.04, 149.03, 140.63, 139.383, 129.69, 128.70, 127.94, 124.55, 121.24, 120.08, 119.23, 107.01, 106.45, 87.81, 82.42, 72.66, 71.02, 51.92, 43.10, 40.98, 29.65, 28.32, 25.95, 25.76. HRMS (ESI): *m*/*z* [M + H]^+^ calcd for C_30_H_38_N_10_O_7_ + H^+^: 651.2998. Found: 651.2997.

#### Methyl 6-(3-(7-(3-(((2*R*,3*S*,4*R*,5*R*)-5-(6-Amino-9*H*-purin-9-yl)-3,4-dihydroxytetrahydrofuran-2-yl)methyl)guanidino)heptyl)ureido)-4-hydroxy-2-naphthoate (**12e**)

Compound **12e** was obtained as a pale yellow solid (58 mg, 75%) starting from compound **29e** (0.100 g, 0.110 mmol) following the procedure described for **12a**. ^1^H NMR (400 MHz, DMSO-*d*_6_) δ 10.28 (s, 1H, exchangeable with D_2_O), 8.85 (s, 1H, exchangeable with D_2_O), 8.42 (s, 1H), 8.27 (d, *J* = 2.1 Hz, 1H), 8.24 (s,1H), 7.97 (s, 1H), 7.88 (d, *J* = 8.9 Hz, 1H), 7.80 (s, 2H, exchangeable with D_2_O), 7.54 (dd, *J* = 8.9, 2.1 Hz, 1H), 7.42–7.26 (m, 5H, 4H exchangeable with D_2_O), 6.29 (br t, 1H, exchangeable with D_2_O), 5.93 (d, *J* = 5.7 Hz, 1H), 4.70 (t, *J* = 5.4 Hz, 1H), 4.17–4.14 (m, 1H), 4.03–4.01 (m, 1H), 3.86 (s, 3H), 3.56–3.50 (m, 2H), 3.13–3.03 (m, 4H), 1.44–1.40 (m, 4H), 1.29–1.24 (m, 4H). ^13^C NMR (101 MHz, DMSO-*d*_6_) δ 167.13, 156.32, 155.57, 153.01, 151.60, 149.54, 141.11, 140.32, 130.20, 129.20, 128.44, 125.05, 121.74, 120.57, 119.74, 107.50, 106.95, 88.31, 82.92, 73.16, 71.52, 52.41, 43.60, 41.47, 30.18, 28.81, 26.78, 26.49. HRMS (ESI): *m*/*z* [M + H]^+^ calcd for C_31_H_40_N_10_O_7_ + H^+^: 665.3154. Found: 665.3148.

#### Methyl 6-(3-(4-(3-(2-((2*R*,3*S*,4*R*,5*R*)-5-(6-Amino-9*H*-purin-9-yl)-3,4-dihydroxytetrahydrofuran-2-yl)ethyl)guanidino)butyl)ureido)-4-hydroxy-2-naphthoate (**12f**)

Compound **12f** was obtained as a pale yellow solid (85 mg, 80%) starting from compound **30** (110 mg, 0.141 mmol) following the procedure described for **12a**. ^1^H NMR (400 MHz, DMSO-*d*_6_) δ 10.28 (s, 1H, exchangeable with D_2_O), 8.90 (s, 1H, exchangeable with D_2_O), 8.41 (s, 1H), 8.27 (d, *J* = 2.2 Hz, 1H), 8.22 (s, 1H), 7.97 (s, 1H), 7.88 (d, *J* = 8.9 Hz, 1H), 7.66 (br s, 2H, exchangeable with D_2_O), 7.55 (dd, *J* = 8.9, 2.2 Hz, 1H), 7.34–7.24 (m, 5H, 4H, exchangeable with D_2_O), 6.36 (br t, 1H, exchangeable with D_2_O), 5.90 (d, *J* = 5.0 Hz, 1H), 4.69 (t, *J* = 5.4 Hz, 1H), 4.13–4.11 (m, 1H), 3.94–3.89 (m, 1H), 3.86 (s, 3H), 3.23–3.18 (m, 2H), 3.16–3.09 (m, 4H), 1.95–1.86 (m, 2H), 1.50–1.46 (m, 4H). ^13^C NMR (101 MHz, DMSO-*d*_6_) δ 167.12, 156.00, 155.66, 153.01, 149.65, 140.96, 140.28, 130.20, 129.22, 128.42, 125.09, 121.74, 120.60, 119.68, 107.59, 106.95, 88.33, 81.64, 73.77, 73.40, 52.42, 46.24, 41.15, 38.54, 32.85, 27.52, 26.47. HRMS (ESI): *m*/*z* [M + H]^+^ calcd for C_29_H_36_N_10_O_7_ + H^+^: 637.2841. Found: 637.2838.

#### Methyl 6-(3-(4-(3-(3-((2*R*,3*S*,4*R*,5*R*)-5-(6-Amino-9*H*-purin-9-yl)-3,4-dihydroxytetrahydrofuran-2-yl)propyl)guanidino)butyl)ureido)-4-hydroxy-2-naphthoate (**12g**)

Compound **12g** was obtained as a white solid (71 mg, 78%) starting from compound **31** (95 mg, 0.120 mmol) following the procedure described for **12a**. ^1^H NMR (400 MHz, DMSO-*d*_6_) δ 10.29 (s, 1H, exchangeable with D_2_O), 8.90 (s, 1H, exchangeable with D_2_O), 8.38 (s, 1H), 8.27 (d, *J* = 2.1 Hz, 1H), 8.23 (s, 1H), 7.97 (s, 1H), 7.88 (d, *J* = 8.9 Hz, 1H), 7.70 (s, 2H, exchangeable with D_2_O), 7.55 (dd, *J* = 8.9, 2.1 Hz, 1H), 7.34–7.28 (m, 5H, 4H exchangeable with D_2_O), 6.36 (br t, 1H, exchangeable with D_2_O), 5.87 (d, *J* = 5.0 Hz, 1H), 4.64 (t, *J* = 5.4 Hz, 1H), 4.08–4.06 (m, 1H), 3.86 (s, 3H), 3.16–3.12 (m, 6H), 1.70–1.48 (m, 8H). ^13^C NMR (101 MHz, DMSO-*d*_6_) δ 167.13, 155.95, 155.67, 153.01, 149.63, 140.93, 140.27, 130.21, 129.23, 128.42, 125.09, 121.74, 120.60, 107.60, 106.96, 88.32, 83.63, 73.72, 73.53, 52.42, 41.21, 41.13, 39.09, 30.52, 27.54, 26.49, 25.55. HRMS (ESI): *m*/*z* [M + H]^+^ calcd for C_30_H_38_N_10_O_7_ + H^+^: 651.2998. Found: 651.2996.

#### Methyl 6-(3-(4-(3-((*E*)-3-((2*R*,3*S*,4*R*,5*R*)-5-(6-Amino-9*H*-purin-9-yl)-3,4-dihydroxytetrahydrofuran-2-yl)allyl)guanidino)butyl)ureido)-4-hydroxy-2-naphthoate (**12h**)

Compound **12h** was obtained as a white solid (63 mg, 78%) starting from compound **32** (84 mg, 0.106 mmol) following the procedure described for **12a**. ^1^H NMR (400 MHz, DMSO-*d*_6_) δ 10.28 (s, 1H, exchangeable with D_2_O), 8.92 (s, 1H, exchangeable with D_2_O), 8.39 (s, 1H), 8.27 (d, *J* = 2.1 Hz, 1H), 8.24 (s, 1H), 7.97 (s, 1H), 7.88 (d, *J* = 9.0 Hz, 1H), 7.72 (s, 2H, exchangeable with D_2_O), 7.57–7.49 (m, 2H, 1H exchangeable with D_2_O), 7.43 (br t, 1H, exchangeable with D_2_O), 7.35 (s, 2H, exchangeable with D_2_O), 7.30 (s, 1H), 6.37 (br t, 1H, exchangeable with D_2_O), 5.93 (d, *J* = 5.0 Hz, 1H), 5.89–5.86 (m, 1H), 4.69 (t, *J* = 5.4 Hz, 1H), 4.38–4.36 (m, 1H), 4.15–4.12 (m, 1H), 3.84 (s, 3H), 3.84–3.81 (m, 2H), 3.17–3.13 (m, 4H), 1.55–1.42 (m, 4H). ^13^C NMR (101 MHz, DMSO-*d*_6_) δ 167.13, 155.94, 155.68, 153.01, 149.63, 140.97, 140.27, 130.48, 130.21, 129.23, 128.38, 125.09, 121.74, 120.60, 88.38, 84.21, 74.44, 73.28, 54.07, 52.42, 44.66, 42.31, 41.21, 27.52, 26.47. HRMS (ESI): *m*/*z* [M + H]^+^ calcd for C_30_H_36_N_10_O_7_ + H^+^: 649.2841. Found: 649.2829.

#### Methyl 4-Hydroxy-6-nitro-2-naphthoate (**14**)

Compound **14** was obtained as a pale yellow solid (1.53 g, 99%) starting from compound **13** (1.80 g, 6.22 mmol) following the procedure described for **11b**. ^1^H NMR (400 MHz, DMSO-*d*_6_) δ 8.99 (s, 1H, exchangeable with D_2_O), 8.38–8.06 (m, 4H), 7.50 (s, 1H), 3.91 (s, 3H). MS (ESI) *m*/*z*: 248 (M + H)^+^.

#### Methyl 4-((*tert*-Butoxycarbonyl)oxy)-6-nitro-2-naphthoate (**15**)

To a stirred suspension of **14** (1.53 g, 6.22 mmol) in dry DCM (8 mL), DMAP (75 mg, 0.613 mmol), TEA (0.95 mL, 6.80 mmol), and Boc_2_O (1.75 g, 8.04 mmol) were added. The resulting mixture was stirred at room temperature for 12 h. Then, the solvent was removed at reduced pressure, and the crude residue was purified by flash chromatography (gradient Hex/AcOEt 80:20 to 40:60) yielding **15** as a white solid (1.51 g, 70%). ^1^H NMR (400 MHz, CDCl_3_) δ 8.98 (d, *J* = 2.0 Hz, 1H), 8.57 (s, 1H), 8.34 (dd, *J* = 9.0, 2.0 Hz, 1H), 8.11 (m, 2H), 4.01 (s, 3H), 1.64 (s, 9H). MS (ESI) *m*/*z*: 348 (M + H)^+^.

#### Methyl 6-Amino-4-((*tert*-butoxycarbonyl)oxy)-2-naphthoate (**16**)

To a solution of **15** (1.51 g, 4.35 mmol) in acetic acid (50 mL), Zn dust (2.83 g, 43.50 mmol) was added. The resulting mixture was stirred for 1 h at room temperature, filtered, and concentrated in vacuo. The acid residue was dissolved in saturated aqueous solution of NaHCO_3_ (50 mL) and extracted with AcOEt (3 × 50 mL). The organic layer was washed with brine (50 mL), dried (Na_2_SO_4_), and filtered. Vacuum evaporation of the solvent gave the title compound **16** (1.35 g, 99%) as a pale yellow solid which was directly used in the next step without further purification. ^1^H NMR (400 MHz, CDCl_3_) δ 8.29 (s, 1H), 7.78 (s, 1H), 7.73 (d, *J* = 8.7 Hz, 1H), 6.98 (s, 1H), 6.95 (d, *J* = 8.7 Hz, 1H), 4.15 (br s, 2H, exchangeable with D_2_O), 3.92 (s, 3H), 1.58 (s, 9H). MS (ESI) *m*/*z*: 318 (M + H)^+^.

#### Methyl 6-(5-((*tert*-Butoxycarbonyl)amino)pentanamido)-4-((*tert*-butoxycarbonyl)oxy)-2-naphthoate (**17a**)

Compound **17a** was obtained as a pale yellow solid (1.80 g, 83%) starting from compound **16** (1.34 g, 4.22 mmol) and 5-*tert*-butoxycarbonylaminovaleric acid (1.10 g, 5.07 mmol) following the procedure described for **9a**. ^1^H NMR (400 MHz, DMSO-*d*_6_) δ 10.34 (s, 1H, exchangeable with D_2_O), 8.48 (s, 1H), 8.43 (s, 1H), 8.16 (d, *J* = 9.0 Hz, 1H), 7.83–7.70 (m, 2H), 6.80 (br t, 1H, exchangeable with D_2_O), 3.90 (s, 3H), 3.02–2.89 (m, 2H), 2.38 (t, *J* = 7.2 Hz, 2H), 1.63–1.50 (m, 11H), 1.49–1.28 (m, 11H). MS (ESI) *m*/*z*: 517 (M + H)^+^.

#### Methyl 6-(6-((*tert*-Butoxycarbonyl)amino)hexanamido)-4-((*tert*-butoxycarbonyl)oxy)-2-naphthoate (**17b**)

Compound **17b** was obtained as a pale yellow solid (950 mg, 80%) by the reaction of 6-*tert*-butoxycarbonylaminohexanoic acid (633 mg, 2.74 mmol) with amine **16** (725 mg, 2.28 mmol) following the procedure described for **9a**. ^1^H NMR (400 MHz, DMSO-*d*_6_) δ 10.36 (s, 1H, exchangeable with D_2_O), 8.49 (s, 1H), 8.42 (s, 1H), 8.16 (d, *J* = 8.9 Hz, 1H), 7.78–7.73 (m, 2H), 6.81 (br t, 1H, exchangeable with D_2_O), 3.91 (s, 3H), 3.01–2.88 (m, 2H), 2.38 (m, 2H), 1.75–1.50 (m, 11H), 1.44–1.20 (m, 13H). MS (ESI) *m*/*z*: 531 (M+H)^+^.

#### Methyl 6-(7-((*tert*-Butoxycarbonyl)amino)heptanamido)-4-((*tert*-butoxycarbonyl)oxy)-2-naphthoate (**17c**)

Compound **17c** was obtained as a pale yellow solid (1.52 g, 88%) by the reaction of 7-*tert*-butoxycarbonylaminoheptanoic acid (928 mg, 3.78 mmol) with amine **16** (1.00 g, 3.15 mmol) following the procedure described for **9a**. ^1^H NMR (400 MHz, DMSO-*d*_6_) δ 10.35 (s, 1H, exchangeable with D_2_O), 8.49 (s, 1H), 8.42 (s, 1H), 8.16 (d, *J* = 8.9 Hz, 1H), 7.83–7.70 (m, 2H), 6.81 (br t, 1H, exchangeable with D_2_O), 3.91 (s, 3H), 3.01–2.88 (m, 2H), 2.39 (t, *J* = 7.4 Hz, 2H), 1.75–1.50 (m, 11H), 1.44–1.20 (m, 15H). MS (ESI) *m*/*z*: 545 (M+H)^+^.

#### Methyl 6-(5-Aminopentanamido)-4-hydroxy-2-naphthoate (**18a**)

The TFA salt of compound **18a** was obtained as a pale brown solid (870 mg, 71%) starting from compound **17a** (1.47 g, 2.85 mmol) following the procedure described for **5a**. ^1^H NMR (400 MHz, DMSO-*d*_6_) δ 10.44 (s, 1H, exchangeable with D_2_O), 10.26 (s, 1H, exchangeable with D_2_O), 8.54 (s, 1H), 8.05–7.89 (m, 2H), 7.79–7.64 (m, 4H, 3H exchangeable with D_2_O), 7.33 (s, 1H), 3.86 (s, 3H), 2.90–2.76 (m, 2H), 2.41 (t, *J* = 7.0 Hz, 2H), 1.80–1.56 (m, 4H). MS (ESI) *m*/*z*: 317 (M + H)^+^.

#### Methyl 6-(6-Aminohexanamido)-4-hydroxy-2-naphthoate (**18b**)

The TFA salt of compound **18b** was obtained as a brown solid (507 mg, 70%) starting from compound **17b** (868 mg, 1.63 mmol) following the procedure described for **5a**. ^1^H NMR (400 MHz, DMSO-*d*_6_) δ 10.44 (s, 1H, exchangeable with D_2_O), 10.26 (s, 1H, exchangeable with D_2_O), 8.56 (s, 1H), 8.05–7.95 (m, 2H), 7.73–7.66 (m, 4H, 3H exchangeable with D_2_O), 7.34 (s, 1H), 3.86 (s, 3H), 2.80–2.82 (m, 2H), 2.41 (t, *J* = 7.2 Hz, 2H), 1.70–1.56 (m, 4H), 1.40–1.37 (m, 2H). MS (ESI) *m*/*z*: 331 (M + H)^+^.

#### Methyl 6-(7-Aminoheptanamido)-4-hydroxy-2-naphthoate (**18c**)

The TFA salt of compound **18c** was obtained as a yellow solid (503 mg, 60%) starting from compound **17c** (1.00 g, 1.83 mmol) following the procedure described for **5a**. ^1^H NMR (400 MHz, DMSO-*d*_6_) δ 10.46 (s, 1H, exchangeable with D_2_O), 10.26 (s, 1H, exchangeable with D_2_O), 8.55 (s, 1H), 8.05–7.96 (m, 2H), 7.73–7.66 (m, 4H, 3H exchangeable with D_2_O), 7.34 (s, 1H), 3.86 (s, 3H), 2.79–2.75 (m, 2H), 2.41 (t, *J* = 7.0 Hz, 2H), 1.65–1.52 (m, 4H), 1.40–1.27 (m, 4H). MS (ESI) *m*/*z*: 345 (M + H)^+^.

#### Methyl 6-(5-(2,3-Bis(*tert*-butoxycarbonyl)guanidino)pentanamido)-4-hydroxy-2-naphthoate (**19a**)

Compound **19a** was obtained as a pale yellow solid (301 mg, 58%) by the reaction of **18a** (400 mg, 0.929 mmol) and 1,3-bis(*tert*-butoxycarbonyl)-2-methyl-2-thiopseudourea (540 mg, 1.86 mmol) following the procedure described for **5f**. ^1^H NMR (400 MHz, DMSO-*d*_6_) δ 11.50 (s, 1H, exchangeable with D_2_O), 10.42 (s, 1H, exchangeable with D_2_O), 10.20 (s, 1H, exchangeable with D_2_O), 8.53 (s, 1H), 8.32 (br t, 1H, exchangeable with D_2_O), 7.99 (s, 1H), 7.94 (d, *J* = 8.8 Hz, 1H), 7.70 (d, *J* = 8.8 Hz, 1H), 7.32 (s, 1H), 3.86 (s, 3H), 2.40 (m, 2H), 1.73–1.51 (m, 6H), 1.47 (s, 9H), 1.38 (s, 9H). MS (ESI) *m*/*z*: 559 (M + H)^+^.

#### Methyl 6-(6-(2,3-bis(*tert*-Butoxycarbonyl)guanidino)hexanamido)-4-hydroxy-2-naphthoate (**19b**)

Compound **19b** was obtained as a yellow product (422 mg, 76%) by the reaction of compound **18b** (433 mg, 0.974 mmol) with 1,3-bis(*tert*-butoxycarbonyl)-2-methyl-2-thiopseudourea (565 mg, 1.94 mmol) following the procedure described for **5f**. ^1^H NMR (400 MHz, DMSO-*d*_6_) δ 11.49 (s, 1H, exchangeable with D_2_O), 10.40 (s, 1H, exchangeable with D_2_O), 10.16 (s, 1H, exchangeable with D_2_O), 8.53 (s, 1H), 8.32 (br t, 1H, exchangeable with D_2_O), 7.99 (s, 1H), 7.94 (d, *J* = 8.8 Hz, 1H), 7.70 (d, *J* = 8.8 Hz, 1H), 7.32 (s, 1H), 3.86 (s, 3H), 3.30–3.22 (m, 2H), 2.40 (t, *J* = 7.4 Hz, 2H), 1.73–1.51 (m, 6H), 1.47 (s, 9H), 1.38 (s, 9H). MS (ESI) *m*/*z*: 573 (M + H)^+^.

#### Methyl 6-(7-(2,3-bis(*tert*-Butoxycarbonyl)guanidino)heptanamido)-4-hydroxy-2-naphthoate (**19c**)

Compound **19c** was obtained as a yellow product (226 mg, 85%) by the reaction of compound **18c** (250 mg, 0.545 mmol) with 1,3-bis(*tert*-butoxycarbonyl)-2-methyl-2-thiopseudourea (317 mg, 1.09 mmol) following the procedure described for **5f**. ^1^H NMR (400 MHz, DMSO-*d*_6_) δ 11.50 (s, 1H, exchangeable with D_2_O), 10.42 (s, 1H, exchangeable with D_2_O), 10.20 (s, 1H, exchangeable with D_2_O), 8.53 (s, 1H), 8.32 (br t, 1H, exchangeable with D_2_O), 7.99 (s, 1H), 7.94 (d, *J* = 8.8 Hz, 1H), 7.70 (d, *J* = 8.8 Hz, 1H), 7.32 (s, 1H), 3.86 (s, 3H), 2.40 (m, 2H), 1.72–1.55 (m, 2H), 1.53–1.44 (m, 11 H), 1.38–1.33 (m, 13 H). MS (ESI) *m*/*z*: 587 (M + H)^+^.

#### Methyl 6-(5-(1,2-Bis(*tert*-butoxycarbonyl)-3-methylguanidino)pentanamido)-4-hydroxy-2-naphthoate (**20a**)

Compound **20a** was obtained as a yellow product (280 mg, 53%) by the reaction of compound **18a** (400 mg, 0.929 mmol) with *N*,*N*′-bis(*tert*-butoxycarbonyl)-*N*,*S*-dimethylisothiourea (566 mg, 1.86 mmol) following the procedure described for **5f**. ^1^H NMR (400 MHz, DMSO-*d*_6_) δ 10.44 (s, 1H, exchangeable with D_2_O), 10.20 (s, 1H, exchangeable with D_2_O), 8.53 (s, 1H), 8.11–7.82 (m, 3H, 1H exchangeable with D_2_O), 7.70 (d, *J* = 9.1 Hz, 1H), 7.32 (s, 1H), 3.86 (s, 3H), 3.17–3.11 (m, 2H), 2.87 (s, 3H), 2.39–2.31 (m, 2H), 1.74–1.48 (m, 4H), 1.36 (m, 18H). MS (ESI) *m*/*z*: 573 (M + H)^+^.

#### Methyl 6-(6-(2,3-Bis(*tert*-Butoxycarbonyl)-3-methylguanidino)hexanamido)-4-hydroxy-2-naphthoate (**20b**)

Compound **20b** was obtained as a yellow product (456 mg, 72%) by the reaction of compound **18b** (480 mg, 1.08 mmol) with *N*,*N*′-bis(*tert*-butoxycarbonyl)-*N*,*S*-dimethylisothiourea (657 mg, 2.16 mmol) following the procedure described for **5f**. ^1^H NMR (400 MHz, DMSO-*d*_6_) δ 10.44 (s, 1H, exchangeable with D_2_O), 10.20 (s, 1H, exchangeable with D_2_O), 8.53 (s, 1H), 8.00 (s, 1H), 7.95 (d, *J* = 8.9 Hz, 1H), 7.71 (dd, *J* = 8.9 Hz, 1H), 7.32 (s, 1H), 3.86 (s, 3H), 3.15–3.11 (m, 2H), 2.87 (s, 3H), 2.38 (t, *J* = 7.4 Hz, 2H), 1.74–1.48 (m, 6H), 1.38 (s, 9H), 1.36 (s, 9H). MS (ESI) *m*/*z*: 587 (M + H)^+^.

#### Methyl 4-Acetoxy-6-amino-2-naphthoate (**21**)

Compound **21** was obtained as a pale yellow solid (2.69 g, 98%) starting from **13** (3.00 g, 10.37 mmol) following the procedure described for **16**. ^1^H NMR (400 MHz, DMSO-*d*_6_) δ 8.28 (s, 1H), 7.86 (d, *J* = 9 Hz, 1 H), 7.54 (s, 1 H), 7.06 (d, *J* = 9 Hz, 1 H), 6.79 (s, 1H), 6.04 (s, 2 H, exchangeable with D_2_O), 3.87 (s, 3 H), 2.42 (s, 3H). MS (ESI) *m*/*z*: 260 (M + H)^+.^

#### Methyl 4-Acetoxy-6-((phenoxycarbonyl)amino)-2-naphthoate (**22**)

Compound **21** (2.68 g, 10.33 mmol) was dissolved in AcOEt, and then TEA (1.58 mL, 11.37 mmol) was added. The resulting mixture was cooled at 0 °C, and phenyl chloroformate (1.94 mL, 15.50 mmol) was added dropwise. The yellow suspension obtained was allowed to warm to room temperature and stirred for 16 h. The reaction was then diluted with AcOEt (60 mL), washed with water (3 × 20 mL), 1N HCl (3× 20 mL), NaHCO_3_ saturated solution (3 × 20 mL), and brine (30 mL), dried over Na_2_SO_4_, filtered, and concentrated under reduced pressure. The resulting solid was crystallized from AcOEt to give the title compound **22** as a pale yellow solid (2.74 g, 70%).

^1^H NMR (400 MHz, DMSO-*d*_6_) δ 10.74 (s, 1H, exchangeable with D_2_O), 8.50 (s, 1H), 8.23–8.14 (m, 2H), 7.79–7.72 (m, 2H), 7.49–7.43 (m, 2H), 7.33–7.24 (m, 3H), 3.91 (s, 3H), 2.41 (s, 3H). MS (ESI) *m*/*z*: 380 (M + H)^+^.

#### Methyl 6-(3-(3-((*tert*-Butoxycarbonyl)amino)propyl)ureido)-4-hydroxy-2-naphthoate (**23a**)

To a stirring solution of methyl 4-acetoxy-6-((phenoxycarbonyl)amino)-2-naphthoate **22** (856 mg, 2.26 mmol) in dry DMF (7 mL), a solution of *tert*-butyl (3-aminopropyl)carbamate (786 mg, 4.51 mmol) and TEA (629 μL, 4.51 mmol) in dry DMF (7 mL) was added, and the reaction was stirred at room temperature for 2 h. NaHCO_3_ saturated solution was added (25 mL), and the resulting mixture was extracted with AcOEt (3 × 25 mL). The combined organic layers were washed with NaHCO_3_ saturated solution (2 × 10 mL) and brine (10 mL), dried over Na_2_SO_4_, filtered, and concentrated under reduced pressure to give a light brown solid that was purified by flash chromatography (gradient AcOEt/MeOH 100:0 to 90:10) yielding a mixture of acetylated and deacetylated compounds. Therefore, the solid was suspended in DCM (30 mL), and piperidine (580 μL) was added. After 1 h, the solvent was removed, and the crude product was diluted with AcOEt (50 mL). The organic layer was washed with 1 N HCl (3× 20 mL) and brine (30 mL), dried over Na_2_SO_4_, filtered, and concentrated under reduced pressure, yielding pure **23a** as an orange solid (754 mg, 80%). ^1^H NMR (400 MHz, DMSO-*d*_6_) δ 10.28 (s, 1H, exchangeable with D_2_O), 8.91 (s, 1H, exchangeable with D_2_O), 8.27 (d, *J* = 2.1 Hz, 1H), 7.97 (s, 1H), 7.89 (d, *J* = 8.9 Hz, 1H), 7.55 (dd, *J* = 8.9, 2.1 Hz, 1H), 7.30 (s, 1H), 6.85 (t, *J* = 5.0 Hz, 1H, exchangeable with D_2_O), 6.25 (t, *J* = 5.7 Hz, 1H, exchangeable with D_2_O), 3.87 (s, 3H), 3.14–3.08 (m, 2H), 3.01–2.94 (m, 2H), 1.59–1.50 (m, 2H), 1.40 (s, 9H). MS (ESI) *m*/*z*: 418 (M + H)^+^.

#### Methyl 6-(3-(4-((*tert*-Butoxycarbonyl)amino)butyl)ureido)-4-hydroxy-2-naphthoate (**23b**)

Compound **23b** was obtained as a yellow solid (828 mg, 85%) by the reaction of **22** (856 mg, 2.26 mmol) with *tert*-butyl (4-aminobutyl)carbamate (849 mg, 4.51 mmol) following the procedure described for **23a**. ^1^H NMR (400 MHz, DMSO-*d*_6_) δ 10.30 (s, 1H, exchangeable with D_2_O), 8.80 (s, 1H, exchangeable with D_2_O), 8.26 (d, *J* = 2.1 Hz, 1H), 7.97 (s, 1H), 7.90 (d, *J* = 8.9 Hz, 1H), 7.54 (dd, *J* = 8.9, 2.1 Hz, 1H), 7.29 (s, 1H), 6.82 (t, *J* = 5.0 Hz, 1H, exchangeable with D_2_O), 6.22 (t, *J* = 5.7 Hz, 1H, exchangeable with D_2_O), 3.85 (s, 3H), 3.17–3.03 (m, 2H), 3.02–2.86 (m, 2H), 1.53–1.29 (m, 13H). MS (ESI) *m*/*z*: 432 (M + H)^+^.

#### Methyl 6-(3-(5-((*tert*-Butoxycarbonyl)amino)pentyl)ureido)-4-hydroxy-2-naphthoate (**23c**)

Compound **23c** was obtained as a yellow solid (855 mg, 85%) by the reaction of **22** (856 mg, 2.26 mmol) with *tert*-butyl (5-aminopentyl)carbamate (911 mg, 4.51 mmol) following the procedure described for **23a**. ^1^H NMR (400 MHz, DMSO-*d*_6_) δ 10.31 (s, 1H, exchangeable with D_2_O), 8.83 (s, 1H, exchangeable with D_2_O), 8.27 (d, *J* = 2.3 Hz 1H), 7.96 (s, 1H), 7.88 (d, *J* = 8.7 Hz, 1H), 7.52 (dd, *J* = 8.7, 2.3 Hz, 1H), 7.30 (s, 1H), 6.81 (t, *J* = 4.8 Hz, 1H, exchangeable with D_2_O), 6.25 (t, *J* = 5.1 Hz, 1H, exchangeable with D_2_O), 3.85 (s, 3H), 3.17–3.03 (m, 2H), 3.02–2.86 (m, 2H), 1.53–1.29 (m, 15H). MS (ESI) *m*/*z*: 446 (M + H)^+^.

#### Methyl 6-(3-(6-((*tert*-Butoxycarbonyl)amino)hexyl)ureido)-4-hydroxy-2-naphthoate (**23d**)

Compound **23d** was obtained as a yellow solid (882 mg, 85%) by the reaction of **22** (856 mg, 2.26 mmol) with *tert*-butyl (6-aminohexyl)carbamate (974 mg, 4.51 mmol) following the procedure described for **23a**. ^1^H NMR (400 MHz, DMSO-*d*_6_) δ 10.32 (s, 1H, exchangeable with D_2_O), 8.84 (s, 1H, exchangeable with D_2_O), 8.28 (d, *J* = 2.4 Hz 1H), 7.96 (s, 1H), 7.87 (d, *J* = 8.5 Hz, 1H), 7.52 (dd, *J* = 8.5, 2.4 Hz, 1H), 7.28 (s, 1H), 6.80 (t, *J* = 5.1 Hz, 1H, exchangeable with D_2_O), 6.24 (t, *J* = 5.0 Hz, 1H, exchangeable with D_2_O), 3.85 (s, 3H), 3.19–3.05 (m, 2H), 3.03–2.89 (m, 2H), 1.58–1.26 (m, 17H). MS (ESI) *m*/*z*: 460 (M + H)^+^.

#### Methyl 6-(3-(7-((*tert*-Butoxycarbonyl)amino)heptyl)ureido)-4-hydroxy-2-naphthoate (**23e**)

Compound **23e** was obtained as a yellow solid (931 mg, 87%) by the reaction of **22** (856 mg, 2.26 mmol) with *tert*-butyl (7-aminoheptyl)carbamate (1.04 g, 4.51 mmol) following the procedure described for **23a**. ^1^H NMR (400 MHz, DMSO-*d*_6_) δ 10.27 (s, 1H, exchangeable with D_2_O), 8.82 (s, 1H, exchangeable with D_2_O), 8.27 (d, *J* = 2.1 Hz, 1H), 7.97 (s, 1H), 7.88 (d, *J* = 8.9 Hz, 1H), 7.53 (dd, *J* = 8.9, 2.2 Hz, 1H), 7.30 (s, 1H), 6.81 (t, *J* = 5.0 Hz, 1H, exchangeable with D_2_O), 6.26 (t, *J* = 5.2 Hz, 1H, exchangeable with D_2_O), 3.86 (s, 3H), 3.16–3.05 (m, 4H), 1.51–1.42 (m, 4H), 1.53–1.30 (m, 15H). MS (ESI) *m*/*z*: 474 (M + H)^+^.

#### Methyl 6-(3-(3-Aminopropyl)ureido)-4-hydroxy-2-naphthoate (**24a**)

The TFA salt of compound **24a** was obtained as a white solid (578 mg, 80%) starting from **23a** (700 mg, 1.67 mmol) following the procedure described for **5a**. ^1^H NMR (400 MHz, DMSO-*d*_6_) δ 10.29 (s, 1H, exchangeable with D_2_O), 9.02 (s, 1H, exchangeable with D_2_O), 8.28 (d, *J* = 2.1 Hz, 1H), 7.99–7.94 (m, 1H), 7.89 (d, *J* = 8.9 Hz, 1H), 7.69 (br s, 3H, exchangeable with D_2_O), 7.55 (dd, *J* = 8.9, 2.1 Hz, 1H), 7.30 (s, 1H), 6.49 (br t, 1H, exchangeable with D_2_O), 3.86 (s, 3H), 3.29–3.10 (m, 2H), 2.99–2.79 (m, 2H), 1.85–1.58 (m, 2H). MS (ESI) *m*/*z*: 318 (M + H)^+^.

#### Methyl 6-(3-(4-Aminobutyl)ureido)-4-hydroxy-2-naphthoate (**24b**)

The TFA salt of compound **24b** was obtained as a white solid (679 mg, 82%) starting from **23b** (800 mg, 1.86 mmol) following the procedure described for **5a**. ^1^H NMR (400 MHz, DMSO-*d*_6_) δ 10.31 (s, 1H, exchangeable with D_2_O), 8.99 (s, 1H, exchangeable with D_2_O), 8.30 (d, *J* = 2.1 Hz, 1H), 7.98 (s, 1H), 7.86 (d, *J* = 8.9 Hz, 1H), 7.71 (br s, 3H, exchangeable with D_2_O) 7.56 (dd, *J* = 8.9, 2.1 Hz, 1H), 7.31 (s, 1H), 6.45 (br t, 1H, exchangeable with D_2_O), 3.85 (s, 3H), 3.16–3.08 (m, 2H), 2.83–2.74 (m, 2H), 1.66–1.43 (m, 4H). MS (ESI) *m*/*z*: 332 (M + H)^+^.

#### Methyl 6-(3-(5-Aminopentyl)ureido)-4-hydroxy-2-naphthoate (**24c**)

The TFA salt of compound **24c** was obtained as a white solid (728 mg, 83%) starting from **23c** (850 mg, 1.91 mmol) following the procedure described for **5a**. ^1^H NMR (400 MHz, DMSO-*d*_6_) δ 10.34 (s, 1H, exchangeable with D_2_O), 8.95 (s, 1H, exchangeable with D_2_O), 8.26 (d, *J* = 2.1 Hz, 1H), 7.97 (s, 1H), 7.89 (d, *J* = 8.9 Hz, 1H), 7.67 (br s, 3H, exchangeable with D_2_O), 7.54 (dd, *J* = 8.9, 2.1 Hz, 1H), 7.29 (s, 1H), 6.85 (br t, 1H, exchangeable with D_2_O), 3.86 (s, 3H), 3.11 (m, 2H), 2.85–2.73 (m, 2H), 1.63–1.42 (m, 4H), 1.39–1.27 (m, 2H). MS (ESI) *m*/*z*: 346 (M + H)^+^.

#### Methyl 6-(3-(6-Aminohexyl)ureido)-4-hydroxy-2-naphthoate (**24d**)

The TFA salt of compound **24d** was obtained as a white solid (745 mg, 85%) starting from compound **23d** (850 mg, 1.85 mmol) following the procedure described for **5a**. ^1^H NMR (400 MHz, DMSO-*d*_6_) δ 10.34 (s, 1H, exchangeable with D_2_O), 8.92 (s, 1H, exchangeable with D_2_O), 8.28 (d, *J* = 2.1 Hz, 1H), 7.99–7.94 (m, 1H), 7.88 (d, *J* = 8.9 Hz, 1H), 7.68 (br s, 3H, exchangeable with D_2_O), 7.54 (dd, *J* = 8.9, 2.1 Hz, 1H), 7.29 (s, 1H), 6.37 (br t, 1H, exchangeable with D_2_O), 3.86 (s, 3H), 3.16–3.09 (m, 2H), 2.83–2.75 (m, 2H), 1.59–1.46 (m, 6H), 1.39–1.34 (m, 2H). MS (ESI) *m*/*z*: 360 (M + H)^+^.

#### Methyl 6-(3-(7-Aminoheptyl)ureido)-4-hydroxy-2-naphthoate (**24e**)

The TFA salt of compound **24e** was obtained as a white solid (750 mg, 81%) starting from compound **23e** (900 mg, 1.90 mmol) following the procedure described for **5a**. ^1^H NMR (400 MHz, DMSO-*d*_6_) δ 10.29 (s, 1H, exchangeable with D_2_O), 8.82 (s, 1H, exchangeable with D_2_O), 8.27 (d, *J* = 2.2 Hz, 1H), 7.98–7.94 (m, 1H), 7.85 (d, *J* = 8.8 Hz, 1H), 7.68 (br s, 3H, exchangeable with D_2_O), 7.53 (dd, *J* = 8.8, 2.2 Hz, 1H), 7.30 (s, 1H), 6.26 (br t, 1H, exchangeable with D_2_O), 3.86 (s, 3H), 3.16–3.05 (m, 4H), 1.51–1.42 (m, 6H), 1.34–1.30 (m, 4H). MS (ESI) *m*/*z*: 374 (M + H)^+^.

#### Methyl 6-(3-(3-(2,3-Bis(*tert*-butoxycarbonyl)guanidino)propyl)ureido)-4-hydroxy-2-naphthoate (**25a**)

Compound **25a** was obtained as a white solid (62 mg, 31%) by the reaction of **24a** (154 mg, 0.357 mmol) with 1,3-bis(*tert*-butoxycarbonyl)-2-methyl-2-thiopseudourea (207 mg, 0.714 mmol) following the procedure described for **5f**. ^1^H NMR (400 MHz, DMSO-*d*_6_) δ 11.50 (s, 1H, exchangeable with D_2_O), 10.30 (s, 1H, exchangeable with D_2_O), 8.86 (s, 1H, exchangeable with D_2_O), 8.35 (br t, 1H, exchangeable with D_2_O), 8.26 (d, *J* = 2.2 Hz, 1H), 7.96 (s, 1H), 7.87 (d, *J* = 8.9 Hz, 1H), 7.54 (dd, *J* = 8.9, 2.2 Hz, 1H), 7.28 (s, 1H), 6.37 (br t, 1H, exchangeable with D_2_O), 3.85 (s, 3H), 3.35–3.23 (m, 2H) 3.19–3.06 (m, 2H), 1.58–1.49 (m, 11H), 1.39 (s, 9H). MS (ESI) *m*/*z*: 560 (M + H)^+^.

#### Methyl 6-(3-(4-(2,3-Bis(*tert*-butoxycarbonyl)guanidino)butyl)ureido)-4-hydroxy-2-naphthoate (**25b**)

Compound **25b** was obtained as a white solid (59 mg, 30%) by the reaction of **24b** (155 mg, 0.348 mmol) with 1,3-bis(*tert*-butoxycarbonyl)-2-methyl-2-thiopseudourea (202 mg, 0.696 mmol) following the procedure described for **5f**. ^1^H NMR (400 MHz, DMSO-*d*_6_) δ 11.52 (s, 1H, exchangeable with D_2_O), 10.35 (s, 1H, exchangeable with D_2_O), 8.89 (s, 1H, exchangeable with D_2_O), 8.41–8.20 (m, 2H, 1H exchangeable with D_2_O), 7.96 (s, 1H), 7.85 (d, *J* = 8.7 Hz, 1H), 7.58 (dd, *J* = 8.7, 2.1 Hz, 1H), 7.30 (s, 1H), 6.31 (br t, 1H, exchangeable with D_2_O), 3.86 (s, 3H), 3.37–3.22 (m, 2H), 3.20–2.98 (m, 2H), 1.56–1.47 (m, 13H), 1.38 (s, 9H). MS (ESI) *m*/*z* (%): 574 (M + H)^+^.

#### Methyl 6-(3-(5-(2,3-Bis(*tert*-butoxycarbonyl)guanidino)pentyl)ureido)-4-hydroxy-2-naphthoate (**25c**)

Compound **25c** was obtained as a yellow solid (128 mg, 40%) by the reaction of **24c** (250 mg, 0.544 mmol) with 1,3-bis(*tert*-butoxycarbonyl)-2-methyl-2-thiopseudourea (316 mg, 1.09 mmol) following the procedure described for **5f**. ^1^H NMR (400 MHz, DMSO-*d*_6_) δ 11.51 (s, 1H, exchangeable with D_2_O), 10.39 (s, 1H, exchangeable with D_2_O), 9.01 (s, 1H, exchangeable with D_2_O), 8.33–8.24 (m, 2H, 1H exchangeable with D_2_O), 7.95 (s, 1H), 7.88 (d, *J* = 8.9 Hz, 1H), 7.53 (dd, *J* = 8.9, 2.2 Hz, 1H), 7.28 (s, 1H), 6.55 (br t, 1H, exchangeable with D_2_O), 3.85 (s, 3H), 3.35–3.23 (m, 2H) 3.14–3.06 (m, 2H), 1.55–1.44 (m, 15H), 1.38 (s, 9H). MS (ESI) *m*/*z* (%): 588 (M + H)^+^.

#### Methyl 6-(3-(3-(2,3-Bis(*tert*-butoxycarbonyl)-3-methylguanidino)propyl)ureido)-4-hydroxy-2-naphthoate (**26a**)

Compound **26a** was obtained as a white solid (64 mg, 32%) by the reaction of **24a** (150 mg, 0.348 mmol) with *N*,*N*′-bis(*tert*-butoxycarbonyl)-*N*,*S*-dimethylisothiourea (212 mg, 0.696 mmol) following the procedure described for **5f**. ^1^H NMR (400 MHz, DMSO-*d*_6_) δ 10.29 (s, 1H, exchangeable with D_2_O), 8.88 (s, 1H, exchangeable with D_2_O), 8.26 (d, *J* = 2.2 Hz, 1H), 7.96 (s, 1H), 7.88 (d, *J* = 8.9 Hz, 1H), 7.54 (dd, *J* = 8.9, 2.2 Hz, 1H), 7.28 (s, 1H), 6.34 (br t, 1H, exchangeable with D_2_O), 3.86 (s, 3H), 3.20–3.12 (m, 4H), 2.89 (s, 3H), 1.74–1.57 (m, 2H), 1.39 (s, 9H) 1.37 (s, 9H). MS (ESI) *m*/*z*: 574 (M + H)^+^.

#### Methyl 6-(3-(4-(2,3-Bis(*tert*-butoxycarbonyl)-3-methylguanidino)butyl)ureido)-4-hydroxy-2-naphthoate (**26b**)

Compound **26b** was obtained as a white solid (126 mg, 40%) by the reaction of **24b** (240 mg, 0.539 mmol) with *N*,*N*′-bis(*tert*-butoxycarbonyl)-*N*,*S*-dimethylisothiourea (328 mg, 1.08 mmol) following the procedure described for **5f**. ^1^H NMR (400 MHz, DMSO-*d*_6_) δ 10.29 (s, 1H, exchangeable with D_2_O), 8.82 (s, 1H, exchangeable with D_2_O), 8.26 (d, *J* = 2.2 Hz, 1H), 7.96 (s, 1H), 7.88 (d, *J* = 8.9 Hz, 1H), 7.54 (dd, *J* = 8.9, 2.2 Hz, 1H), 7.28 (s, 1H), 6.27 (br t, 1H, exchangeable with D_2_O), 3.86 (s, 3H), 3.16–3.12 (m, 4H), 2.89 (s, 3H), 1.74–1.57 (m, 4H), 1.39 (s, 9H) 1.37 (s, 9H). MS (ESI) *m*/*z*: 588 (M + H)^+^.

#### 9-((4*R*,6*R*)-6-(Aminomethyl)-2,2-dimethyltetrahydrofuro[3,4-*d*][1,3]dioxol-4-yl)-9*H*-purin-6-amine (**27a**)

Pd/C (5 wt % on activated carbon, 0.1 equiv) was added to a solution of **34** (1.00 g, 3.01 mmol) in MeOH (100 mL), and the reaction was stirred under H_2_ (1 atm, balloon) for 16 h. The reaction mixture was filtered and concentrated to give the title compound **27a**, used in the next step without further purification. ^1^H NMR (400 MHz, DMSO-*d*_6_) δ 8.37 (s, 1H), 8.16 (s, 1H), 7.32 (br s, 2H), 6.08 (d, *J* = 3.2 Hz, 1H), 5.45 (dd, *J* = 6.4, 3.3 Hz, 1H), 4.98 (dd, *J* = 6.4, 2.7 Hz, 1H), 4.09 (td, *J* = 5.8, 2.6 Hz, 1H), 2.70 (m, 2H), 1.54 (s, 3H), 1.33 (s, 3H). MS (ESI) *m*/*z*: 307 (M + H)^+^.

#### 9-((4*R*,6*R*)-6-(2-Aminoethyl)-2,2-dimethyltetrahydrofuro[3,4-*d*][1,3]dioxol-4-yl)-9*H*-purin-6-amine (**27b**)

**36** (0.25 g, 0.60 mmol) was dissolved in 10 mL of a solution of DCM/TFA (95:5), and the mixture was stirred at room temperature. After 24 h, the solvent was removed under vacuum, and the crude was resuspended in a solution of citric acid 10%. The aqueous solution was washed with DCM (3 × 25 mL), basified with 2 N NaOH until pH 12, and then extracted with CHCl_3_ (3 × 25 mL). The collected organic phases were dried (Na_2_SO_4_), filtered, and concentrated under vacuum to give **27b** as a white solid (0.30 g, 65%). ^1^H NMR (400 MHz, CDCl_3_) δ 8.35 (s, 1H), 7.89 (s, 1H), 6.03 (d, *J* = 2.4 Hz, 1H), 5.70 (br s, 2H), 5.48 (dd, *J* = 6.5, 2.4 Hz, 1H), 4.90 (dd, *J* = 6.5, 4.1 Hz, 1H), 4.33–4.21 (m, 1H), 2.86–2.72 (m, 2H), 1.90–1.78 (m, 2H), 1.61 (s, 3H), 1.39 (s, 3H). MS (ESI) *m*/*z*: 321 (M + H)^+^.

#### 9-((4*R*,6*R*)-6-((*E*)-3-Aminoprop-1-en-1-yl)-2,2-dimethyltetrahydrofuro[3,4-*d*][1,3]dioxol-4-yl)-9*H*-purin-6-amine (**27c**)

To a stirred solution of compound **39** (0.40 g, 0.86 mmol) in MeOH (2 mL), a solution of hydrazine monohydrate (0.87 g, 1.73 mmol) was added, and the mixture was stirred at room temperature for 16 h. Then, the solvent was evaporated, and the crude reside was resuspended in a solution of 10% citric acid. The aqueous solution was washed with DCM (3 × 25 mL), basified with 2 N NaOH until pH 12, and then extracted with CHCl_3_ (3 × 25 mL). The collected organic phases were dried (Na_2_SO_4_), filtered, and concentrated under vacuum to give the amine **27c** as a white solid (0.26 g, 90%). ^1^H NMR (400 MHz, CDCl_3_) δ 8.35 (s, 1H), 7.87 (s, 1H), 6.08 (d, *J* = 2.0 Hz, 1H), 5.87 (br s, 2H), 5.82–5.79 (m, 1H), 5.75–5.65 (m, 1H), 5.53 (dd, *J* = 6.3, 2.0 Hz, 1H), 4.99 (dd, *J* = 6.4, 3.5 Hz, 1H), 4.71–4.66 (m, 1H), 3.23–3.20 (m, 2H), 1.62 (s, 3H), 1.39 (s, 3H). MS (ESI) *m*/*z*: 333 (M + H)^+^.

#### 9-((4*R*,6*R*)-6-(3-aminopropyl)-2,2-dimethyltetrahydrofuro[3,4-*d*][1,3]dioxol-4-yl)-9*H*-purin-6-amine (**27d**)

Compound **27d** was obtained as a white solid (0.35 g, 99%) starting from **27c** (0.35 g, 1.05 mmol) following the procedure described for **27a**. ^1^H NMR (400 MHz, CDCl_3_) δ 8.37 (s, 1H), 7.89 (s, 1H), 6.06 (d, *J* = 2.4 Hz, 1H), 5.72 (br s, 2H), 5.48 (dd, *J* = 6.5, 2.4 Hz, 1H), 4.95 (dd, *J* = 6.5, 4.1 Hz, 1H), 4.35–4.24 (m, 1H), 2.86–2.72 (m, 2H), 1.97–1.65 (m, 4H), 1.61 (s, 3H), 1.39 (s, 3H). MS (ESI) *m*/*z*: 335 (M + H)^+^.

#### *tert*-Butyl (9-((3a*R*,4*R*,6*R*,6a*R*)-6-((3-*tert*-Butyloxythioureido)methyl)-2,2-dimethyltetrahydrofuro[3,4-*d*][1,3]dioxol-4-yl)-9*H*-purin-6-yl)carbamate (**28a**)

To a cooled solution of *N*,*N*′-bis-*tert*-butoxycarbonylthiourea (1.00 g, 3.62 mmol) dissolved in dry THF, NaH (60% dispersion in mineral oil, 173 mg, 4.34 mmol) was added, and the resulting mixture was stirred at the same temperature for 1 h. Then, TFAA (603 μL, 4.34 mmol) was added, and the stirring continued for an additional 1 h at 0 °C; afterward, amine **27a** (1.61 g, 3.96 mmol) was added, and the resulting suspension was stirred for 16 h. The crude was diluted with brine (50 mL), and the aqueous phase was extracted with AcOEt (3 × 25 mL). The collected organic phases were washed with brine, dried (Na_2_SO_4_), and concentrated under vacuum, yielding a residue that was purified by column chromatography (gradient Hex/AcOEt 80:20 to 40:50 + 10% MeOH) to give **28a** as a white solid (716 mg, 35%). ^1^H NMR (400 MHz, CDCl_3_) δ 9.80 (t, *J* = 4.3 Hz, 1H, exchangeable with D_2_O), 8.76 (s, 1H, exchangeable with D_2_O), 8.07 (s, 1H, exchangeable with D_2_O), 7.92 (s, 1H), 7.84 (s, 1H), 6.14 (d, *J* = 2.5 Hz, 1H), 5.42 (dd, *J* = 6.4, 2.5 Hz, 1H), 5.09 (dd, *J* = 6.4, 3.7 Hz, 1H), 4.54–4.50 (m, 1H), 4.18–3.94 (m, 2H), 1.62 (s, 3H), 1.57 (s, 9H), 1.45 (s, 9H), 1.39 (s, 3H). MS (ESI) *m*/*z*: 566 (M + H)^+^.

#### *tert*-Butyl (9-((3a*R*,4*R*,6*R*,6a*R*)-6-((3-*tert*-Butyloxythioureido)ethyl)-2,2-dimethyltetrahydrofuro[3,4-*d*][1,3]dioxol-4-yl)-9*H*-purin-6-yl)carbamate (**28b**)

Compound **28b** was obtained as a white solid (198 mg, 53%) starting from **27b** (350 mg, 1.09 mmol) following the procedure described for **28a**. ^1^H NMR (400 MHz, CDCl_3_) δ 9.77 (br s, 1H, exchangeable with D_2_O), 8.34 (s, 1H), 8.14 (br s, 1H, exchangeable with D_2_O), 7.88 (s, 1H), 6.04 (d, *J* = 2.5 Hz, 1H), 5.89 (br s, 2H, exchangeable with D_2_O), 5.47 (dd, *J* = 6.6, 2.5 Hz, 1H), 4.97 (dd, *J* = 6.6, 4.0 Hz, 1H), 4.29–4.24 (m, 1H), 3.90–3.80 (m, 1H), 3.69–3.59 (m, 1H), 2.17–2.10 (m, 2H), 1.60 (s, 3H), 1.38 (s, 12H). MS (ESI) *m*/*z*: 480 (M + H)^+^.

#### *tert*-Butyl (9-((3a*R*,4*R*,6*R*,6a*R*)-6-((3-*tert*-Butyloxythioureido)propyl)-2,2-dimethyltetrahydrofuro[3,4-*d*][1,3]dioxol-4-yl)-9*H*-purin-6-yl)carbamate (**28c**)

Compound **28c** was obtained as a white solid (191 mg, 35%) starting from **27c** (250 mg, 0.75 mmol) following the procedure described for **28a**. ^1^H NMR (400 MHz, CDCl_3_) δ 9.77 (br s, 1H, exchangeable with D_2_O), 8.34 (s, 1H), 8.14 (br s, 1H, exchangeable with D_2_O), 7.88 (s, 1H), 6.03 (d, *J* = 2.5 Hz, 1H), 5.89 (br s, 2H, exchangeable with D_2_O), 5.45 (dd, *J* = 6.6, 2.5 Hz, 1H), 4.92 (dd, *J* = 6.6, 4.0 Hz, 1H), 4.29–4.24 (m, 1H), 3.70–3.58 (m, 2H), 1.82–1.74 (m, 2H), 1.70–1.64 (m, 2H), 1.61 (s, 3H), 1.47 (s, 9H), 1.38 (s, 3H). MS (ESI) *m*/*z*: 494 (M + H)^+^.

#### *tert*-Butyl (9-((3a*R*,4*R*,6*R*,6a*R*)-6-((3-*tert*-Butyloxythioureido)propyl-1-en)-2,2-dimethyltetrahydrofuro[3,4-*d*][1,3]dioxol-4-yl)-9*H*-purin-6-yl)carbamate (**28d**)

Compound **28d** was obtained as a white solid (121 mg, 33%) starting from **27d** (250 mg, 0.75 mmol) following the procedure described for **28a**. ^1^H NMR (400 MHz, CHCl_3_) δ 9.70 (br s, 1H exchangeable with D_2_O), 8.37 (s, 1H), 8.01 (br s, 1H, exchangeable with D_2_O), 7.88 (s, 1H), 6.09 (d, *J* = 2.1 Hz, 1H), 5.85–5.79 (m, 2H), 5.75 (br s, 2H, exchangeable with D_2_O), 5.51 (dd, *J* = 6.3, 2.0 Hz, 1H), 5.03 (dd, *J* = 6.4, 3.5 Hz, 1H), 4.73–4.66 (m, 1H), 4.23–4.19 (m, 2H), 1.62 (s, 3H), 1.48 (s, 9H), 1.39 (s, 3H). MS (ESI) *m*/*z*: 492 (M + H)^+^.

#### Methyl 6-(3-(3-((*E*)-2-(*tert*-Butoxycarbonyl)-3-(((3a*R*,4*R*,6*R*,6a*R*)-6-(6-((*tert*-butoxycarbonyl)amino)-9*H*-purin-9-yl)-2,2-dimethyltetrahydrofuro[3,4-*d*][1,3]dioxol-4-yl)methyl)guanidino)propyl)ureido)-4-hydroxy-2-naphthoate (**29a**)

To a stirred suspension of **28a** (100 mg, 0.177 mmol) and **24a** (152 mg, 0.354 mmol) in dry DCM, EDC hydrochloride (69 mg, 0.354 mmol) and TEA (74 μL, 0.531 mmol) were added at room temperature. The resulting mixture was stirred for 18 h, then the solvent was evaporated under reduced pressure, and the resulting oil was taken up with water. The aqueous phase was extracted with AcOEt (3 × 25 mL), and the collected organic phases were washed with brine, dried (Na_2_SO_4_), and concentrated under reduced pressure. The crude material was purified by flash chromatography (gradient DCM/MeOH 95:5 to 90:10) yielding **29a** as white solid (114 mg, 75%). ^1^H NMR (400 MHz, DMSO-*d*_6_) δ 10.28 (s, 1H, exchangeable with D_2_O), 10.18 (s, 1H, exchangeable with D_2_O), 8.83 (s, 1H, exchangeable with D_2_O), 8.66–8.62 (m, 2H), 8.24 (d, *J* = 2.2 Hz, 1H), 7.95 (s, 1H), 7.87 (d, *J* = 8.9 Hz, 1H), 7.54 (dd, *J* = 8.9, 2.2 Hz, 1H), 7.28 (s, 1H), 6.32 (br t, 1H, exchangeable with D_2_O), 6.25 (s, 1H), 5.51–5.46 (m, 1H), 5.08–5.02 (m, 1H), 4.34–4.29 (m, 1H), 3.85 (s, 3H), 3.45–3.39 (m, 2H), 3.20–3.08 (m, 4H), 1.70–1.60 (m, 2H), 1.54 (s, 3H), 1.46 (s, 9H), 1.35 (s, 9H), 1.32 (s, 3H). MS (ESI) *m*/*z*: 849 (M + H)^+^.

#### Methyl 6-(3-(4-((*E*)-2-(*tert*-Butoxycarbonyl)-3-(((3a*R*,4*R*,6*R*,6a*R*)-6-(6-((*tert*-butoxycarbonyl)amino)-9*H*-purin-9-yl)-2,2-dimethyltetrahydrofuro[3,4-*d*][1,3]dioxol-4-yl)methyl)guanidino)butyl)ureido)-4-hydroxy-2-naphthoate (**29b**)

Compound **29b** was obtained as a white solid (37 mg, 72%) by the reaction of **28a** (52 mg, 0.060 mmol) with compound **24b** (53 mg, 0.120 mmol) following the procedure described for **29a**. ^1^H NMR (400 MHz, DMSO-*d*_6_) δ 10.27 (s, 1H, exchangeable with D_2_O), 10.15 (s, 1H, exchangeable with D_2_O), 8.86 (s, 1H, exchangeable with D_2_O), 8.69–8.58 (m, 2H), 8.26 (d, *J* = 2.2 Hz, 1H), 7.96 (s, 1H), 7.87 (d, *J* = 8.9 Hz, 1H), 7.55 (dd, *J* = 8.9, 2.2 Hz, 1H), 7.29 (s, 1H), 6.31 (br t, 1H, exchangeable with D_2_O), 6.24 (s, 1H), 5.53–5.45 (m, 1H), 5.12–4.94 (m, 1H), 4.39–4.28 (m, 1H), 3.85 (s, 3H), 3.44–3. 38 (m, 2H), 3.17–3.07 (m, 4H), 1.58–1.51 (m, 4H), 1.49–1.46 (m, 12H), 1.37 (s, 9H), 1.30 (s, 3H). MS (ESI) *m*/*z*: 863 (M + H)^+^.

#### Methyl 6-(3-(5-((*E*)-2-(*tert*-Butoxycarbonyl)-3-(((3a*R*,4*R*,6*R*,6a*R*)-6-(6-((*tert*-butoxycarbonyl)amino)-9*H*-purin-9-yl)-2,2-dimethyltetrahydrofuro[3,4-*d*][1,3]dioxol-4-yl)methyl)guanidino)pentyl)ureido)-4-hydroxy-2-naphthoate (**29c**)

Compound **29c** was obtained as a white solid (197 mg, 77%) by the reaction of compound **28a** (165 mg, 0.292 mmol) with compound **24c** (268 mg, 0.584 mmol) following the procedure described for **29a**. ^1^H NMR (400 MHz, DMSO-*d*_6_) δ 10.29 (s, 1H, exchangeable with D_2_O), 10.15 (s, 1H, exchangeable with D_2_O), 8.86 (s, 1H, exchangeable with D_2_O), 8.64–8.59 (m, 2H), 8.26 (d, *J* = 2.2 Hz, 1H), 7.96 (s, 1H), 7.88 (d, *J* = 8.9 Hz, 1H), 7.55 (dd, *J* = 8.9, 2.2 Hz, 1H), 7.29 (s, 1H), 6.29 (br t, 1H, exchangeable with D_2_O), 6.24 (s, 1H), 5.52–5.46 (m, 1H), 5.13–5.02 (m, 1H), 4.36–4.31 (m, 1H), 3.86 (s, 3H), 3.45–3.39 (m, 2H), 3.21–3.11 (m, 4H), 1.55 (s, 3H), 1.48 (s, 9H), 1.40–1.17 (m, 18H). MS (ESI) *m*/*z*: 878 (M + H)^+^.

#### Methyl 6-(3-(6-((*E*)-2-(*tert*-Butoxycarbonyl)-3-(((3a*R*,4*R*,6*R*,6a*R*)-6-(6-((*tert*-butoxycarbonyl)amino)-9*H*-purin-9-yl)-2,2-dimethyltetrahydrofuro[3,4-*d*][1,3]dioxol-4-yl)methyl)guanidino)hexyl)ureido)-4-hydroxy-2-naphthoate (**29d**)

Compound **29d** was obtained as a white solid (195 mg, 73%) by the reaction of **28a** (170 mg, 0.300 mmol) with compound **24d** (284 mg, 0.600 mmol) following the procedure described for **29a**. ^1^H NMR (400 MHz, DMSO-*d*_6_) δ 10.29 (s, 1H, exchangeable with D_2_O), 10.16 (s, 1H, exchangeable with D_2_O), 8.85 (s, 1H, exchangeable with D_2_O), 8.60–8.54 (m, 2H), 8.27 (d, *J* = 2.3 Hz, 1H), 7.98 (s, 1H), 7.89 (d, *J* = 8.9 Hz, 1H), 7.61 (dd, *J* = 8.9, 2.3 Hz, 1H), 7.33 (s, 1H), 6.34 (br t, 1H, exchangeable with D_2_O), 6.29 (s, 1H), 5.55–5.49 (m, 1H), 5.13–5.05 (m, 1H), 4.34–4.28 (m, 1H), 3.86 (s, 3H), 3.47–3.41 (m, 2H), 3.26–3.13 (m, 4H), 2.31–2.23 (m, 4H), 1.59 (s, 3H), 1.48–1.43 (m, 13H), 1.39- 1.32 (m, 12H). MS (ESI) *m*/*z*: 892 (M + H)^+^.

#### Methyl 6-(3-(7-((*E*)-2-(*tert*-Butoxycarbonyl)-3-(((3a*R*,4*R*,6*R*,6a*R*)-6-(6-((*tert*-butoxycarbonyl)amino)-9*H*-purin-9-yl)-2,2-dimethyltetrahydrofuro[3,4-*d*][1,3]dioxol-4-yl)methyl)guanidino)heptyl)ureido)-4-hydroxy-2-naphthoate (**29e**)

Compound **29e** was obtained as a white solid (201 mg, 72%) by the reaction of compound **28a** (175 mg, 0.309 mmol) with compound **24e** (301 mg, 0.618 mmol) following the procedure described for **29a**. ^1^H NMR (400 MHz, DMSO-*d*_6_) δ 10.31 (s, 1H, exchangeable with D_2_O), 10.25 (s, 1H, exchangeable with D_2_O), 8.79 (s, 1H, exchangeable with D_2_O), 8.63–8.58 (m, 2H), 8.25 (d, *J* = 1.7 Hz, 1H), 7.96 (s, 1H), 7.89 (d, *J* = 8.9 Hz, 1H), 7.51 (dd, *J* = 8.9, 1.7 Hz, 1H), 7.31 (s, 1H), 6.33 (br t, exchangeable with D_2_O), 6.21 (s, 1H), 5.52–5.49 (m, 1H), 5.08–5.04 (m, 1H), 4.37–4.30 (m, 1H), 3.81 (s, 3H), 3.40–3.27 (m, 2H), 3.19–3.09 (m, 4H), 1.54 (s, 3H), 1.51–1.42 (m, 13H), 1.36 (s, 9H), 1.29–1.17 (m, 9H). MS (ESI) *m*/*z*: 906 (M + H)^+^.

#### Methyl 6-(3-(4-((*E*)-3-(2-((3a*R*,4*R*,6*R*,6a*R*)-6-(6-Amino-9*H*-purin-9-yl)-2,2-dimethyltetrahydrofuro[3,4-*d*][1,3]dioxol-4-yl)ethyl)-2-(*tert*-butoxycarbonyl)guanidino)butyl)ureido)-4-hydroxy-2-naphthoate (**30**)

Compound **30** was obtained as a white solid (129 mg, 80%) by the reaction of compound **28b** (100 mg, 0.208 mmol) with compound **24b** (185 mg, 0.416 mmol) following the procedure described for **29a**. ^1^H NMR (400 MHz, DMSO-*d*_6_) δ 10.26 (s, 1H, exchangeable with D_2_O), 8.80 (s, 1H, exchangeable with D_2_O), 8.34–8.30 (m, 2H), 8.25 (d, *J* = 2.1 Hz, 1H), 8.19–8.13 (m, 1H), 7.96 (s, 1H), 7.87 (d, *J* = 8.9 Hz, 1H), 7.54 (dd, *J* = 8.9, 2.1 Hz, 1H), 7.36–7.25 (m, 3H, 2H exchangeable with D_2_O), 6.27 (br t, 1H, exchangeable with D_2_O), 6.11 (s, 1H), 5.47–5.34 (m, 1H), 5.01–4.89 (m, 1H), 4.17–4.05 (m, 1H), 3.86 (s, 3H), 3.20–3.09 (m, 6H), 1.99–1.73 (m, 2H), 1.53 (s, 3H), 1.49–1.42 (m, 4H), 1.35 (s, 9H), 1.31 (s, 3H). MS (ESI) *m*/*z*: 778 (M + H)^+^.

#### Methyl 6-(3-(4-((*E*)-3-(3-((3a*R*,4*R*,6*R*,6a*R*)-6-(6-Amino-9*H*-purin-9-yl)-2,2-dimethyltetrahydrofuro[3,4-*d*][1,3]dioxol-4-yl)propyl)-2-(*tert*-butoxycarbonyl)guanidino)butyl)ureido)-4-hydroxy-2-naphthoate (**31**)

Compound **31** was obtained as a white solid (155 mg, 79%) starting from compound **28c** (123 mg, 0.249 mmol) and compound **24b** (222 mg, 0.498 mmol) following the procedure described for **29a**. ^1^H NMR (400 MHz, DMSO-*d*_6_) δ 10.29 (s, 1H, exchangeable with D_2_O), 8.85 (s, 1H, exchangeable with D_2_O), 8.29–8.21 (m, 2H), 8.26 (d, *J* = 2.2 Hz, 1H), 7.92 (s, 1H), 7.85 (d, *J* = 8.9 Hz, 1H), 7.56 (dd, *J* = 8.9, 2.2 Hz, 1H), 7.30–7.22 (m, 3H, 2H exchangeable with D_2_O), 6.27 (br t, 1H, exchangeable with D_2_O), 6.07 (s, 1H), 5.47–5.44 (m, 1H), 4.89–4.77 (m, 1H), 4.11–4.02 (m, 1H), 3.86 (s, 3H), 3.46–3.37 (m, 2H), 3.17–3.10 (m, 4H), 1.67–1.58 (m, 2H), 1.57–1.44 (m, 9H), 1.37 (s, 9H), 1.30 (s, 3H). MS (ESI) *m*/*z*: 791 (M + H)^+^.

#### Methyl 6-(3-(4-((*E*)-3-((E)-3-((3a*R*,4*R*,6*R*,6a*R*)-6-(6-Amino-9*H*-purin-9-yl)-2,2-dimethyltetrahydrofuro[3,4-*d*][1,3]dioxol-4-yl)allyl)-2-(*tert*-butoxycarbonyl)guanidino)butyl)ureido)-4-hydroxy-2-naphthoate (**32**)

Compound **32** was obtained as a white solid (143 mg, 81%) by the reaction of compound **28d** (110 mg, 0.224 mmol) with compound **24b** (199 mg, 0.448 mmol) following the procedure described for **29a**. ^1^H NMR (400 MHz, DMSO-*d*_6_) δ 10.27 (s, 1H, exchangeable with D_2_O), 8.84 (s, 1H, exchangeable with D_2_O), 8.31–8.23 (m, 2H), 8.16 (d, *J* = 1.9 Hz, 1H), 7.96 (s, 1H), 7.87 (d, *J* = 8.9 Hz, 1H), 7.54 (dd, *J* = 8.9, 1.9 Hz, 1H), 7.33–7.25 (m, 3H, 2H exchangeable with D_2_O), 6.30 (br t, exchangeable with D_2_O), 6.15 (s, 1H), 5.76–5.70 (m, 2H), 5.43 (m, 1H), 4.95 (m, 1H), 4.60–4.55 (m, 1H), 3.85 (s, 3H), 3.78–3.73 (m, 2H), 3.15–3.10 (m, 4H), 1.55 (s, 3H), 1.47–1.38 (m, 4H), 1.36 (s, 9H), 1.31 (s, 3H). MS (ESI) *m*/*z*: 789 (M + H)^+^.

#### ((4*R*,6*R*)-6-(6-Amino-9*H*-purin-9-yl)-2,2-dimethyltetrahydrofuro[3,4-*d*][1,3]dioxol-4-yl)methanol (**33**)

To a suspension of adenosine (2.00 g, 7.50 mmol) in dry acetone (150 mL), 0.75 mL of HClO_4_ was added dropwise. After stirring overnight, the clear solution was treated with a saturated solution of NaHCO_3_ until the pH was 7. The solvent was concentrated under vacuum, and the product was extracted with AcOEt (3 × 25 mL). The collected organic phases were washed with brine (30 mL), dried (Na_2_SO_4_), filtered, and concentrated under vacuum, yielding compound **33** as a white solid (1.86 g, 81%). ^1^H NMR (400 MHz, DMSO-*d*_6_) δ 8.16 (s, 1H), 8.00 (s, 1H), 6.48 (br s, 2H), 6.09 (s, 1H), 5.25 (s, 1H), 5.01 (s, 1H), 4.47 (s, 1H), 4.24 (m, 2H), 1.53 (s, 3H), 1.30 (s, 3H). MS (ESI) *m*/*z*: 308 (M + H)^+^.

#### 9-((4*R*,6*R*)-6-(Azidomethyl)-2,2-dimethyltetrahydrofuro[3,4-*d*][1,3]dioxol-4-yl)-9*H*-purin-6-amine (**34**)

To a solution of **33** (1.00 g, 3.25 mmol) in dry 1,4-dioxane (10 mL), DPPA (1.48 mL, 6.50 mmol) and DBU (1.50 mL, 9.80 mmol) were added dropwise, and the reaction mixture was stirred at room temperature. After 24 h, sodium azide (1.06 g, 16.3 mmol) and 15-crown-5 (0.06 mL, 0.33 mmol) were added, and the resulting mixture was refluxed for 2 h. The mixture was warmed up to room temperature, filtered, and concentrated under vacuum. The residue was taken up with CHCl_3_ (50 mL), washed with water (50 mL) and brine (50 mL), dried (Na_2_SO_4_), filtered, and concentrated under vacuum. The crude brown oil was purified by flash column chromatography (gradient DCM/AcOEt/MeOH 80:20:0 to 20:80:10), yielding the azide **34** as a yellow solid (0.93 g, 86%). ^1^H NMR (400 MHz, DMSO-*d*_6_) δ 8.35 (s, 1H), 8.18 (s, 1H), 7.34 (br s, 2H), 6.21 (d, *J* = 3.2 Hz, 1H), 5.52 (dd, *J* = 6.4, 3.3 Hz, 1H), 5.01 (dd, *J* = 6.4, 2.7 Hz, 1H), 4.31 (td, *J* = 5.8, 2.6 Hz, 1H), 3.68–3.48 (m, 2H), 1.55 (s, 3H), 1.34 (s, 3H). MS (ESI) *m*/*z*: 333 (M + H)^+^.

#### 2-((4*R*,6*R*)-6-(6-Amino-9*H*-purin-9-yl)-2,2-dimethyltetrahydrofuro[3,4-*d*][1,3]dioxol-4-yl)acetonitrile (**35**)

To a solution of **33** (0.76 g, 2.47 mmol) and PPh_3_ (1.62 g, 6.17 mmol) in THF (25 mL), acetone cyanohydrin (0.57 mL, 6.20 mmol) was added. Within 5 min, DEAD (40% in toluene, 2.79 mL, 6.10 mmol) was added at 0 °C. The solution was stirred at the same temperature for 10 min and then warmed to room temperature. After 16 h, the solvent was evaporated, and the crude residue was purified by flash column chromatography (gradient AcOEt/MeOH 99:1 to 80:20), yielding the title compound (0.70 g, 89%) as a brown oil. ^1^H NMR (400 MHz, CDCl_3_) δ 8.35 (s, 1H), 7.88 (s, 1H), 6.09 (s, 1H), 5.60 (br s, 2H), 5.48 (dd, *J* = 6.4, 2.0 Hz, 1H), 5.13 (dd, *J* = 6.3, 3.1 Hz, 1H), 4.56–4.50 (m, 1H), 3.03–2.86 (m, 2H), 1.62 (s, 3H), 1.40 (s, 3H). MS (ESI) *m*/*z*: 317 (M + H)^+^.

#### *tert*-Butyl (2-((4*R*,6*R*)-6-(6-Amino-9*H*-purin-9-yl)-2,2-dimethyltetrahydrofuro[3,4-*d*][1,3]dioxol-4-yl)ethyl)carbamate (**36**)

To a stirred solution of **35** (0.50 g, 1.58 mmol) in MeOH (20 mL), di-*tert*-butyl dicarbonate (1.03 g, 4.74 mmol) and nickel(II) chloride hexahydrate (0.04 g, 0.16 mmol) were added. The solution was cooled at 0 °C, and sodium borohydride (0.42 g, 11.1 mmol) was added portionwise; the black suspension was stirred at room temperature for 4 h. Then, diethylenetriamine (0.17 mL, 1.58 mmol) was added, and the resulting mixture was stirred for 24 h. The solvent was removed under vacuum, and the crude was extracted with AcOEt (3 × 50 mL). The collected organic phases were washed with water (50 mL) and brine (50 mL), dried (Na_2_SO_4_), filtered, and concentrated under vacuum. The crude product was purified by flash column chromatography (gradient DCM/MeOH 99:1 to 90:10), yielding the protected amine **36** as a pale yellow solid (0.53 g, 80%). ^1^H NMR (400 MHz, CDCl_3_) δ 8.36 (s, 1H), 7.87 (s, 1H), 5.99 (d, *J* = 2.6 Hz, 1H), 5.60 (br s, 2H), 5.48 (dd, *J* = 6.6, 2.7 Hz, 1H), 5.03 (s, 1H), 4.91 (dd, *J* = 6.6, 4.0 Hz, 1H), 4.28–4.18 (m, 1H), 3.30–3.11 (m, 1H), 1.97–1.87 (m, 2H), 1.60 (s, 3H), 1.41 (s, 9H), 1.38 (s, 3H). MS (ESI) *m*/*z*: 421 (M + H)^+^.

#### Ethyl (*E*)-3-((4*R*,6*R*)-6-(6-Amino-9*H*-purin-9-yl)-2,2-dimethylperhydrofuro[3,4-*d*][1,3]dioxol-4-yl)acrylate (**37**)

To a solution of **33** (1.50 g, 4.88 mmol) in 15 mL of DMSO, (ethoxycarbonylmethylene)triphenylphosphorane (4.25 g, 12.2 mmol) and *o*-iodoxybenzoic acid (IBX) (1.37 g, 12.2 mmol) were added. The mixture was stirred at room temperature for 72 h. Water (25 mL) was added, and the mixture was extracted with AcOEt (3 × 50 mL). The combined organic phases were dried (Na_2_SO_4_), filtered, and concentrated under vacuum to obtain a crude residue which was purified by column chromatography (AcOEt/MeOH 95:5) to give the title compound as an orange solid (1.28 g, 70%). ^1^H NMR (400 MHz, CDCl_3_) δ 8.33 (s, 1H), 7.87 (s, 1H), 6.96 (dd, *J* = 15.7, 5.5 Hz, 1H), 6.13 (d, *J* = 1.9 Hz, 1H), 5.81 (dd, *J* = 15.6, 1.7 Hz, 1H), 5.61 (br s, 2H), 5.56 (dd, *J* = 6.2, 1.9 Hz, 1H), 5.14 (dd, *J* = 6.3, 3.5 Hz, 1H), 4.84–4.77 (m, 1H), 4.12 (q, *J* = 7.1 Hz, 2H), 1.63 (s, 3H), 1.40 (s, 3H), 1.23 (t, *J* = 7.1 Hz, 3H). MS (ESI) *m*/*z*: 376 (M + H)^+^.

#### (*E*)-3-((4*R*,6*R*)-6-(6-Amino-9*H*-purin-9-yl)-2,2-dimethyltetrahydrofuro[3,4-*d*][1,3]dioxol-4-yl)prop-2-en-1-ol (**38**)

To a solution of **37** (1.00 g, 2.65 mmol) in dry DCM (10 mL), DIBAL-H (1 M in THF, 20 mL, 20.0 mmol) was added dropwise at −78 °C, and the mixture was stirred for 2 h. Then, the reaction was quenched with MeOH (8 mL) and warmed to room temperature. A saturated solution of Rochelle salt (40 mL) was added, and the resulting suspension was stirred overnight; then the aqueous phase was extracted with AcOEt (3 × 25 mL). The collected organic phases were washed with brine (30 mL), dried (Na_2_SO_4_), and filtered. Evaporation under vacuum of the solvent gave the title compound **38** (0.86 g, 98%) as a white yellow solid, which was directly used in the next step without further purification. ^1^H NMR (400 MHz, CDCl_3_) δ 8.36 (s, 1H), 7.87 (s, 1H), 6.10 (d, *J* = 2.0 Hz, 1H), 5.87–5.84 (m, 1H), 5.58–5.53 (m, 3H), 5.02 (dd, *J* = 6.3, 3.4 Hz, 1H), 4.73–4.71 (m, 1H), 4.09–4.06 (m, 2H), 1.63 (s, 3H), 1.40 (s, 3H). MS (ESI) *m*/*z*: 334 (M + H)^+^.

#### 2-((*E*)-3-((4*R*,6*R*)-6-(6-Amino-9*H*-purin-9-yl)-2,2-dimethyltetrahydrofuro[3,4-*d*][1,3]dioxol-4 yl)allyl)isoindoline-1,3-dione (**39**)

To a stirred suspension of **38** (0.48 g, 1.44 mmol), phthalimide (0.21 g, 1.44 mmol), and Ph_3_P (0.38 g, 1.44 mmol) in THF (10 mL), DEAD (40% in toluene, 0.66 mL, 1.44 mmol) was added dropwise. After stirring for 1.5 h at room temperature, a colorless solid started to precipitate. Stirring was continued for 1 h, after which the mixture was cooled to 0 °C and filtered. The residue was washed with Et_2_O and dried in vacuum to give **39** (0.47 g, 70%) as a white solid. ^1^H NMR (400 MHz, CDCl_3_) δ 8.24 (s, 1H), 7.86–7.83 (m, 3H), 7.74–7.71 (m, 2H), 6.07 (d, *J* = 2.0 Hz, 1H), 5.87–5.80 (m, 1H), 5.77–5.68 (m, 1H), 5.60 (br s, 2H), 5.48 (dd, *J* = 6.3, 2.0 Hz, 1H), 4.98 (dd, *J* = 6.3, 3.3 Hz, 1H), 4.71–4.65 (m, 1H), 4.21–4.18 (m, 2H), 1.59 (s, 3H), 1.37 (s, 3H). MS (ESI) *m*/*z*: 463 (M + H)^+^.

### AlphaLISA PRMT1 Activity Assay

PRMT1 activity assays were performed by taking advantage of the AlphaLISA homogeneous proximity assay.

The assays were performed in white opaque OptiPlate-384 (PerkinElmer, #6007299) at 22 °C in a final volume of 25 μL, using the following assay buffer: 30 mM Tris–HCl pH 8.0, 1 mM DTT, 0.01% BSA, 0.01% Tween-20.

In each well, 3 μL of human recombinant PRMT1 (BPS BioScience, #51041) (final concentration, 0.9 nM) was first incubated for 30 min with 2.5 μL of each compound (dissolved in DMSO and diluted in assay buffer to obtain 1% DMSO); then, 4.5 μL of a mixture of histone H4 (1–21) peptide biotinylated (AnaSpec, # 62555) (final concentration, 100 nM) and SAM (Sigma, #A7007) (final concentration, 2 μM) was added. The reaction was incubated for 60 min, and then it was stopped by the addition of 5 μL of antimethyl-histone H4 arginine 3 (H4R3me) AlphaLISA acceptor beads (PerkinElmer, #AL150) (final concentration, 20 μg/mL) diluted in Epigenetic Buffer 1X (PerkinElmer, #AL008). After an incubation of 60 min, 10 μL of streptavidin donor beads (PerkinElmer, #6760002) diluted in Epigenetic Buffer 1X was added in each well (final concentration, 20 μg/mL). After an incubation of 30 min, signals were read in Alpha mode with a PerkinElmer EnSight multimode microplate reader (excitation at 680 nm and emission at 615 nm).

For each incubation step, the OptiPlate was sealed with protective foil to prevent evaporation and contamination. Donor and acceptor beads were added in subdued light.

The 100% activity (positive control) was reached using the vehicle (DMSO) while the 0% activity (negative control) was obtained without the protein. Data were analyzed using Excel software. Values obtained for each compound are mean ± SD determined for three separate experiments.

### Radioisotope-Based IC_50_ Profiling against PRMTs

The effects of compounds **12a**–**12h** on the catalytic activity of PRMT1, PRMT3, PRMT4, PRMT5, PRMT6, PRMT7, and PRMT8 were determined with a HotSpot PRMT activity assay by Reaction Biology Corporation (Malvern, PA) according to the company’s standard operating procedure.^119,120^ Briefly, the full-length human recombinant proteins PRMT1 (residues 2–371, C-terminus; with an N-terminal GST-tag; MW = 68.3 kDa; Genbank Accession # NM_001536) or PRMT3 (residues 2–531, C-terminus; with an N-terminal His-tag; MW = 62.0 kDa; Genbank Accession # NM_005788), or PRMT4 (residues 2–608, C-terminus; with an N-terminal GST-tag; MW = 91.7 kDa; Genbank Accession # NM_199141), or PRMT5/MEP50 complex^121,122^ (residues PRMT5 2–637, C-terminus, and MEP50 2–342, C-terminus; with an N-terminal FLAG-tag, PRMT5, or His-tag, MEP50; MW = 73.7/39.9 kDa; Genbank Accession #NM_006109, #NM_006109), or PRMT6 (residues 2–375, C-terminus; with an N-terminal GST-tag; MW = 67.8 kDa; Genbank Accession # NM_018137), or PRMT7 (residues 2–692, C-terminus; with an N-terminal His-tag; MW = 81.7 kDa; Genbank Accession # NM_019023), or ΔN(1–60)-PRMT8^123^ (residues 61–394, C-terminus; with C- and N-terminal His-tags; MW = 43.2 kDa; Genbank Accession # NM_019854) were added to a solution of the proper substrate (histone H4 for PRMT1, PRMT3, and PRMT8; histone H3.3 for PRMT4; histone H2A for PRMT5/MEP50; GST-GAR for PRMT6 and PRMT7; final concentration 5 μM) in freshly prepared reaction buffer (50 mM Tris-HCl (pH 8.5), 5 mM MgCl_2_, 50 mM NaCl, 1 mM DTT, 1 mM PMSF, 1% DMSO) and gently mixed. The proper solution of compounds **12a**–**12h** in DMSO was delivered into the PRMT reaction mixture by using an Acoustic Technology instrument (Echo 550, LabCyte Inc. Sunnyvale, CA) in the nanoliter range and incubated for 20 min at room temperature. Then, ^3^H-SAM (final concentration 1 μM) was delivered into the reaction mixture to initiate the reaction. After incubation for 60 min at 30 °C, the reaction mixture was delivered to filter paper for detection (as assessed by scintillation). Data were analyzed using Excel and GraphPad Prism 6.0 software (GraphPad Software Inc., San Diego, CA) for IC_50_ curve fits using sigmoidal dose vs response-variable slope (four parameters) equations.

### Selectivity Assay against KMTs

The effects of compound **12h** on the catalytic activity of ASH1L/KMT2H, EZH2/KMT6, MLL1/KMT2A, SET7/9/KMT7, SETD8/KMT5A, SUV39H2/KMT1B, SUV420H1/KMT5B, and DOT1L/KMT4 were determined with a HotSpot KMT activity assay by the Reaction Biology Corporation (Malvern, PA) according to the company’s standard operating procedure.^119,120^ Briefly, the human recombinant ASH1L (residues 2046–2330, with an N-terminal His-tag; MW = 35.4 kDa; Genbank Accession # NM_018489), or the human recombinant EZH2-containing five-member polycomb repressive complex 2 (including EZH2 residues 2–746, AEBP2 2–517, EED 2–441, RbAp48 2–425, SUZ12 2–739; all full-length; with N-terminal Flag-tag on EED and N-terminal His-tag on all others; MW = 333.8 kDa; Genbank Accession # NM_001203247, NM_001114176, NM_003797, NM_005610, NM_015355), or the human recombinant MLL1 complex (including MLL1 residues 3745–3969, C-terminus, WDR5 22–334, C-terminus, RbBP5 1–538, C-terminus, ASH2L 2–534, C-terminus, DPY-30 1–99, C-terminus; N-terminal His-tag on all subunits; MW = 212.0 kDa; Genbank Accession # NM_005933, NM_017588, NM_005057, NM_001105214, NM_0325742), or the human recombinant SET7/9 (residues 2–366, C-terminus; with a N-terminal GST-tag and a C-terminal His-tag; MW = 68.5 kDa; Genbank Accession # NM_030648), or the human recombinant SETD8 (residues 190–352, C-terminus; N-terminal His-tag; MW = 22.0 kDa; Genbank Accession # NM_020382), or the human recombinant SUV39H2 (residues 46–410, C-terminus; N-terminal fusion protein with a C-terminal His-tag; MW = 98.8 kDa; GenBank Accession No. NM_001193424), or the human recombinant SUV420H1-tv2 (residues 2–393; C-terminus; N-terminal GST-tag; MW = 73.2 kDa; GenBank Accession No. NM_016028), or the human recombinant DOT1L (residues 1–416; N-terminal GST-tag; MW = 80.0 kDa; Genbank Accession # NM_032482) were added to a solution of the proper substrate (oligonucleosomes for ASH1L, MLL1 complex, SETD8, SUV420H1 and DOT1L, final concentration 0.05 mg/mL; core histone for EZH2 complex and SET7/9, final concentration 0.05 mg/mL; histone H3 for SUV39H2, final concentration 5 μM) in freshly prepared reaction buffer (50 mM Tris-HCl (pH 8.5), 5 mM MgCl_2_, 50 mM NaCl, 1 mM DTT, 1 mM PMSF, 1% DMSO) and gently mixed. The proper solution (1 or 10 μM fixed concentrations) of compound **12h** in DMSO was delivered into the KMT reaction mixture by using Acoustic Technology (Echo 550, LabCyte Inc. Sunnyvale, CA) in nanoliter range and incubated for 20 min at room temperature. Then, ^3^H-SAM (final concentration 1 μM) was delivered into the reaction mixture to initiate the reaction. After incubation for 60 min at 30 °C, the reaction mixture was delivered to filter-paper for detection (as assessed by scintillation). SAH,^78−80^ chaetocin (for ASH1L),^81^ or ryuvidine (for SETD8)^82^ were used as reference compounds and tested in 10-dose IC_50_ mode with threefold serial dilution starting at 100 μM. No inhibitor control (DMSO) was considered as 100% enzyme activity. Data were analyzed using Excel and GraphPad Prism 6.0 software (GraphPad Software Inc., San Diego, CA). Values obtained for each compound are mean ± SD determined for two separate experiments.

### PRMT4 SPR Experiments

SPR experiments were performed on a Biacore T200 biosensor (Cytiva). PBS buffer (phosphate-buffered saline, pH 7.4) supplemented with 0.05% Tween-20 was used as the running buffer. Full-length recombinant PRMT4 (Active motif, # 81107) was diluted at the concentration of 50 μg/mL in 10 mM sodium acetate, pH 4.5, and then immobilized on a Series S Sensor Chip CM5 at a flow rate of 10 μL/min by using standard amine-coupling protocols to obtain densities of 4.6 kRU. One flow cell was left empty for background subtractions. Compounds **12f**–**12h** were diluted in PBS supplemented with 0.05% Tween-20, keeping a final 2% DMSO concentration. Each compound was tested in 12 serial dilutions, prepared starting from 640 to 10 nM. Binding experiments were performed at 25 °C by using a flow rate of 30 μL/min, with 90 s monitoring of association and 90 s monitoring of dissociation. Regeneration of the surfaces was performed, when necessary, by a 10 s injection of 5 mM NaOH. The sensorgrams obtained at the 12 concentrations of each compound were first corrected taking advantage of the solvent correction performed by the instrument (correction range from 1.5% to 2.8% DMSO), and then they were double referenced. The corrected sensorgrams for each compound were fitted simultaneously using a 1:1 Langmuir model to obtain equilibrium dissociation constants (*K*_D_) and kinetic dissociation (*k*_off_) and association (*k*_on_) constants.

### PAINS Analysis

Compounds **5**–**12** were analyzed for known classes of assay interference compounds.^68^ All derivatives were not recognized as PAINS according to the SwissADME web tool (http://www.swissadme.ch),^69^ the Free ADME-Tox Filtering Tool (FAF-Drugs4) program (http://fafdrugs4.mti.univ-paris-diderot.fr/),^70^ and the “False Positive Remover” software (http://www.cbligand.org/PAINS/)^71^ nor as aggregators according to the software “Aggregator Advisor” (http://advisor.bkslab.org/).^72^

### Crystallography

#### PRMT4 Cloning, Expression, and Purification

The *Mus musculus* PRMT4 gene sequence corresponding to the PRMT core (residues 130 to 487 or 497, *mm*PRMT4_130–487_ or *mm*PRMT4_130–497_, respectively) were amplified by PCR from the original GST-PRMT4 construct. The sequences were cloned in the pDONR207 (Invitrogen) vector using a BP reaction (Gateway Cloning, Life Technologies). The positive clones were confirmed by sequencing (GATC). The sequences were subcloned in a pDEST20 vector using an LR reaction. The resulting recombinant proteins harbor an amino-terminal glutathione S-transferase (GST) tag followed by a Tobacco etch virus (TEV) protease cleavage site. DH10Bac competent cells containing the baculovirus genome were transformed with the pDEST20-PRMT4 plasmids and plated onto LB agar media containing 15 μg mL^–1^ tetracycline, 7 μg mL^–1^ gentamicin, 50 μg mL^–1^ kanamycin, 25 μg mL^–1^ X-Gal, and 40 μg mL^–1^ IPTG. Bacmid DNA purified from recombination-positive white colonies was transfected into *Sf*9 cells using the Lipofectin reagent (Invitrogen). Viruses were harvested 10 days after transfection. *Sf*9 cells were grown at 300 K in suspension culture in Grace medium (Gibco) using Bellco spinner flasks. *Sf*9 cell culture (1 L, at 0.8 × 10^6^ cells mL^–1^) was infected with recombinant GST-*mm*PRMT4 viruses with an infection multiplicity of 1. Cells were harvested 48 h postinfection. Cell lysis was performed by sonication in 50 mL of buffer A [50 mM Tris-HCl pH 8.0, 250 mM NaCl, 5% glycerol, 5 mM TCEP, 0.01% NP40 and antiproteases (Roche, cOmplete, EDTA-free)], and cellular debris was sedimented by centrifugation of the lysate at 40 000*g* for 30 min. The supernatant was incubated overnight at 277 K with 2 mL of glutathione Sepharose resin (GE Healthcare). After a short centrifugation, the supernatants were discarded, and the beads were poured in an Econo-column (Bio-Rad). After two wash steps with 10 mL of buffer A, 2 mL of buffer A supplemented with in-house produced TEV protease was applied to the columns, and digestion was performed for 4 h at 303 K with gentle mixing. The digest was concentrated with an Amicon Ultra 10K (Millipore), loaded on a gel-filtration column (HiLoadTM 16/60 SuperdexTM S200, GE Healthcare), and eluted at 1 mL min^–1^ with buffer B (20 mM Tris-HCl pH 7.5, 50 mM NaCl, 1 mM TCEP) using an ÄKTA Purifier device (GE Healthcare). Fractions containing *mm*PRMT4 were pooled and concentrated to 5 mg mL^–1^.

#### PRMT4 Crystallization, Data Collection, and Structure Determination

The inihibitors were solubilized in water before addition to the protein solution (2–3 mg mL^–1^) at the final concentration of 0.5–2 mM. A vapor diffusion method utilizing hanging drop trays (VDX plate, Hampton Research) with a 0.5 mL reservoir was used for crystallization. Typically, 2 μL of protein–ligand solution was added to 1 μL of a well solution consisting of (1) 100 mM Tris-HCl pH 8.0, 10% (v/v) PEG 2000 MME for cocrystallization with **12a**; (2) 100 mM Tris-HCl pH 8.0, 10% (v/v) PEG 2000 MME and 100 mM NaCl for cocrystallization with **12b** and **12c**; (3) 100 mM Tris-HCl pH 8.5, 20% (v/v) PEG 3350 and 200 mM ammonium sulfate for cocrystallization with **12f** and **12h**; and (4) 100 mM Tris-HCl pH 8.5, 20% (v/v) PEG 3350 and 100 mM NaCl for cocrystallization with **12g**. Crystals grew in a few days at 293 K. Crystals were flash-frozen in liquid nitrogen after a brief transfer to 5 μL of reservoir solution containing 15% (v/v) PEG 400 as a cryoprotectant and were stored in liquid nitrogen.

The diffraction data sets were collected using SOLEIL PROXIMA1 and PROXIMA2 and ESRF ID30-B beamlines, on a Pilatus 6M, EIGER 9M, and EIGER 6 M (Dectris) detector, respectively, and processed with XDS^124^ and HKL-2000.^125^ The crystals belonged to the *P*2_1_2_1_2 space group with four monomers of *mm*PRMT4 in the asymmetric unit. The structures were solved by molecular replacement using a *mm*PRMT4 structure as a probe.^106^ Model building and refinement were carried out using Coot,^126^ PHENIX,^127−129^ and BUSTER.^130^ TLS refinement with 20 groups per polypeptide chain was used. All other crystallographic calculations were carried out with the CCP4 package.^131^ A summary of the data statistics for the solved and structures is provided in Table 4. The atomic coordinates and experimental data (PDB ID XXXX) have been deposited in the Protein Data Bank. Structure figures were generated with PyMol (http://www.pymol.org). Initial 3D structures of compounds **12a**–**12c** and **12f**–**12h** were generated using the Marvin suite, Marvin version 5.2.3_1, ChemAxon (https://www.chemaxon.com). Stereochemical restraint dictionaries for all compounds have been produced using the grade Web Server (http://grade.globalphasing.org).

#### PRMT6 Cloning, Expression, and Purification

The *Mus musculus* PRMT6 gene sequence (UNP Q6NZB1) corresponding to the residues 34–378 of the protein was cloned in the pnEA-vHis or pnEA/vHis vector (TEV protease cleavable hexahistidine tag at the amino- or carboxy-terminus of the recombinant protein, respectively)^132^ between the NdeI and XhoI restriction sites and used to transform *Escherichia coli* BL21 (DE3) cells. The protein also contains an F315L point mutation, reported for the entry as a natural variant. The construction in the pnEA/vHis plasmid also contains a C53S point mutation to prevent a disufide bond formation with the C232 in the second monomer of the homodimer. The protein expression was induced for 4 h at 310 K, with 0.1 mM IPTG in LB medium. The cells were harvested by centrifugation and resuspended in 10 mL per pellet g of buffer A (50 mM Tris-HCl, pH 8.0, 300 mM NaCl, 7 mM β-ME, 20 mM imidazole) supplemented with 1 mM PMSF. After sonication, the lysate was centrifuged at 40 000 × *g* for 25 min at 4 °C. The supernatant was mixed with 0.5 mL of NiNTA superflow resin (Qiagen) per liter of culture, incubated for 1 h at 4 °C, and then loaded on an Econo column (Bio-Rad). After the column was washed with 10 column volumes (c.v.) of buffer A, the bound protein was eluted with 5 c.v. of buffer A supplemented with 500 mM imidazole. The fractions containing the recombinant protein were detected by a Bradford assay, pooled, and supplemented with TEV protease. The sample was then dialyzed in a 10 000 MWCO dialysis membrane (Spectrum) against 1 L of buffer B (50 mM Tris-HCl, pH 8.0, 150 mM NaCl, 7 mM β-ME, and 1 mM EDTA]) for 16 h at 4 °C. The dialyzed sample was loaded onto a HiLoad 16/60 Superdex 200 prep grade column (GE Healthcare) and equilibrated in buffer C (20 mM Tris-HCl, pH 7.5, 150 mM NaCl, and 5 mM TCEP). The protein was finally concentrated on an Amicon Ultra-4 10 000 MWCO filter (Millipore) to a 6 mg mL^–1^ final concentration. Samples were either directly used for crystallization or flash-frozen as 25 μL aliquots in liquid nitrogen and stored at −80 °C.

#### PRMT6 Crystallization, Data Collection, and Structure Determination

EML ligands were solubilized in water before addition to the protein solution (6 mg mL^–1^) at the final concentration of 1 mM. A vapor diffusion method utilizing hanging drop trays (VDX plate, Hampton Research) with a 1 mL reservoir was used for crystallization. Typically, 1 μL of protein–ligand solution was added to 1 μL of well solution consisting of (1) 100 mM Tris-HCl pH 7.5, 200 mM NaNO_3_, and 20% (v/v) PEG 3350 for the cocrystallization with **12a**; (2) 100 mM Tris-HCl pH 8.0, 200 mM HCOONa, and 19% PEG 3350 for the cocrystallization with **12c**; and (3) 100 mM Tris-HCl pH 7.5, 200 mM HCOONa, and 19% PEG 3350 for the cocrystallization with **12f**. Crystals grew in a week at 293 K. Crystals were flash-frozen in liquid nitrogen after a brief transfer to 5 μL of reservoir solution containing 15% (v/v) PEG 400 as a cryoprotectant and were stored in liquid nitrogen.

Data sets were all collected on an in-house RIGAKU FR-X diffraction source, using a PILATUS 4 M detector (Dectris), and processed with XDS. Three structures of *mm*PRMT6 have been solved and refined. The crystals belong to the *P*2_1_ space group with two *mm*PRMT6 molecules in the asymmetric unit. The structures were solved by molecular replacement using previously solved *mm*PRMT6 structures as the search model (PDB ID 4C03).^107^ Iterative cycles of model building and refinement were carried out using Coot, PHENIX, and Buster. A summary of the data statistics for the two solved and refined structures is provided in Table 5. The atomic coordinates and experimental data (PDB ID 7NUD, 7NUE, and 7P2R) have been deposited in the Protein Data Bank.

#### Parallel Artificial Membrane Permeability Assay (PAMPA)

A donor solution (0.2 mM) was prepared by diluting 20 mM dimethyl sulfoxide (DMSO) compound stock solution using phosphate buffer (pH 7.4, 0.01 M). Filters were coated with 5 μL of a 1% (w/v) dodecane solution of *l*-α-phosphatidylcholine. Donor solution (150 μL) was added to each well of the filter plate. To each well of the acceptor plate, 300 μL of solution (5% DMSO in phosphate buffer) was added. Selected compounds were tested in triplicate; propranolol and furosemide were used as positive and negative controls. The sandwich was incubated for 24 h at room temperature under gentle shaking. After the incubation time, the sandwich plates were separated, and 250 μL of the acceptor plate was transferred to a UV quartz microtiter plate and measured by UV spectroscopy, using a Multiskan GO microplate spectrophotometer (Thermo Fisher Scientific) at 250–500 nm at a step of 5 nm. Reference solutions (250 μL) were prepared diluting the sample stock solutions to the same concentration as that with no membrane barrier. The apparent permeability value *P*_app_ is determined from the ratio *r* of the absorbance of the compound found in the acceptor chamber divided by the theoretical equilibrium absorbance (determined independently), the Faller modification^133^ of the Sugano equation:^134^
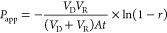
In this equation, *V*_R_ is the volume of the acceptor compartment (0.3 cm^3^), *V*_D_ is the donor volume (0.15 cm^3^), *A* is the accessible filter area (0.24 cm^2^), and *t* is the incubation time in seconds.

#### Cell Viability Assay

The HEK293T cell line was cultured in DMEM (Euroclone) supplemented with 10% (v/v) fetal bovine serum (Euroclone), 100 U/mL penicillin, 100 μg/mL streptomycin (Euroclone), and 2 mM *l*-glutamine (Euroclone) at 37 °C in a 5% CO_2_ atmosphere. Cell viability was measured via a 3-(4,5-dimethylthiazol-2-yl)-2,5-diphenyltetrazolium bromide (MTT) assay. A total of 200 μL of cells seeded in 96-well microtiter plates (5 × 10^4^ cells/mL) was exposed for 24 or 72 h to different concentrations of compounds **12f**–**12h** (10, 50, and 100 μM) in media containing 0.2% DMSO. The mitochondrial-dependent reduction of MTT to formazan was used to assess cell viability. Live cells reduce yellow MTT to purple formazan. The resulting formazan was solubilized in DMSO, and the absorbance was measured at 550 nm and corrected for 620 nm background. Experiments were performed in quadruplicate and all values are expressed as the percentage of the control containing 0.2% DMSO.

#### Western Blotting Method

HEK293T or MCF7 cells were treated with different compounds and then harvested and lysed at the indicated time, and the cell lysates were applied to Western blot analysis. In brief, cells were harvested and washed three times with cold PBS, and then cell lysis buffer (50 mM Tris-HCl, pH 7.5, 150 mM NaCl, 1% Nonidet P-40, 0.1% SDS, 1% sodium deoxycholate, 5 mM EDTA, supplemented with proteinase inhibitor mixture) was added to obtain the cell lysates. Cell debris was pelleted and discarded, whereas the supernatant was kept. Protein samples were added with SDS loading buffer and boiled for 10 min, followed by SDS-PAGE. Then the proteins were transferred to a PVDF membrane. The membrane was blocked with 5% fat-free milk for 1 h at room temperature and then incubated with primary antibodies (antihistone H3, Abcam, #ab18521; pan-PRMT4 substrate antibody, made in-house) at 4 °C overnight. The blot was then washed three times with PBST and incubated with secondary antibodies for 1 h at room temperature. After being washed three times with PBST, the membrane was incubated with ECL reagent, and the signals were detected on X-ray film.

#### Anti-PRMT4 Substrate Antibody

The anti-PRMT4 substrate antibody was raised against the R388 site in the transcription factor NFIB. This antibody recognizes NFIB-Me in a PRMT4-dependent manner but also recognizes additional PRMT4 substrates.^102,108^

#### Cell Proliferation Assay

HEK293T cells were seeded into 96-well plates at the density of 250 cells/well in 100 μL of medium. MCF7 cells were seeded into 96-well plates at the density of 1000 cells/well in 100 μL of medium. Cell proliferation was analyzed at day 0, day 2, day 4, day 6, and day 8, and the medium was changed at day 4. The proliferation analysis was based on viability as determined by CellTiter-Glo (#G7572, Promega) according to the manufacturer’s instructions. Triple independent repeats were conducted.

## Supporting Information Available

Synthesis of intermediates **27a**–**d**, PAMPA assay, additional results from crystallographic studies, and copies of ^1^H NMR and ^13^C NMR spectra of all final compounds (PDF)
PDB validation report for 7PPY (PDF)
PDB validation report for 7PV6 (PDF)
PDB validation report for 7PPQ (PDF)
PDB validation report for 7PU8 (PDF)
PDB validation report for 7PUC (PDF)
PDB validation report for 7PUQ (PDF)
PDB validation report for 7NUD (PDF)
PDB validation report for 7NUE (PDF)
PDB validation report for 7P2R (PDF)
Molecular formula strings (CSV)

## Accession Codes

The atomic coordinates and structure factors of PRMT4 in complex with **12a**–**c** and **12f**–**h** in space group *P*2_1_2_1_2 (PDB ID: 7PV6, 7PPY, 7PPQ, 7PU8, 7PUQ, 7PUC) and of PRMT6 in complex with **12a**, **12c**, and **12f** in space group *P*2_1_ (PDB ID: 7NUD, 7NUE, and 7P2R) have been deposited in the Protein Data Bank, and authors will release the atomic coordinates and experimental data upon article publication.

## Abbreviations Used

AcOEt: ethyl acetate
ACN: acetonitrile
AEBP2: zinc finger protein AEBP2, also known as adipocyte enhancer-binding protein 2
ALPHA: amplified luminescent proximity homogeneous assay
ASH1L: absent small and homeotic disks protein 1 homologue
ASH2L: Set1/Ash2 histone methyltransferase complex subunit ASH2, also known as absent small and homeotic disks protein 2 homologue
CA150: also known as TCERG1
CARM1: coactivator-associated arginine methyltransferase 1
CBP: CREB-binding protein
CREB: cAMP response element-binding protein
DEPTQ: distortionless enhancement by polarization transfer quaternary
DOT1L: DOT1-like (disruptor of telomeric silencing 1-like)
DPY30: protein dpy-30 homologue, also known as Dpy-30-like protein
EDC: 1-ethyl-3-(3-(dimethylamino)propyl)carbodiimide
ECL: enhanced chemiluminescence
EED: polycomb protein EED, also known as embryonic ectoderm development protein
ELAV: embryonic lethal, abnormal vision, Drosophila
ELAVL1: ELAV like RNA binding protein 1
ELAVL4: ELAV like RNA binding protein 4
EZH2: enhancer of zeste homologue 2
GAR: glycine- and arginine-rich motif
HuD: human antigen D, also known as ELAVL4
HuR: human antigen R, also known as ELAVL1
MED12: mediator of RNA polymerase II transcription subunit 12
MLL1: histone-lysine *N*-methyltransferase 2A, also known as myeloid/lymphoid or mixed-lineage leukemia protein 1
MTT: 3-(4,5-dimethylthiazol-2-yl)-2,5-diphenyltetrazolium bromide
NFIB-Me: nuclear factor 1 B-type
p300: E1A-associated protein of 300 kDa
*P*_app_: apparent permeability
PABP1: poly(A)-binding protein 1
Pbf: 2,2,4,6,7-pentamethyldihydrobenzofuran-5-sulfonyl
PBST: phosphate-buffered saline with Tween-20
PMSF: phenylmethylsulfonyl fluoride
PRMT: protein arginine methyltransferase
PRMT1: protein arginine methyltransferase 1
PRMT3: protein arginine methyltransferase 3
PRMT4: protein arginine methyltransferase 4
PRMT5: protein arginine methyltransferase 5
PRMT6: protein arginine methyltransferase 6
PRMT7: protein arginine methyltransferase 7
PRMT8: protein arginine methyltransferase 8
PVDF: pol(vinylidene difluoride)
RBAP48: histone-binding protein RBBP4, also known as retinoblastoma-binding protein p48
RBBP5: retinoblastoma-binding protein 5
SAH: *S*-5′-adenosyl-*l*-homocysteine
SAM: *S*-adenosyl-*l*-methionine
SD: standard deviation
SRC-3: steroid receptor coactivator-3
SET: suppressor of variegation 3-9 enhancer-of-zeste trithorax
SET7/9: SET domain-containing protein 7
SETD8: SET domain-containing protein 8, also known as PR/SET domain-containing protein 07
siRNA: small interference ribonucleic acid
SUV39H2: suppressor of variegation 3-9 homologue 2, also known as Su(var.)3-9 homologue 2
SUV420H1-TV2: suppressor of variegation 4-20 homologue 1, -transcription variant 2
SUZ12: polycomb protein SUZ12, also known as suppressor of zeste 12 protein homologue
TCERG1: transcription elongation regulator 1
WDR5: WD repeat-containing protein 5
