# Supplementary material for: Turning Nonselective Inhibitors of Type I Protein Arginine Methyltransferases into Potent and Selective Inhibitors of Protein Arginine Methyltransferase 4 through a Deconstruction–Reconstruction and Fragment-Growing Approach
Source: J Med Chem. 2022 Apr 28;65(17):11574–606. doi: 10.1021/acs.jmedchem.2c00252 (PMC9469100; doi:10.1021/acs.jmedchem.2c00252)
Supplement: Supplementary file 1 — jm2c00252_si_001.pdf [file jm2c00252_si_001.pdf]

Supporting Information

Turning Non-Selective Inhibitors of Type I Protein Arginine Methyltransferases into Potent and Selective Inhibitors of Protein Arginine Methyltransferase 4 through a Deconstruction-Reconstruction and Fragment Growing Approach

Giulia Iannelli,[‡,∫,‖] Ciro Milite,[‡,‖] Nils Marechal,[§] Vincent Cura,[§] Luc Bonnefond,[§] Nathalie-Troffer-Charlier,[§] Alessandra Feoli,[‡] Donatella Rescigno,[‡,†] Yalong Wang,[∆] Alessandra Cipriano,[‡] Monica Viviano,[‡] Mark T. Bedford,[∆] Jean Cavarelli,[,§] Sabrina Castellano,[*,‡] and Gianluca Sbardella[*,‡]*

[‡]Department of Pharmacy, Epigenetic Med Chem Lab, University of Salerno, via Giovanni Paolo II 132, I-84084 Fisciano (SA), Italy
[§] Department of Integrated Structural Biology, Institut de Génétique et de Biologie Moléculaire et Cellulaire, Illkirch, France; Centre National de la Recherche Scientifique, UMR7104, Illkirch, France; Institut National de la Santé et de la Recherche Médicale, U1258, Illkirch, France; Université de Strasbourg, Illkirch, France.
[∫]PhD Program in Drug Discovery and Development, University of Salerno, Via Giovanni Paolo II 132, I-84084 Fisciano (SA), Italy
[∆] Department of Epigenetics and Molecular Carcinogenesis, The University of Texas MD Anderson Cancer Center, Houston, Texas 77030, United States

*J.C.: E-mail, cava@igbmc.fr; *S.C.: E-mail, scastellano@unisa.it; *G.S.: E-mail, gsbardella@unisa.it

Table of Contents:

Figure S1: SPR interaction analysis of compounds **12b–e** S2

Figure S2: Inhibitory activity profiles of compound **12h** towards a panel of KMTs S3

Table S1. Inhibitory activity values of compound **12h** against a panel of KMTs S3

Figure S3: overview of Tetramer of *mm*PRMT4 with compound **12h** (EML981) bound S4

Figure S4: superimposition of compound **12h** on a transition state mimic-mmPRMT4 S5

Figures S5–S7: protein-ligand interactions between **12a**–**c** and PRMT4 residues S6–S8

Figure S8–S10: protein-ligand interactions between **12f**–**h** and PRMT4 residues S9–S11

Figure S11: superimposition of compounds **12a**, **12c**, **12f** bound to mmPRMT6 S12

Figure S12: Conformational changes of PRMT6 alpha-X helix containing the motif I S12

Table S2. Permeability values of compounds **12f–h** S13

^{1}H-NMR and ^{13}C-NMR spectra of compound **5–12** S14–S81

HPLC Traces for compounds **12a-h** S82-S89

Figure S1. SPR interaction analysis of compounds **12b–e** binding to immobilized PRMT4**.**

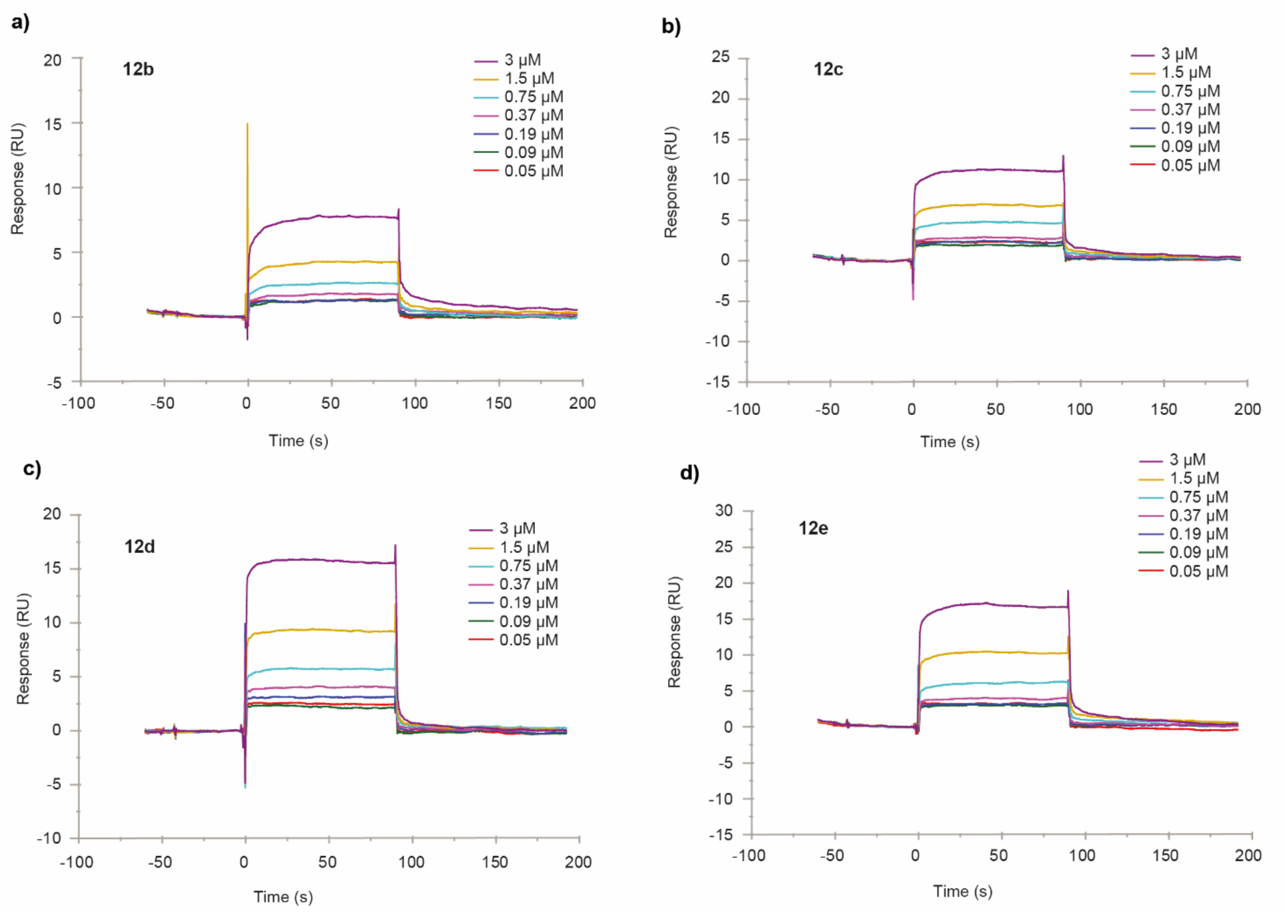

Sensorgrams obtained from the SPR interaction analysis of compounds **12b–e** (panels a–d, respectively) binding to immobilized PRMT4. Each compound was injected at different concentrations (from 3 μM to 0.05 μM) with an association and a dissociation time of 90 sec, with a flow rate of 30 μL/min. The equilibrium dissociation constants (K_D) were derived from the ratio between kinetic dissociation (k_{off}) and association (k_{on}) constants.

Figure S2

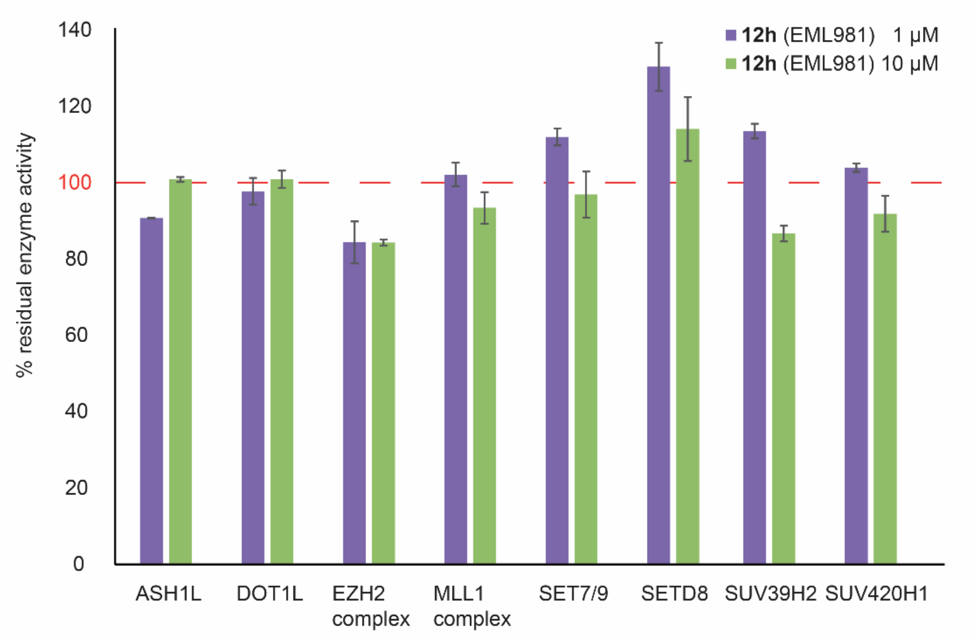

Inhibitory activity profiles of compound 12h towards ASH1L, DOT1L, EZH2, MLL1, SET7/9, SETD8, SUV39H2, and SUV420H1 at two different fixed concentrations (1 and 10 µM).

Table S1. Inhibitory activity values of compound **12h** against a panel of KMTs

	**% enzyme residual activ. after treatment with 12h[a,b,]**		**ref. cmpd. IC_{50}(µM)[b,c]**		
	1 µM	10 µM	SAH	chaet	ryuv
**ASH1L**	90.76±0.13	90.76±0.13	-	0.14	-
**DOT1L**	97.74±3.48	90.76±0.13	0.174	-	-
**EZH2**	84.39±5.51	90.76±0.13	18.8	-	-
**G9a**	101.54±1.67	90.76±0.13	1.19	-	-
**MLL1**	102.15±3.11	90.76±0.13	0.464	-	-
**SET7/9**	112.02±2.25	90.76±0.13	139	-	-
**SETD8**	130.39±6.36	90.76±0.13	-	-	0.186
**SMYD3**	72.83±0.68	90.76±0.13	18.7	-	-
**SUV39H2**	113.51±1.92	90.76±0.13	32.7	-	-
**SUV420H1**	104.00±1.13	90.76±0.13	46.5	-	-

[a]Compounds were tested at 1 and 10 µM fixed concentrations; values obtained for each compound are mean ± SD determined for two separate experiments.in duplicate; [b]values were obtained in a radioisotope-based filter assay, using 0.05 mg/mL oligo nucleosomes (for ASH1L, MLL1 complex, SETD8, SUV420H1 and DOT1L), 0.05 mg/mL core histone (for EZH2 complex and SET7/9), or 5 µM histone H3 (for SUV39H2) as substrate and *S*-adenosyl-L-[methyl-^{3}H]methionine (1 µM) as methyl donor; [c]reference compounds were tested in 10-dose IC_{50} mode with 3-fold serial dilution starting at 100 µM; data were analyzed using Excel and GraphPad Prism 6.0 software (GraphPad Software Inc., San Diego, CA) for IC_{50} curve fits using sigmoidal dose *vs.* response - variable slope (four parameters) equations.

Figure S3.

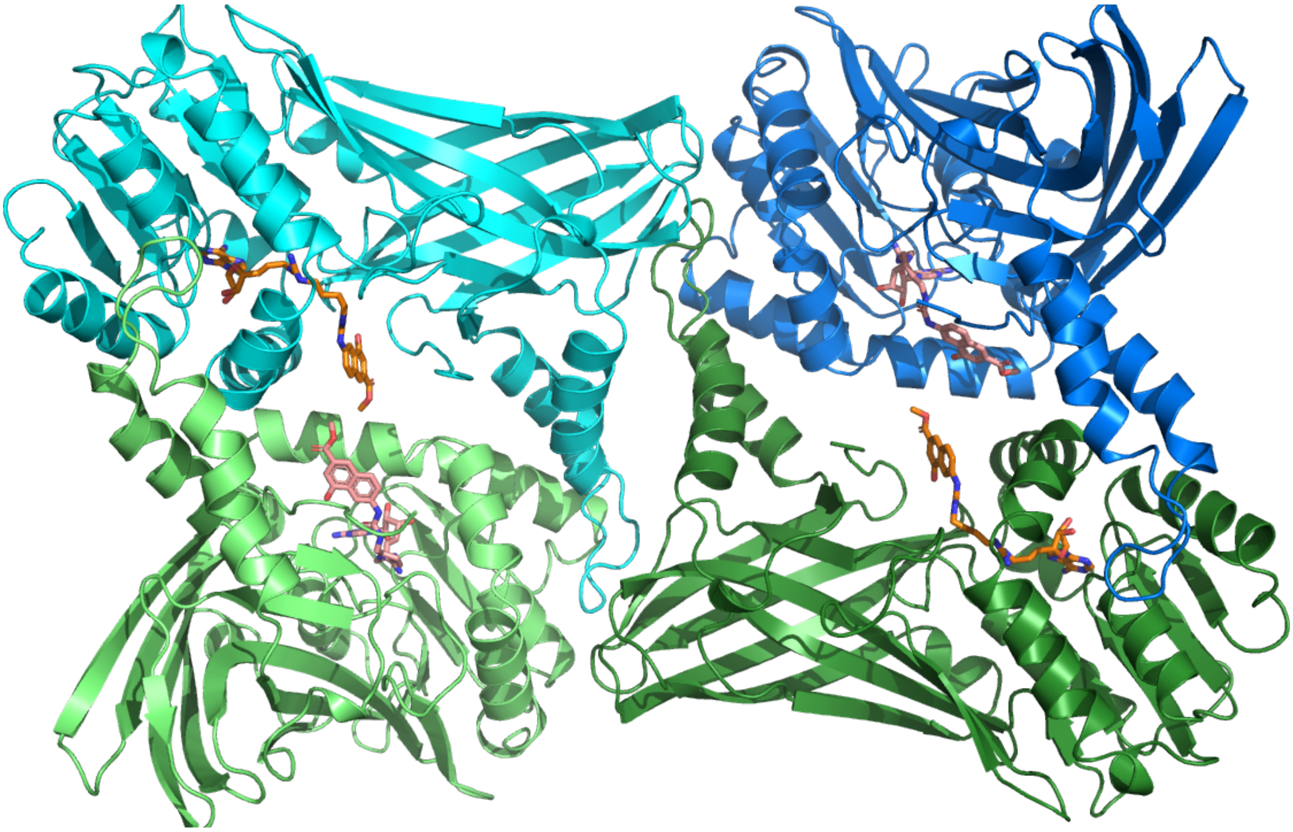

An overview of Tetramer of *mm*PRMT4 with compound **12h** (EML981) bound (PDB ID 7PUC). PRMT4 monomers within tetramer (dodger blue, cyan, light green, sea green) are represented as a cartoon. Compound **12h** (EML981) is represented as orange sticks. PRMT4 crystallized in the space group $P2_12_12$ which contains one copy of the PRMT4 tetramer in the asymmetric unit.

Figure S4.

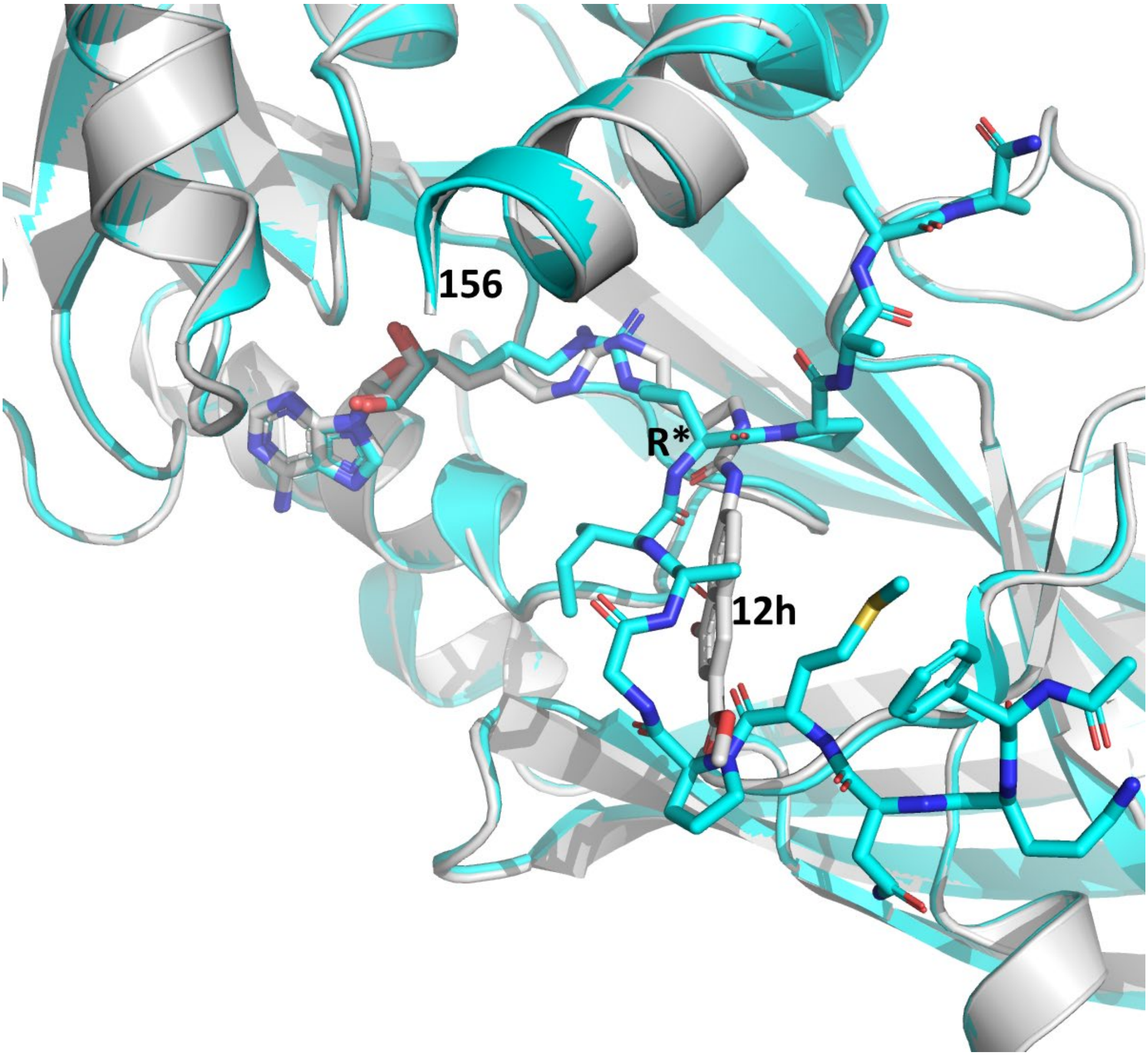

Superimposition of compound **12h** (grey sticks) bound to *mm*PRMT4 (represented as grey cartoon, PDB ID 7PUC) with a transition state mimic (cyan sticks) bound to *mm*PRMT4 (cyan cartoon, PDB ID 5LGP). R* represent the mimic of the arginine to be methylated. Superimposition is done on protein backbones. For clarity, the N-terminal helices of mmCARM1 are not shown.

Figure S5.

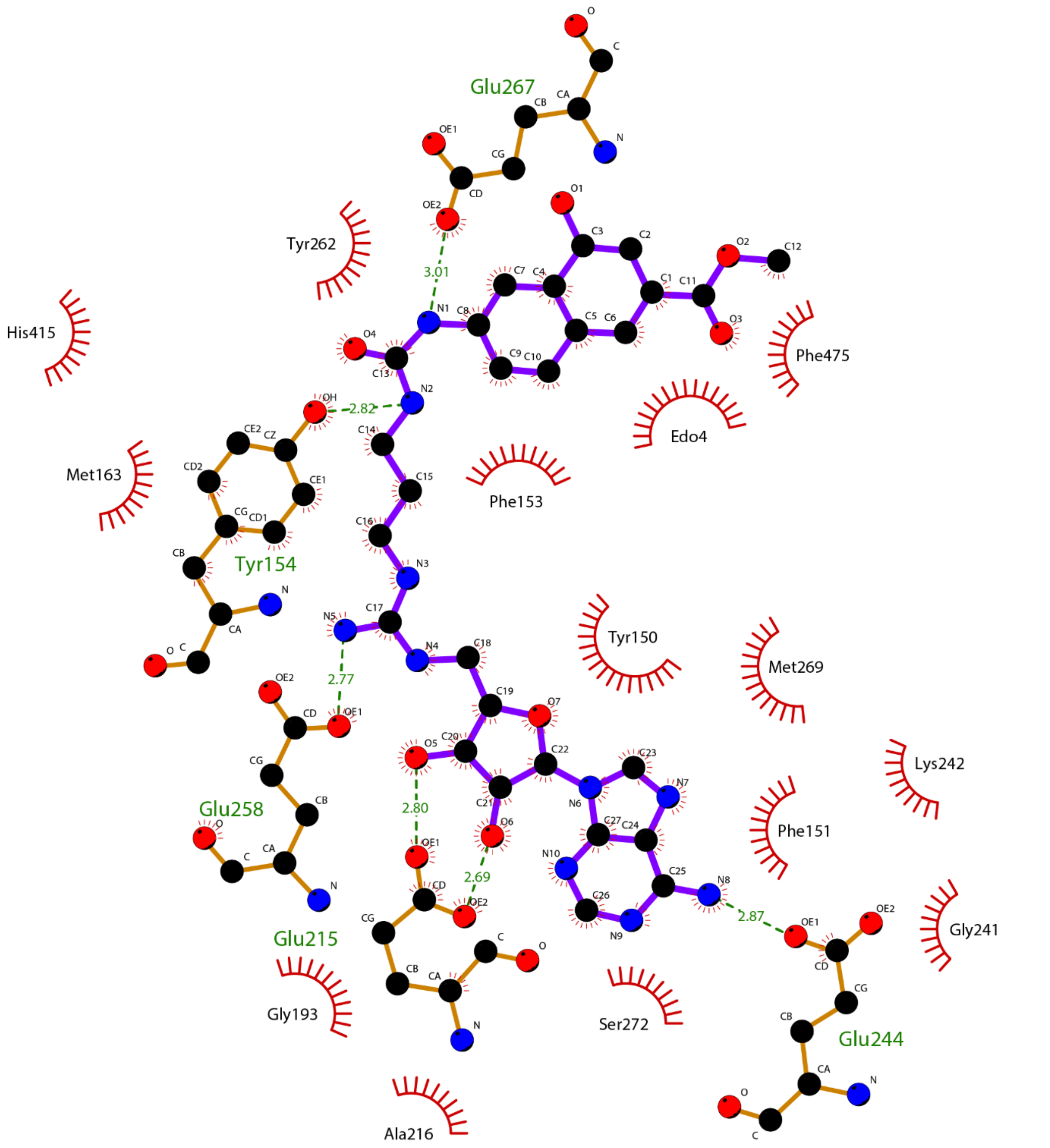

Flat representation done with LigPlot+ showing contacts between compound **12a** (EML734) and active site residues in PRMT4 (subunit B). Hydrogen bonds are shown as dotted lines. The spoked arcs represent protein residues making non-bonded (hydrophobic) contacts with compounds.

Figure S6.

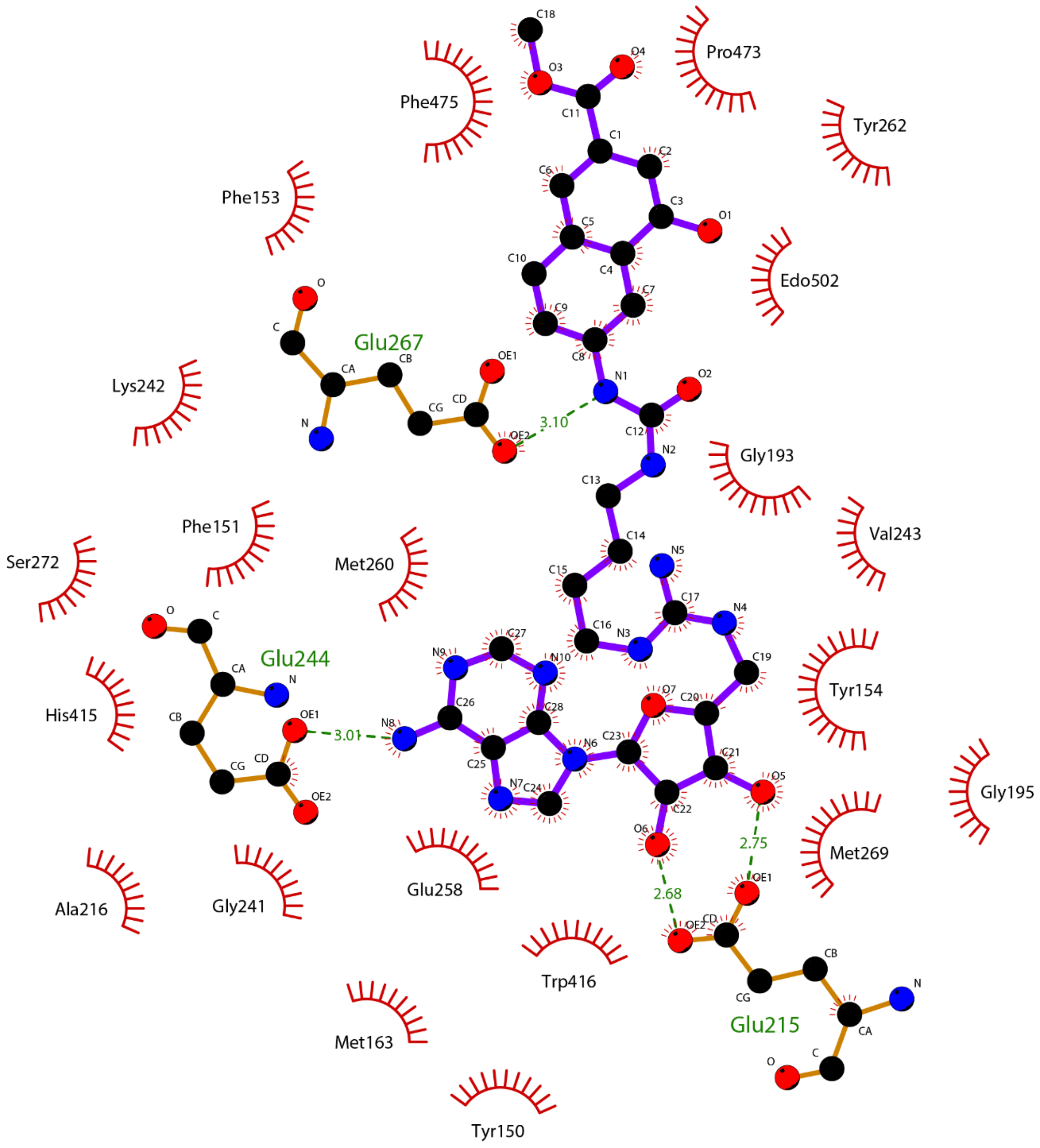

Flat representation done with LigPlot+ showing contacts between compound **12b** (EML709) and active site residues in PRMT4 (subunit B). Hydrogen bonds are shown as dotted lines. The spoked arcs represent protein residues making non-bonded (hydrophobic) contacts with compounds.

Figure S7.

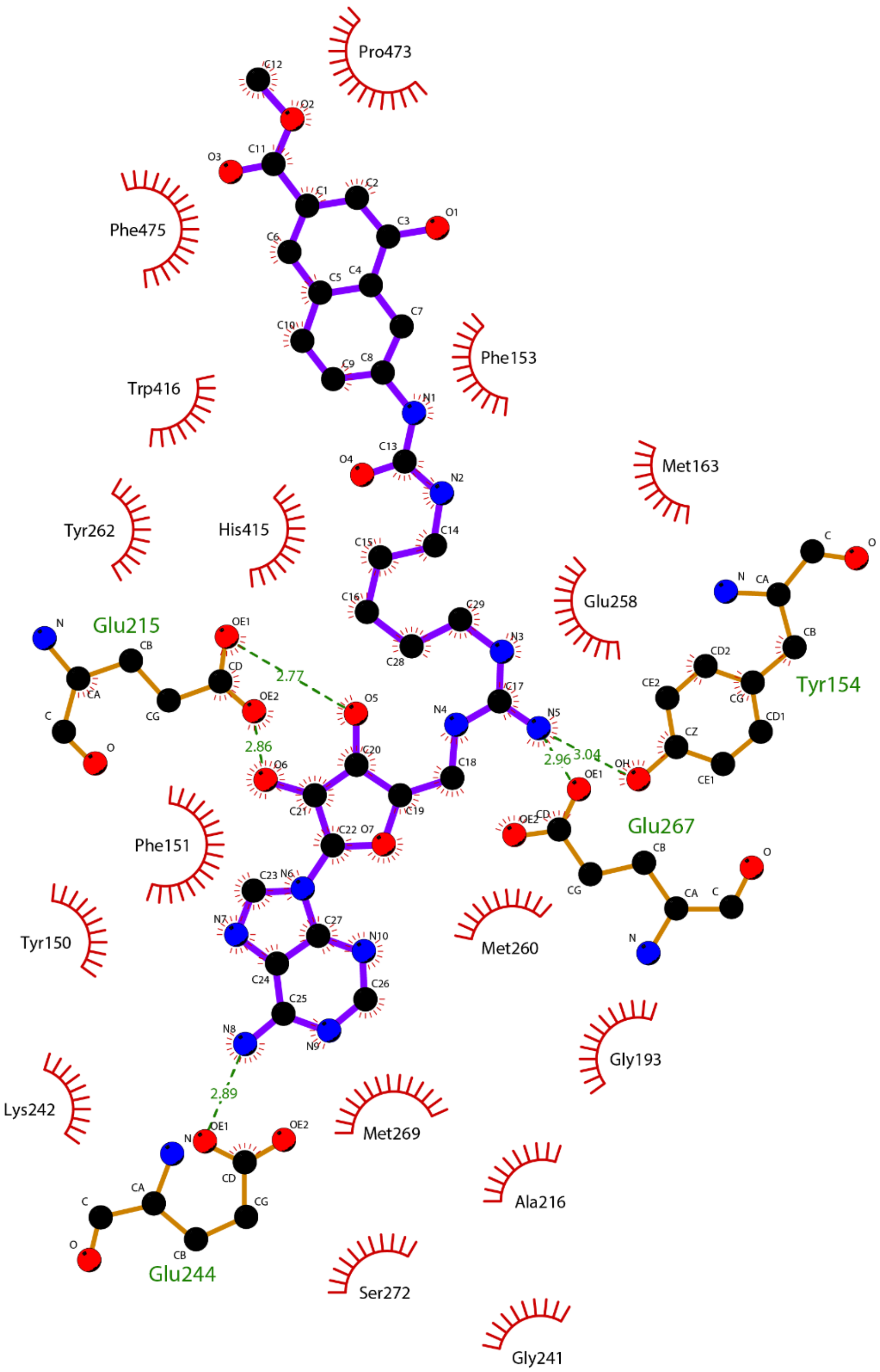

Flat representation done with LigPlot+ showing contacts between compound **12c** (EML736) and active site residues in PRMT4 (subunit B). Hydrogen bonds are shown as dotted lines. The spoked arcs represent protein residues making non-bonded (hydrophobic) contacts with compounds.

Figure S8.

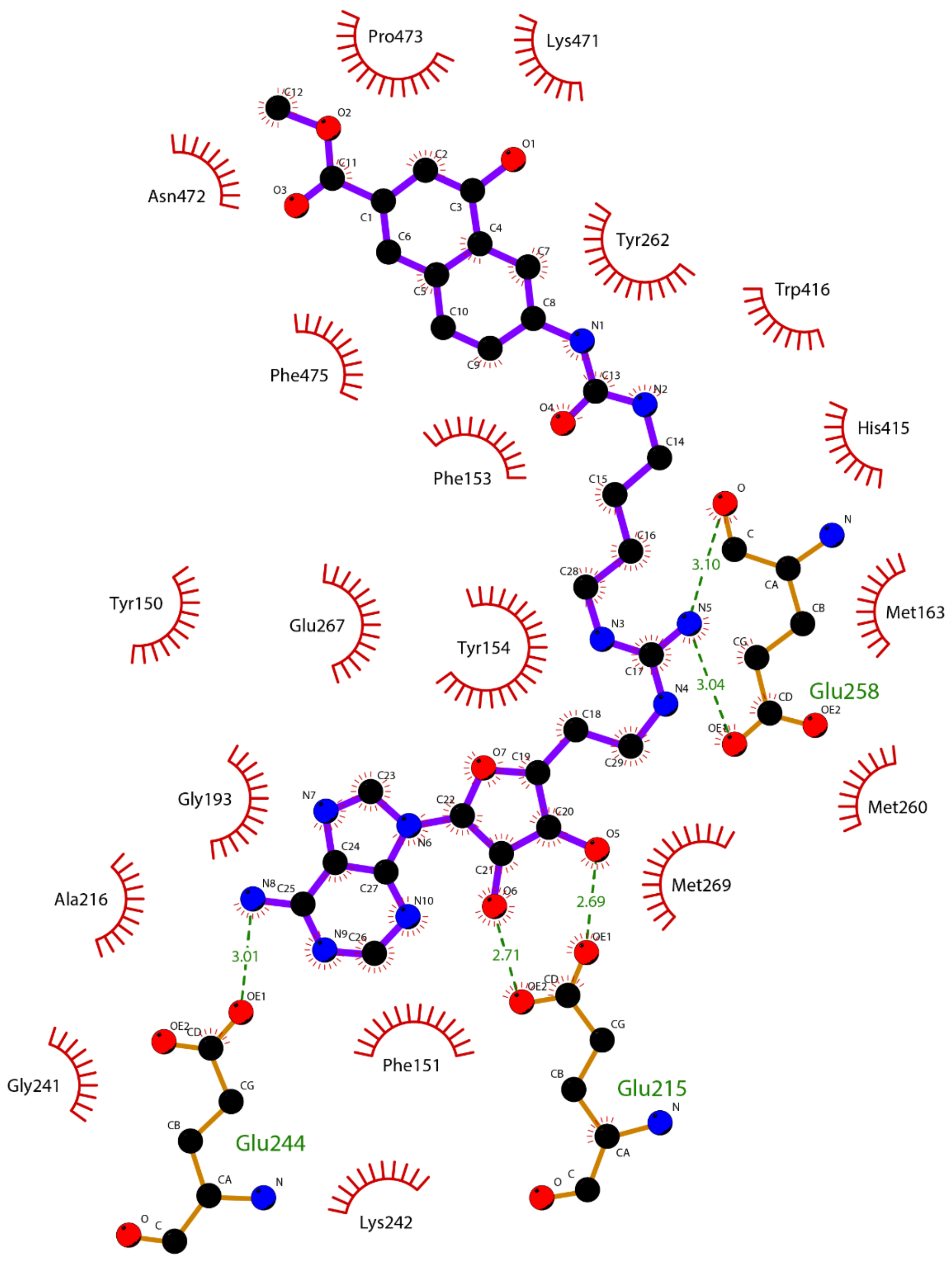

Flat representation done with LigPlot+ showing contacts between compound **12f** (EML980) and active site residues in PRMT4 (subunit B). Hydrogen bonds are shown as dotted lines. The spoked arcs represent protein residues making non-bonded (hydrophobic) contacts with compounds.

Figure S9.

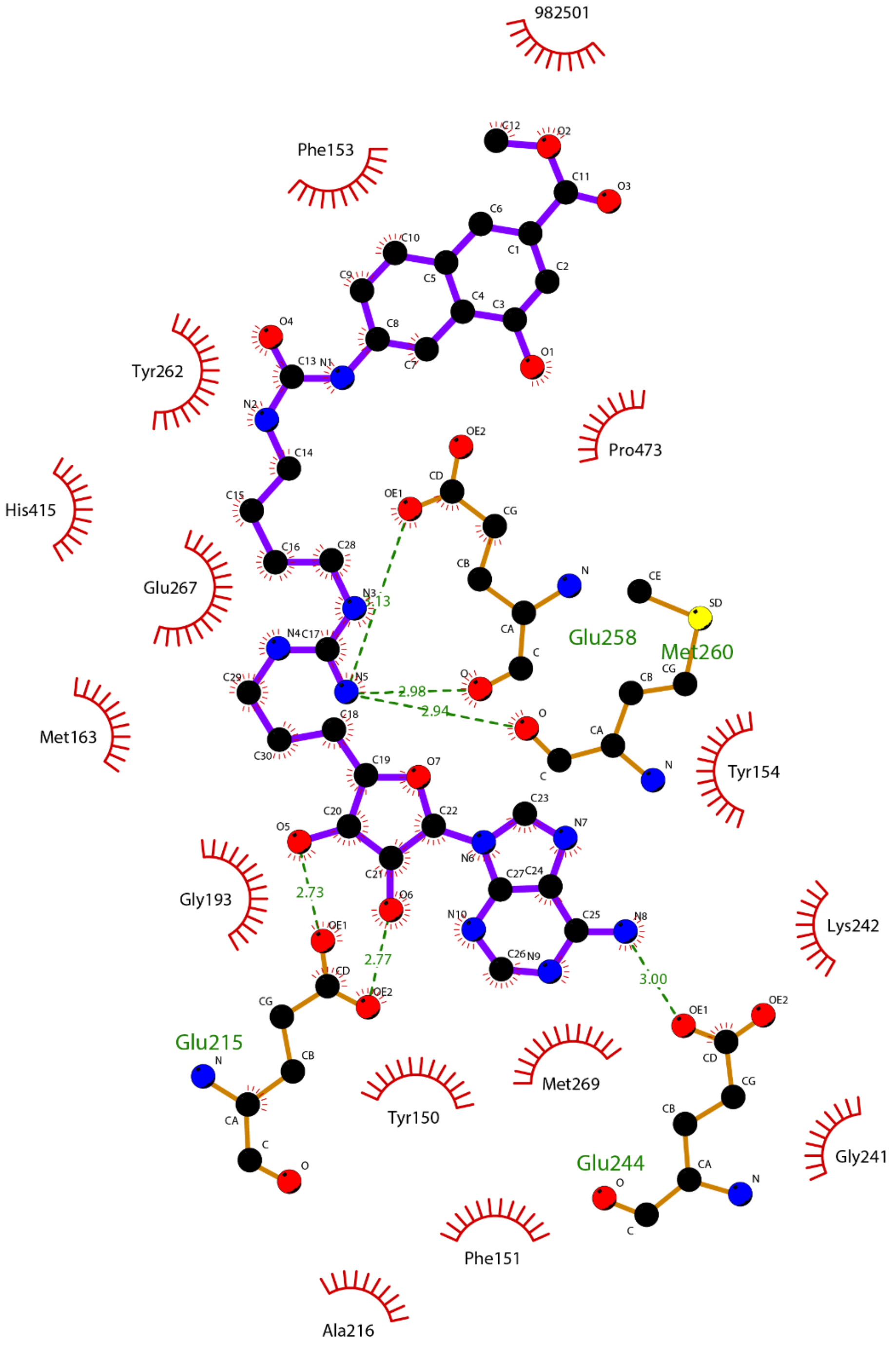

Flat representation done with LigPlot+ showing contacts between compound **12g** (EML982) and active site residues in PRMT4 (subunit B). Hydrogen bonds are shown as dotted lines. The spoked arcs represent protein residues making non-bonded (hydrophobic) contacts with compounds.

Figure S10.

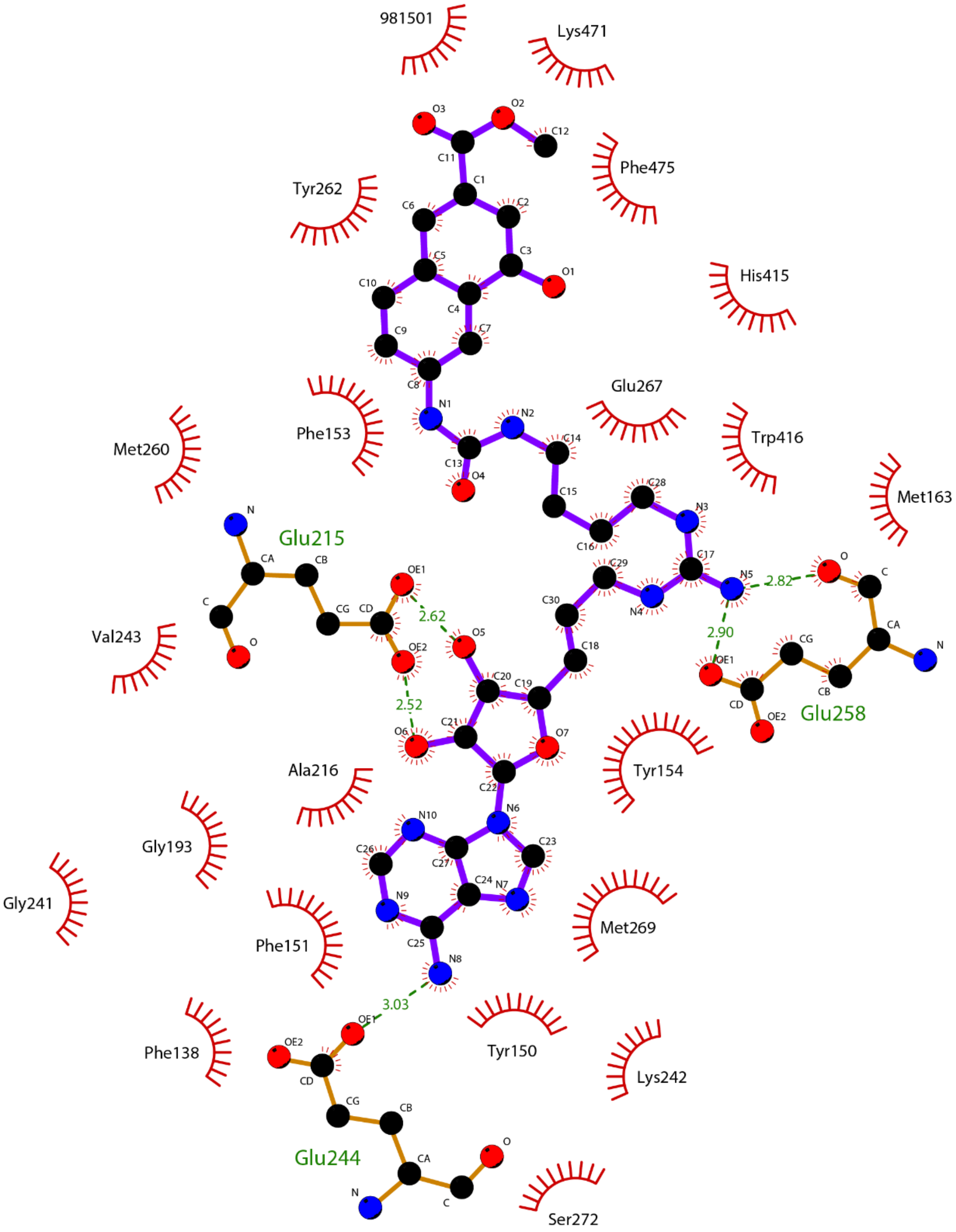

Flat representation done with LigPlot+ showing contacts between compound **12h** (EML981) and active site residues in PRMT4 (subunit B). Hydrogen bonds are shown as dotted lines. The spoked arcs represent protein residues making non-bonded (hydrophobic) contacts with compounds.

Figure S11.

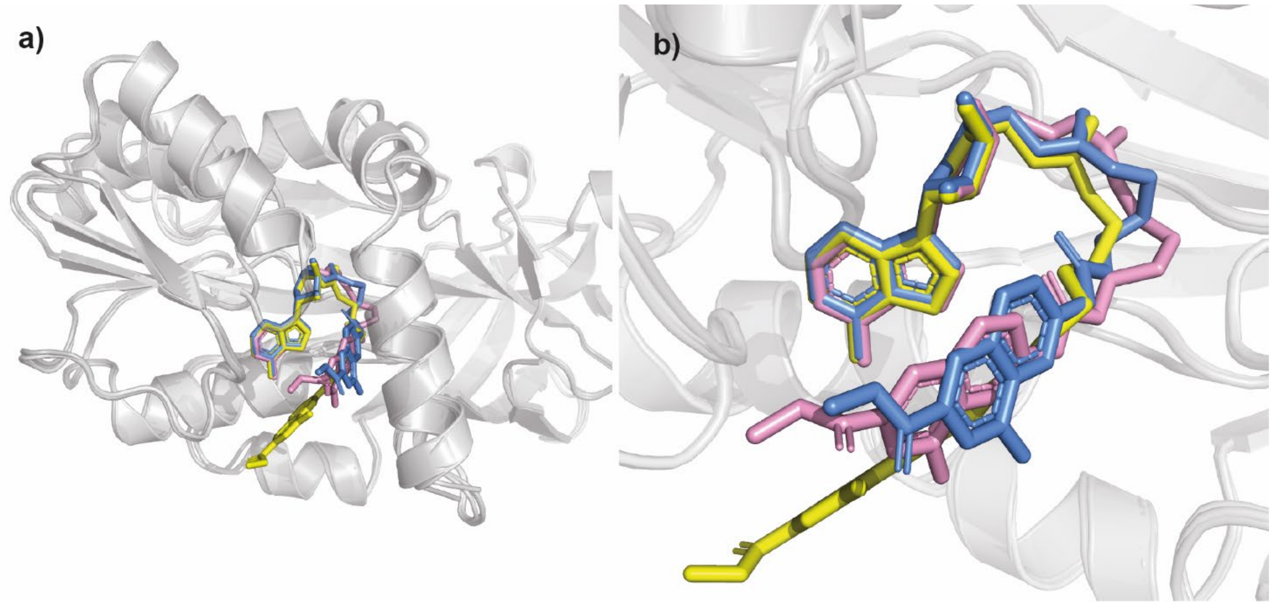

Structures of the complexes of PRMT6 and compounds **12a**, **12c**, and **12f** (PDB ID 7NUD, 7NUE, 7P2R, respectively). (a) Superimposition of compound **12a** (cornflower blue sticks) bound to subunit A of *mm*PRMT6, compound **12c** (yellow sticks) bound to subunit A of *mm*PRMT6, compound **12f** (pink sticks) bound to subunit A of *mm*PRMT6. Superimposition is done on protein backbones. Protein subunits are shown as cartoons colored in different shades of grey; (b) close-up view of bound compound conformations. For clarity, the N-terminal helices of *mm*PRMT6 (residues 42–56) are not shown.

Figure S12.

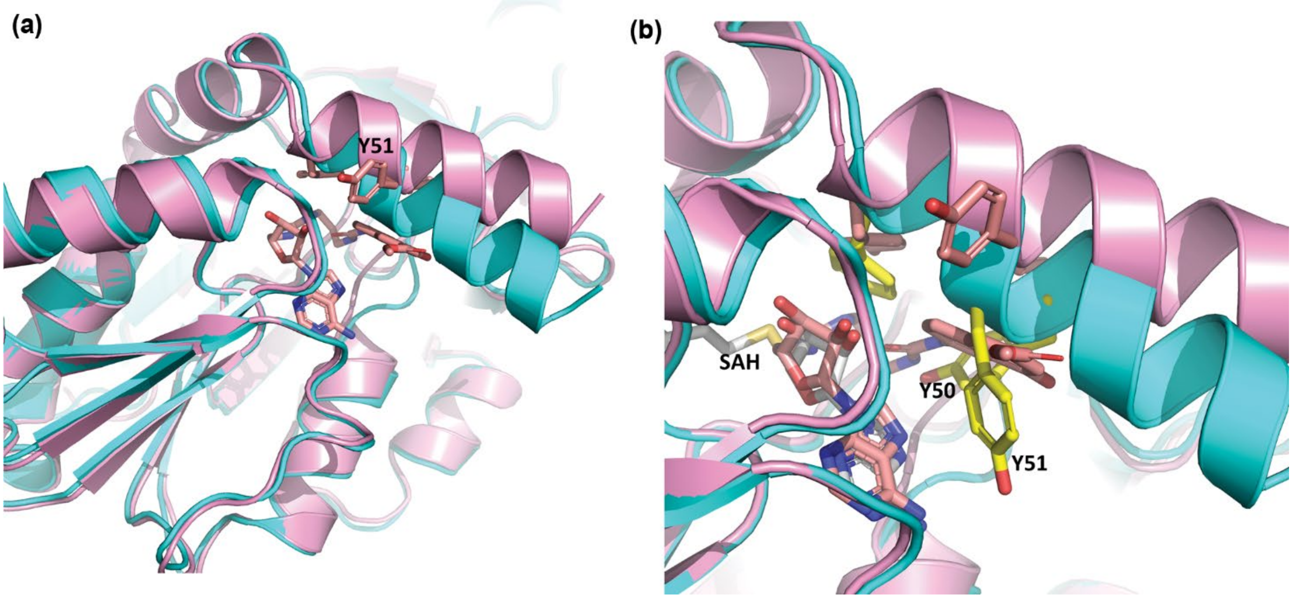

Conformational changes of PRMT6 alpha-X helix containing the motif I (Y50-Y51-X-X-Y54) (a) Superimposition of the structures of compound **12a** (EML734) bound to PRMT6 (pink cartoon, PDB ID 7NUD) and of the PRMT6/SAH complex (PDB ID 5FQN, cyan cartoon). Y51 of motif I and **12a** are shown as sticks. (B) Close-up view displaying also Y50, Y51 (yellow sticks) and SAH molecule (grey sticks) as seen in PRMT6/SAH structure.

Table S2. Permeability values of compounds **12f–h**

#	PAMPA P_{app} (cm/s)
**12f**	0.55×10^{-7}
**12g**	0.39×10^{-7}
**12h**	0.38×10^{-7}
**propranolol**	60×10^{-7}
**furosemide**	0.34×10^{-7}

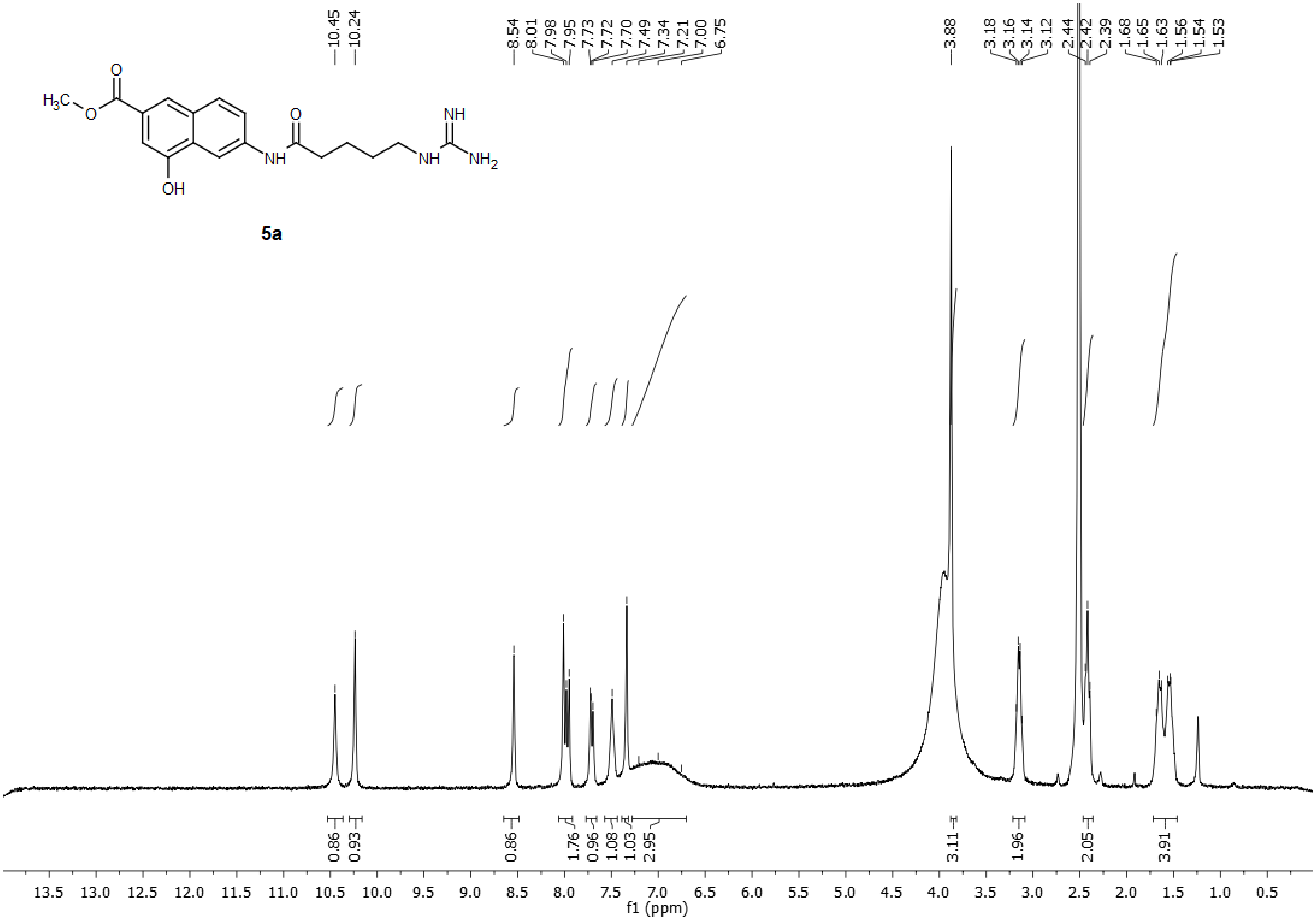

5a
10.45
10.24
8.54
8.01
7.98
7.95
7.73
7.72
7.70
7.49
7.34
7.21
7.00
6.75
3.88
3.18
3.16
3.14
3.12
2.44
2.42
2.39
1.68
1.65
1.63
1.56
1.54
1.53
0.86
0.93
0.86
1.76
0.96
1.08
1.03
2.95
3.11
1.96
2.05
3.91
f1 (ppm)

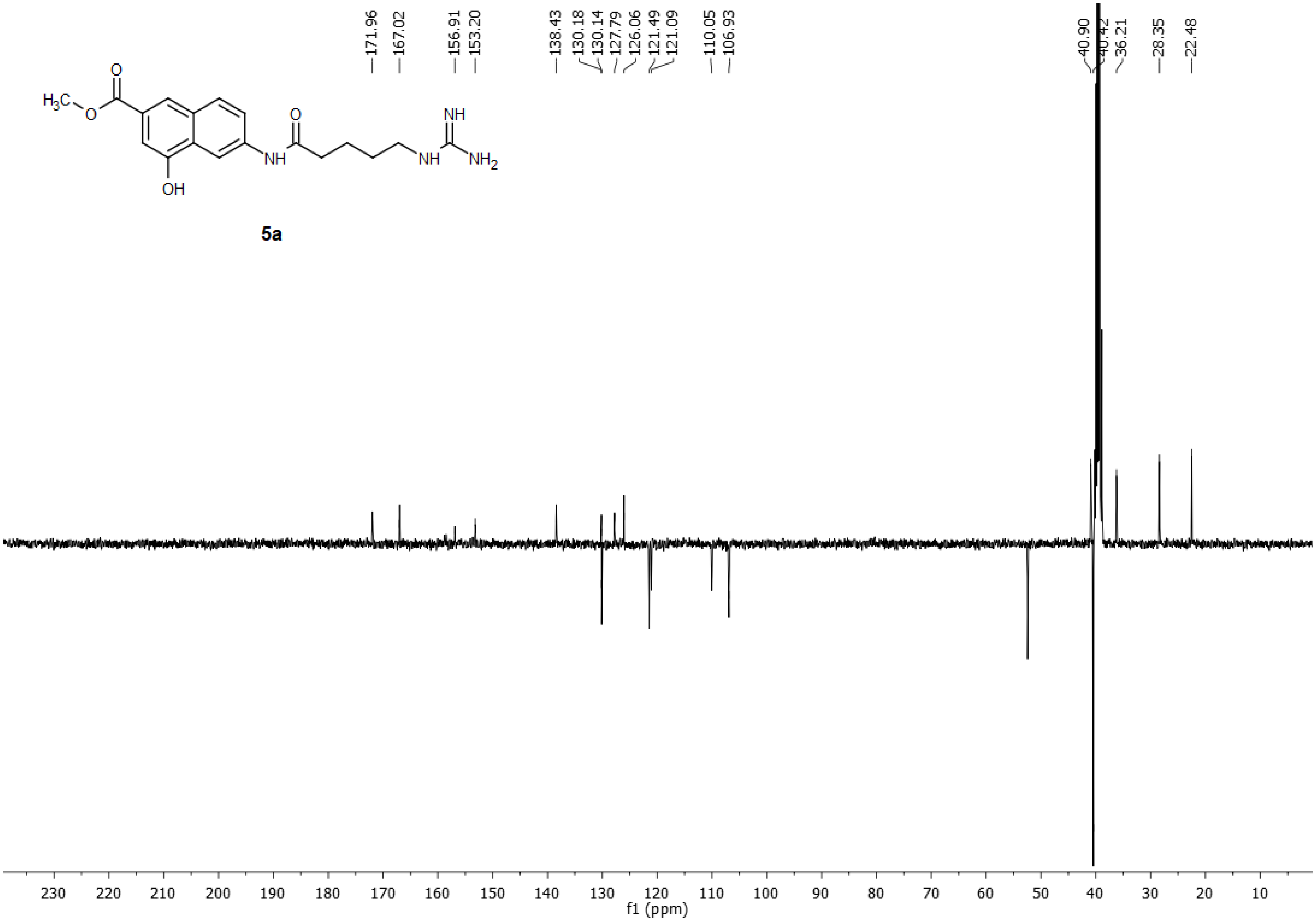
171.96
167.02
156.91
153.20
138.43
130.18
130.14
127.79
126.06
121.49
121.09
110.05
106.93
40.90
40.42
36.21
28.35
22.48
H3C
O
O
OH
NH
O
NH
NH
NH2
5a
f1 (ppm)

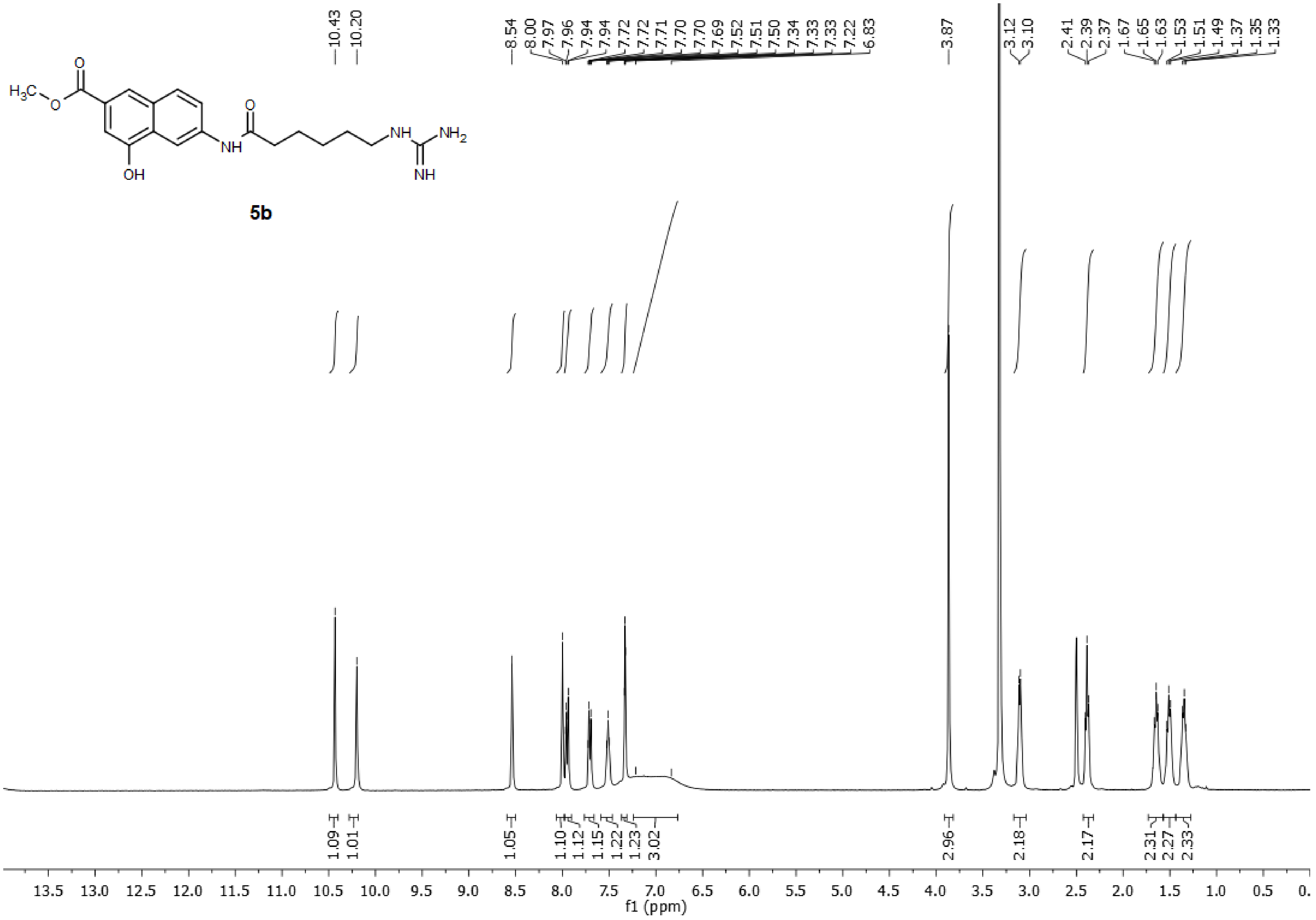
5b
f1 (ppm)

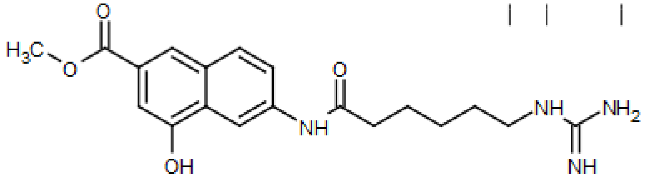

5b

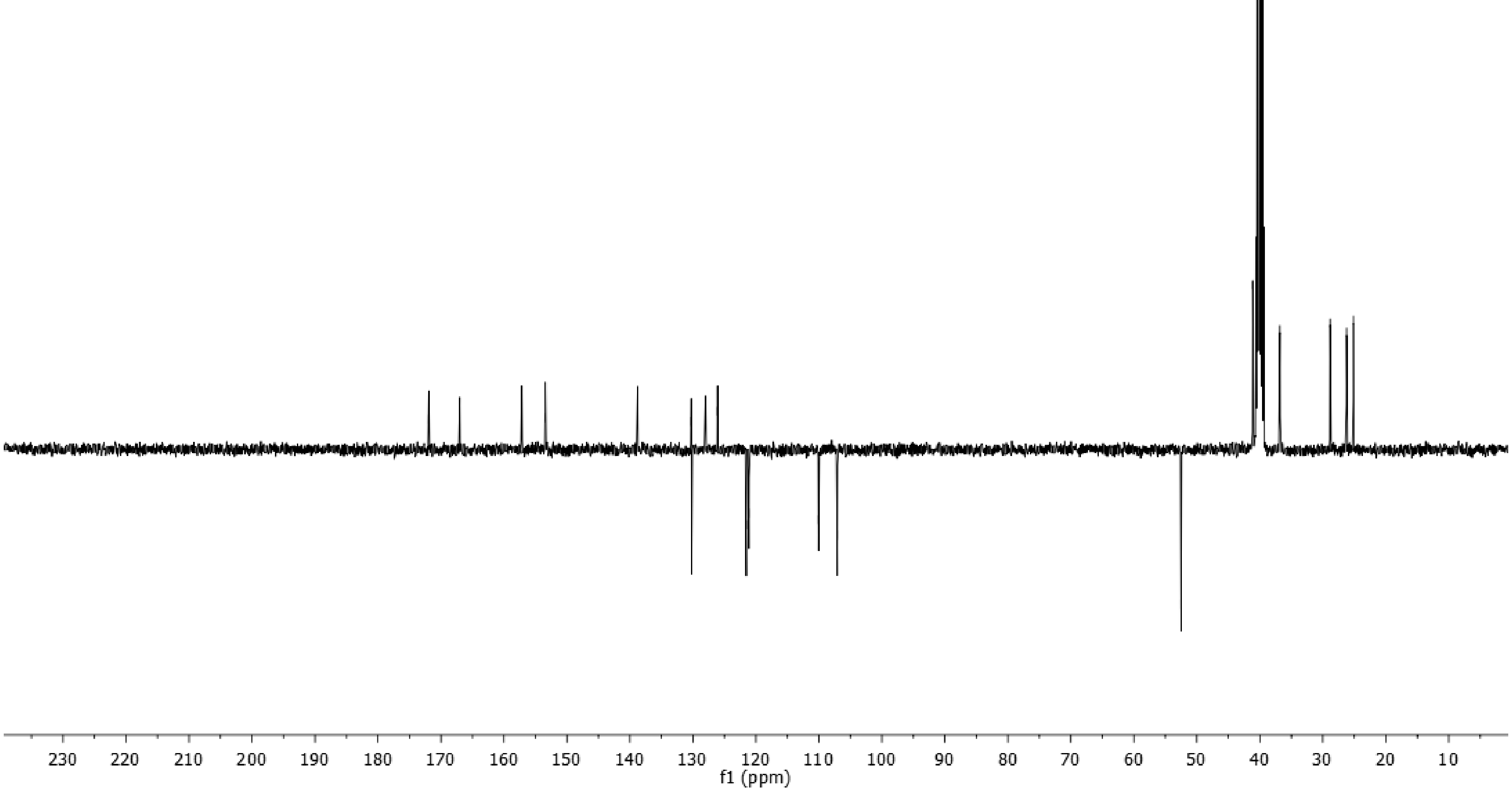
171.94
167.05
157.18
153.45
138.77
130.27
130.19
128.00
126.10
121.55
121.13
110.05
107.10
52.51
41.13
36.82
28.84
26.23
25.13
230 220 210 200 190 180 170 160 150 140 130 120 110 100 90 80 70 60 50 40 30 20 10
f1 (ppm)

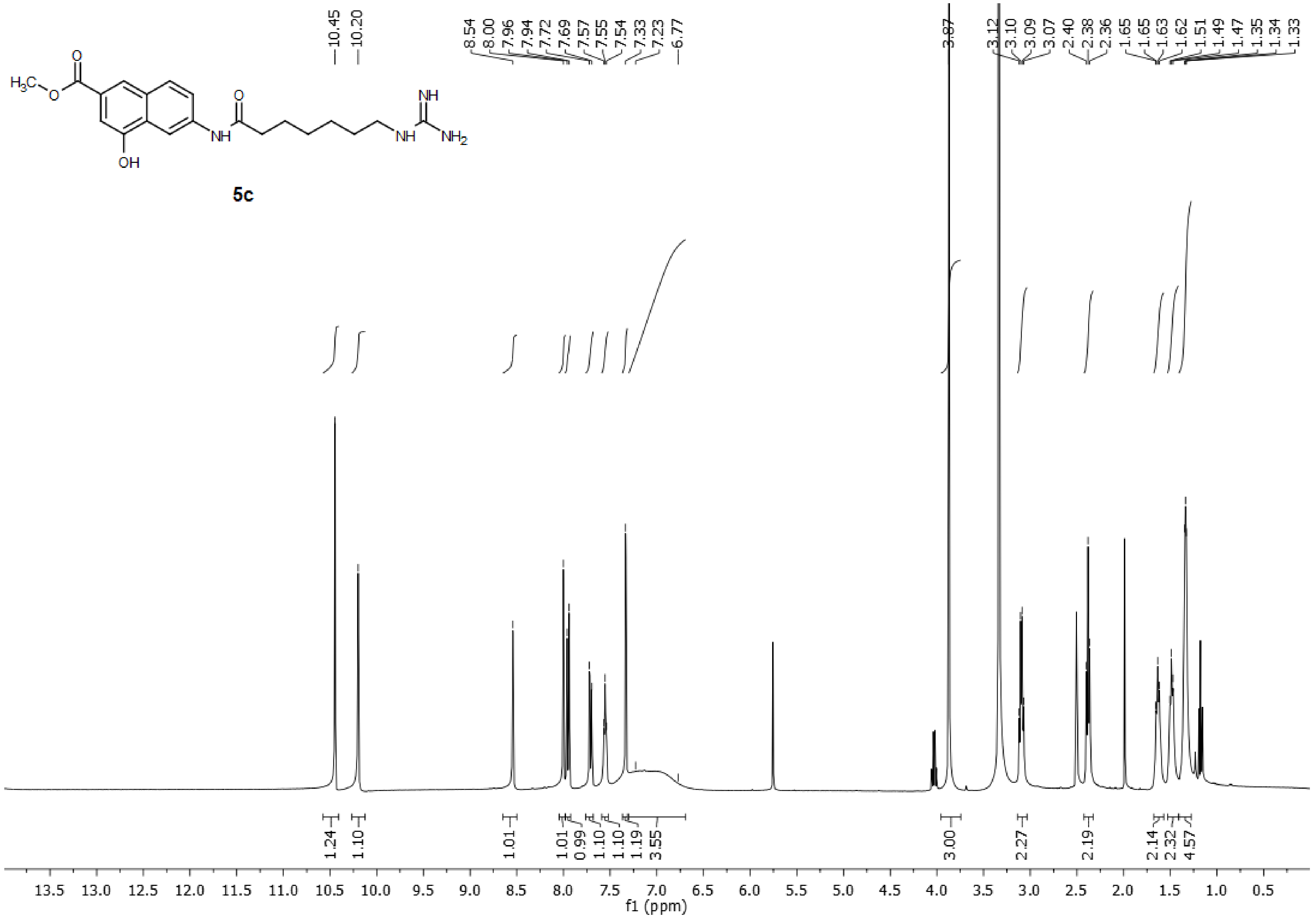

H3C
O
O
OH
NH
O
NH
NH
NH2
5c
10.45
10.20
8.54
8.00
7.96
7.94
7.72
7.69
7.57
7.55
7.54
7.33
7.23
6.77
3.87
3.12
3.10
3.09
3.07
2.40
2.38
2.36
1.65
1.65
1.63
1.62
1.51
1.49
1.47
1.35
1.34
1.33
1.24
1.10
1.01
1.01
0.99
1.10
1.10
1.19
3.55
3.00
2.27
2.19
2.14
2.32
4.57
f1 (ppm)

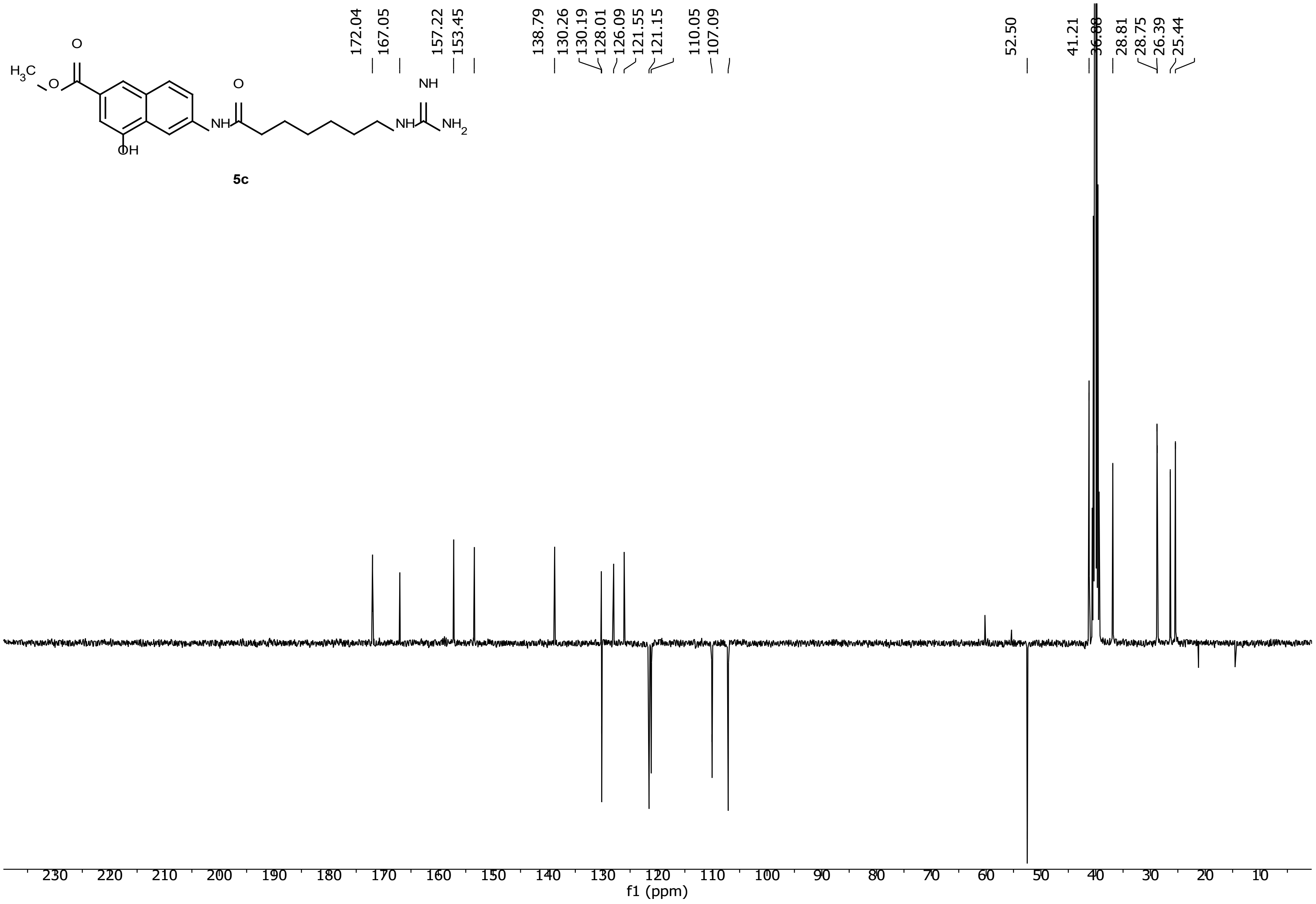
172.04
167.05
157.22
153.45
138.79
130.26
130.19
128.01
126.09
121.55
121.15
110.05
107.09
52.50
41.21
28.81
28.75
26.39
25.44
f1 (ppm)

5c

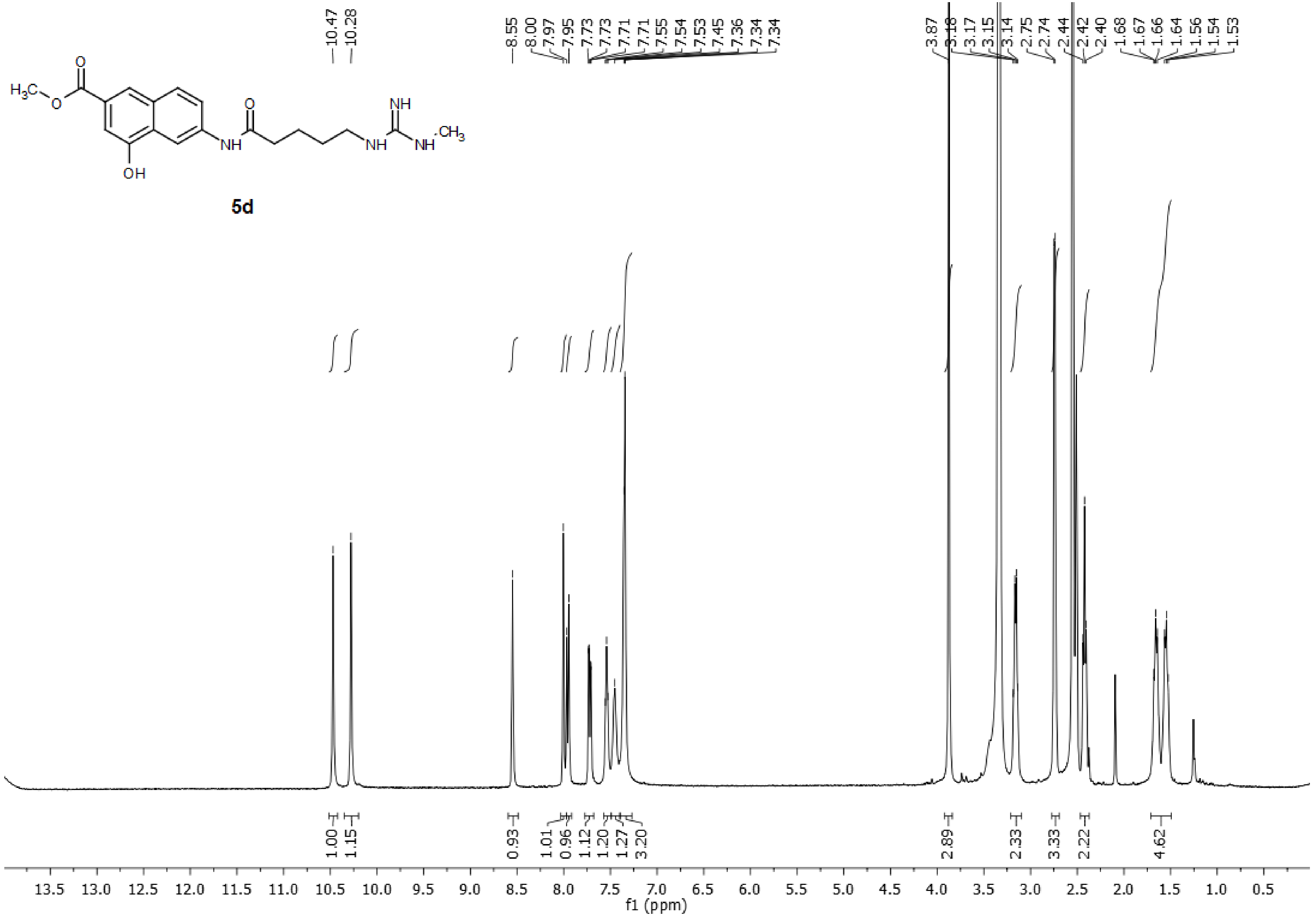

5d
10.47
10.28
8.55
8.00
7.97
7.95
7.73
7.73
7.71
7.71
7.55
7.54
7.53
7.45
7.36
7.34
7.34
3.87
3.18
3.17
3.15
3.14
2.75
2.74
2.44
2.42
2.40
1.68
1.67
1.66
1.64
1.56
1.54
1.53
1.00
1.15
0.93
1.01
0.96
1.12
1.20
1.27
3.20
2.89
2.33
3.33
2.22
4.62
13.5 13.0 12.5 12.0 11.5 11.0 10.5 10.0 9.5 9.0 8.5 8.0 7.5 7.0 6.5 6.0 5.5 5.0 4.5 4.0 3.5 3.0 2.5 2.0 1.5 1.0 0.5
f1 (ppm)

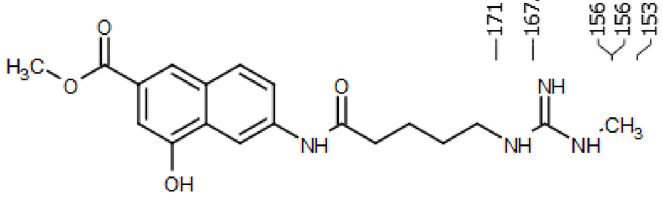
H3C
O
O
NH
O
NH
NH
NH
CH3
OH

5d

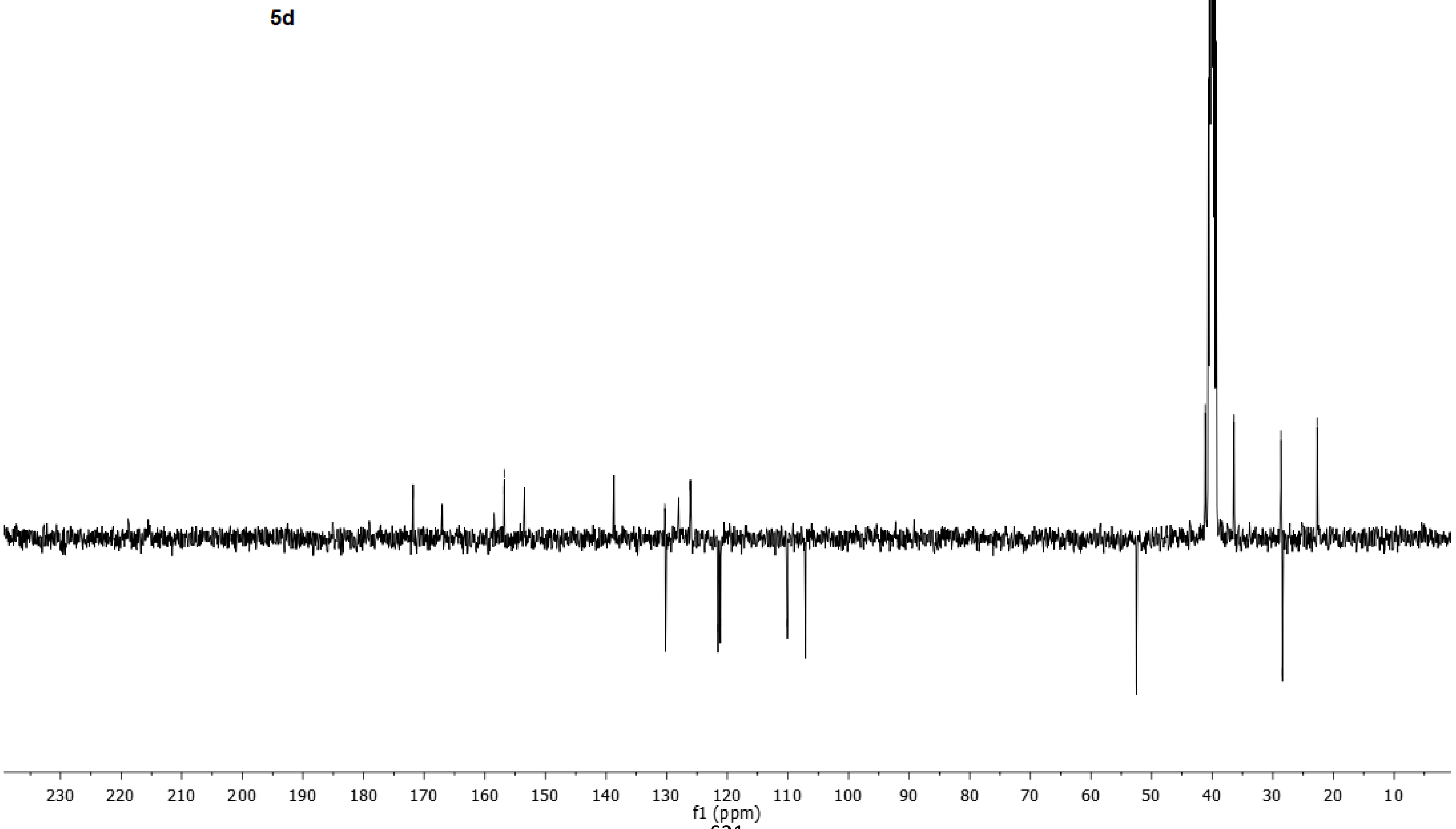
171.85
167.09
156.80
156.79
153.47
138.73
130.29
130.20
128.01
126.12
121.53
121.15
110.10
107.11
52.51
41.13
36.42
28.64
28.39
22.67
230
220
210
200
190
180
170
160
150
140
130
120
110
100
90
80
70
60
50
40
30
20
10
f1 (ppm)

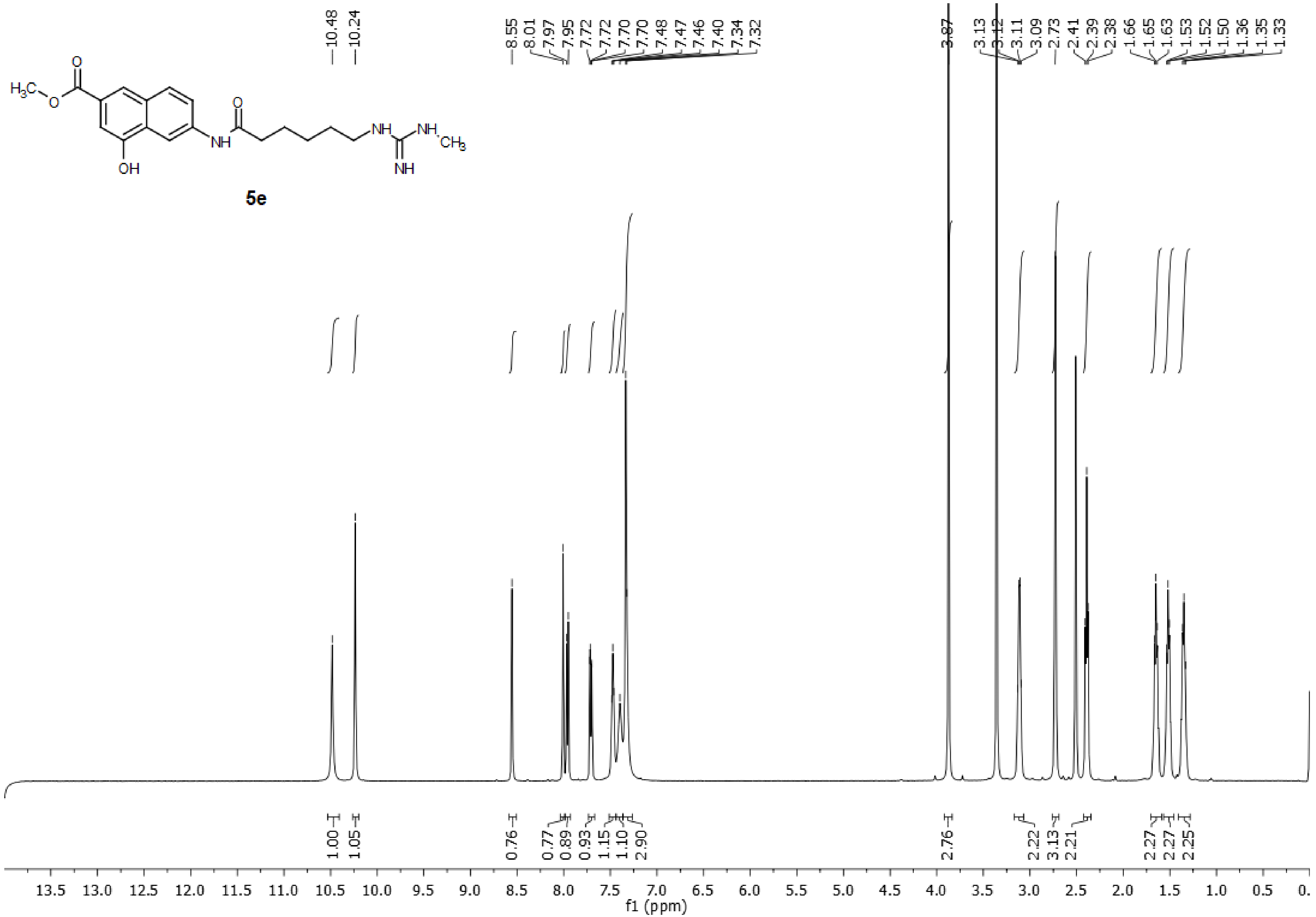

H3C
O
O
NH
O
NH
NH
CH3
NH
OH
5e
10.48
10.24
8.55
8.01
7.97
7.95
7.72
7.72
7.70
7.70
7.48
7.47
7.46
7.40
7.34
7.32
3.87
3.13
3.12
3.11
3.09
2.73
2.41
2.39
2.38
1.66
1.65
1.63
1.53
1.52
1.50
1.36
1.35
1.33
1.00
1.05
0.76
0.77
0.89
0.93
1.15
1.10
2.90
2.76
2.22
3.13
2.21
2.27
2.27
2.25
13.5 13.0 12.5 12.0 11.5 11.0 10.5 10.0 9.5 9.0 8.5 8.0 7.5 7.0 6.5 6.0 5.5 5.0 4.5 4.0 3.5 3.0 2.5 2.0 1.5 1.0 0.5 0.
f1 (ppm)

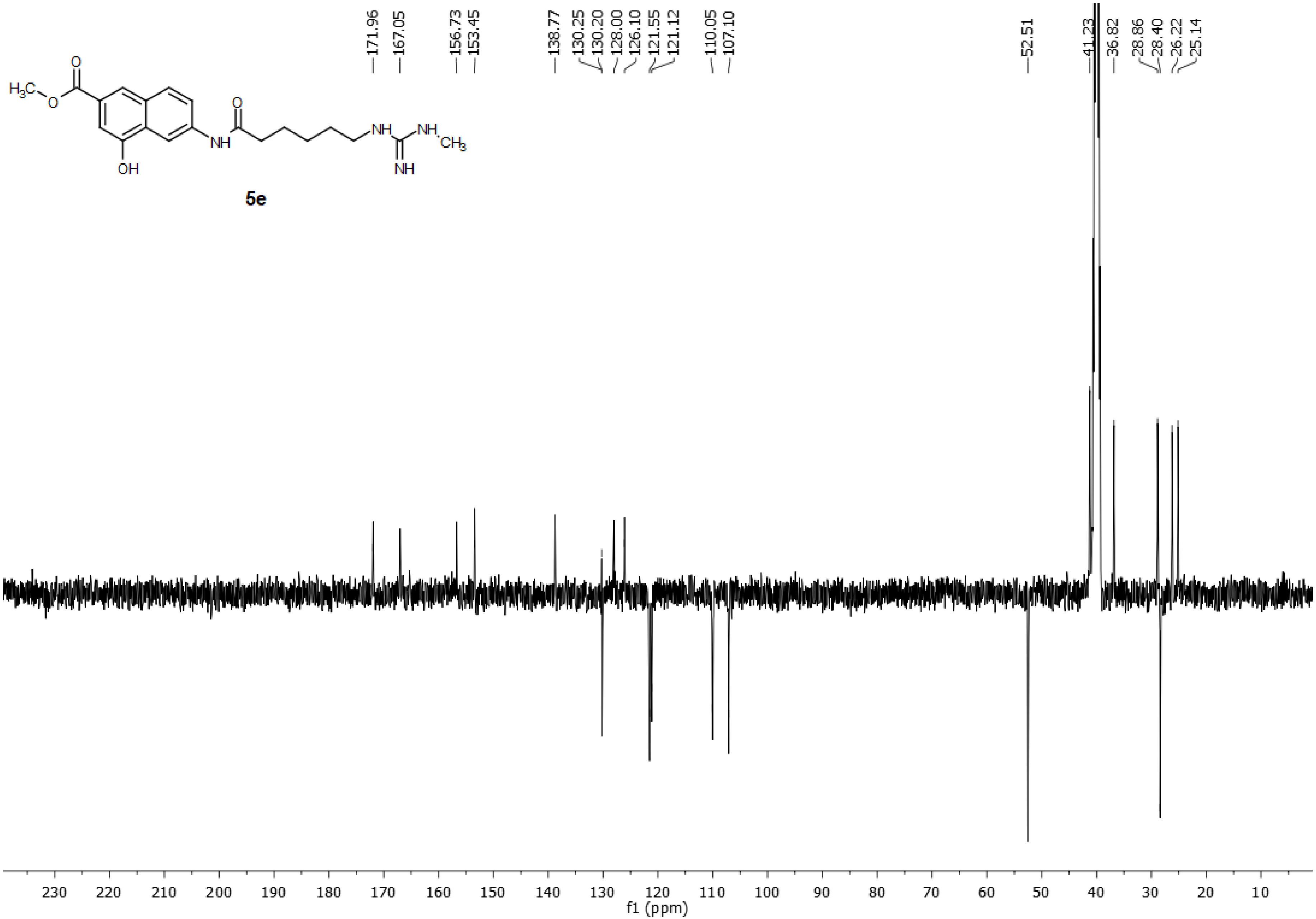
171.96
167.05
156.73
153.45
138.77
130.25
130.20
128.00
126.10
121.55
121.12
110.05
107.10
52.51
41.23
36.82
28.86
28.40
26.22
25.14
H3C
O
O
NH
O
OH
NH
NH
CH3
NH
5e
f1 (ppm)

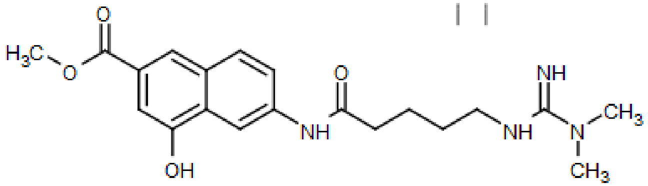

5f

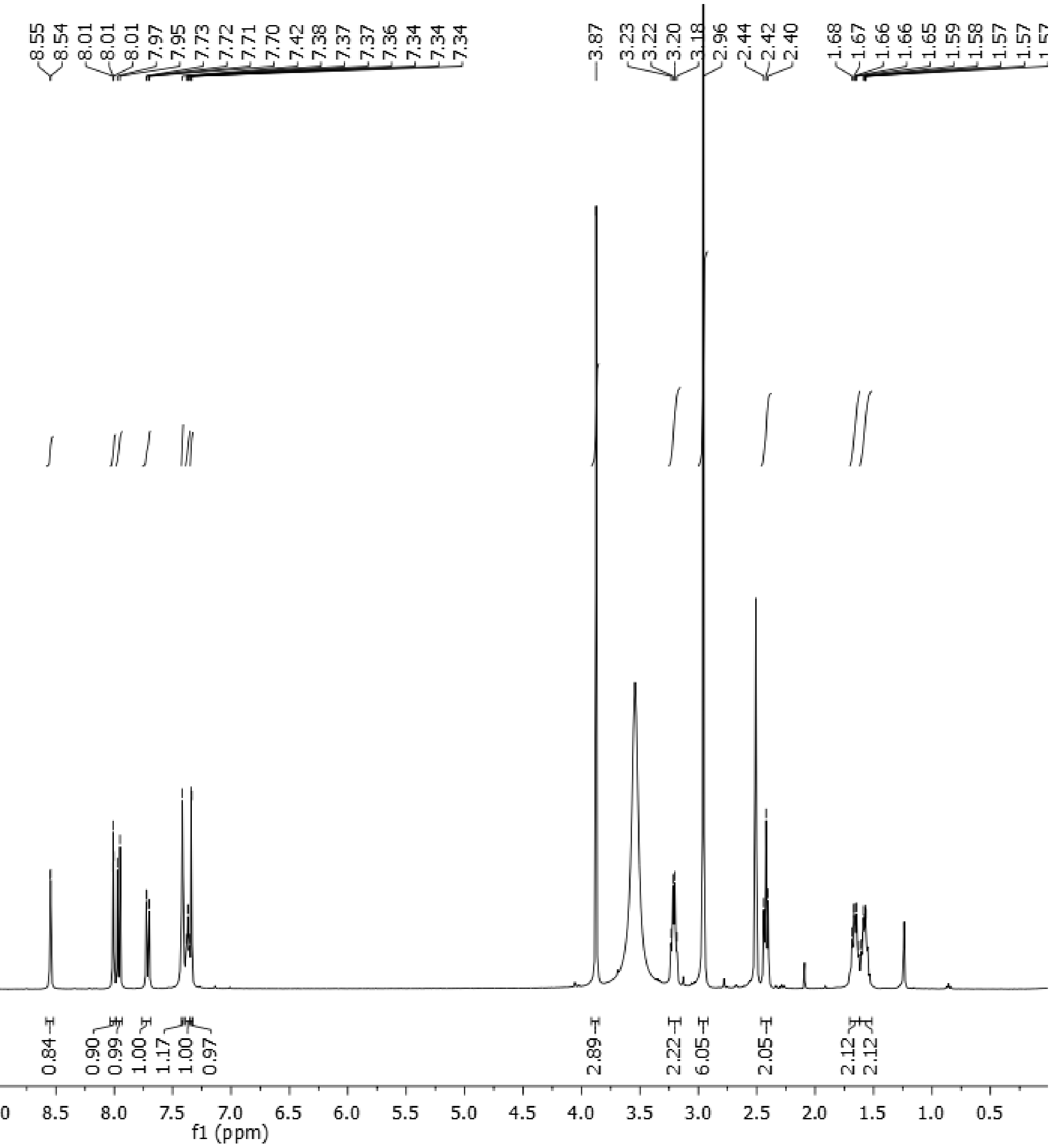

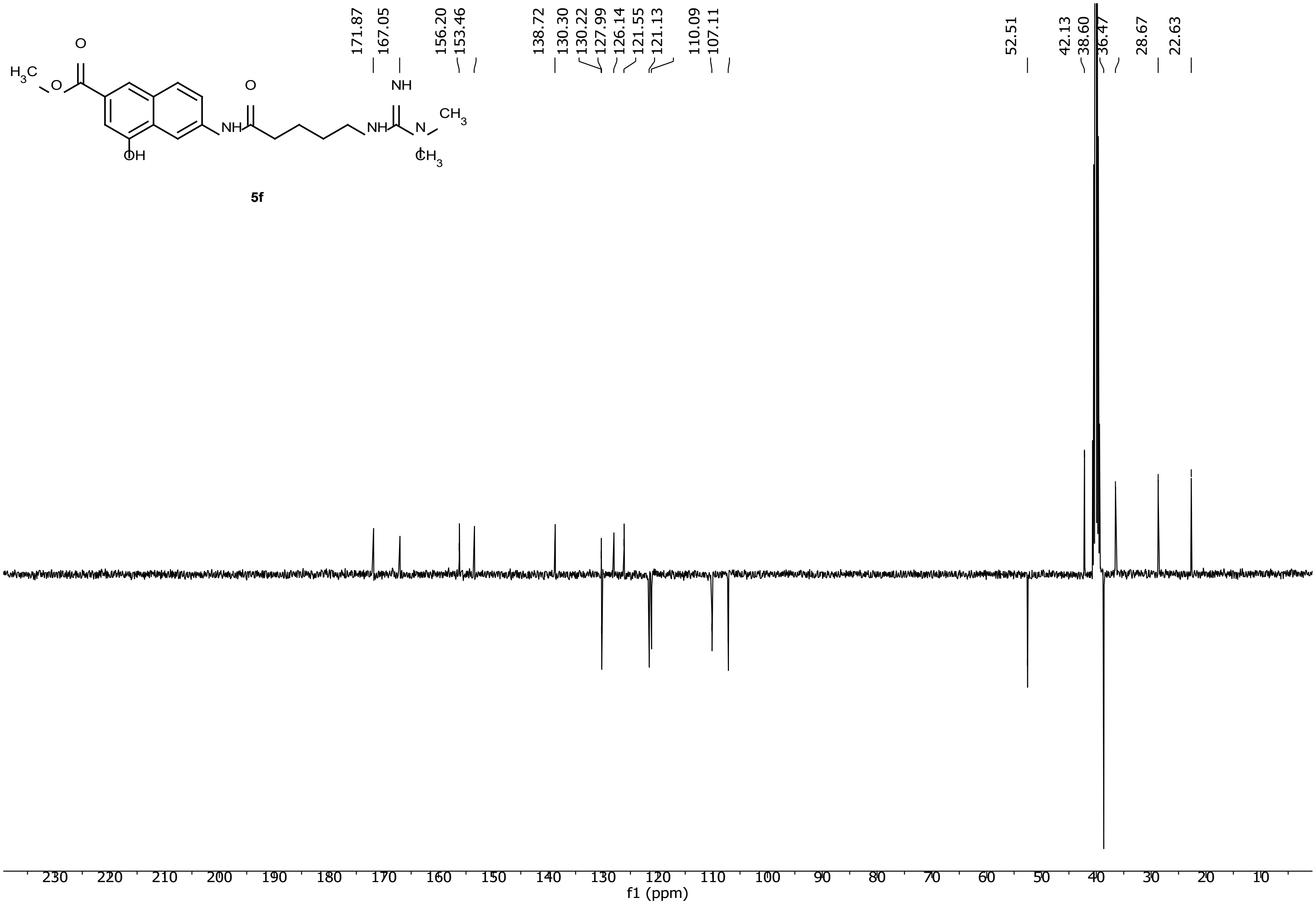
171.87
167.05
156.20
153.46
138.72
130.30
130.22
127.99
126.14
121.55
121.13
110.09
107.11
52.51
42.13
38.60
36.47
28.67
22.63
5f
f1 (ppm)

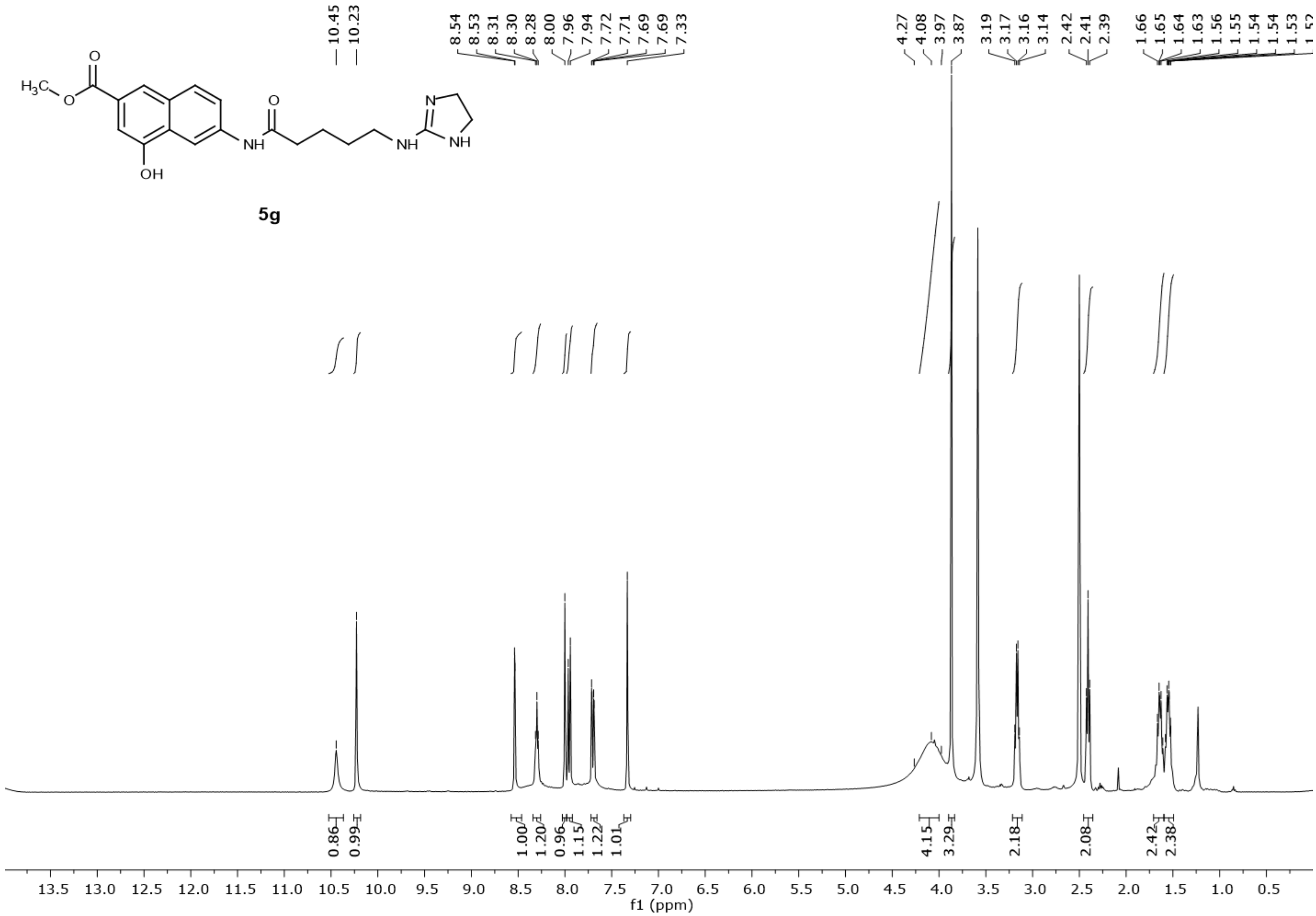

5g
10.45
10.23
8.54
8.53
8.31
8.30
8.28
8.00
7.96
7.94
7.72
7.71
7.69
7.69
7.33
4.27
4.08
3.97
3.87
3.19
3.17
3.16
3.14
2.42
2.41
2.39
1.66
1.65
1.64
1.63
1.56
1.55
1.54
1.54
1.53
0.86
0.99
1.00
1.20
0.96
1.15
1.22
1.01
4.15
3.29
2.18
2.08
2.42
2.38
13.5 13.0 12.5 12.0 11.5 11.0 10.5 10.0 9.5 9.0 8.5 8.0 7.5 7.0 6.5 6.0 5.5 5.0 4.5 4.0 3.5 3.0 2.5 2.0 1.5 1.0 0.5
f1 (ppm)

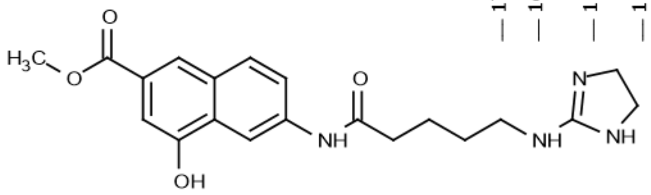

O
H_3C
O
O
N
NH
NH
NH
OH

5g

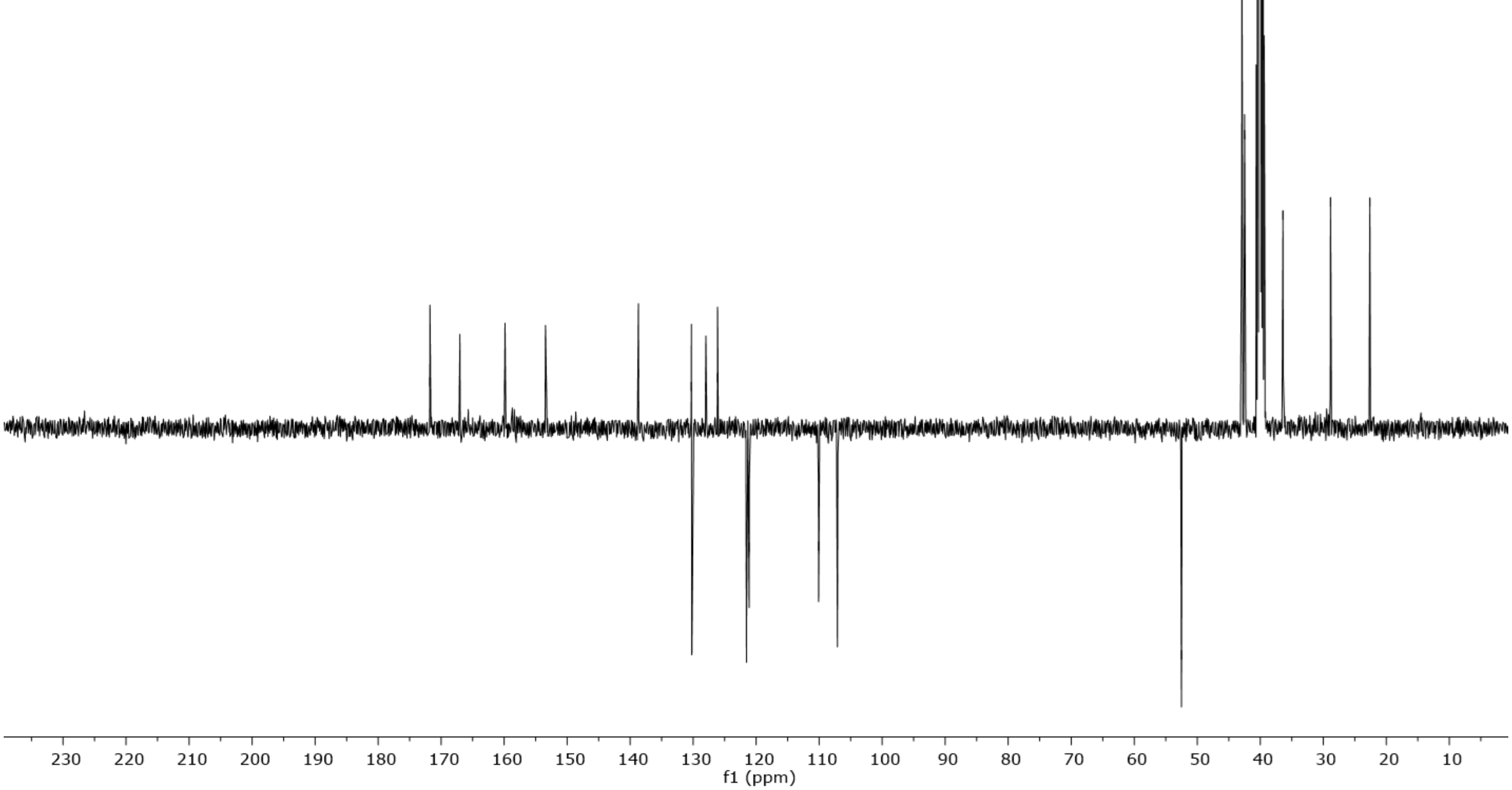

171.79
167.04
159.87
153.45
138.70
130.30
130.22
127.99
126.14
121.55
121.13
110.09
107.11
52.51
42.92
36.39
28.85
22.62
230 220 210 200 190 180 170 160 150 140 130 120 110 100 90 80 70 60 50 40 30 20 10
f1 (ppm)

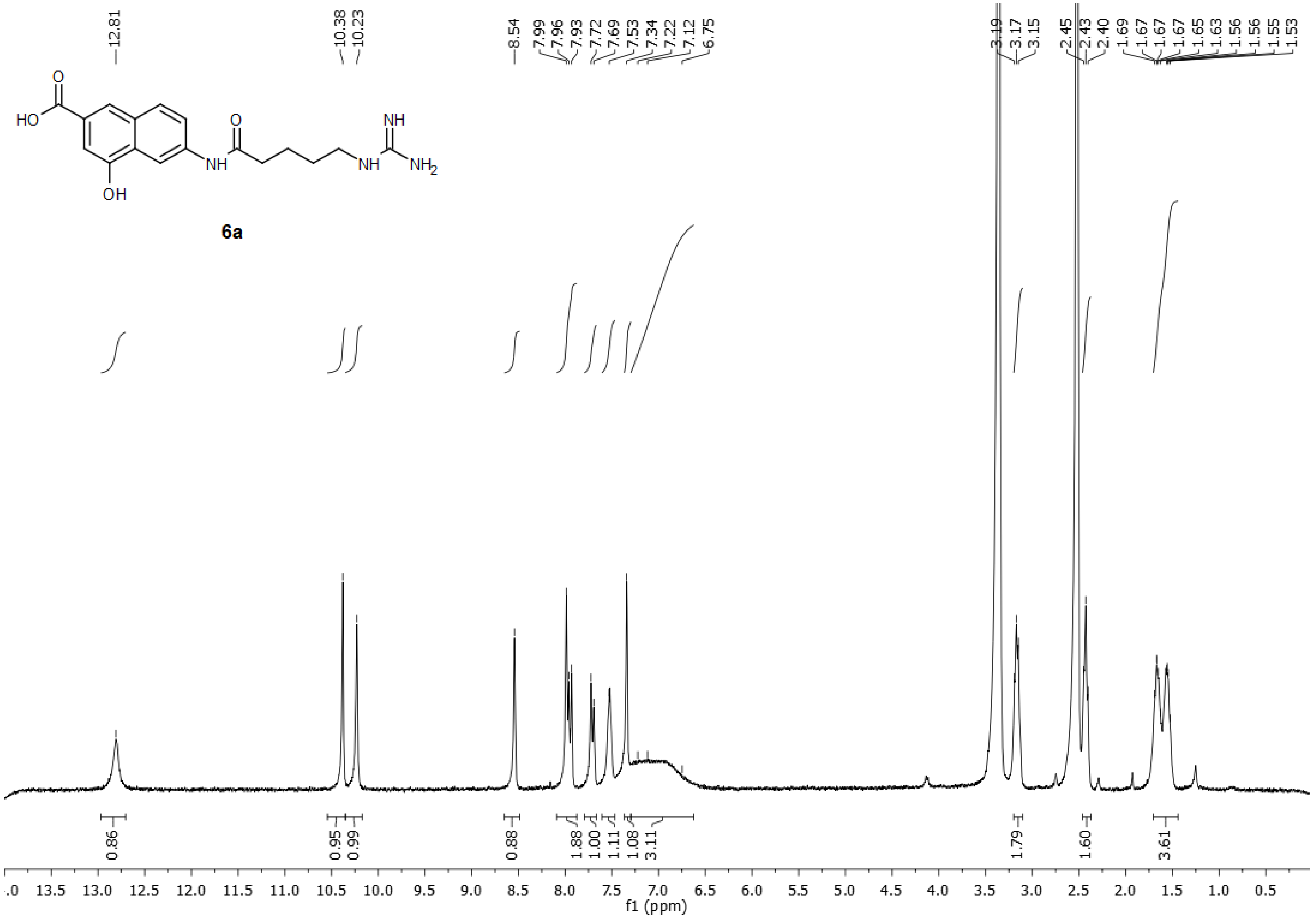
12.81
10.38
10.23
8.54
7.99
7.96
7.93
7.72
7.69
7.53
7.34
7.22
7.12
6.75
3.19
3.17
3.15
2.45
2.43
2.40
1.69
1.67
1.67
1.67
1.65
1.63
1.56
1.56
1.55
1.53
HO
O
OH
NH
O
NH
NH
NH2
0.86
0.95
0.99
0.88
1.88
1.00
1.11
1.08
3.11
1.79
1.60
3.61
f1 (ppm)

6a

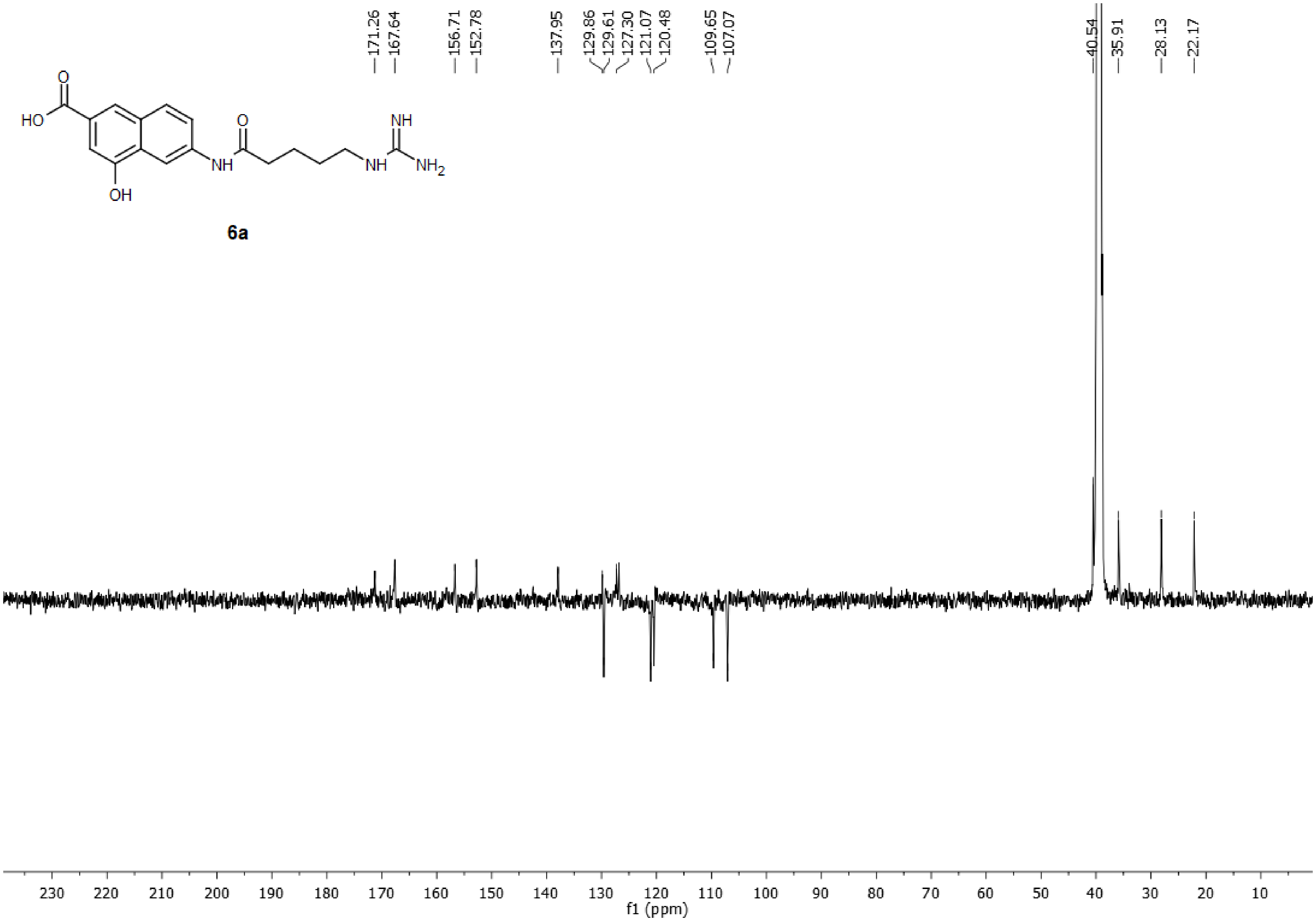
6a
171.26
167.64
156.71
152.78
137.95
129.86
129.61
127.30
121.07
120.48
109.65
107.07
40.54
35.91
28.13
22.17
230 220 210 200 190 180 170 160 150 140 130 120 110 100 90 80 70 60 50 40 30 20 10
f1 (ppm)

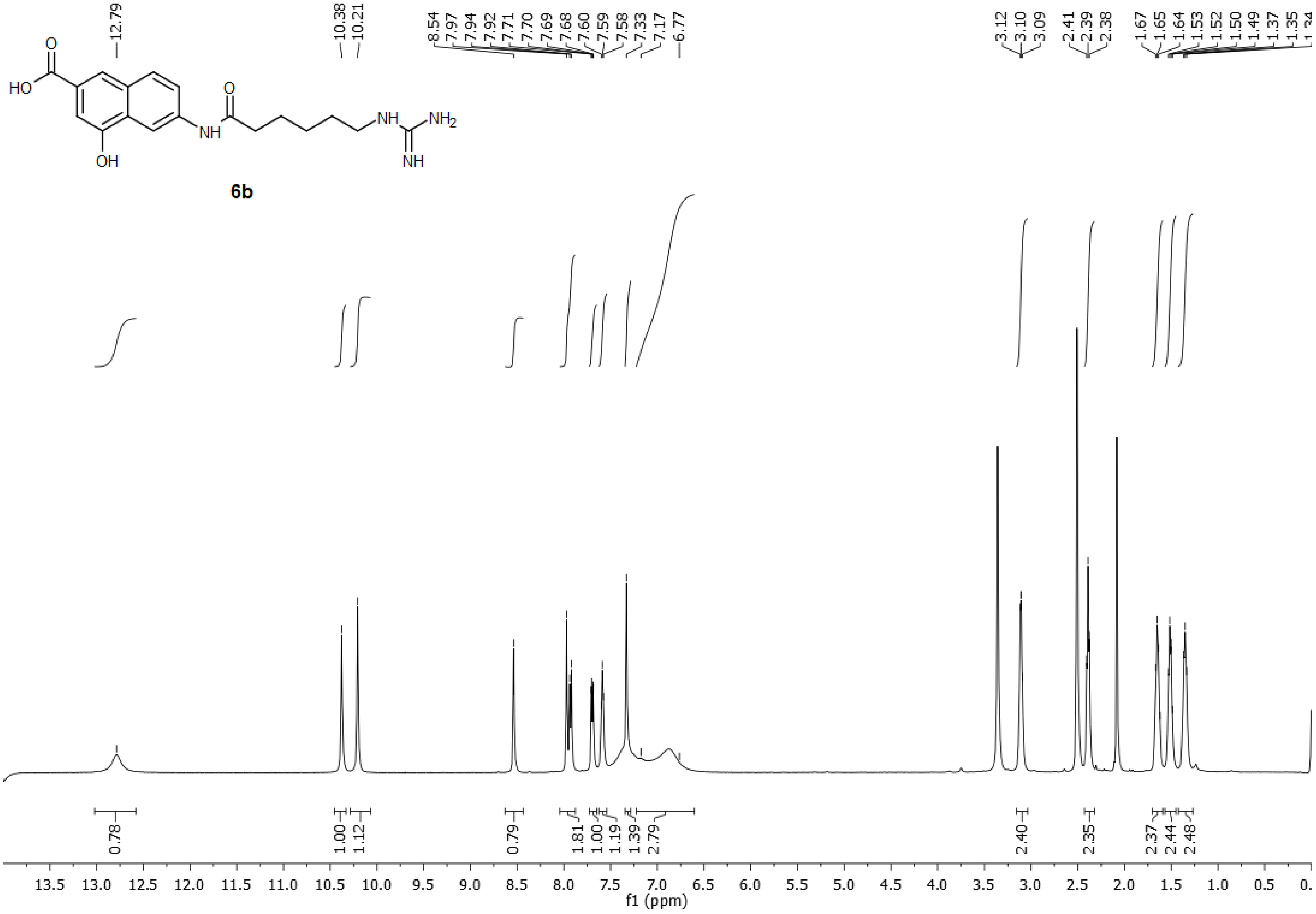

6b
f1 (ppm)

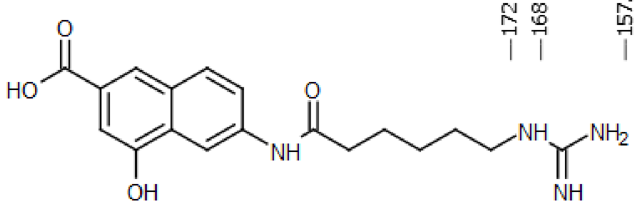

O
HO
O
NH
NH
NH2
OH
NH

6b

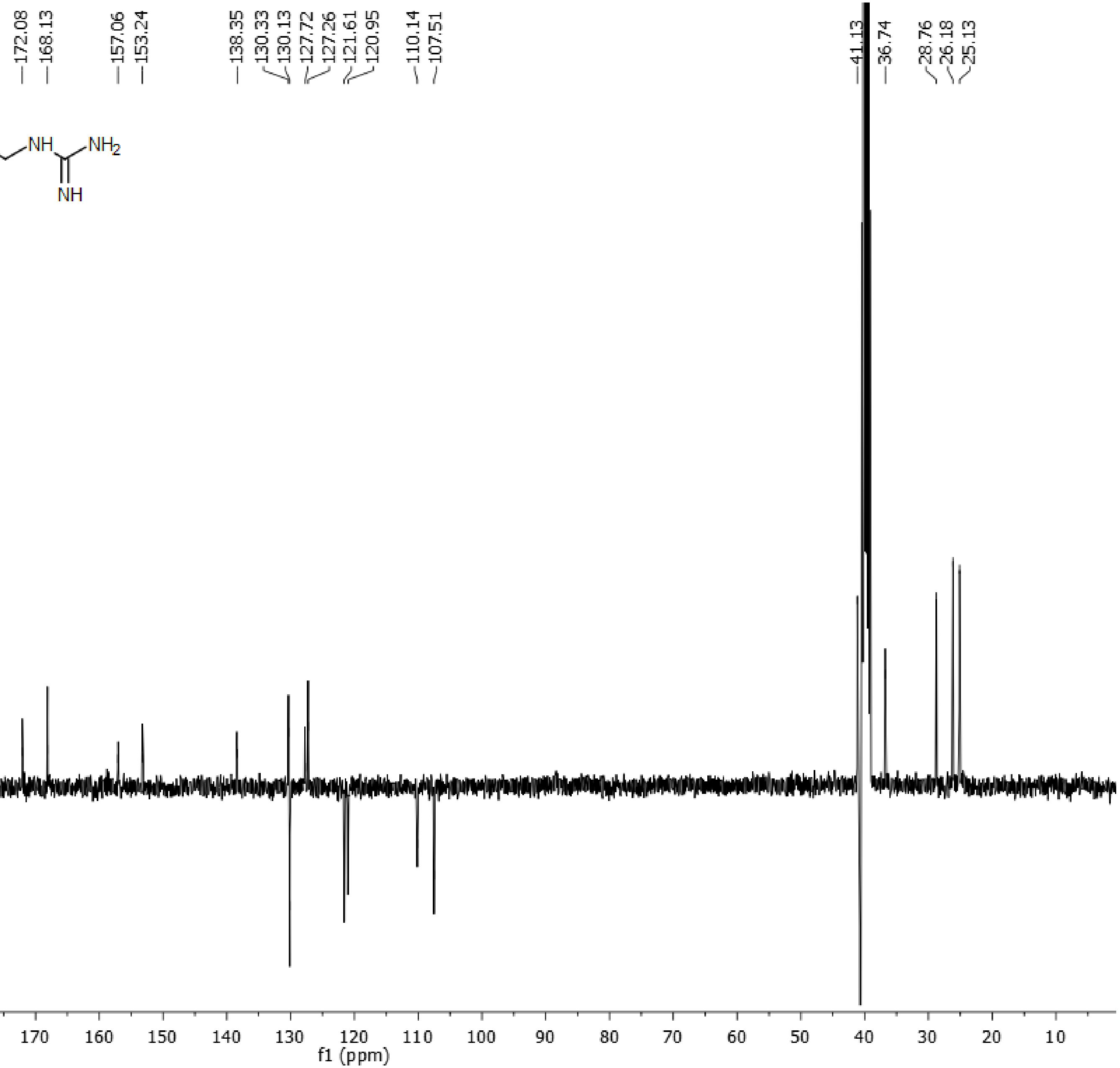

172.08
168.13
157.06
153.24
138.35
130.33
130.13
127.72
127.26
121.61
120.95
110.14
107.51
41.13
36.74
28.76
26.18
25.13
230 220 210 200 190 180 170 160 150 140 130 120 110 100 90 80 70 60 50 40 30 20 10
f1 (ppm)

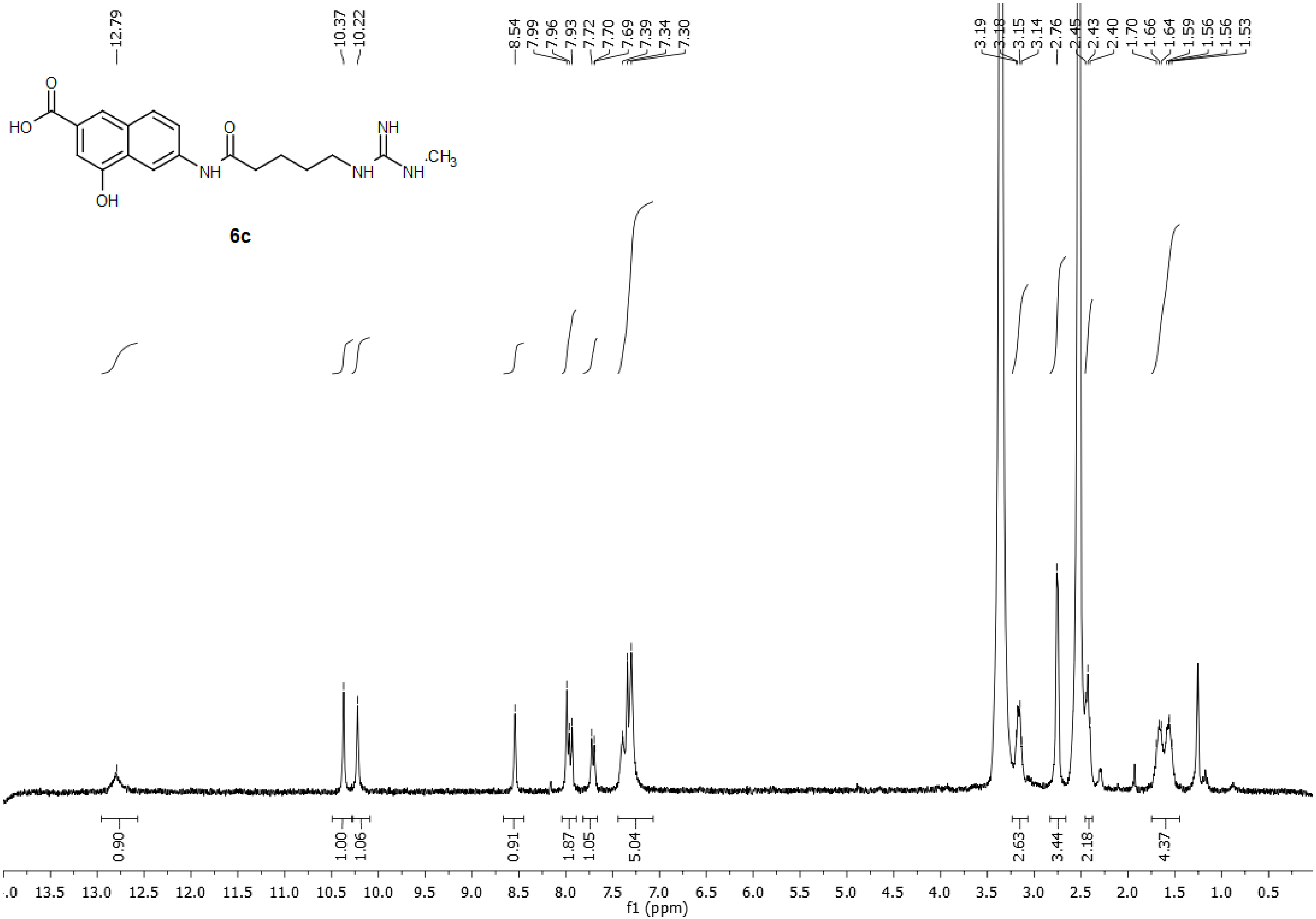

6c
12.79
10.37
10.22
8.54
7.99
7.96
7.93
7.72
7.70
7.69
7.39
7.34
7.30
3.19
3.18
3.15
3.14
2.76
2.45
2.43
2.40
1.70
1.66
1.64
1.59
1.56
1.56
1.53
0.90
1.00
1.06
0.91
1.87
1.05
5.04
2.63
3.44
2.18
4.37
f1 (ppm)

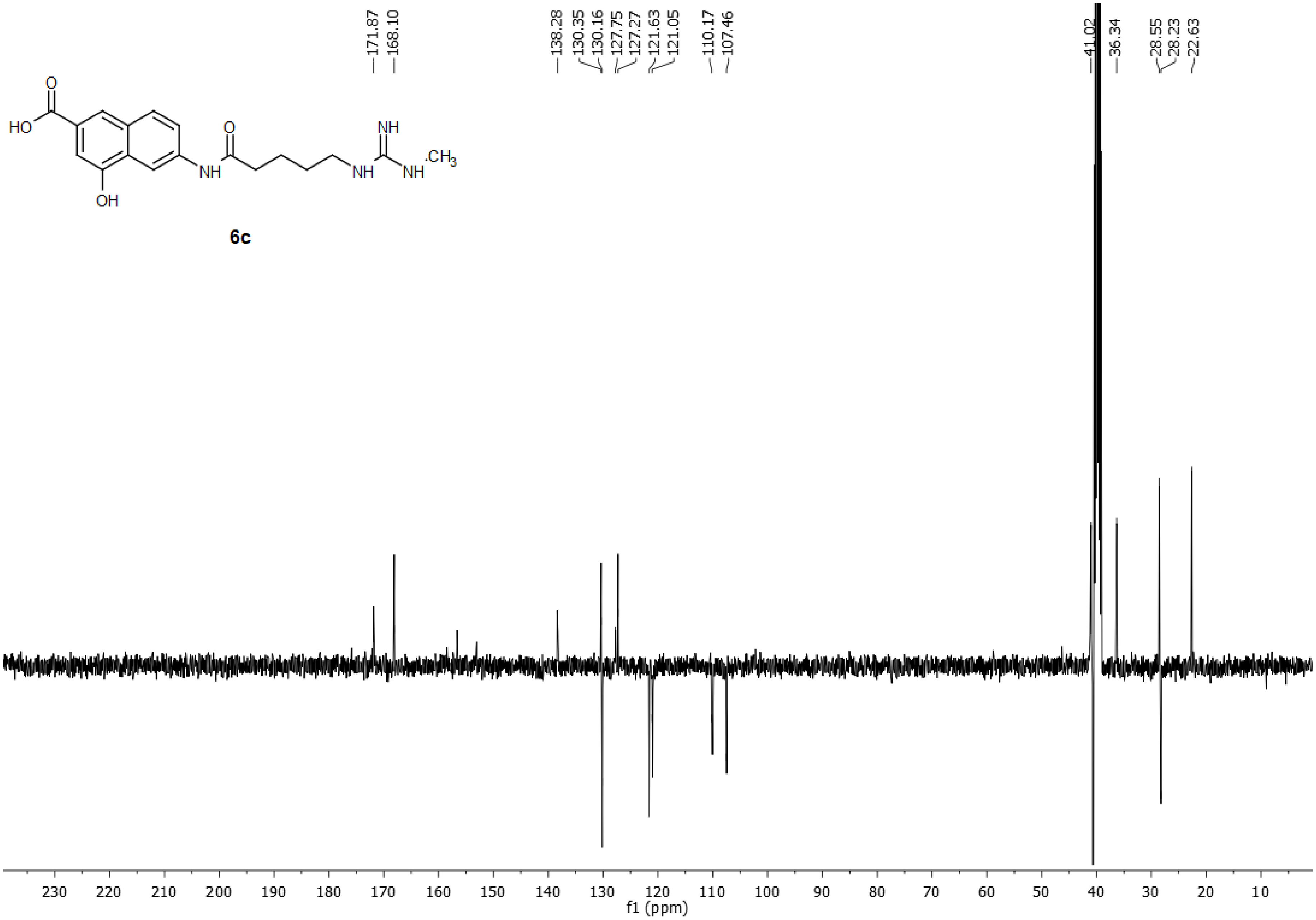
HO
O
OH
O
NH
NH
NH
NH
CH3
171.87
168.10
138.28
130.35
130.16
127.75
127.27
121.63
121.05
110.17
107.46
41.02
36.34
28.55
28.23
22.63
230
220
210
200
190
180
170
160
150
140
130
120
110
100
90
80
70
60
50
40
30
20
10
f1 (ppm)

6c

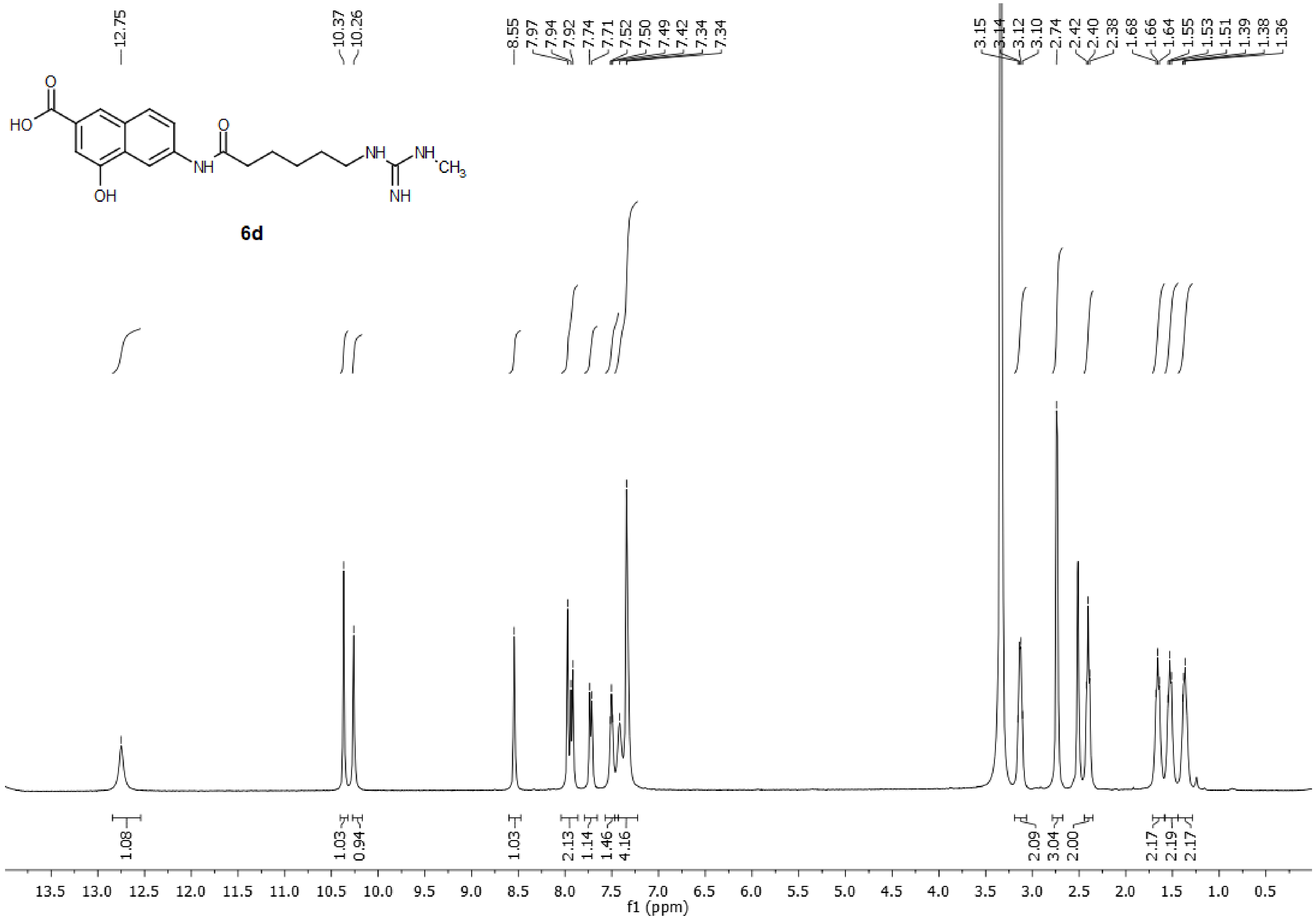

12.75
10.37
10.26
8.55
7.97
7.94
7.92
7.74
7.71
7.52
7.50
7.49
7.42
7.34
7.34
3.15
3.14
3.12
3.10
2.74
2.42
2.40
2.38
1.68
1.66
1.64
1.55
1.53
1.51
1.39
1.38
1.36
HO
O
OH
NH
O
NH
NH
CH3
NH
6d
1.08
1.03
0.94
1.03
2.13
1.14
1.46
4.16
2.09
3.04
2.00
2.17
2.19
2.17
f1 (ppm)

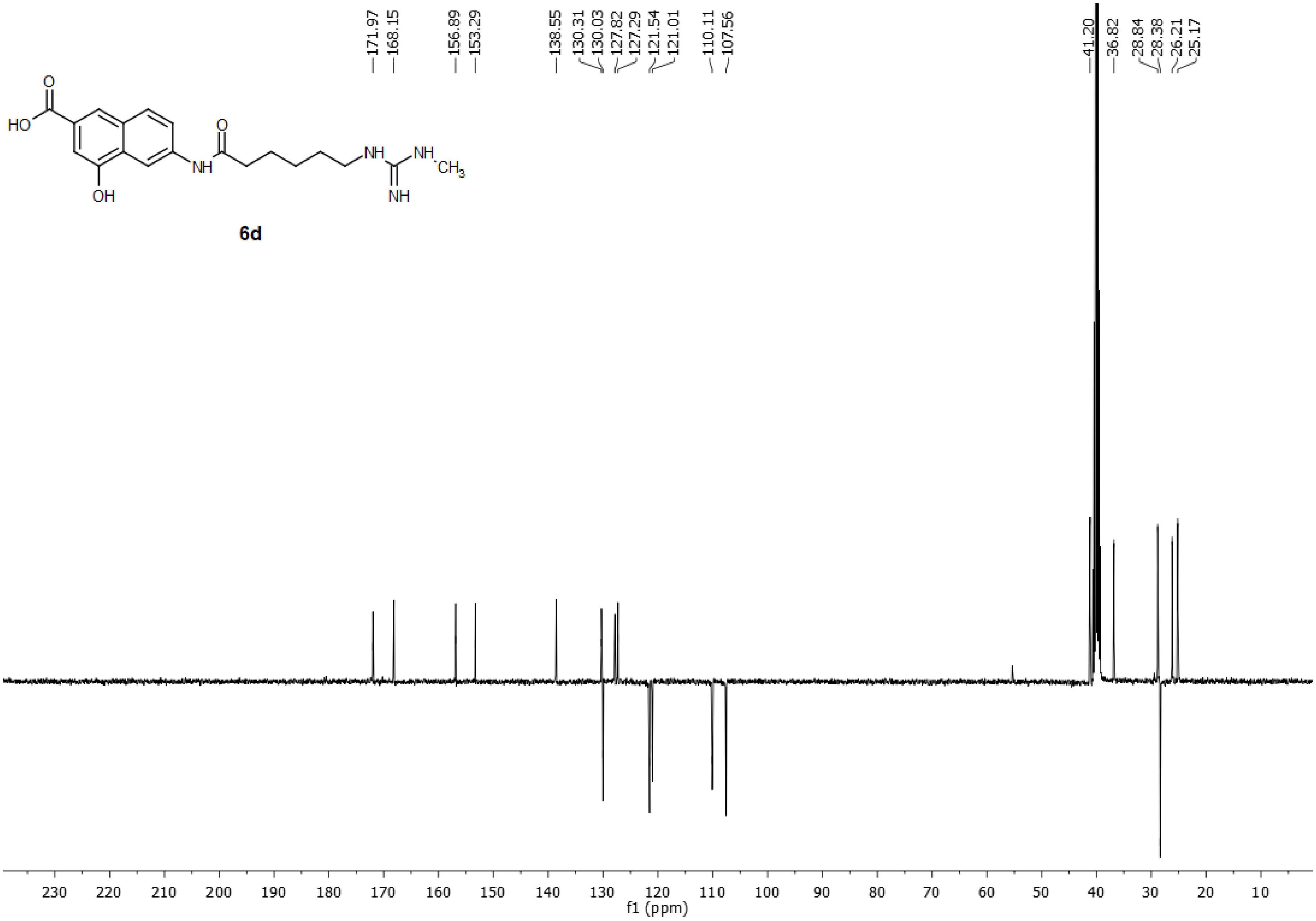

HO
O
OH
NH
O
NH
NH
CH3
NH
6d
171.97
168.15
156.89
153.29
138.55
130.31
130.03
127.82
127.29
121.54
121.01
110.11
107.56
41.20
36.82
28.84
28.38
26.21
25.17
230 220 210 200 190 180 170 160 150 140 130 120 110 100 90 80 70 60 50 40 30 20 10
f1 (ppm)

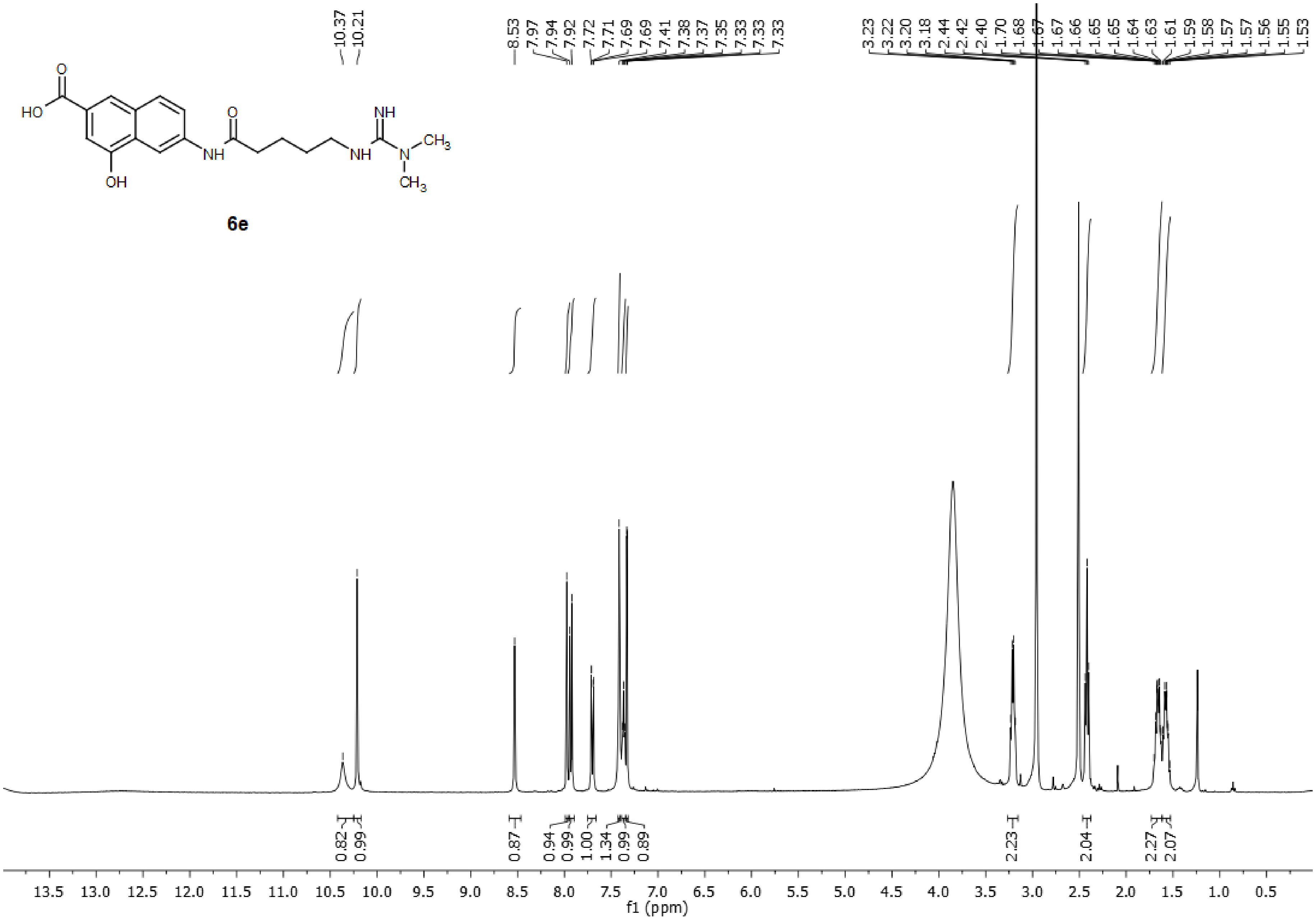

6e
10.37
10.21
8.53
7.97
7.94
7.92
7.72
7.71
7.69
7.69
7.41
7.38
7.37
7.35
7.33
7.33
7.33
3.23
3.22
3.20
3.18
2.44
2.42
2.40
1.70
1.68
1.67
1.67
1.66
1.65
1.65
1.64
1.63
1.61
1.59
1.58
1.57
1.57
1.56
1.55
1.53
0.82
0.99
0.87
0.94
0.99
1.00
1.34
0.99
0.89
2.23
2.04
2.27
2.07
f1 (ppm)

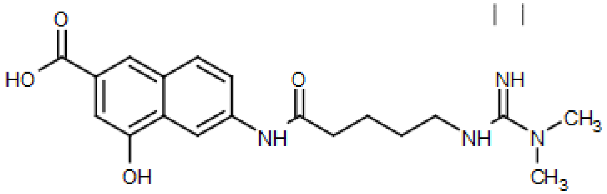

6e

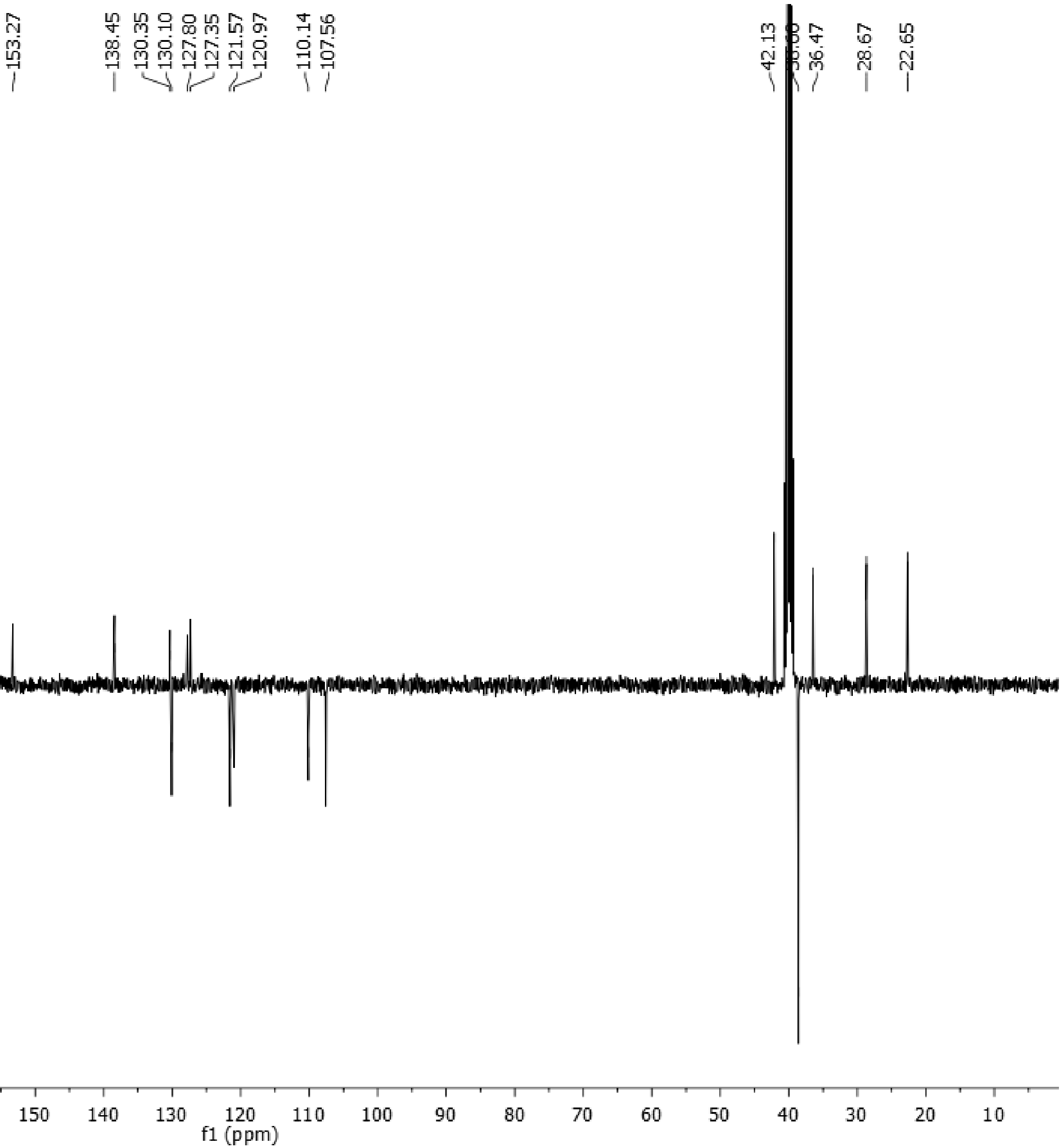
171.81
168.13
156.20
153.27
138.45
130.35
130.10
127.80
127.35
121.57
120.97
110.14
107.56
42.13
36.47
28.67
22.65
f1 (ppm)

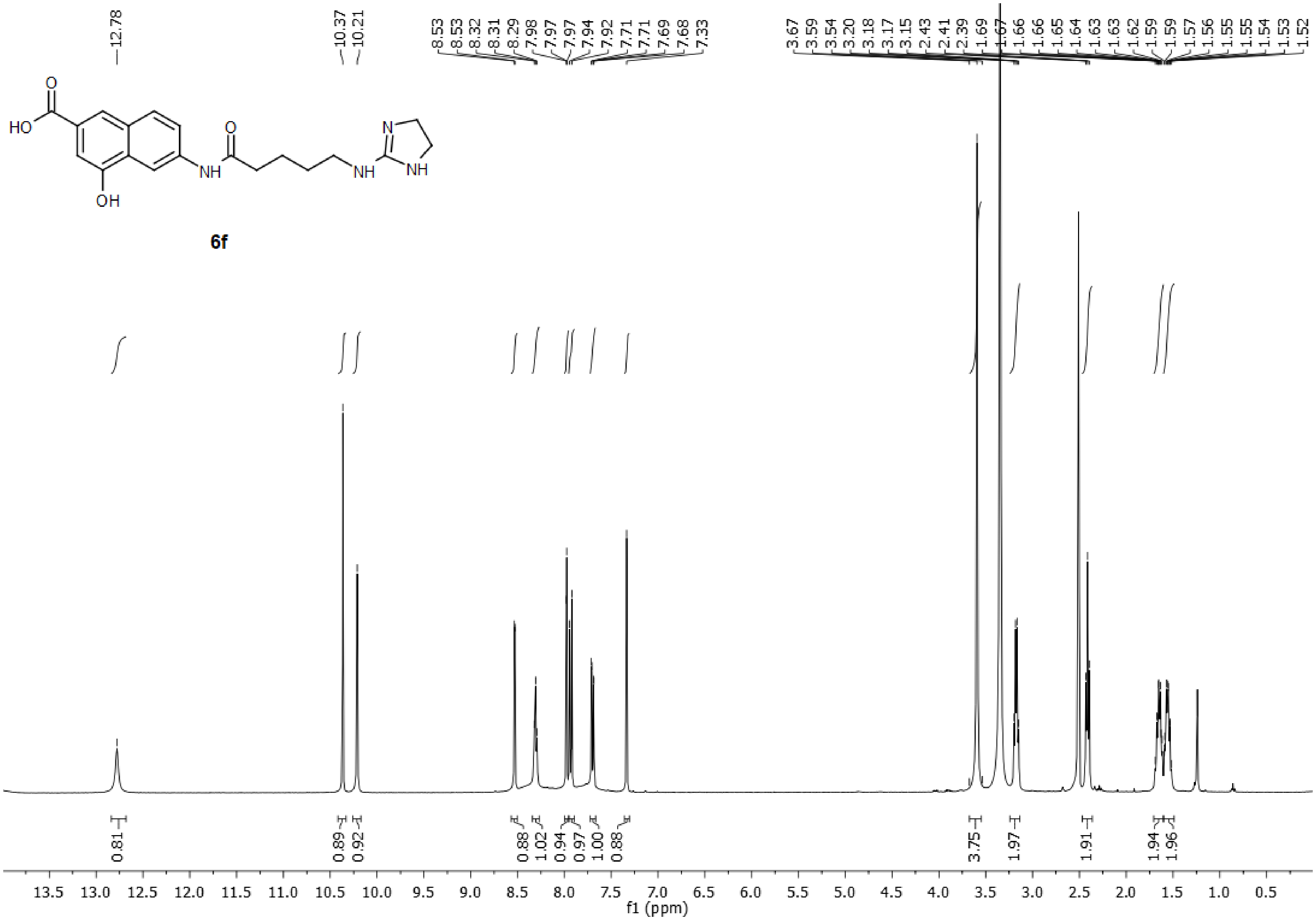
12.78
10.37
10.21
8.53 8.53 8.32 8.31 8.29 7.98 7.97 7.97 7.94 7.92 7.71 7.71 7.69 7.68 7.33
3.67 3.59 3.54 3.20 3.18 3.17 3.15 2.43 2.41 2.39 1.69 1.67 1.66 1.66 1.65 1.64 1.63 1.63 1.62 1.59 1.59 1.57 1.56 1.55 1.55 1.54 1.53 1.52
HO
O
OH
O
NH
NH
N
NH
0.81
0.89 0.92
0.88 1.02 0.94 0.97 1.00 0.88
3.75 1.97 1.91 1.94 1.96
13.5 13.0 12.5 12.0 11.5 11.0 10.5 10.0 9.5 9.0 8.5 8.0 7.5 7.0 6.5 6.0 5.5 5.0 4.5 4.0 3.5 3.0 2.5 2.0 1.5 1.0 0.5
f1 (ppm)

6f

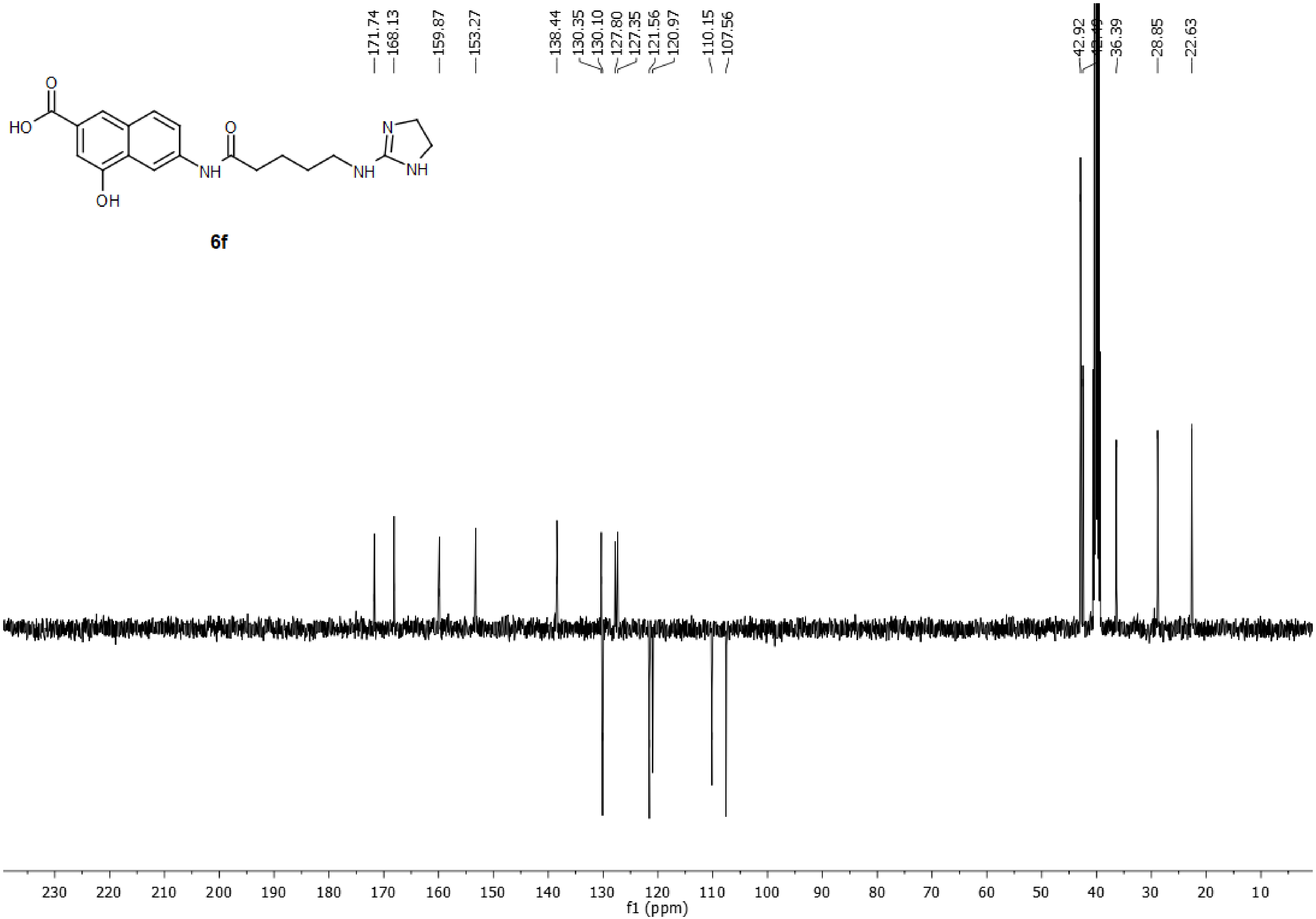
6f
171.74
168.13
159.87
153.27
138.44
130.35
130.10
127.80
127.35
121.56
120.97
110.15
107.56
42.92
36.39
28.85
22.63
HO
O
OH
NH
NH
N
NH
230 220 210 200 190 180 170 160 150 140 130 120 110 100 90 80 70 60 50 40 30 20 10
f1 (ppm)

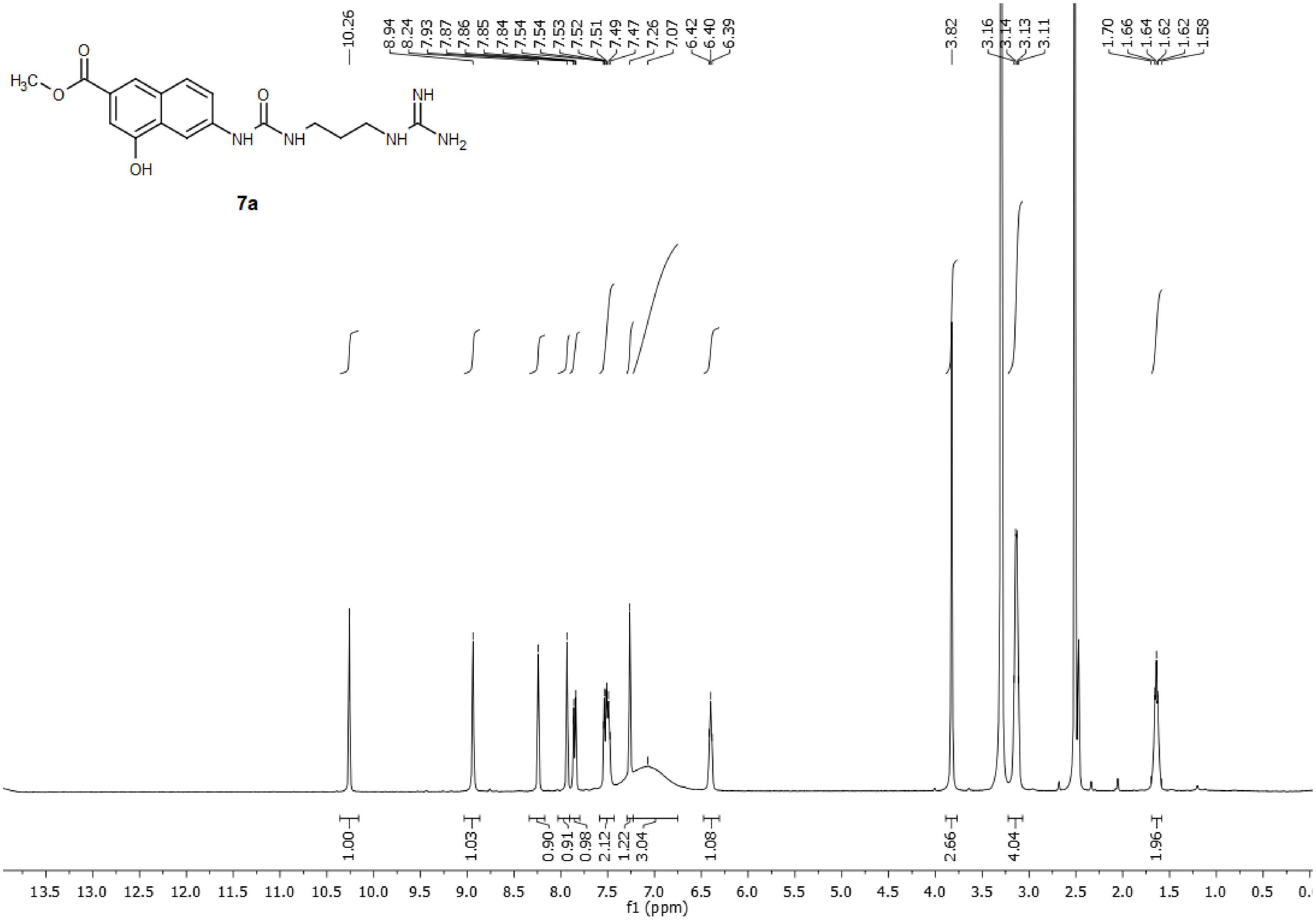
7a
10.26
8.94
8.24
7.93
7.87
7.86
7.85
7.84
7.54
7.54
7.53
7.52
7.51
7.49
7.47
7.26
7.07
6.42
6.40
6.39
3.82
3.16
3.14
3.13
3.11
1.70
1.66
1.64
1.62
1.62
1.58
1.00
1.03
0.90
0.91
0.98
2.12
1.22
3.04
1.08
2.66
4.04
1.96
f1 (ppm)

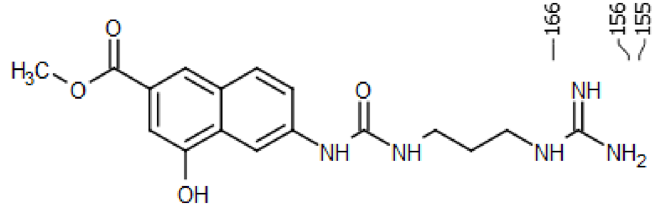
H3C
O
O
OH
NH
O
NH
NH
NH
NH2

7a

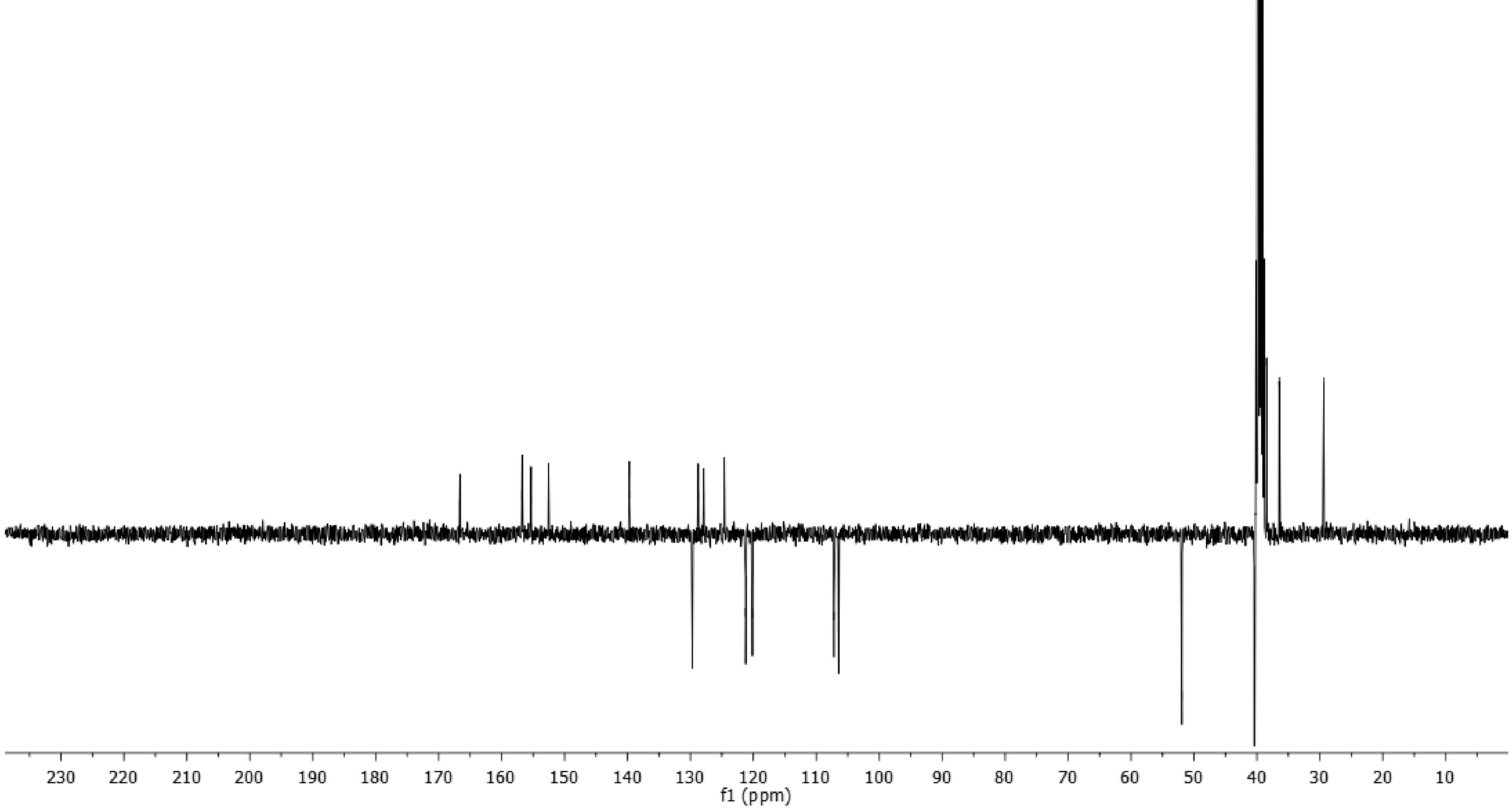
166.62
156.75
155.32
152.54
139.72
129.70
128.77
127.91
124.63
121.23
120.16
107.22
106.46
51.92
36.45
29.39
230 220 210 200 190 180 170 160 150 140 130 120 110 100 90 80 70 60 50 40 30 20 10
f1 (ppm)

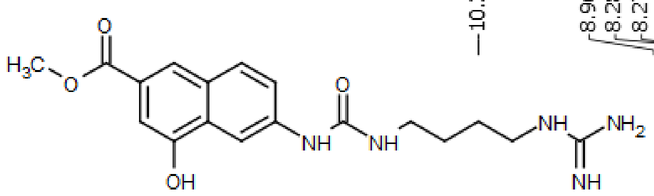
O
H_3C
O
O
NH
NH
NH
NH_2
NH
OH

7b

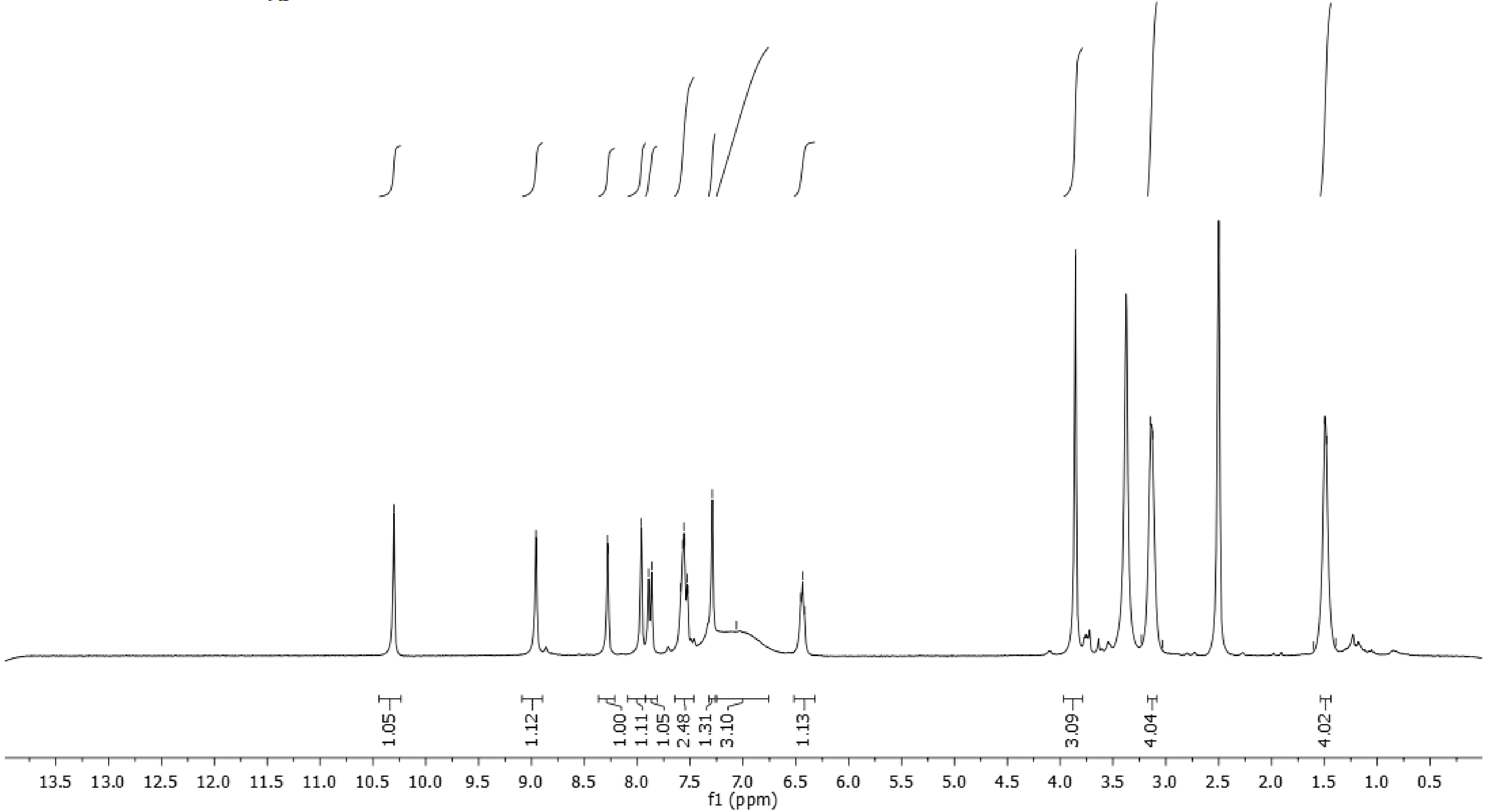
10.30
8.96
8.28
8.27
7.96
7.89
7.86
7.59
7.57
7.56
7.55
7.53
7.52
7.29
7.06
6.45
6.44
6.42
3.85
3.23
3.14
3.12
3.03
1.60
1.50
1.48
1.39
1.05
1.12
1.00
1.11
1.05
2.48
1.31
3.10
1.13
3.09
4.04
4.02
13.5 13.0 12.5 12.0 11.5 11.0 10.5 10.0 9.5 9.0 8.5 8.0 7.5 7.0 6.5 6.0 5.5 5.0 4.5 4.0 3.5 3.0 2.5 2.0 1.5 1.0 0.5
f1 (ppm)

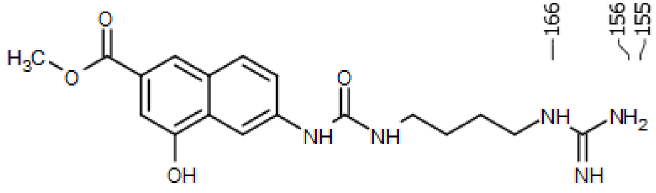

O
H3C
O
O
NH
NH
NH
NH2
NH
OH

7b

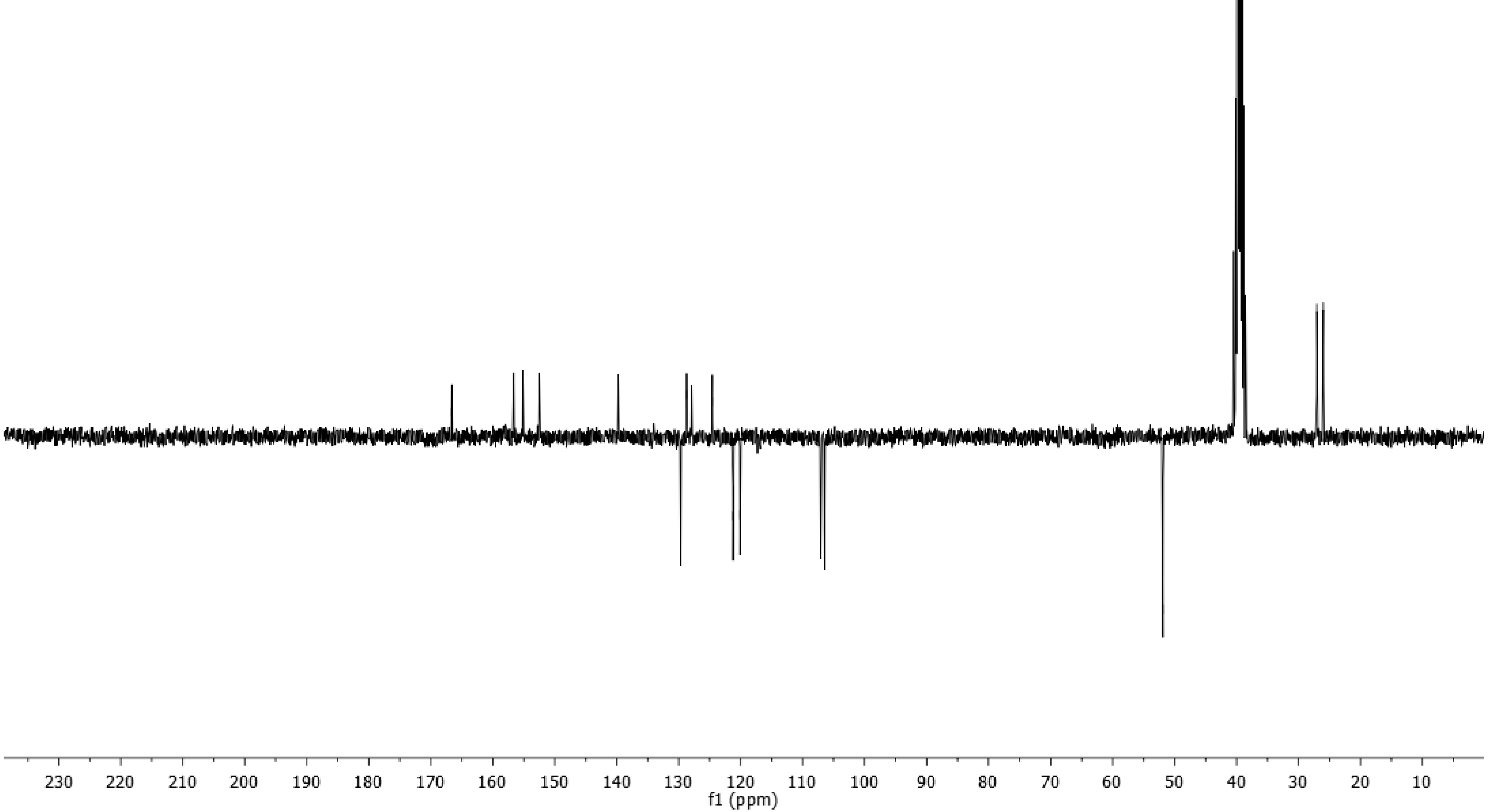

166.63
156.67
155.18
152.52
139.79
129.70
128.72
127.93
124.58
121.23
120.10
107.08
106.45
51.92
27.04
26.00
230 220 210 200 190 180 170 160 150 140 130 120 110 100 90 80 70 60 50 40 30 20 10
f1 (ppm)

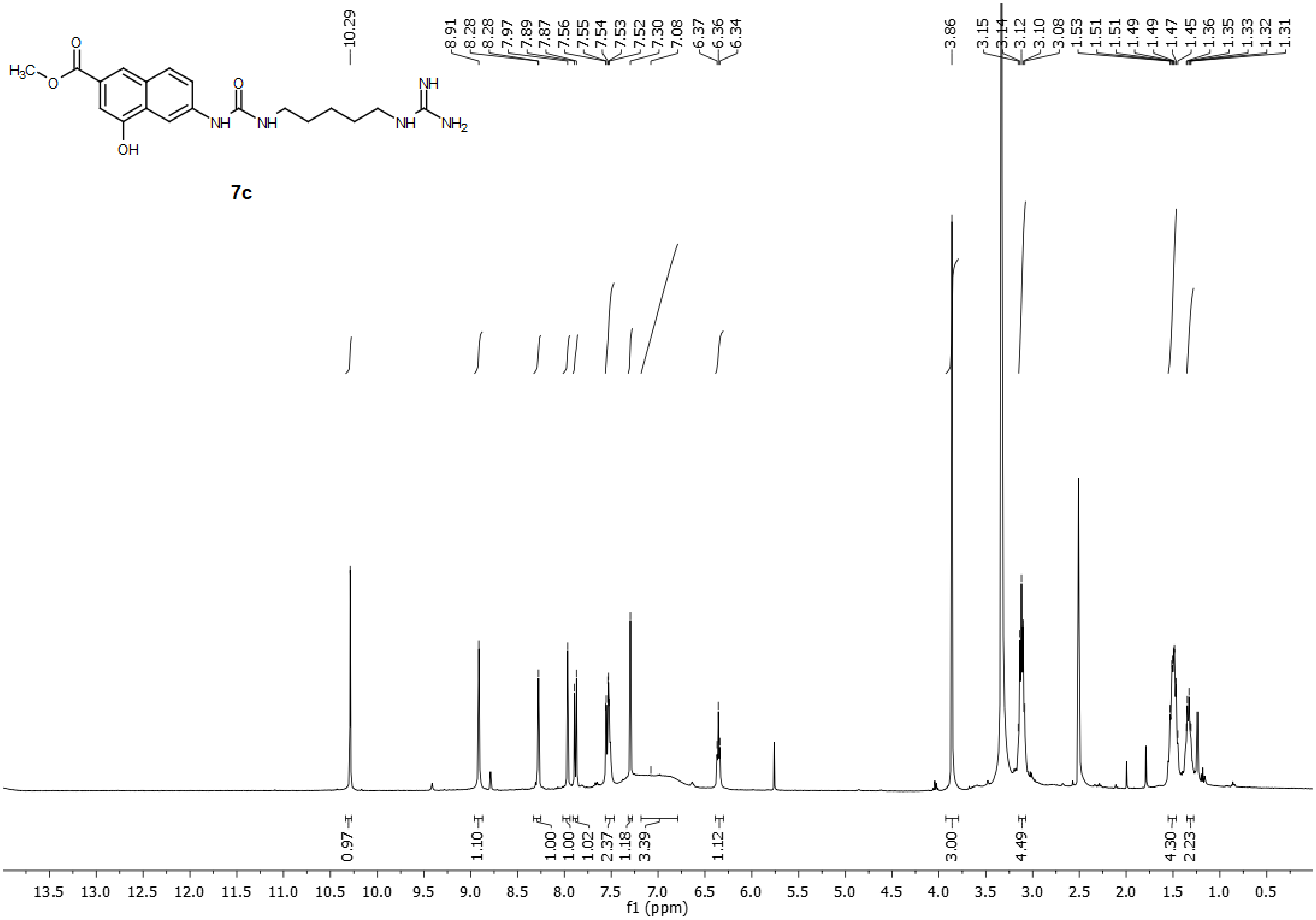
H3C O O OH NH NH NH NH NH2
10.29
8.91 8.28 8.28 7.97 7.89 7.87 7.56 7.55 7.54 7.53 7.52 7.30 7.08 6.37 6.36 6.34
3.86 3.15 3.14 3.12 3.10 3.08 1.53 1.51 1.51 1.49 1.49 1.47 1.45 1.36 1.35 1.33 1.32 1.31
0.97 1.10 1.00 1.00 1.02 2.37 1.18 3.39 1.12 3.00 4.49 4.30 2.23
13.5 13.0 12.5 12.0 11.5 11.0 10.5 10.0 9.5 9.0 8.5 8.0 7.5 7.0 6.5 6.0 5.5 5.0 4.5 4.0 3.5 3.0 2.5 2.0 1.5 1.0 0.5
f1 (ppm)

7c

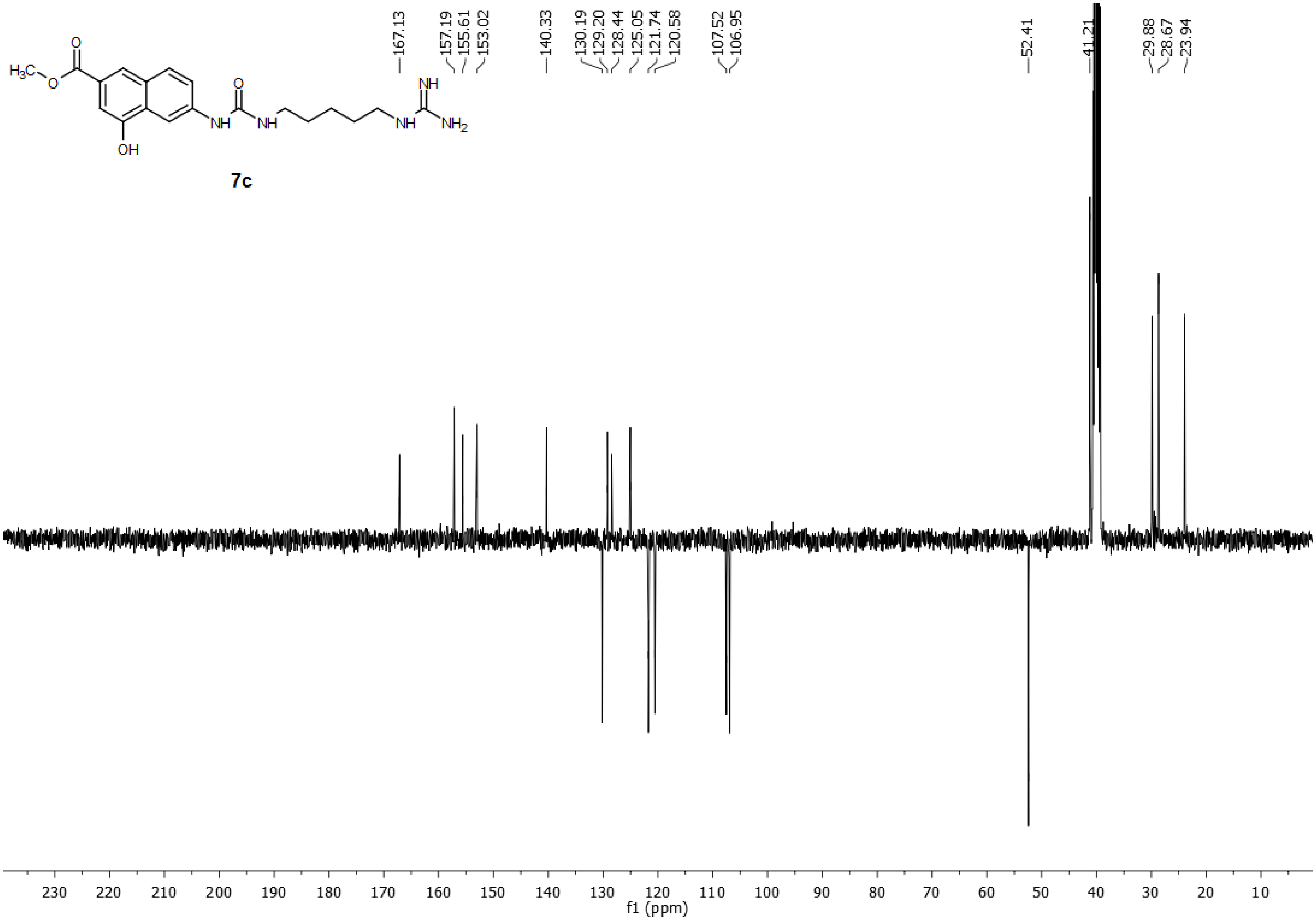
H3C
O
O
NH
O
NH
NH
NH
NH2
OH
167.13
157.19
155.61
153.02
140.33
130.19
129.20
128.44
125.05
121.74
120.58
107.52
106.95
52.41
41.21
29.88
28.67
23.94
230 220 210 200 190 180 170 160 150 140 130 120 110 100 90 80 70 60 50 40 30 20 10
f1 (ppm)

7c

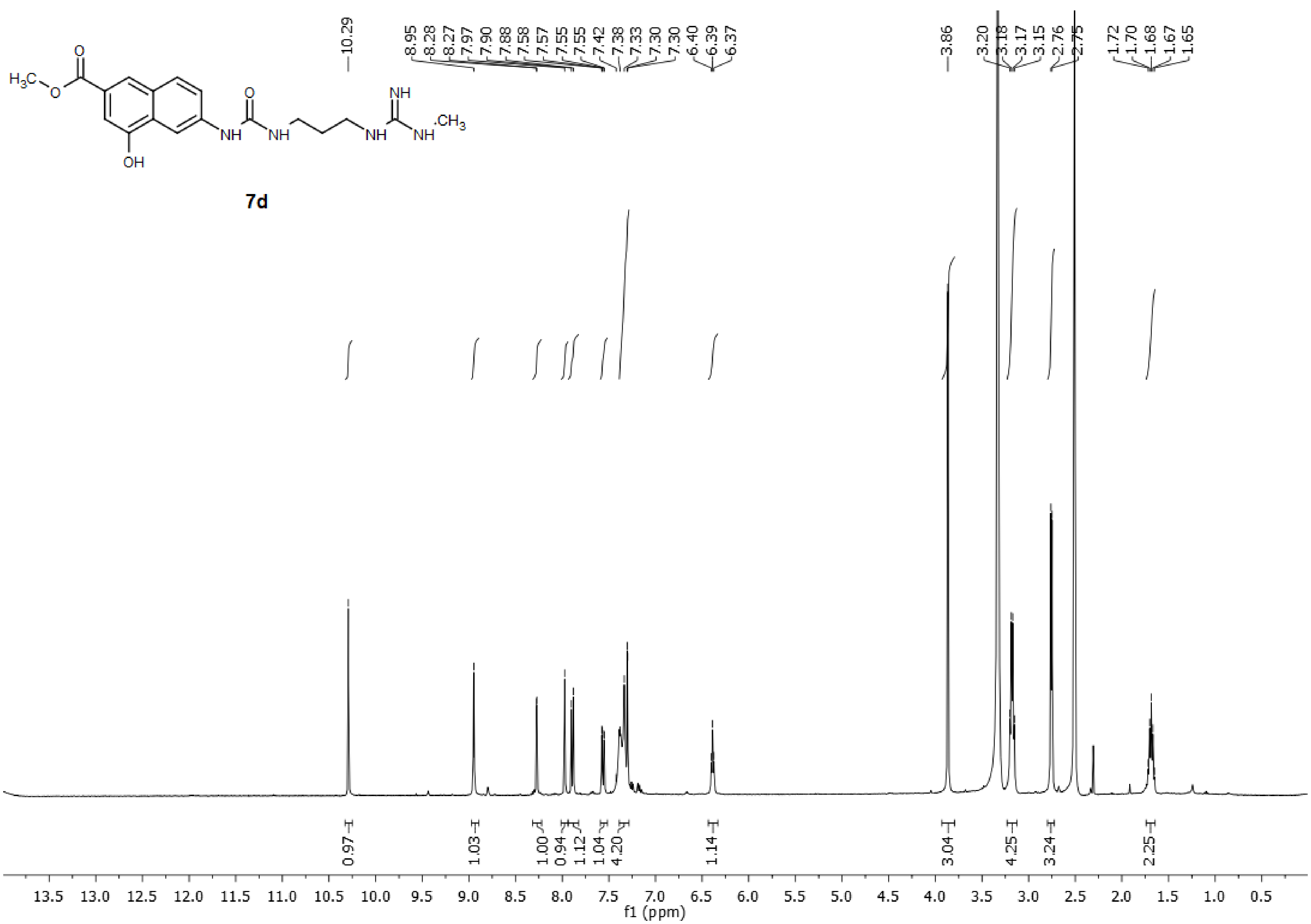

7d

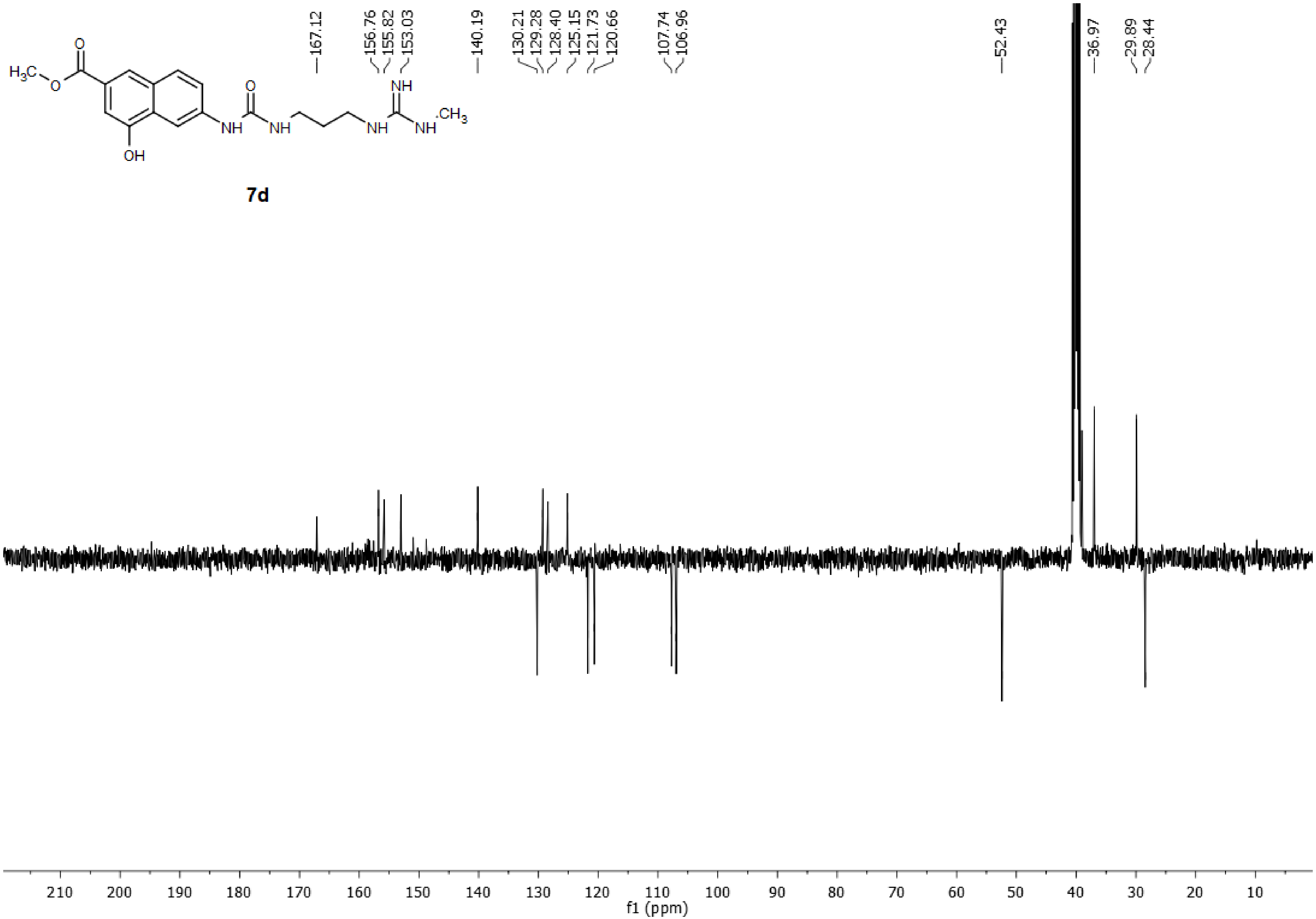
167.12
156.76
155.82
153.03
140.19
130.21
129.28
128.40
125.15
121.73
120.66
107.74
106.96
52.43
36.97
29.89
28.44
7d
f1 (ppm)

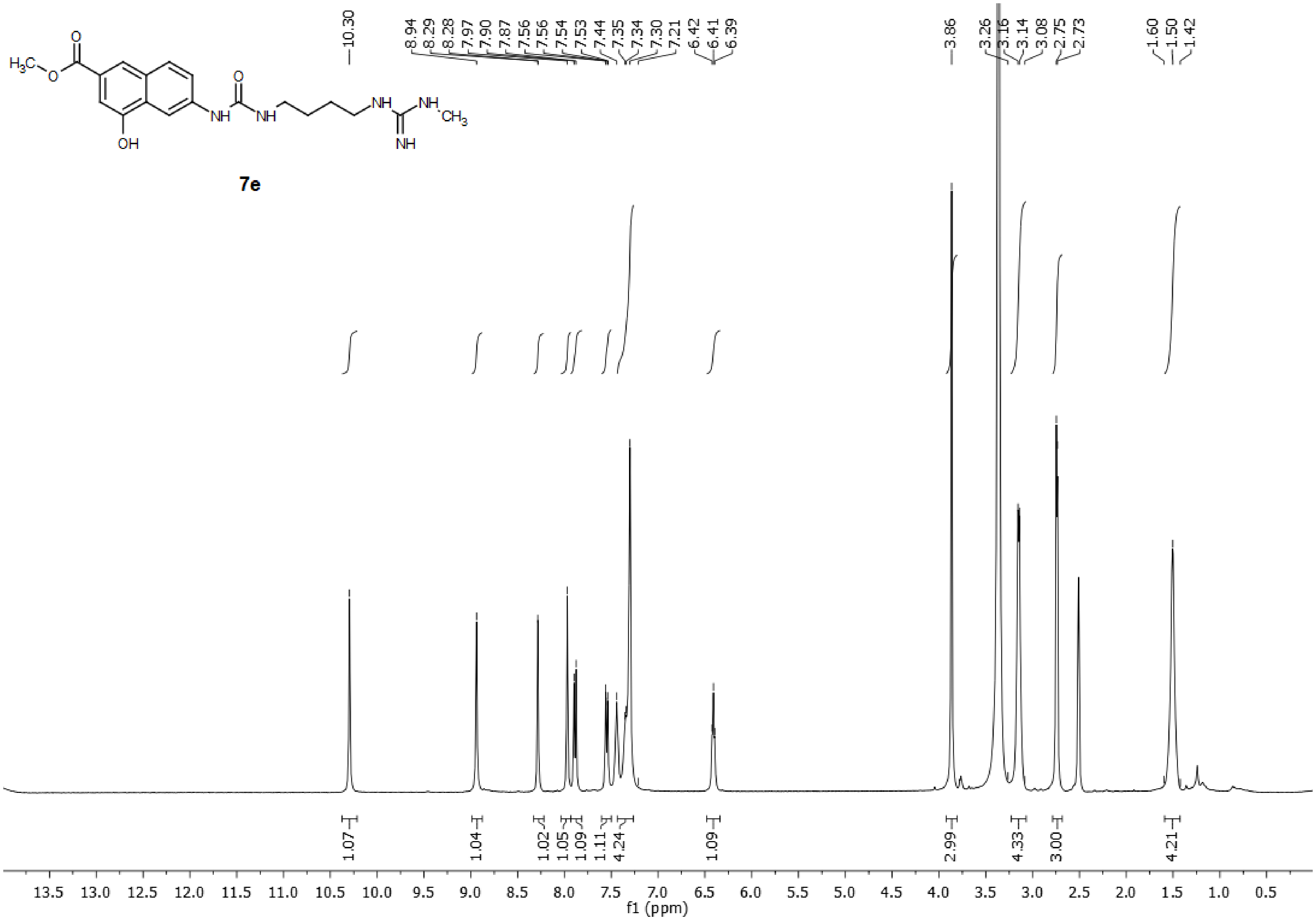
7e
10.30
8.94 8.29 8.28 7.97 7.90 7.87 7.56 7.56 7.54 7.53 7.44 7.35 7.34 7.30 7.21 6.42 6.41 6.39
3.86 3.26 3.16 3.14 3.08 2.75 2.73 1.60 1.50 1.42
1.07 1.04 1.02 1.05 1.09 1.11 4.24 1.09 2.99 4.33 3.00 4.21
f1 (ppm)

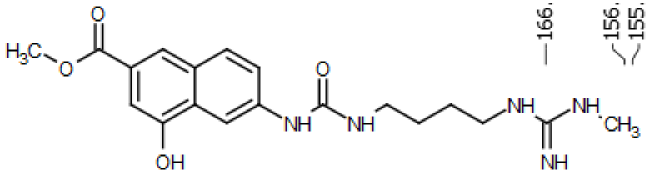

7e

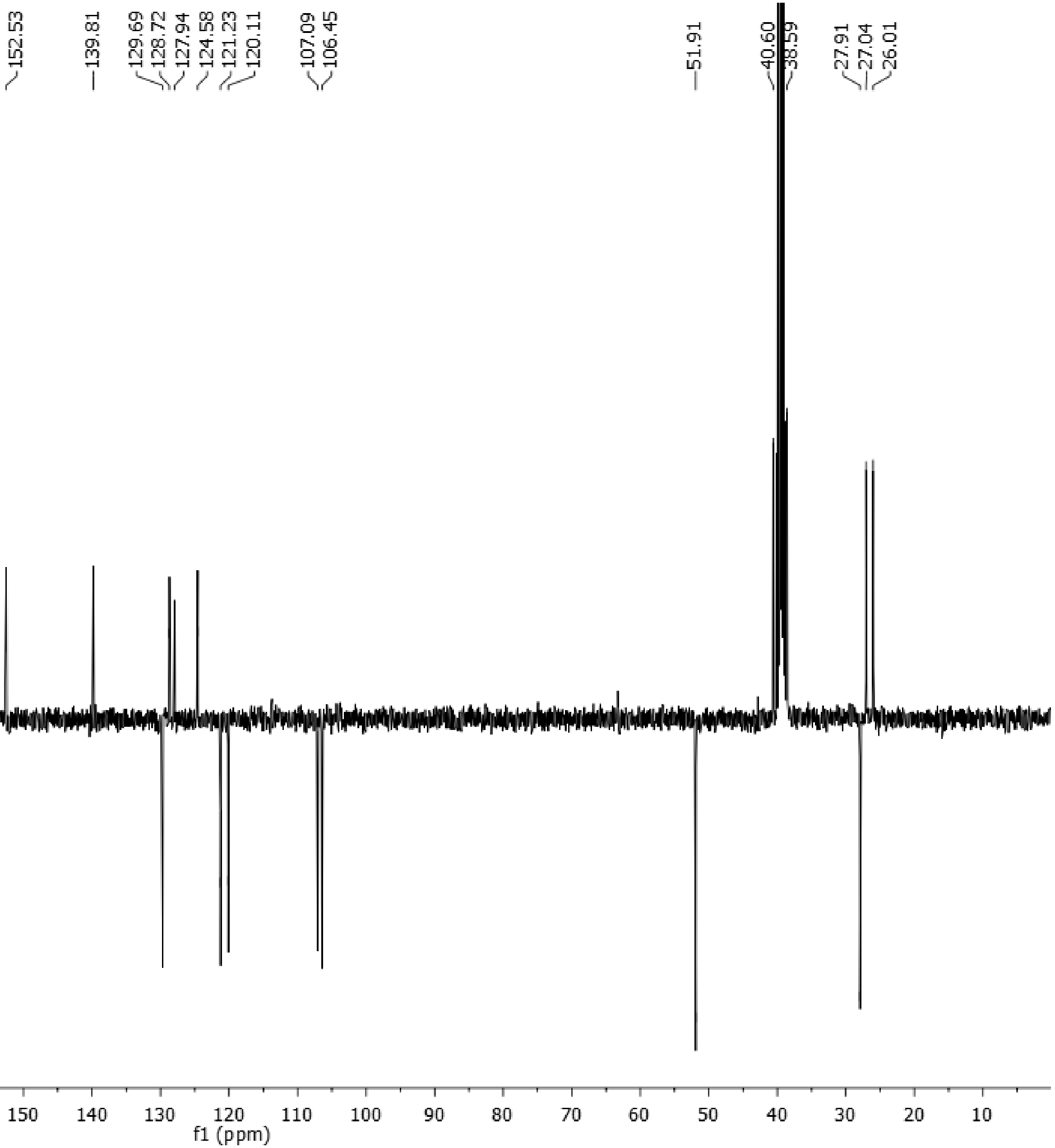

166.63
156.23
155.21
152.53
139.81
129.69
128.72
127.94
124.58
121.23
120.11
107.09
106.45
51.91
40.60
38.59
27.91
27.04
26.01
230 220 210 200 190 180 170 160 150 140 130 120 110 100 90 80 70 60 50 40 30 20 10
f1 (ppm)

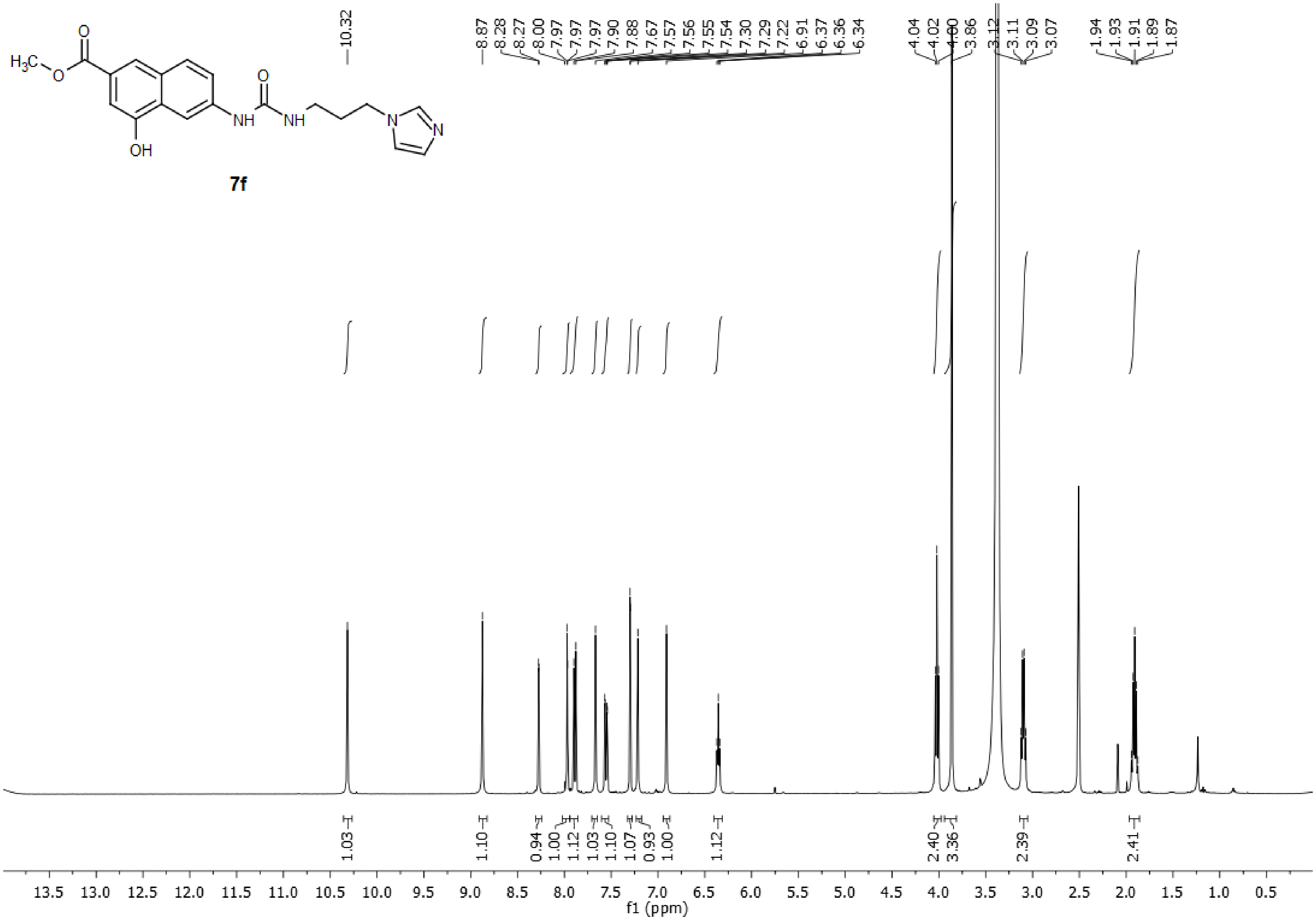
7f
f1 (ppm)

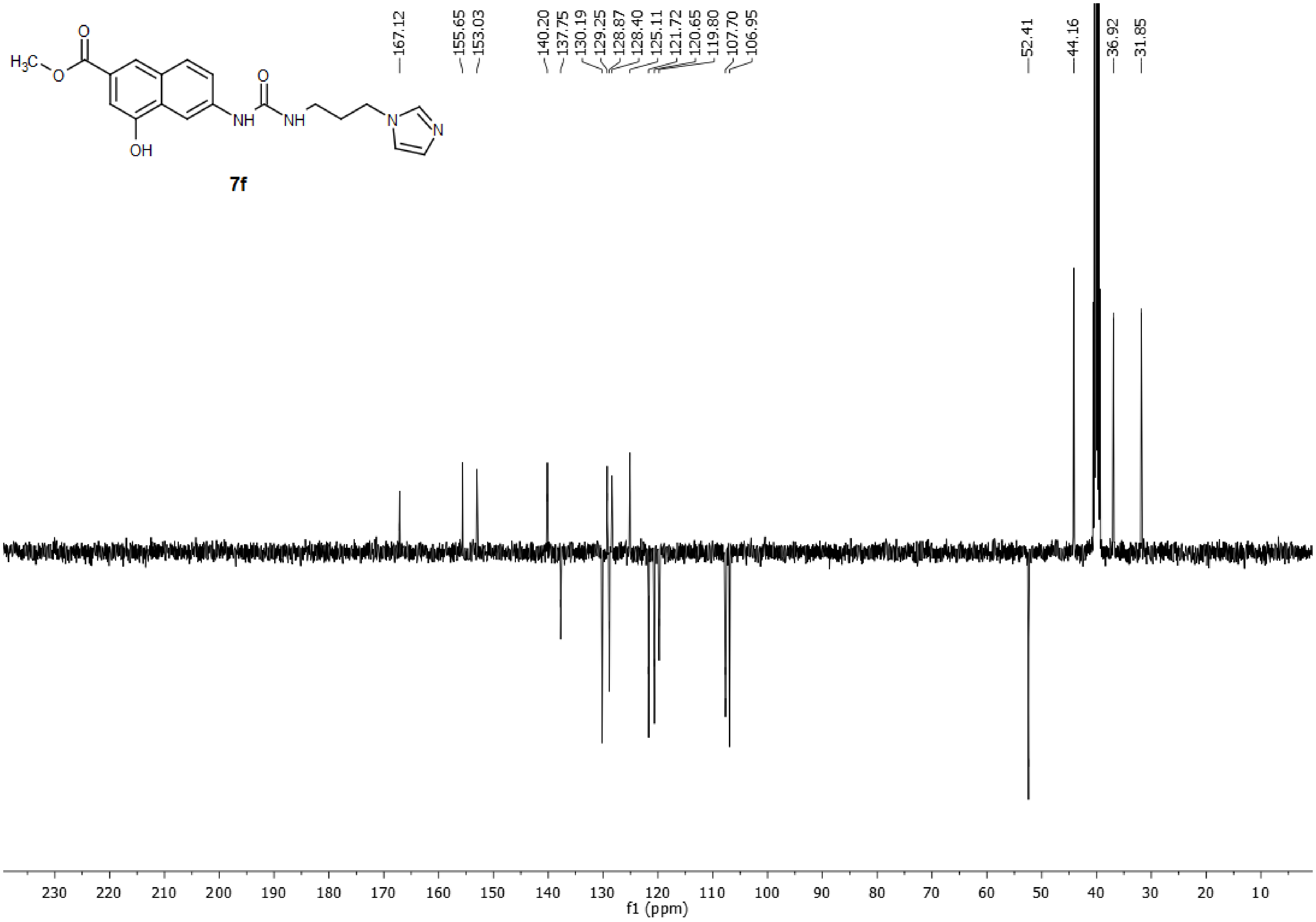

H3C
O
O
NH
O
NH
N
N
OH
7f
167.12
155.65
153.03
140.20
137.75
130.19
129.25
128.87
128.40
125.11
121.72
120.65
119.80
107.70
106.95
52.41
44.16
36.92
31.85
230 220 210 200 190 180 170 160 150 140 130 120 110 100 90 80 70 60 50 40 30 20 10
f1 (ppm)

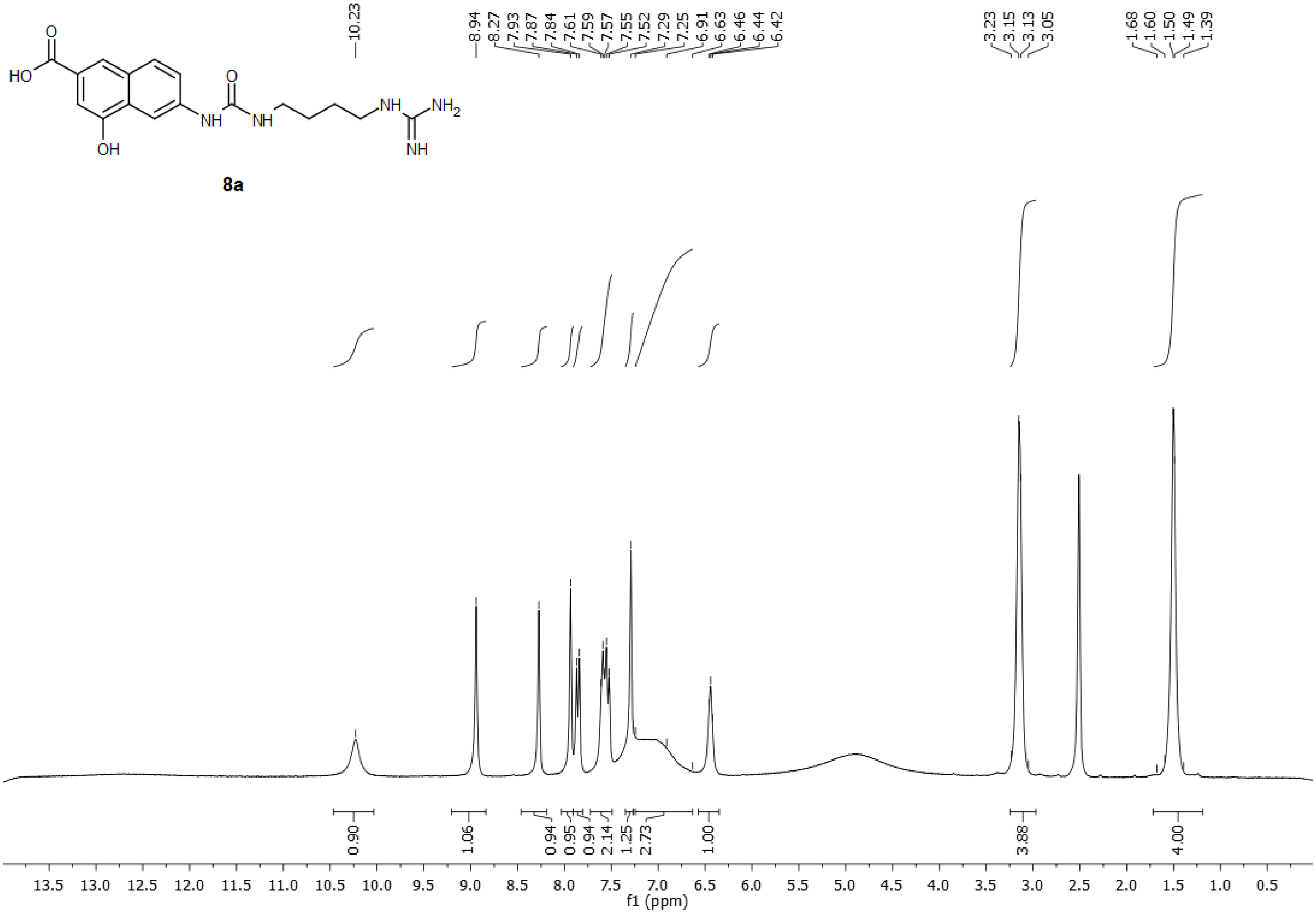

HO
O
NH
O
NH
NH
NH2
NH
OH
8a
10.23
8.94
8.27
7.93
7.87
7.84
7.61
7.59
7.57
7.55
7.52
7.29
7.25
6.91
6.63
6.46
6.44
6.42
3.23
3.15
3.13
3.05
1.68
1.60
1.50
1.49
1.39
0.90
1.06
0.94
0.95
0.94
2.14
1.25
2.73
1.00
3.88
4.00
13.5 13.0 12.5 12.0 11.5 11.0 10.5 10.0 9.5 9.0 8.5 8.0 7.5 7.0 6.5 6.0 5.5 5.0 4.5 4.0 3.5 3.0 2.5 2.0 1.5 1.0 0.5
f1 (ppm)

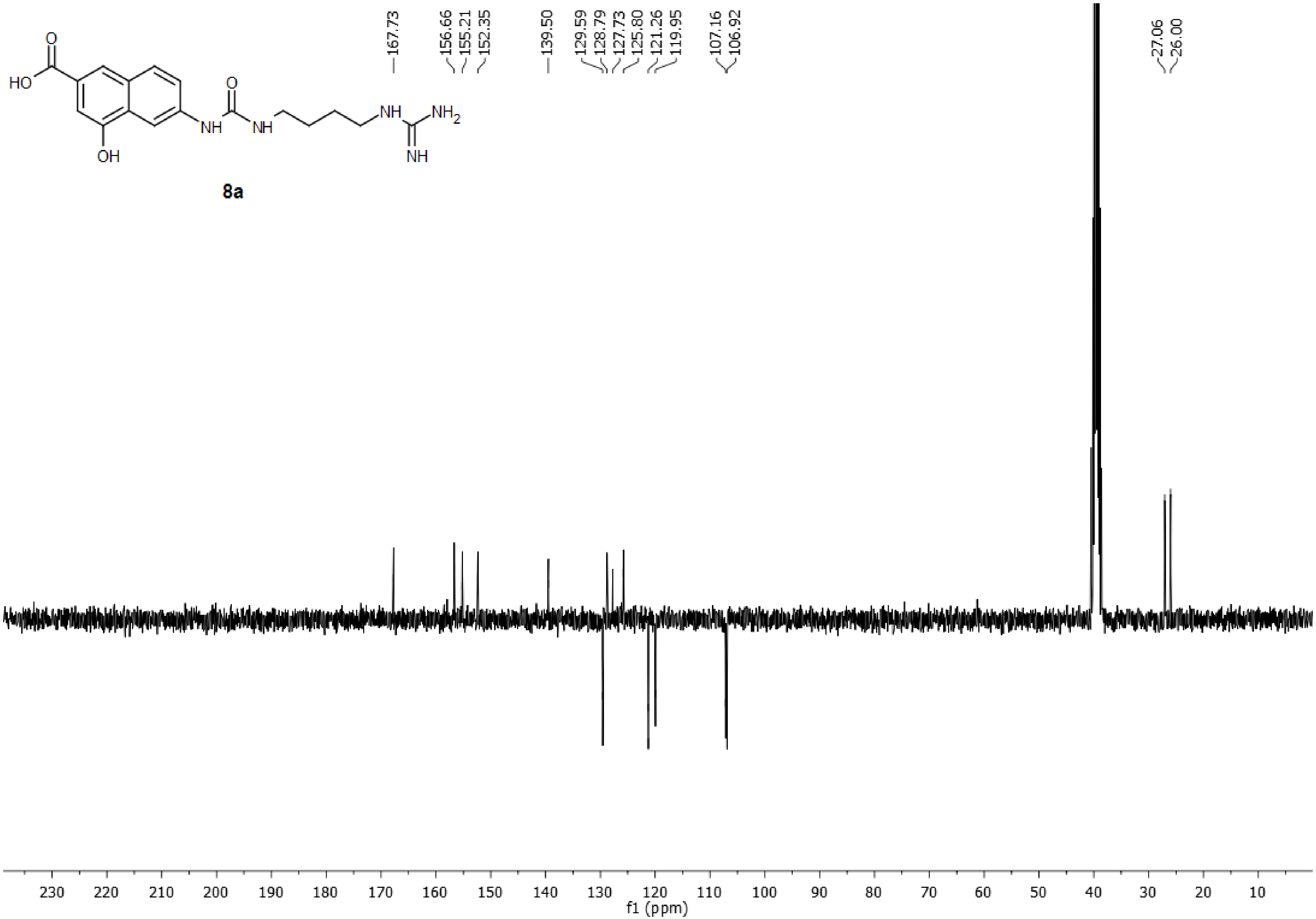

HO
O
O
NH
NH
NH
NH2
NH
OH
8a
167.73
156.66
155.21
152.35
139.50
129.59
128.79
127.73
125.80
121.26
119.95
107.16
106.92
27.06
26.00
230 220 210 200 190 180 170 160 150 140 130 120 110 100 90 80 70 60 50 40 30 20 10
f1 (ppm)

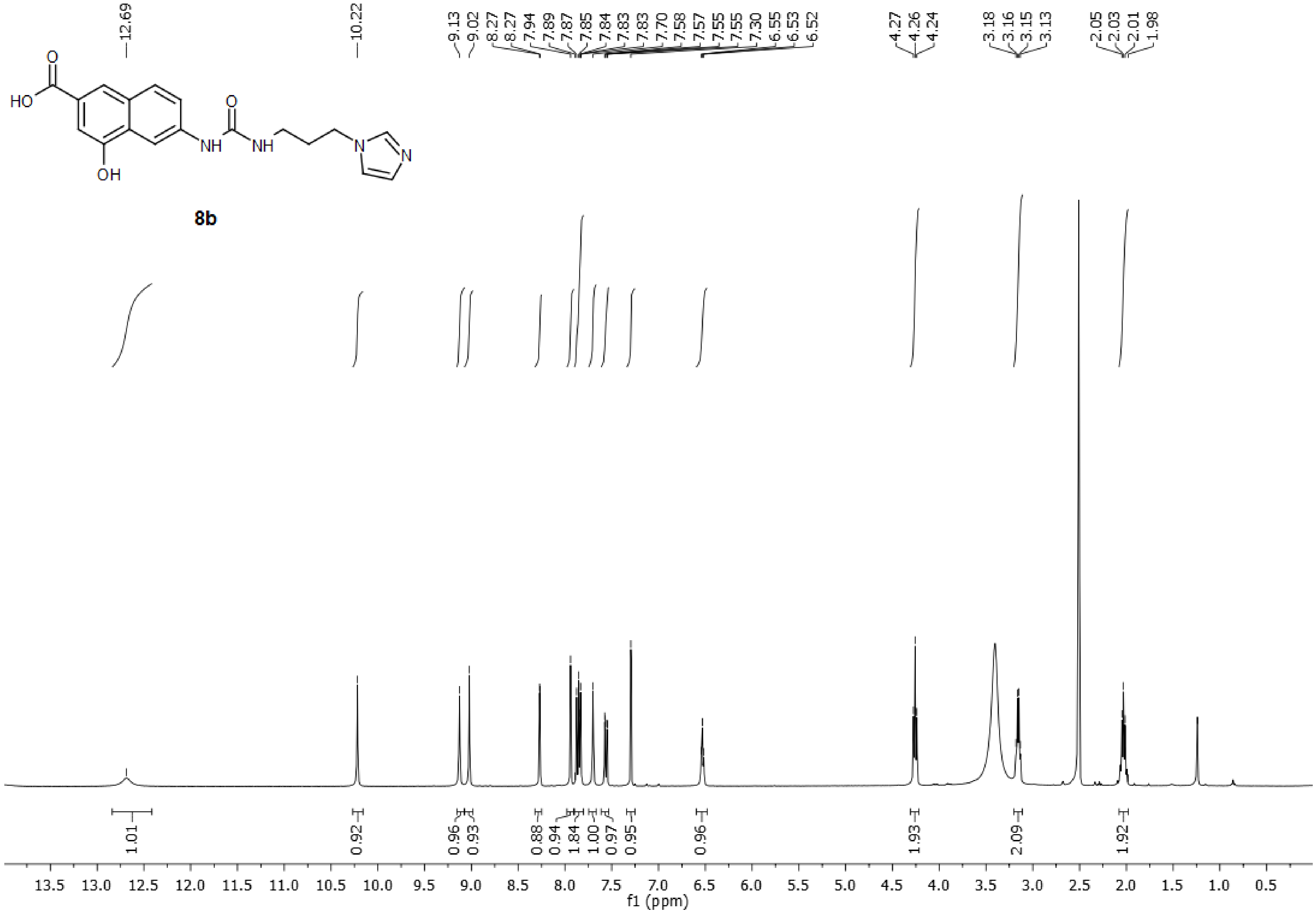

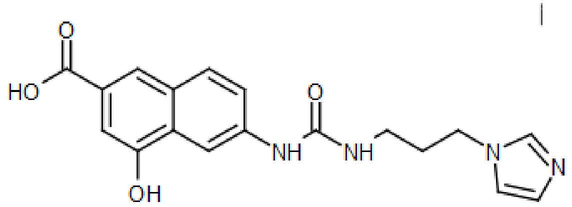

8b

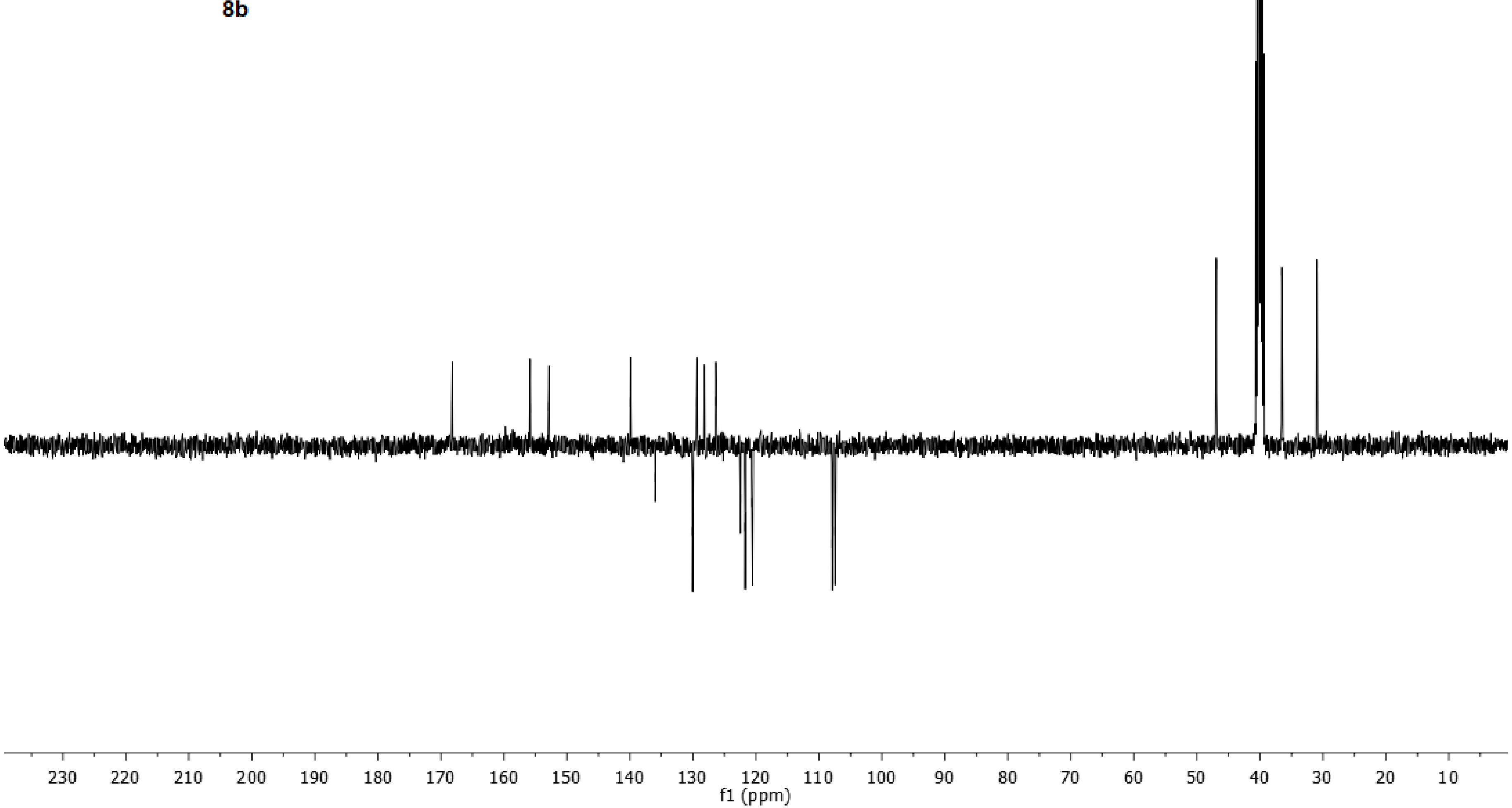

168.21
155.84
152.87
139.90
135.99
130.06
129.35
128.20
126.36
122.47
121.74
120.65
120.54
107.86
107.42
46.92
36.51
30.94
230
220
210
200
190
180
170
160
150
140
130
120
110
100
90
80
70
60
50
40
30
20
10
f1 (ppm)

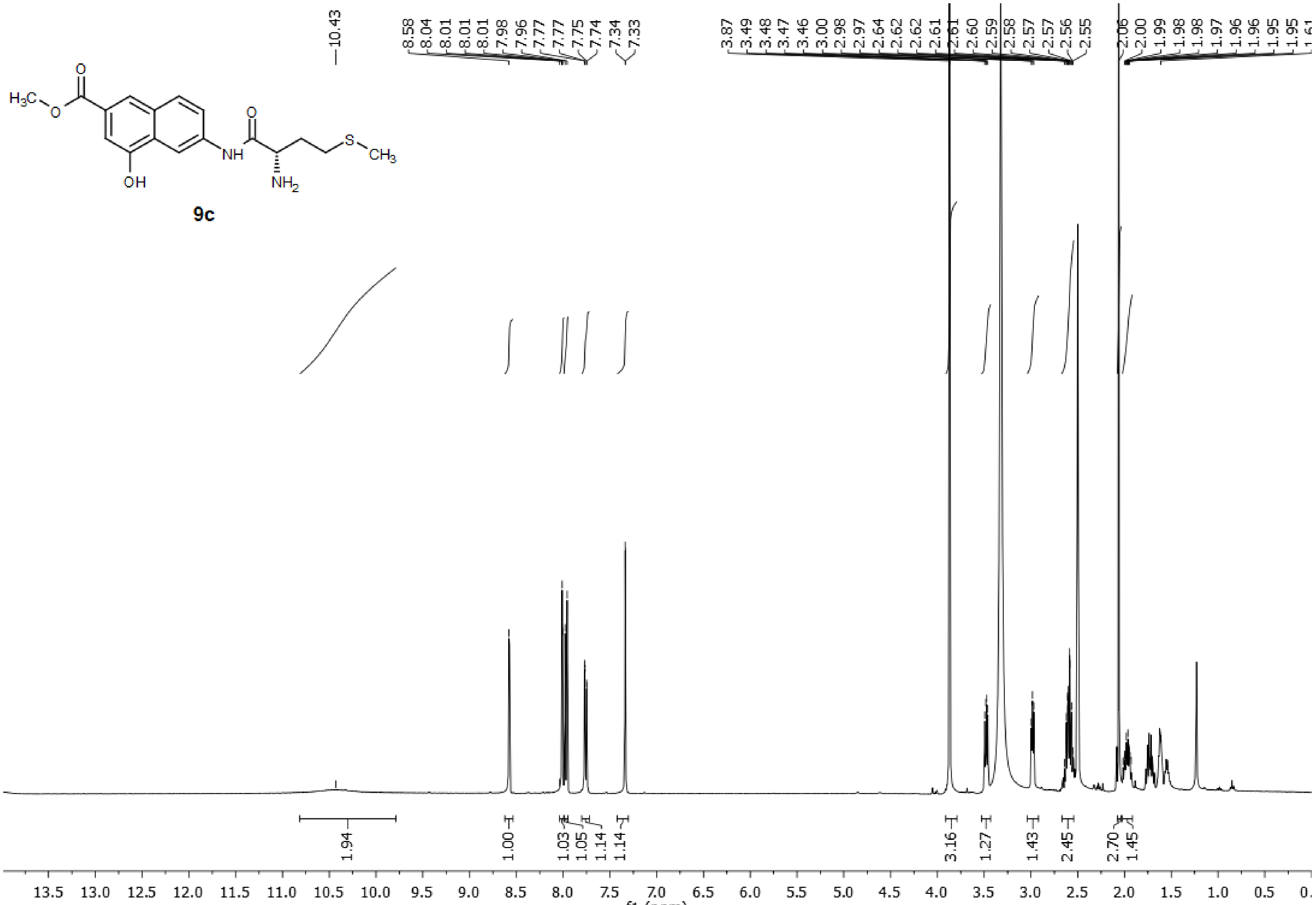
9c
10.43
8.58
8.04
8.01
8.01
8.01
7.98
7.96
7.77
7.77
7.75
7.74
7.34
7.33
3.87
3.49
3.48
3.47
3.46
3.00
2.98
2.97
2.64
2.62
2.62
2.61
2.61
2.60
2.59
2.58
2.57
2.57
2.56
2.55
2.06
2.00
1.99
1.98
1.98
1.97
1.96
1.96
1.95
1.95
1.94
1.00
1.03
1.05
1.14
1.14
3.16
1.27
1.43
2.45
2.70
1.45
f1 (ppm)

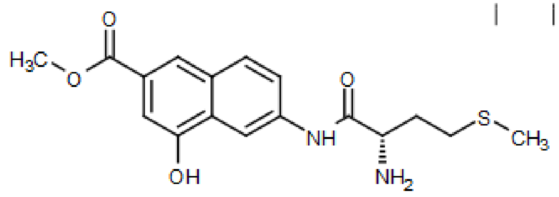

H3C
O
O
NH
O
S
CH3
OH
NH2

9c

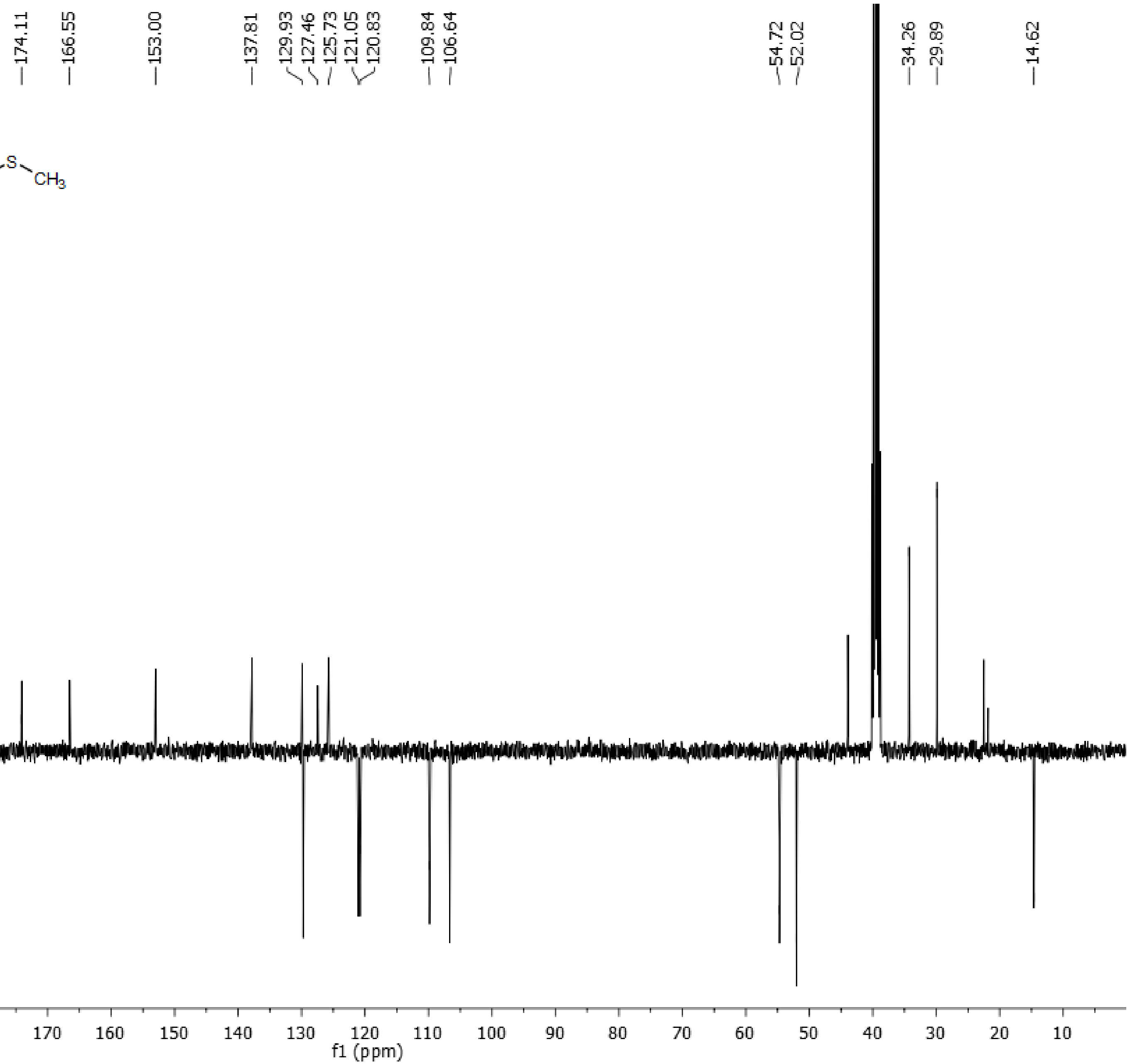

174.11
166.55
153.00
137.81
129.93
127.46
125.73
121.05
120.83
109.84
106.64
54.72
52.02
34.26
29.89
14.62
230 220 210 200 190 180 170 160 150 140 130 120 110 100 90 80 70 60 50 40 30 20 10
f1 (ppm)

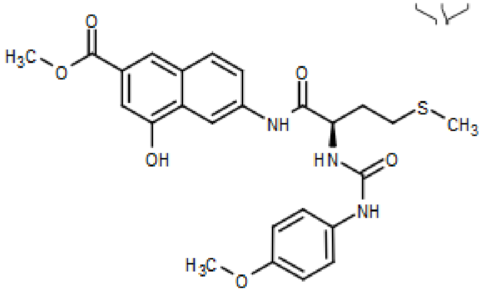

9d

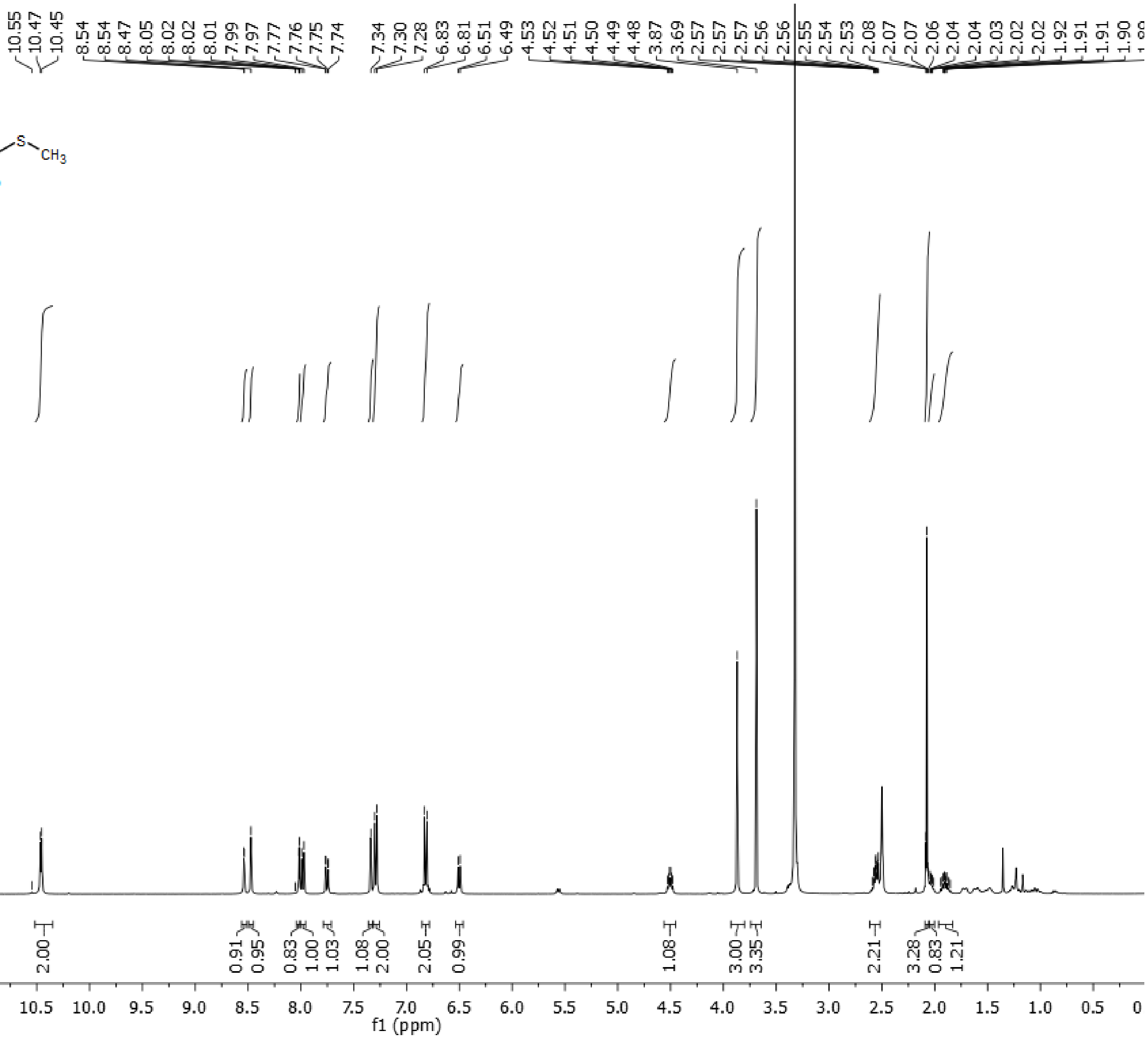

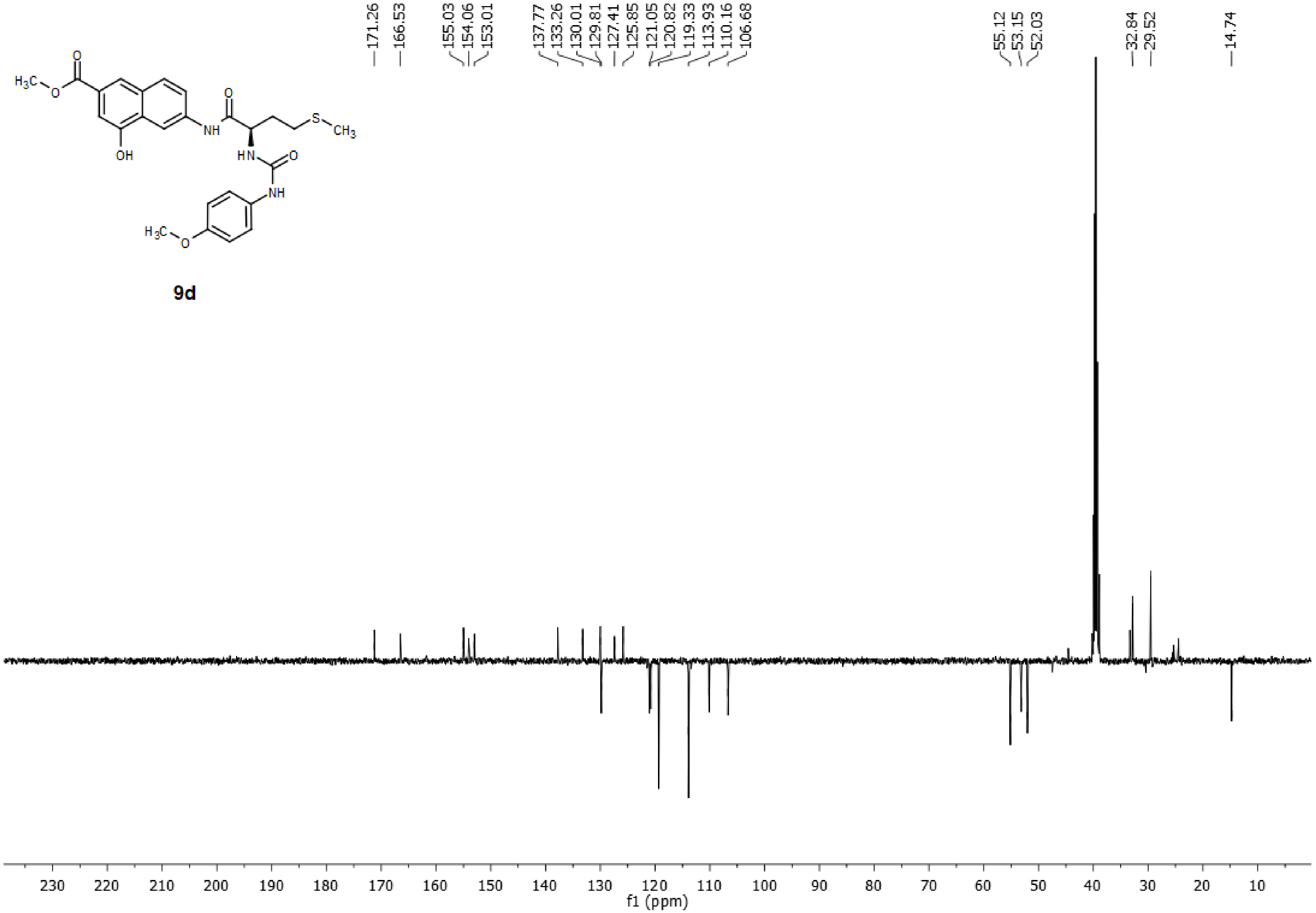
171.26
166.53
155.03
154.06
153.01
137.77
133.26
130.01
129.81
127.41
125.85
121.05
120.82
119.33
113.93
110.16
106.68
55.12
53.15
52.03
32.84
29.52
14.74
f1 (ppm)

9d

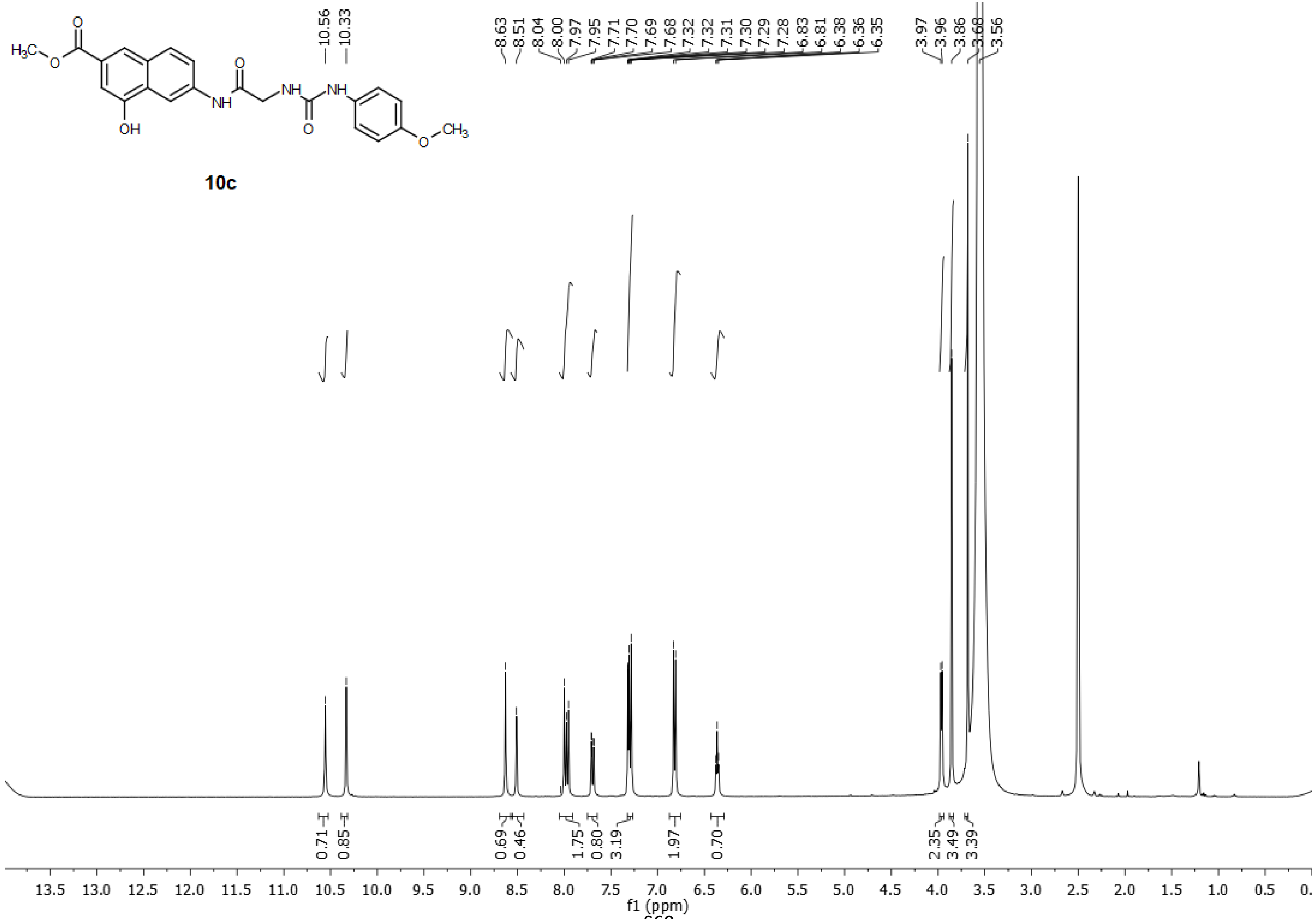

10c
H3C O O NH O NH NH O OH O CH3
10.56
10.33
8.63
8.51
8.04
8.00
7.97
7.95
7.71
7.70
7.69
7.68
7.32
7.32
7.31
7.30
7.29
7.28
6.83
6.81
6.38
6.36
6.35
3.97
3.96
3.86
3.68
3.56
0.71
0.85
0.69
0.46
1.75
0.80
3.19
1.97
0.70
2.35
3.49
3.39
13.5 13.0 12.5 12.0 11.5 11.0 10.5 10.0 9.5 9.0 8.5 8.0 7.5 7.0 6.5 6.0 5.5 5.0 4.5 4.0 3.5 3.0 2.5 2.0 1.5 1.0 0.5 0.
f1 (ppm)

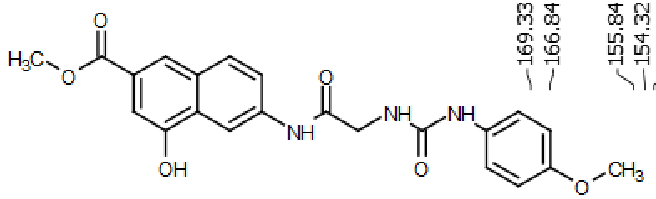

H3C
O
O
NH
O
NH
NH
O
O
CH3
OH

10c

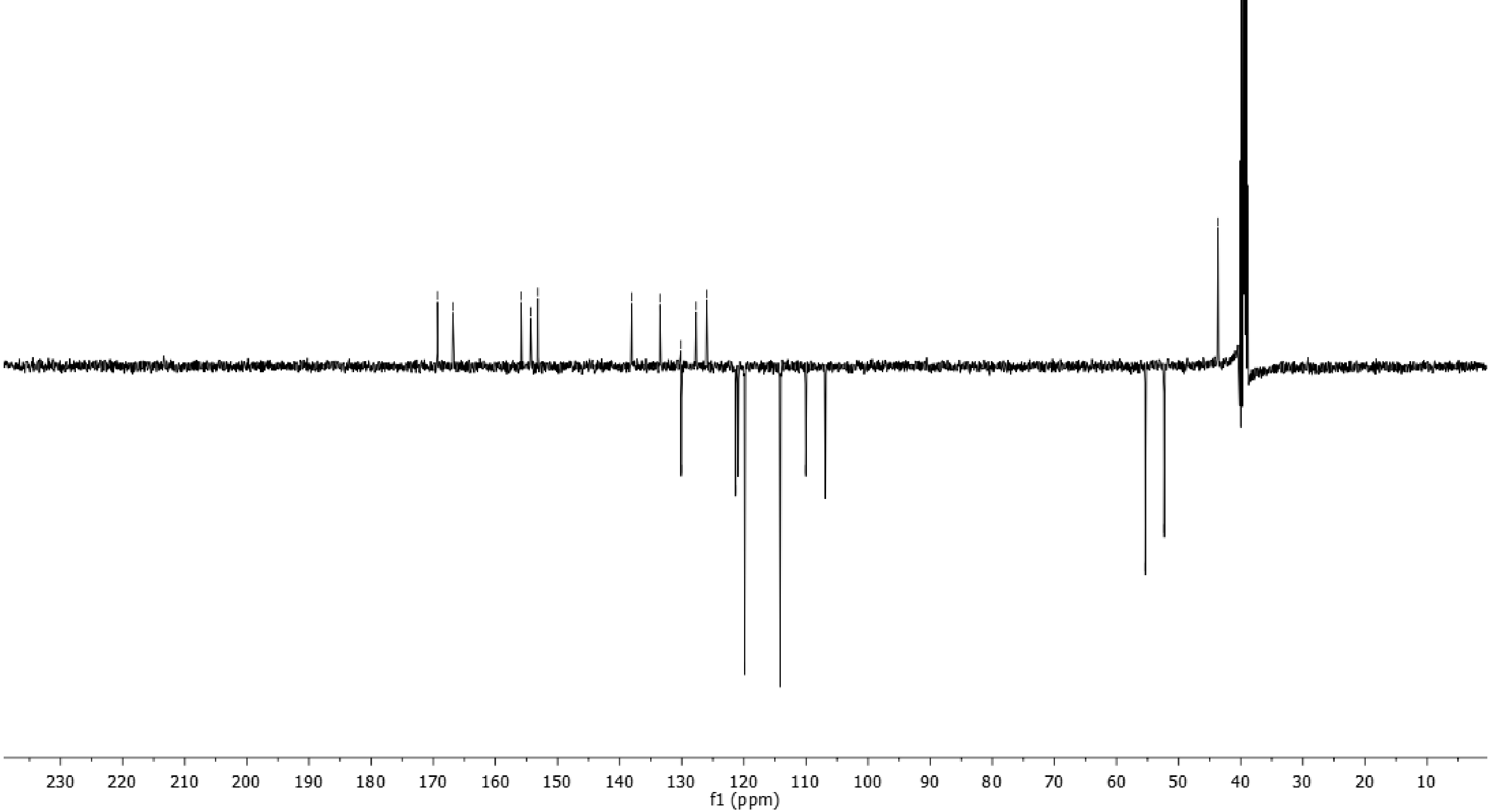

169.33
166.84
155.84
154.32
153.16
138.05
133.50
130.12
130.09
127.70
125.99
121.33
120.91
119.84
114.14
110.03
106.87
55.36
52.30
43.65
230
220
210
200
190
180
170
160
150
140
130
120
110
100
90
80
70
60
50
40
30
20
10
f1 (ppm)

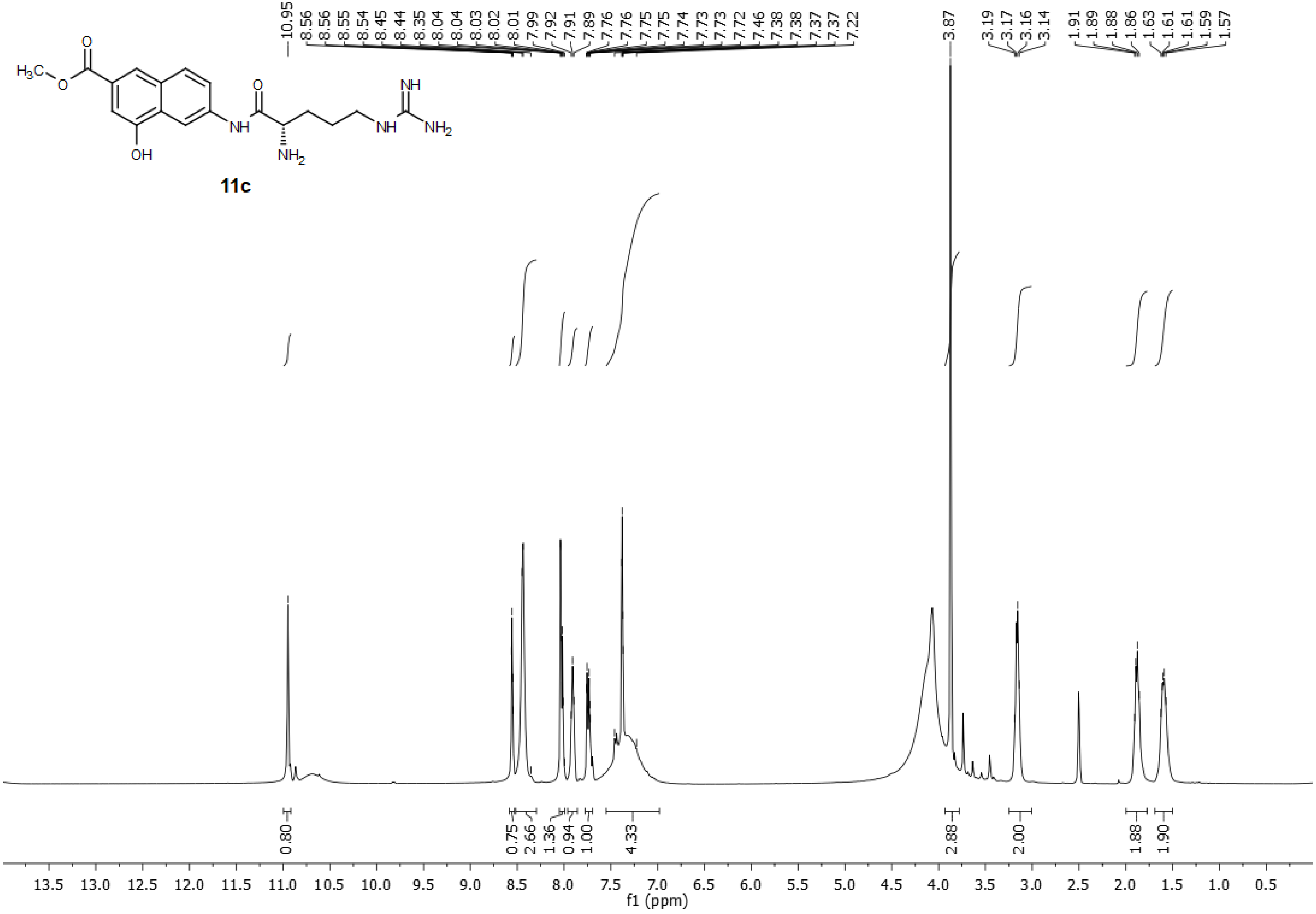
11c
f1 (ppm)

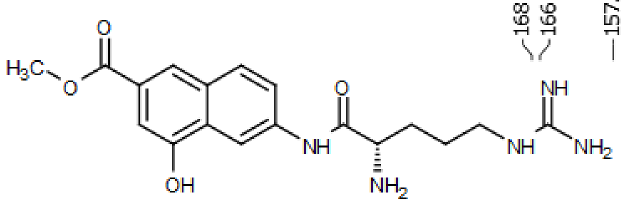

H3C
O
O
OH
NH
O
NH2
NH
NH
NH2

11c

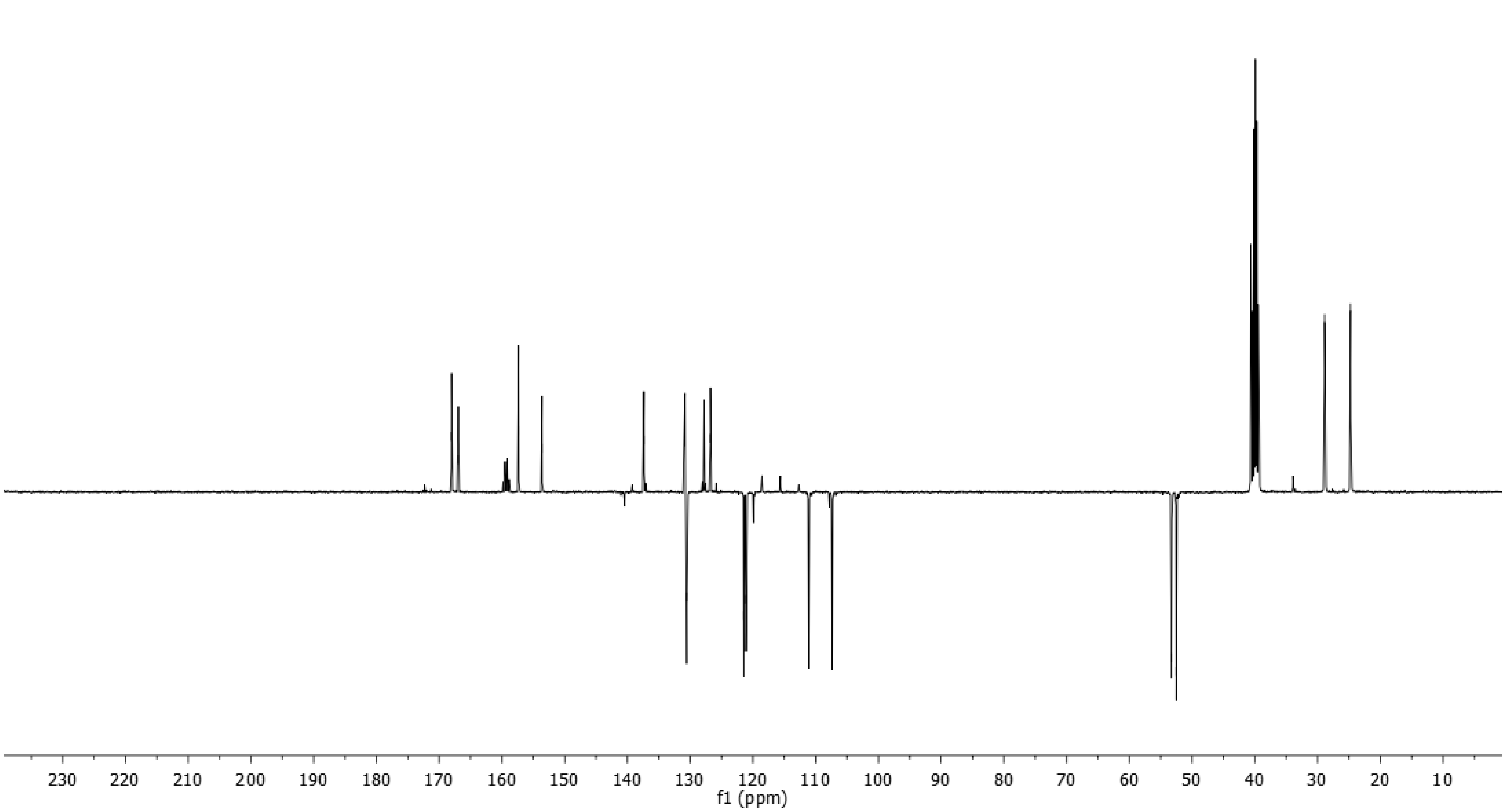

168.05
166.99
157.38
153.63
137.38
130.83
130.55
127.80
126.79
121.45
121.09
111.11
107.36
53.32
52.53
40.61
28.87
24.74
230 220 210 200 190 180 170 160 150 140 130 120 110 100 90 80 70 60 50 40 30 20 10
f1 (ppm)

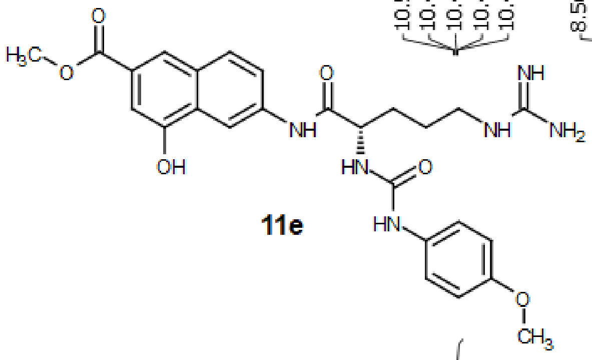

11e

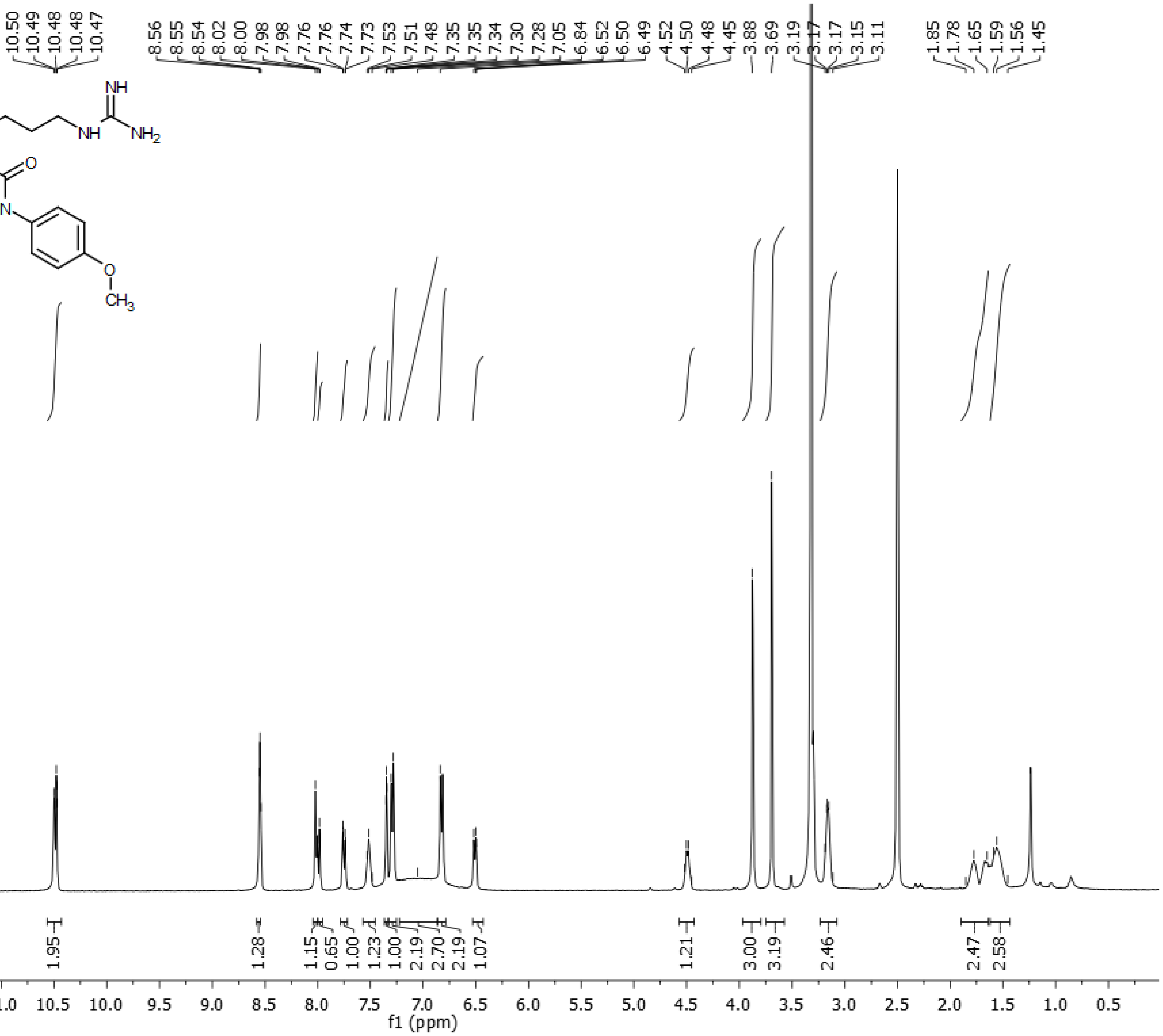

10.50
10.49
10.48
10.48
10.47
8.56
8.55
8.54
8.02
8.00
7.98
7.98
7.76
7.76
7.74
7.73
7.53
7.51
7.48
7.35
7.35
7.34
7.30
7.28
7.05
6.84
6.52
6.50
6.49
4.52
4.50
4.48
4.45
3.88
3.69
3.19
3.17
3.17
3.15
3.11
1.85
1.78
1.65
1.59
1.56
1.45
1.95
1.28
1.15
0.65
1.00
1.23
1.00
2.19
2.70
2.19
1.07
1.21
3.00
3.19
2.46
2.47
2.58
13.5
13.0
12.5
12.0
11.5
11.0
10.5
10.0
9.5
9.0
8.5
8.0
7.5
7.0
6.5
6.0
5.5
5.0
4.5
4.0
3.5
3.0
2.5
2.0
1.5
1.0
0.5
f1 (ppm)

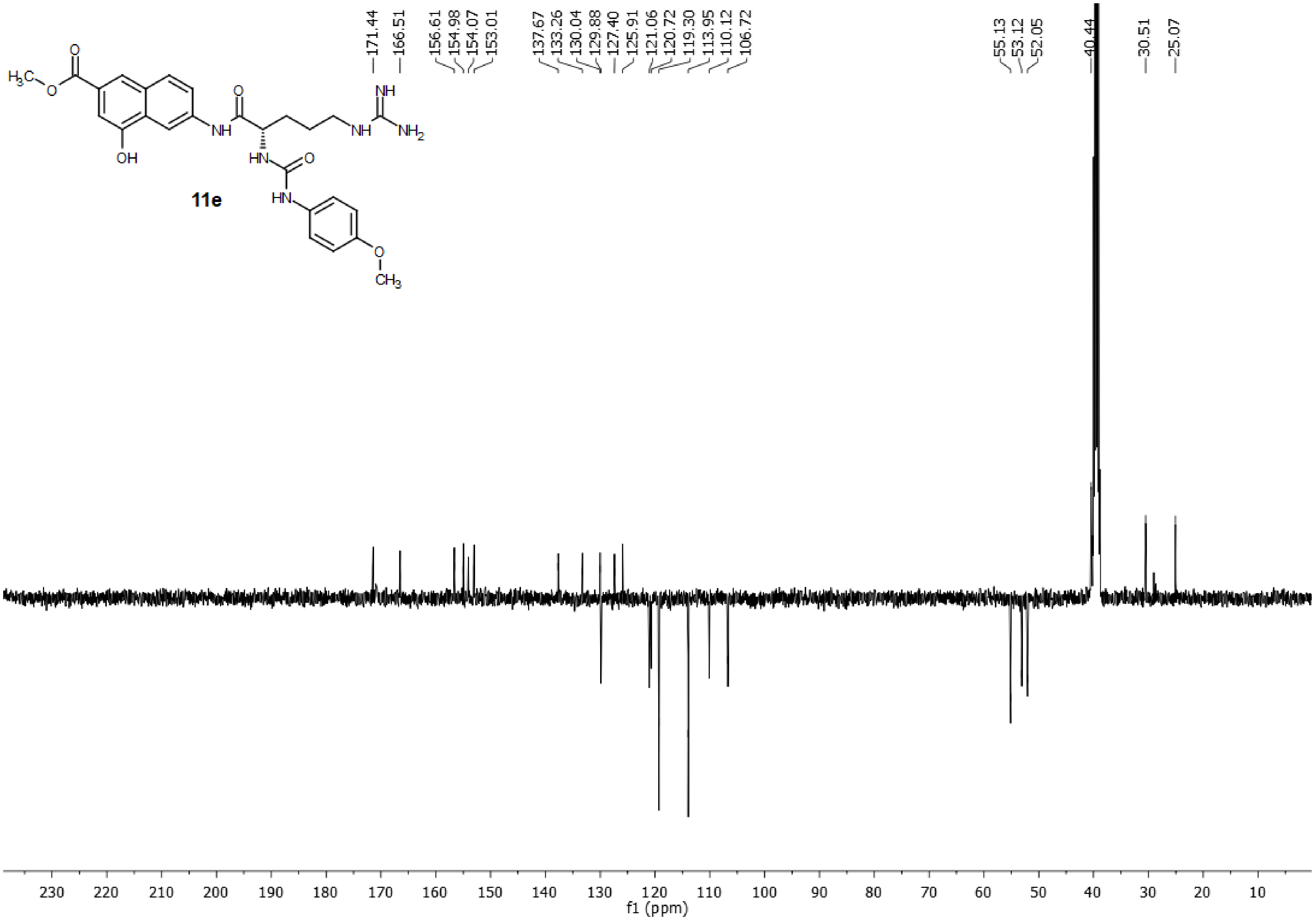
11e
171.44
166.51
156.61
154.98
154.07
153.01
137.67
133.26
130.04
129.88
127.40
125.91
121.06
120.72
119.30
113.95
110.12
106.72
55.13
53.12
52.05
40.44
30.51
25.07
f1 (ppm)

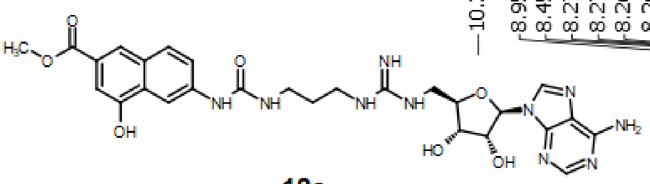

12a

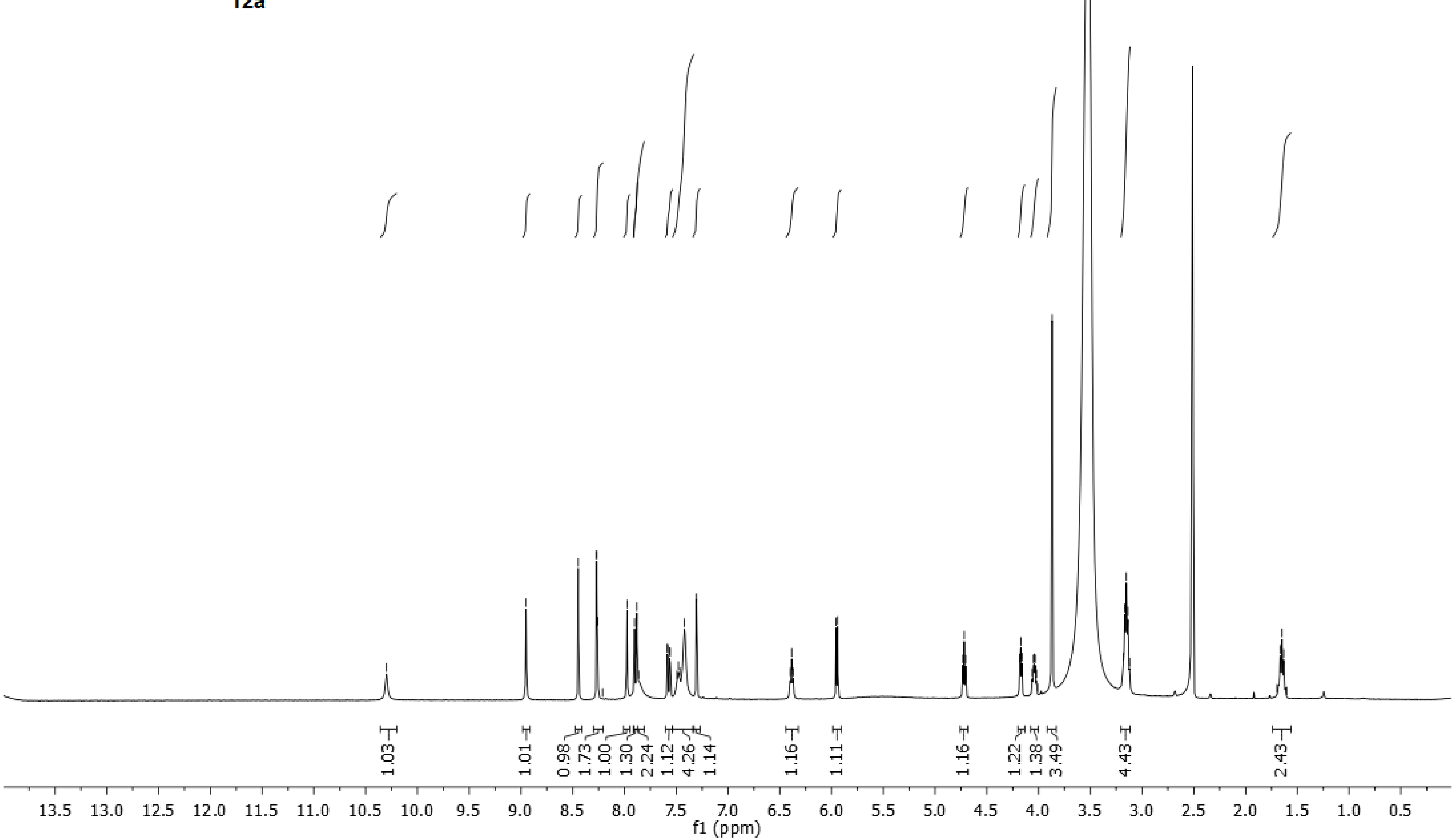

10.30
8.95
8.45
8.27
8.27
8.26
8.21
7.98
7.90
7.88
7.86
7.59
7.58
7.56
7.56
7.49
7.48
7.47
7.42
7.30
6.40
6.39
6.37
5.96
5.94
4.73
4.72
4.70
4.18
4.17
4.16
4.06
4.05
4.04
4.03
4.02
4.02
4.01
3.87
3.18
3.17
3.15
3.14
3.12
1.70
1.67
1.65
1.63
1.60
1.03
1.01
0.98
1.73
1.00
1.30
2.24
1.12
4.26
1.14
1.16
1.11
1.16
1.22
1.38
3.49
4.43
2.43
13.5
13.0
12.5
12.0
11.5
11.0
10.5
10.0
9.5
9.0
8.5
8.0
7.5
7.0
6.5
6.0
5.5
5.0
4.5
4.0
3.5
3.0
2.5
2.0
1.5
1.0
0.5
f1 (ppm)

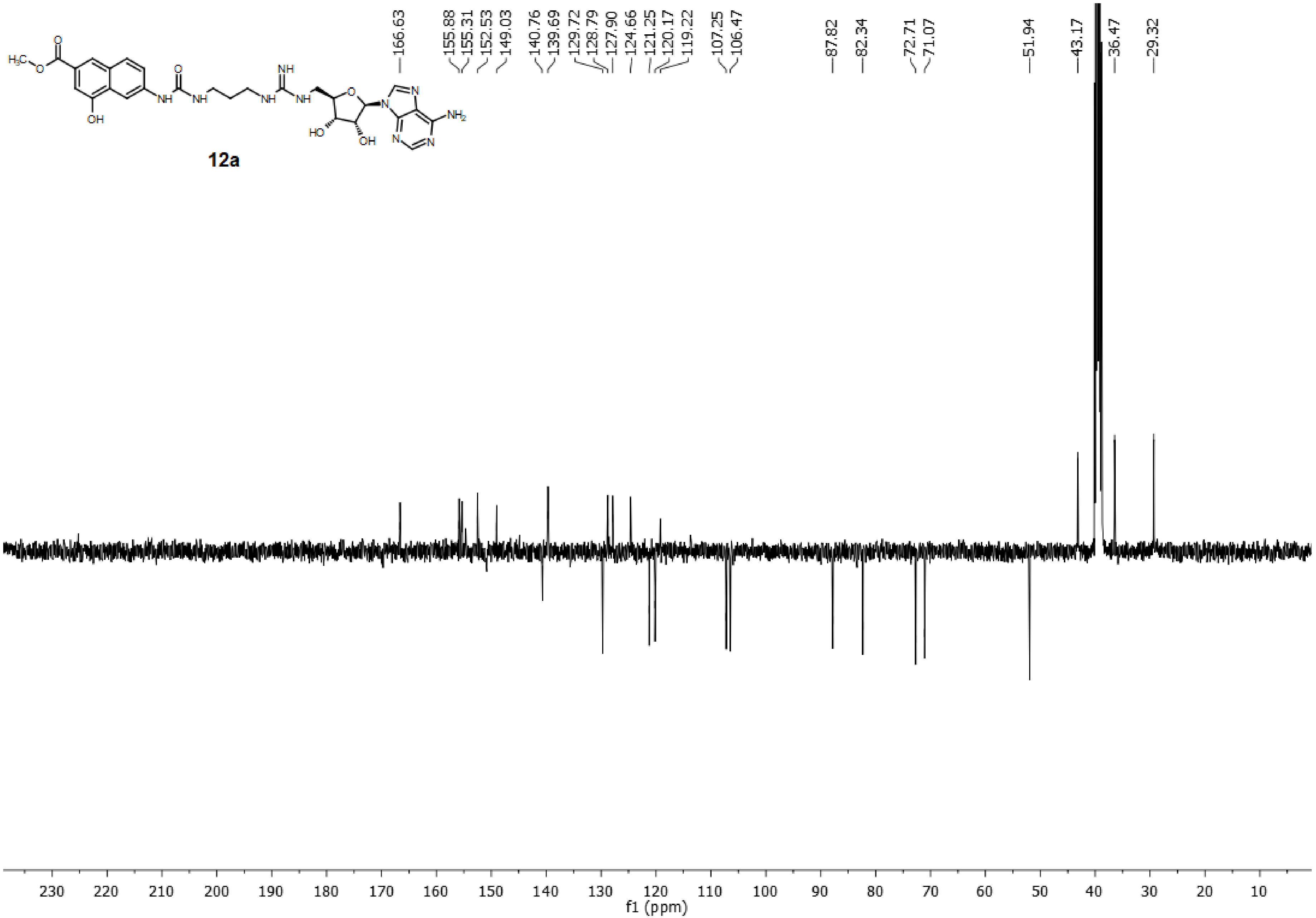
12a
166.63
155.88
155.31
152.53
149.03
140.76
139.69
129.72
128.79
127.90
124.66
121.25
120.17
119.22
107.25
106.47
87.82
82.34
72.71
71.07
51.94
43.17
36.47
29.32
230 220 210 200 190 180 170 160 150 140 130 120 110 100 90 80 70 60 50 40 30 20 10
f1 (ppm)

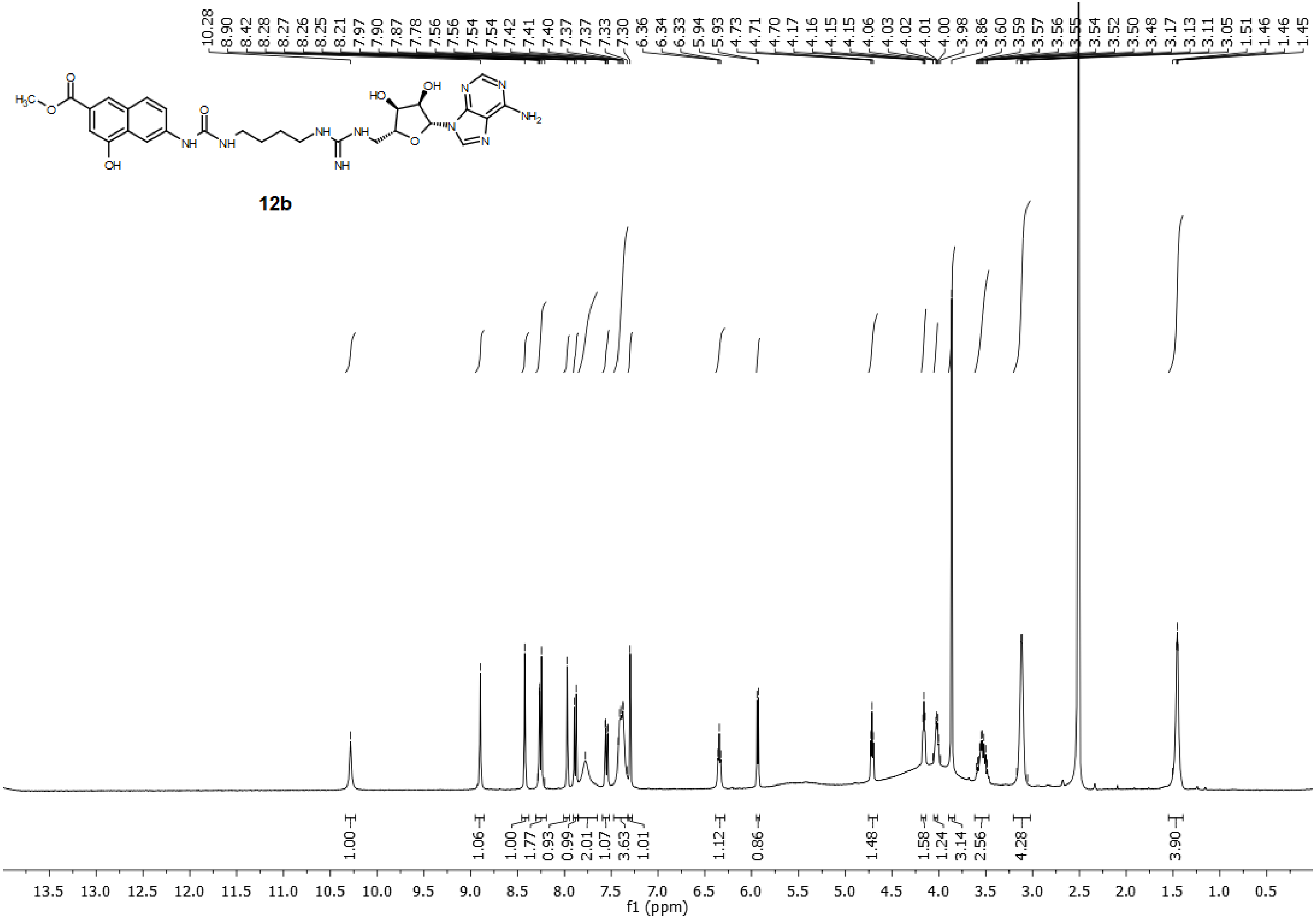
12b
f1 (ppm)

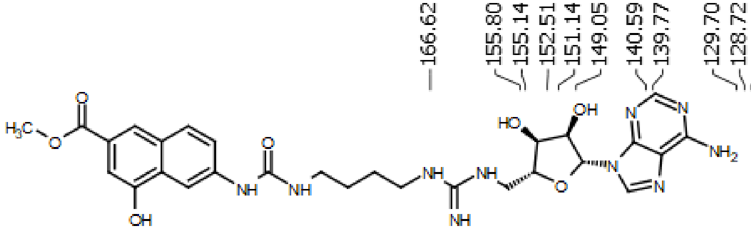

12b

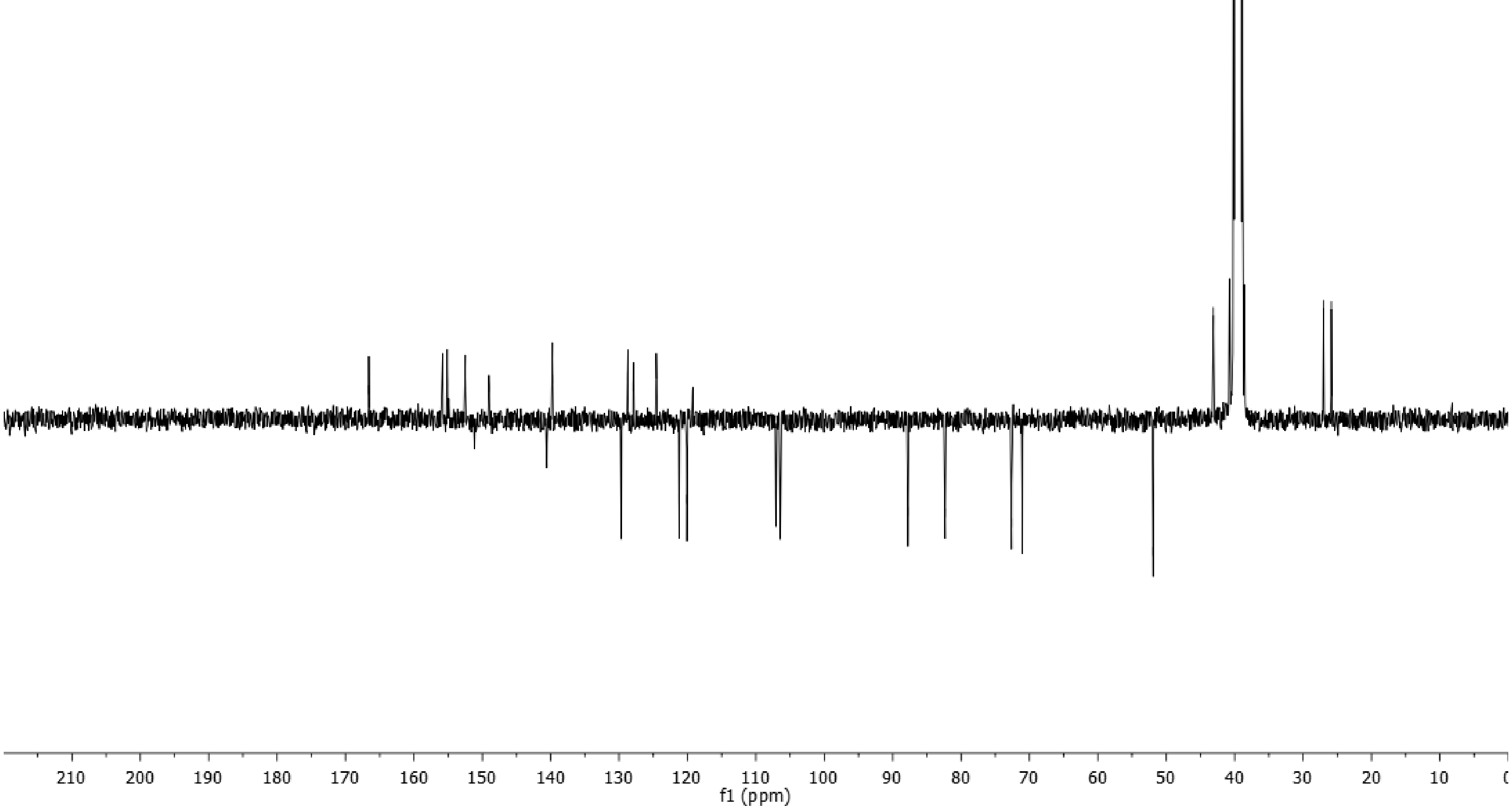

166.62
155.80
155.14
152.51
151.14
149.05
140.59
139.77
129.70
128.72
127.91
121.23
120.09
119.22
107.08
106.45
87.79
82.34
72.64
71.05
51.92
43.12
26.99
25.88
210
200
190
180
170
160
150
140
130
120
110
100
90
80
70
60
50
40
30
20
10
f1 (ppm)

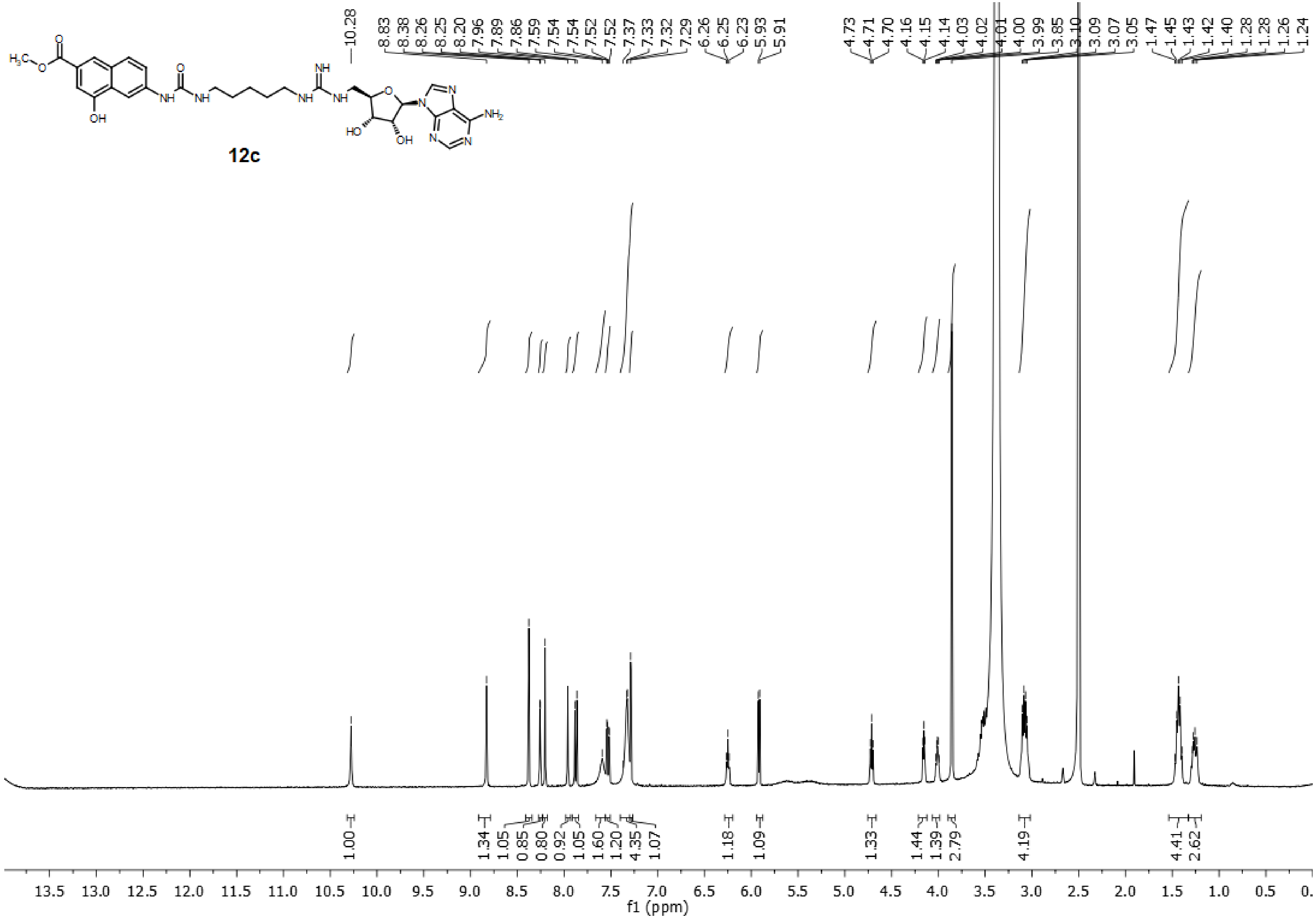
12c
f1 (ppm)

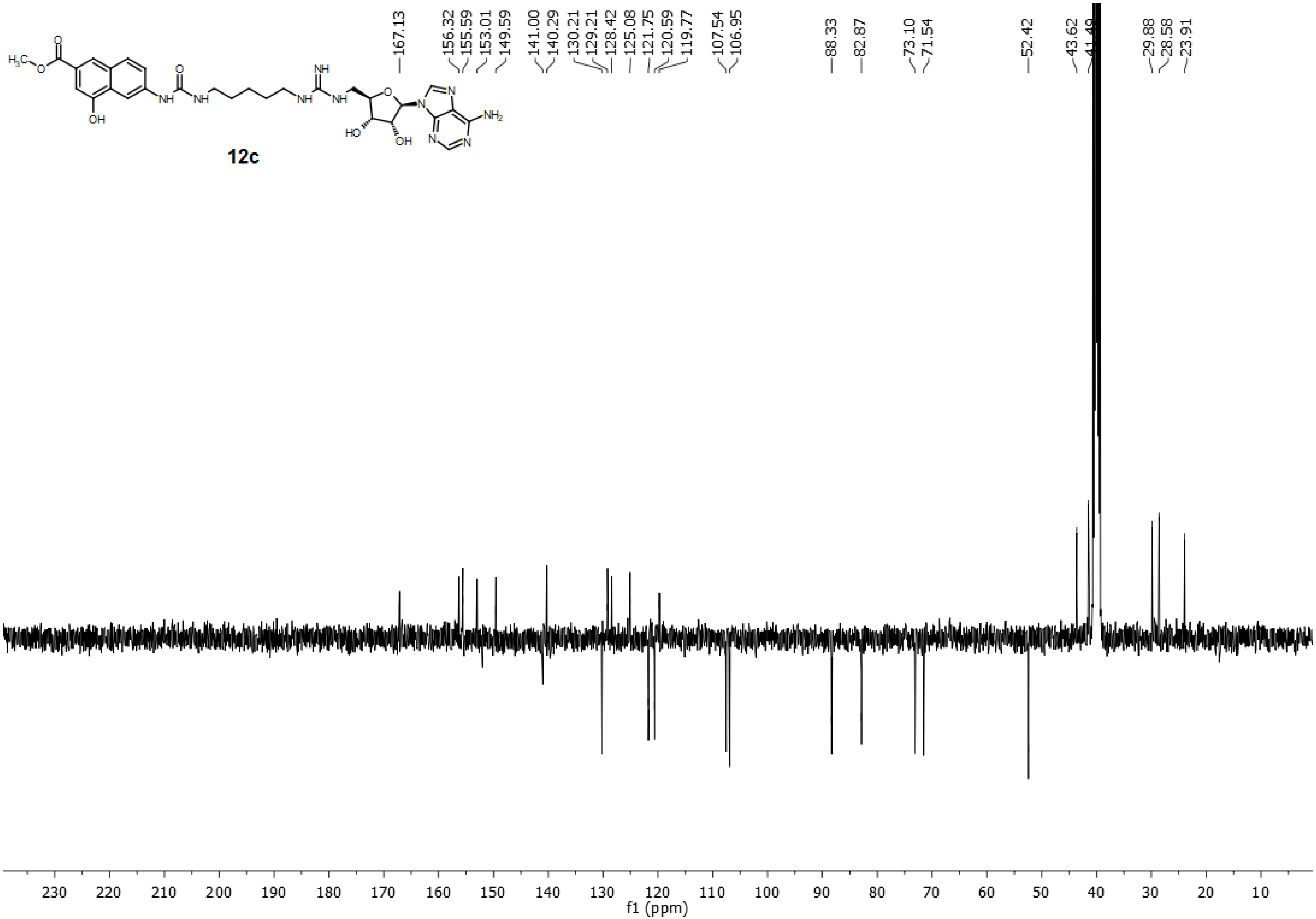
12c
167.13
156.32
155.59
153.01
149.59
141.00
140.29
130.21
129.21
128.42
125.08
121.75
120.59
119.77
107.54
106.95
88.33
82.87
73.10
71.54
52.42
43.62
41.49
29.88
28.58
23.91
230
220
210
200
190
180
170
160
150
140
130
120
110
100
90
80
70
60
50
40
30
20
10
f1 (ppm)

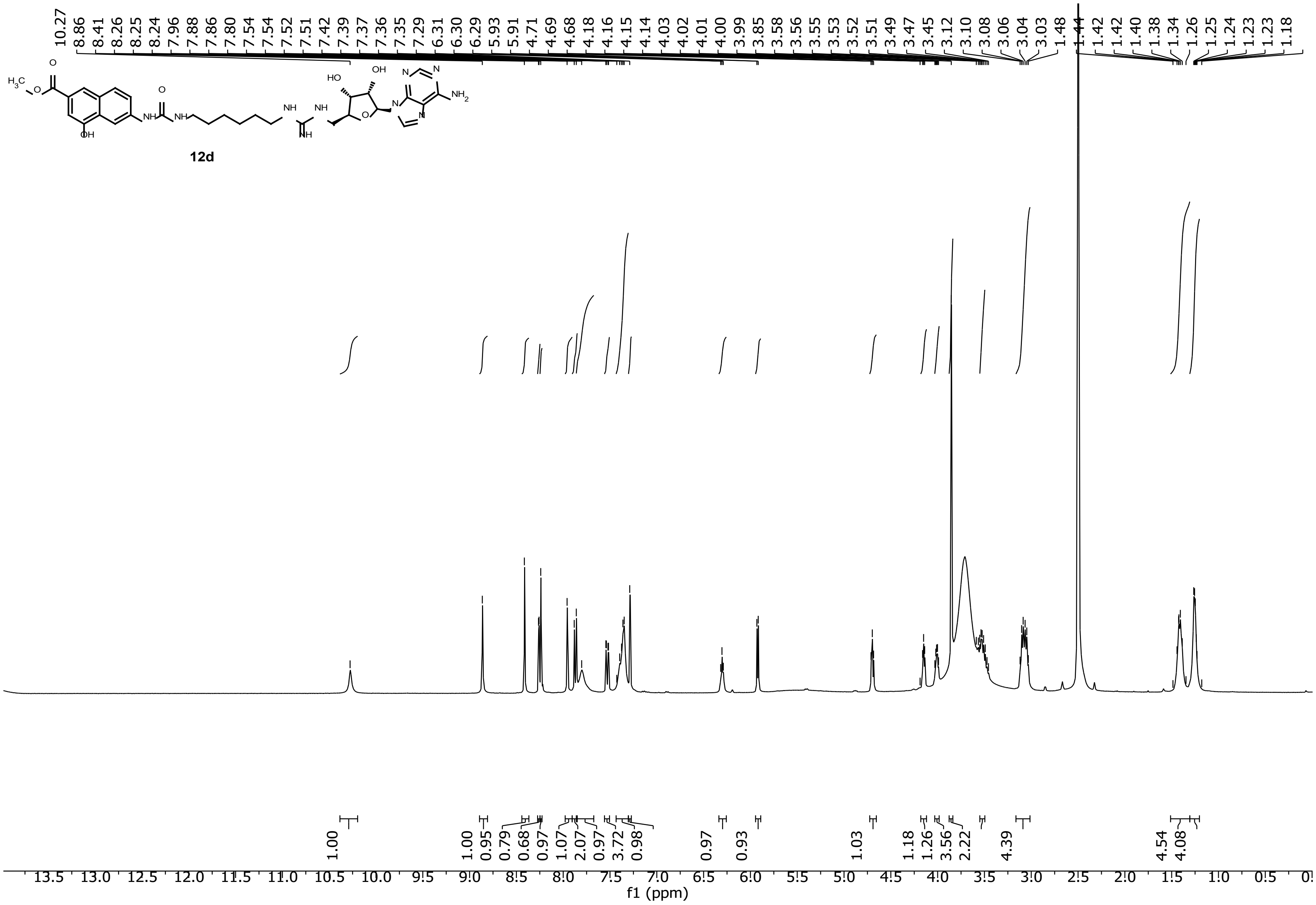

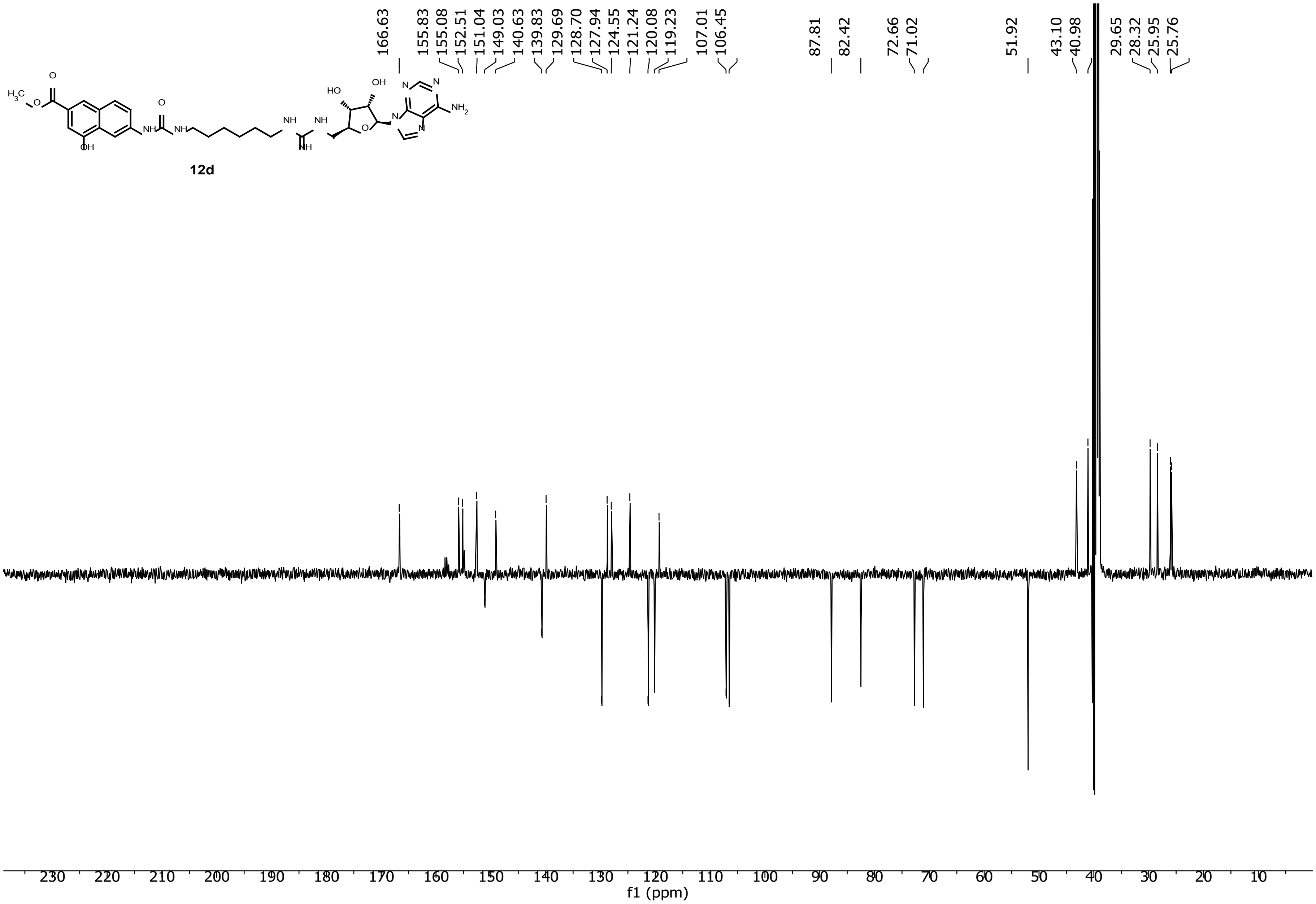

166.63
155.83
155.08
152.51
151.04
149.03
140.63
139.83
129.69
128.70
127.94
124.55
121.24
120.08
119.23
107.01
106.45
87.81
82.42
72.66
71.02
51.92
43.10
40.98
29.65
28.32
25.95
25.76
12d
230
220
210
200
190
180
170
160
150
140
130
120
110
100
90
80
70
60
50
40
30
20
10
f1 (ppm)

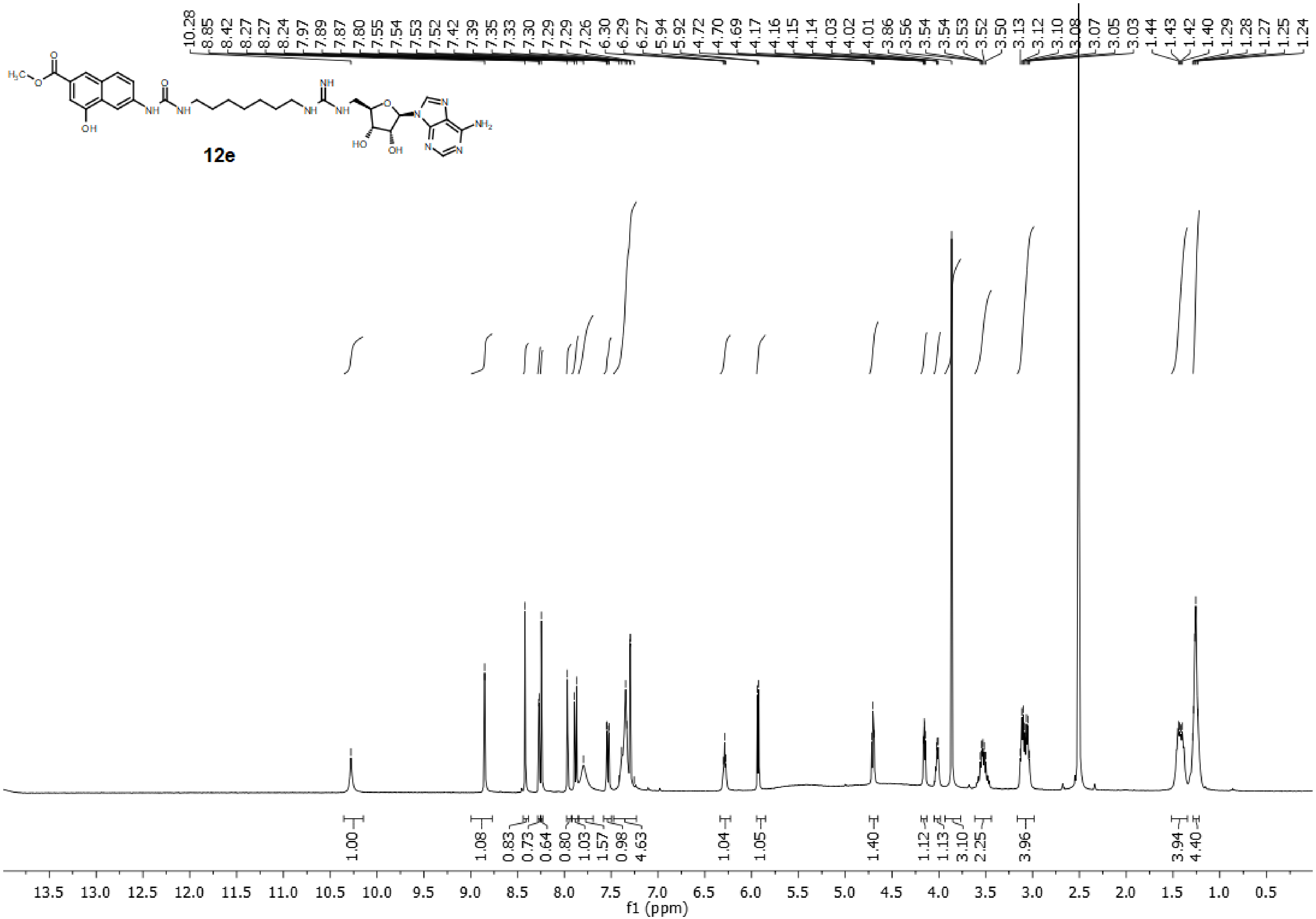
10.28 8.85 8.42 8.27 8.27 8.24 7.97 7.89 7.87 7.80 7.55 7.54 7.53 7.52 7.42 7.39 7.35 7.33 7.30 7.29 7.29 7.26 6.30 6.29 6.27 5.94 5.92 4.72 4.70 4.69 4.17 4.16 4.15 4.14 4.03 4.02 4.01 3.86 3.56 3.54 3.54 3.53 3.52 3.50 3.13 3.12 3.10 3.08 3.07 3.05 3.03 1.44 1.43 1.42 1.40 1.29 1.28 1.27 1.25 1.24
12e
1.00 1.08 0.83 0.73 0.64 0.80 1.03 1.57 0.98 4.63 1.04 1.05 1.40 1.12 1.13 3.10 2.25 3.96 3.94 4.40
13.5 13.0 12.5 12.0 11.5 11.0 10.5 10.0 9.5 9.0 8.5 8.0 7.5 7.0 6.5 6.0 5.5 5.0 4.5 4.0 3.5 3.0 2.5 2.0 1.5 1.0 0.5
f1 (ppm)

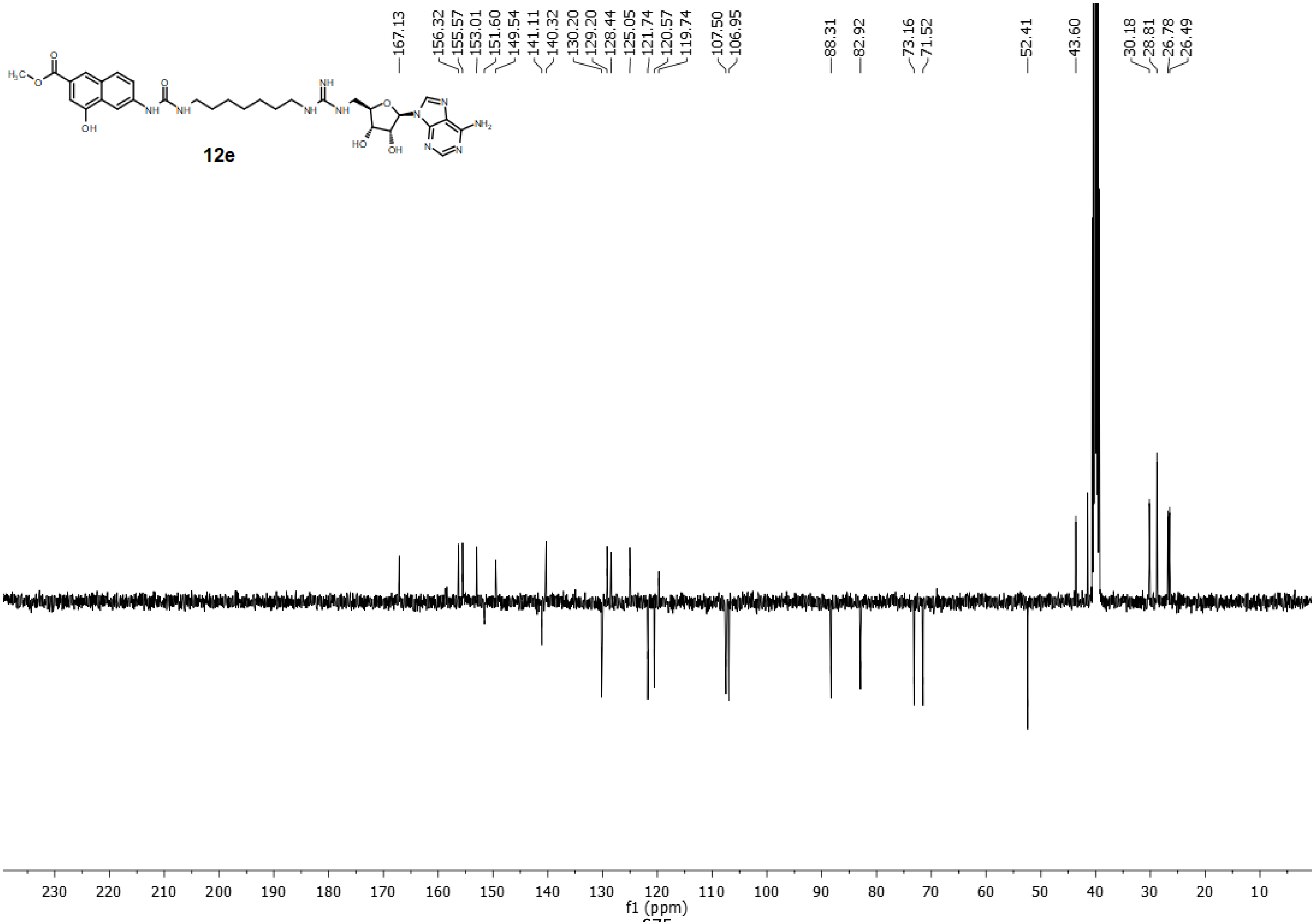
12e
167.13
156.32
155.57
153.01
151.60
149.54
141.11
140.32
130.20
129.20
128.44
125.05
121.74
120.57
119.74
107.50
106.95
88.31
82.92
73.16
71.52
52.41
43.60
30.18
28.81
26.78
26.49
f1 (ppm)

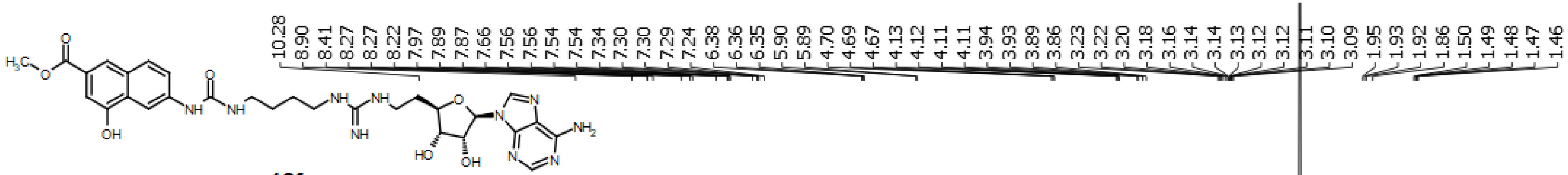

12f

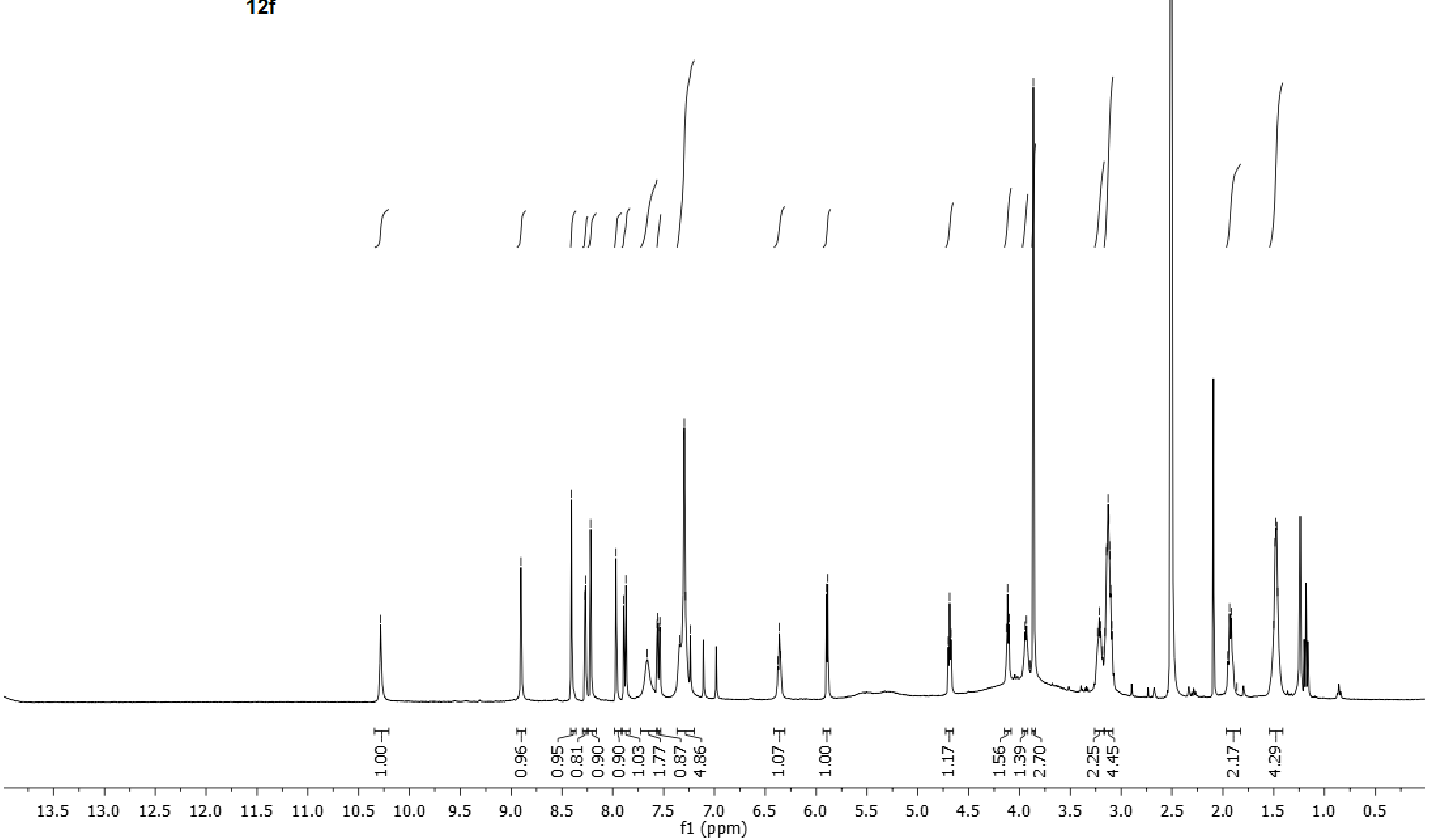

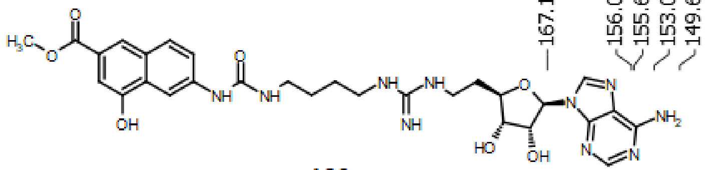

12f

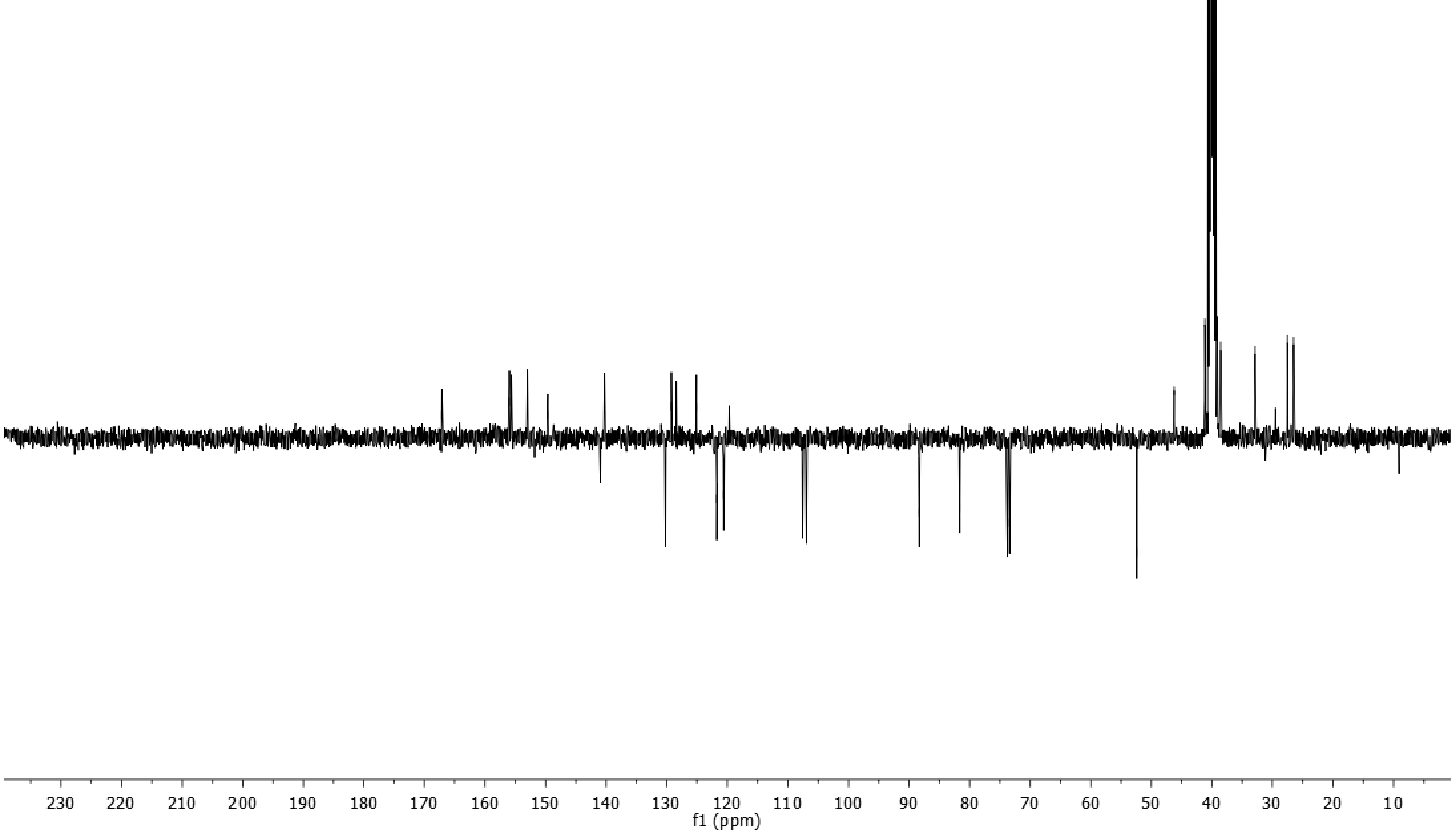

167.12
156.00
155.66
153.01
149.65
140.96
140.28
130.20
129.22
128.42
125.09
121.74
120.60
119.68
107.59
106.95
88.33
81.64
73.77
73.40
52.42
46.24
41.15
32.85
27.52
26.47
230 220 210 200 190 180 170 160 150 140 130 120 110 100 90 80 70 60 50 40 30 20 10
f1 (ppm)

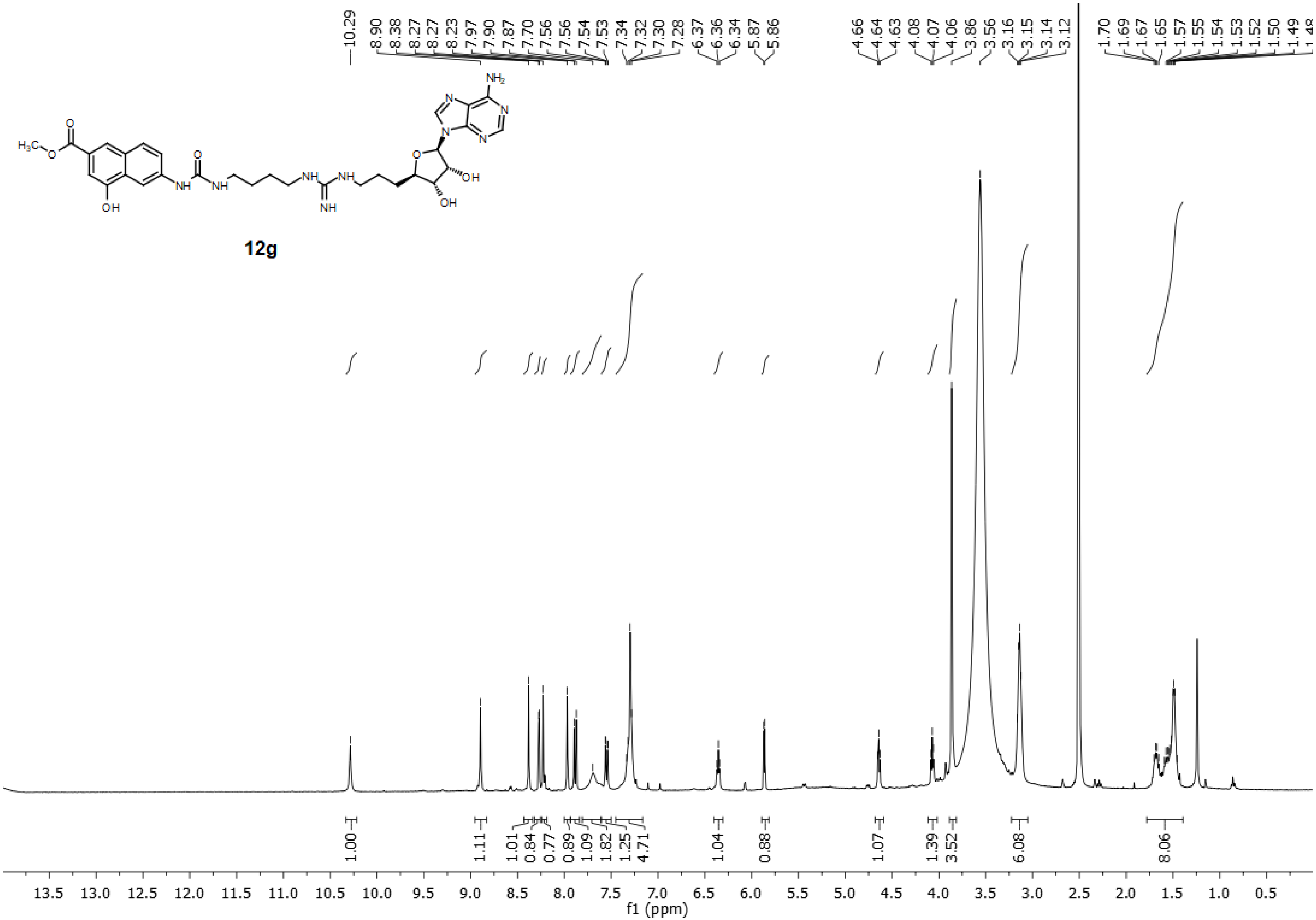

12g

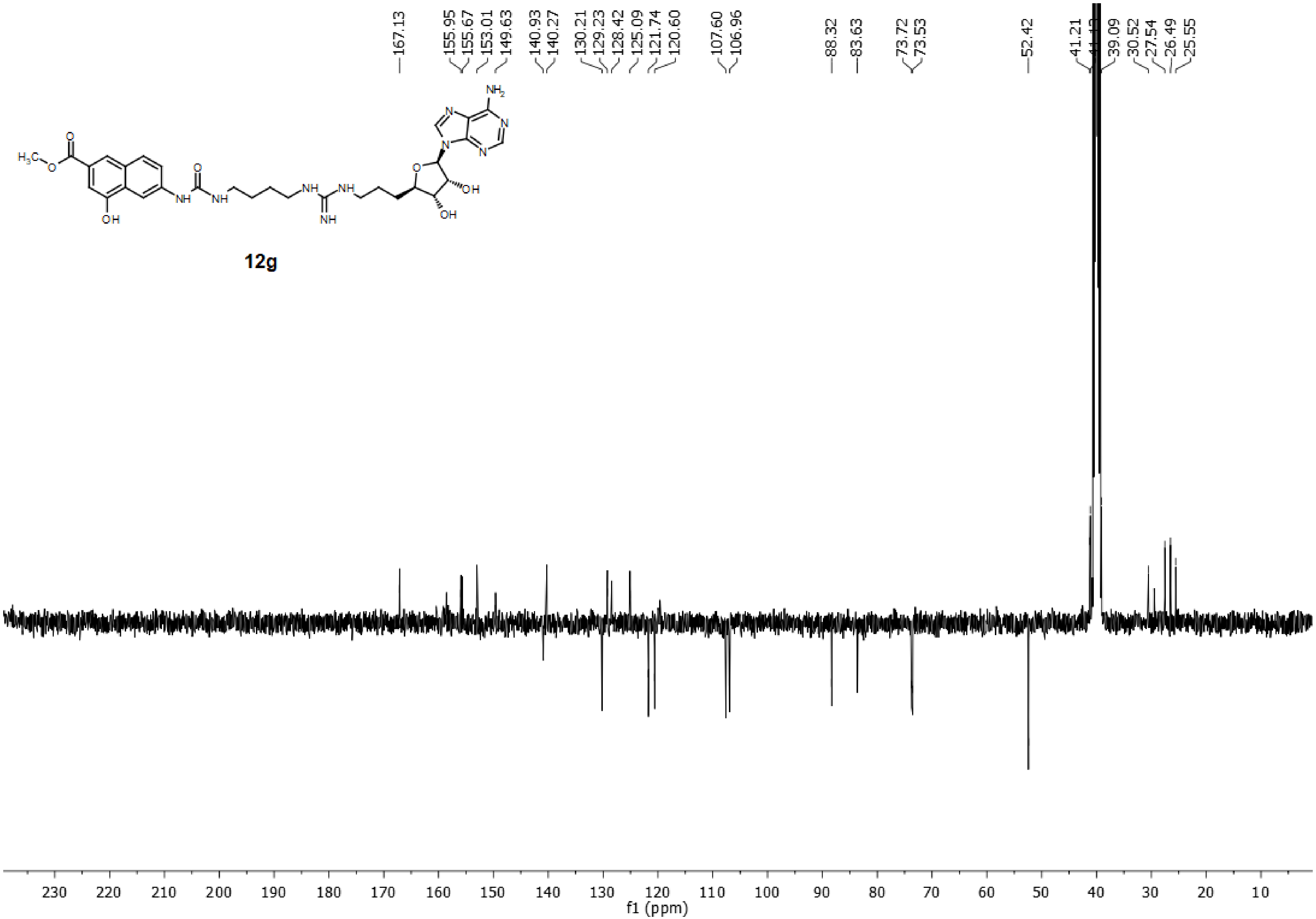

167.13
155.95
155.67
153.01
149.63
140.93
140.27
130.21
129.23
128.42
125.09
121.74
120.60
107.60
106.96
88.32
83.63
73.72
73.53
52.42
41.21
39.09
30.52
27.54
26.49
25.55
12g
230 220 210 200 190 180 170 160 150 140 130 120 110 100 90 80 70 60 50 40 30 20 10
f1 (ppm)

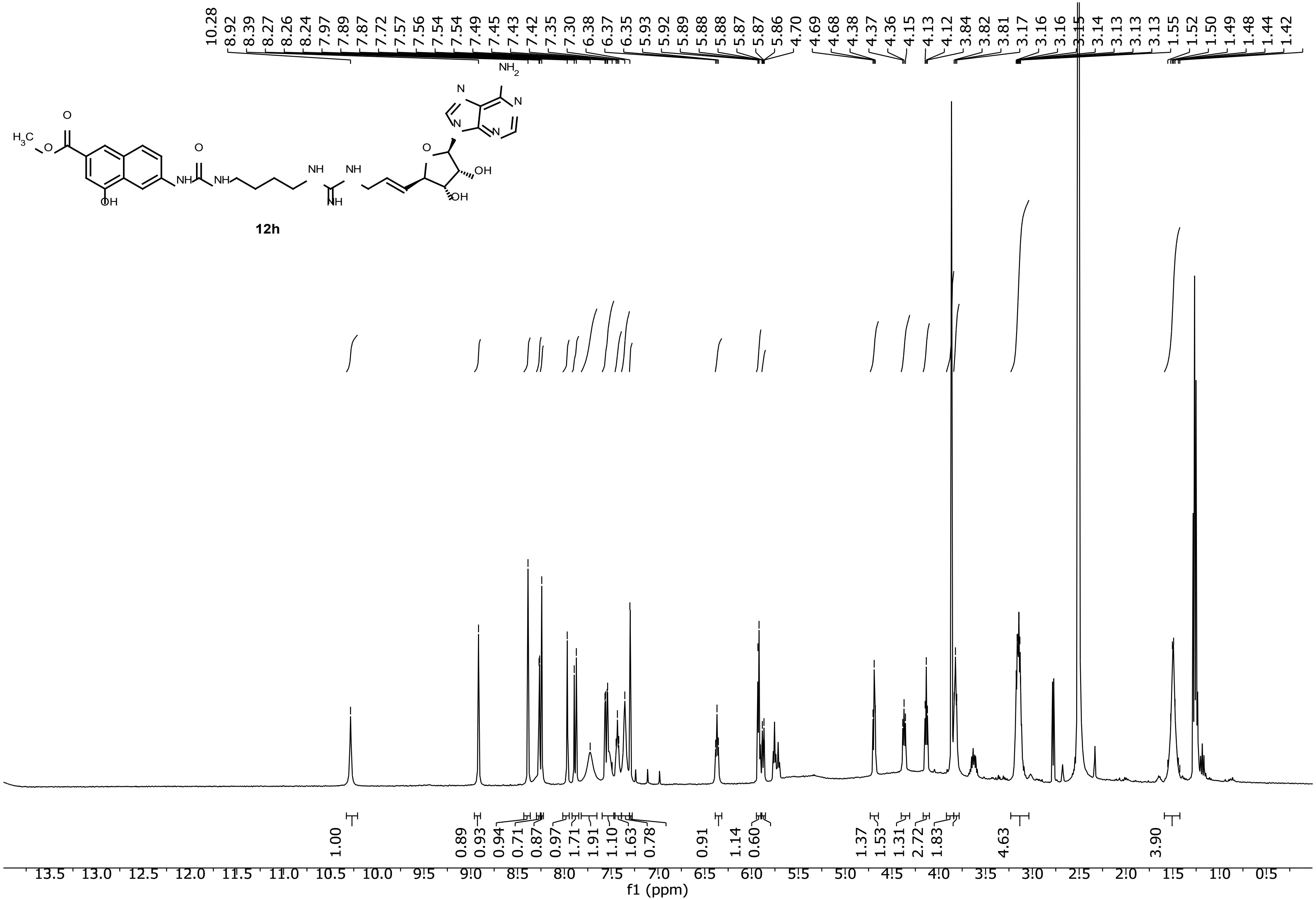

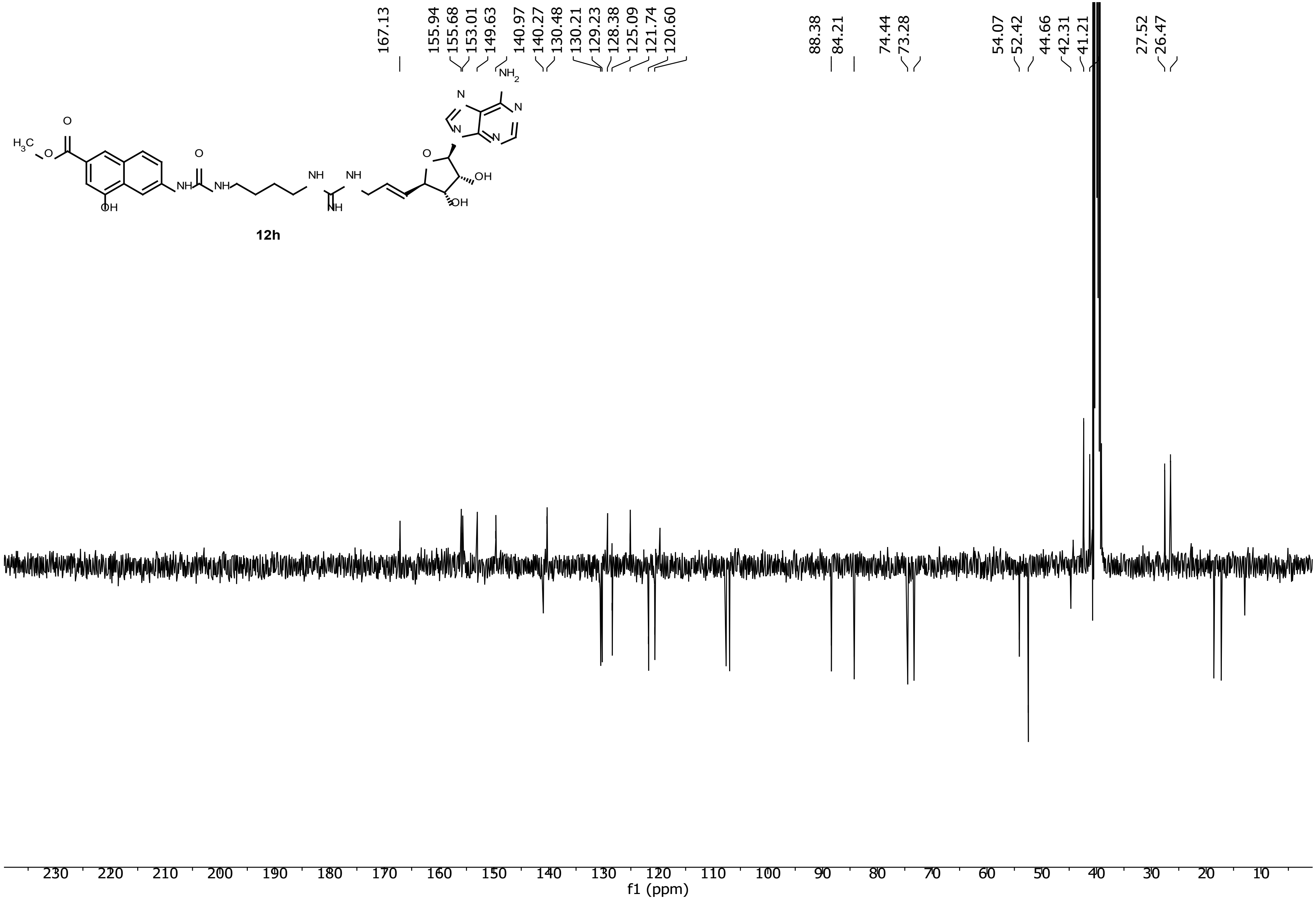
167.13
155.94
155.68
153.01
149.63
140.97
140.27
130.48
130.21
129.23
128.38
125.09
121.74
120.60
88.38
84.21
74.44
73.28
54.07
52.42
44.66
42.31
41.21
27.52
26.47
12h
230 220 210 200 190 180 170 160 150 140 130 120 110 100 90 80 70 60 50 40 30 20 10
f1 (ppm)

HPLC Traces of compound **12a**

SHIMADTZU HPLC ANALYSIS REPORT

Data File Name : EML734.lcd

Chromatogram 220 nm

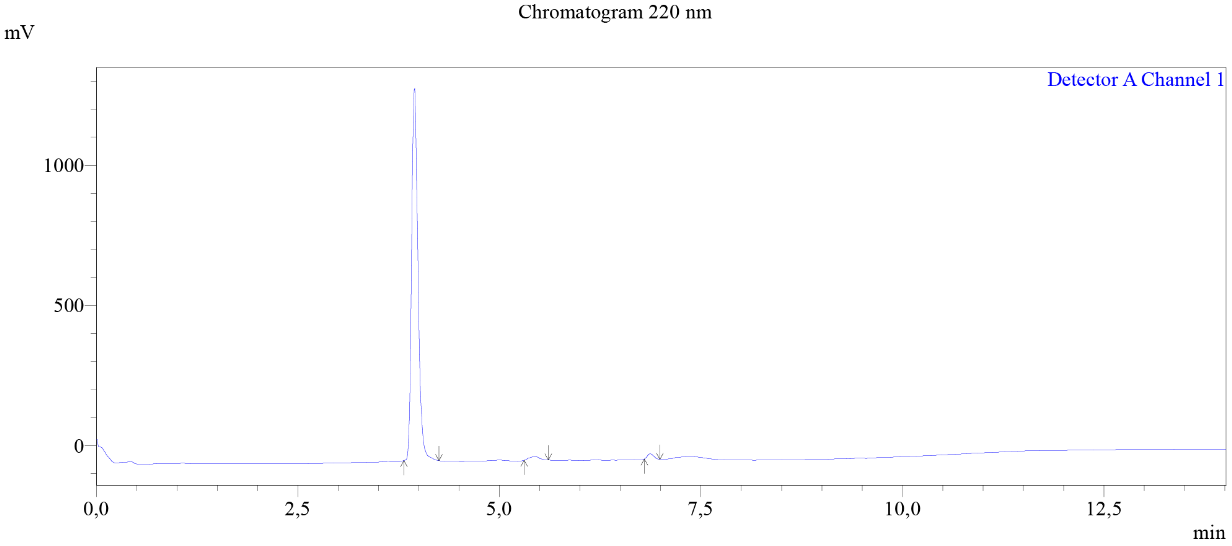

Peak Table

Detector A Channel 1 220nm

Peak#	Ret. Time	Height	Area	Area%
1	3,942	1327058	7302685	97,122
2	5,436	13546	115682	1,539
3	6,867	18730	100711	1,339
Total		1359334	7519077	100,000

Chromatogram 254 nm

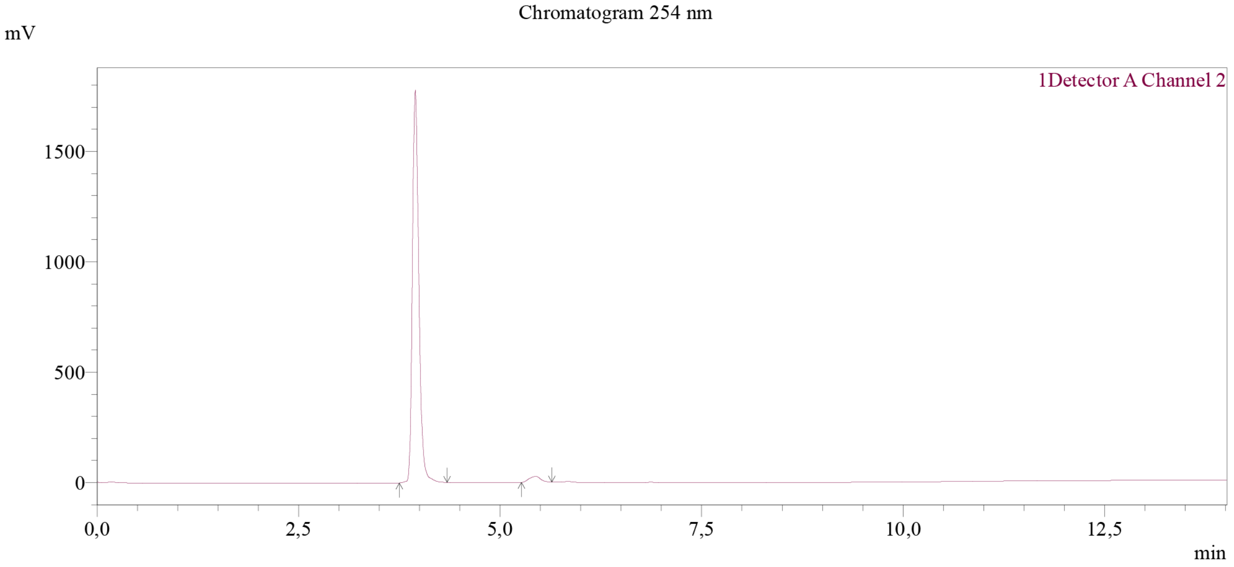

Peak Table

Detector A Channel 2 254nm

Peak#	Ret. Time	Height	Area	Area%
1	3,944	1779565	9879389	97,360
2	5,436	26906	267928	2,640
Total		1806471	10147317	100,000

HPLC Traces of compound **12b**

SHIMADTZU HPLC ANALYSIS REPORT

Data File Name : EML709.lcd

Chromatogram 220 nm

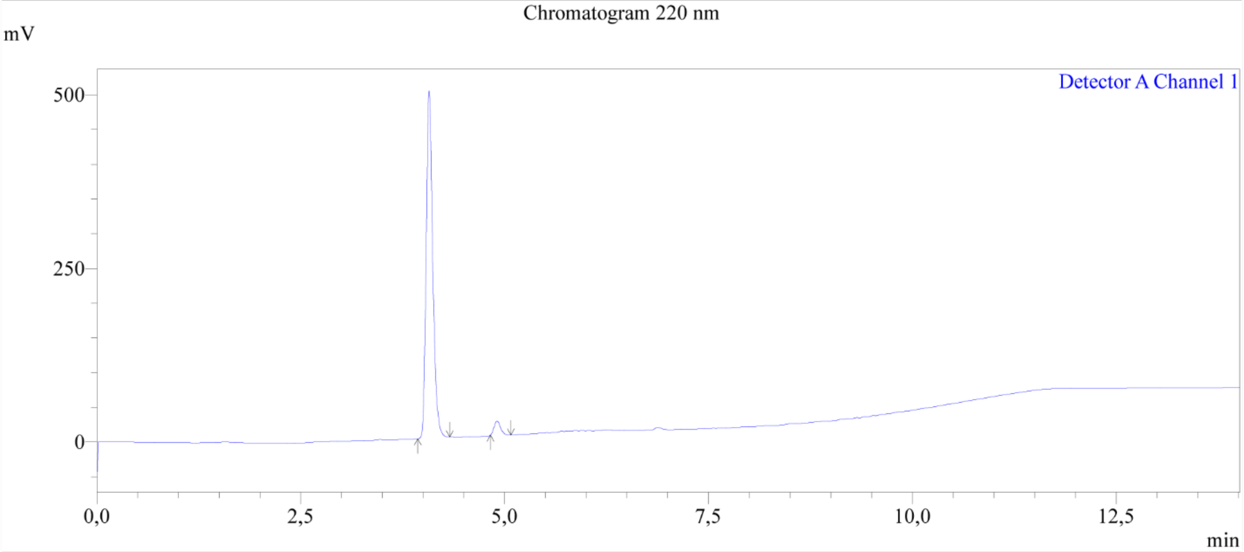

Peak Table

Detector A Channel 1 220nm

Peak#	Ret. Time	Height	Area	Area%
1	4,070	500641	2798814	96,212
2	4,903	20471	110182	3,788
Total		521112	2908996	100,000

Chromatogram 254 nm

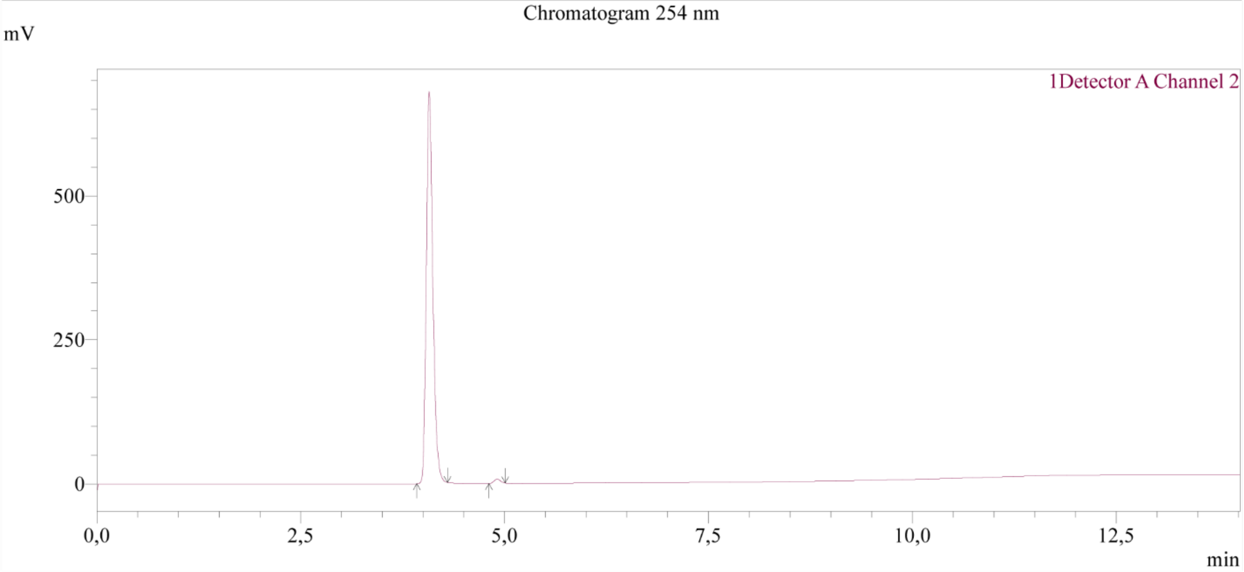

Peak Table

Detector A Channel 2 254nm

Peak#	Ret. Time	Height	Area	Area%
1	4,072	680261	3785977	99,007
2	4,904	7129	37989	0,993
Total		687389	3823966	100,000

HPLC Traces of compound **12c**

SHIMADTZU HPLC ANALYSIS REPORT

Data File Name : EML736.lcd

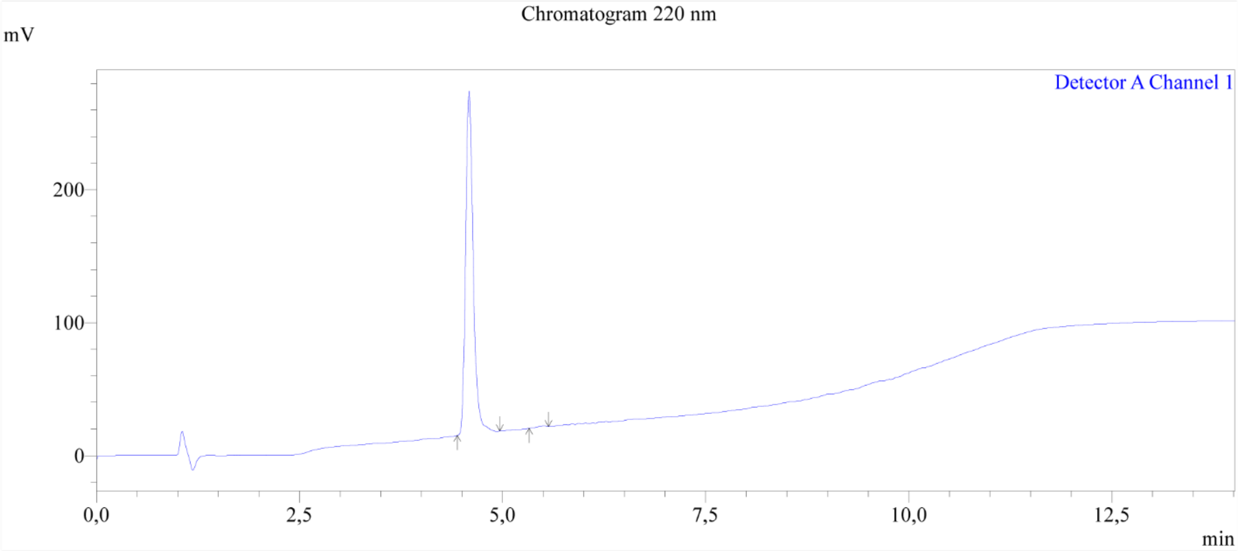

Peak Table

Detector A Channel 1 220nm

Peak#	Ret. Time	Height	Area	Area%
1	4,583	257927	1676431	99,666
2	5,504	827	5621	0,334
Total		258754	1682053	100,000

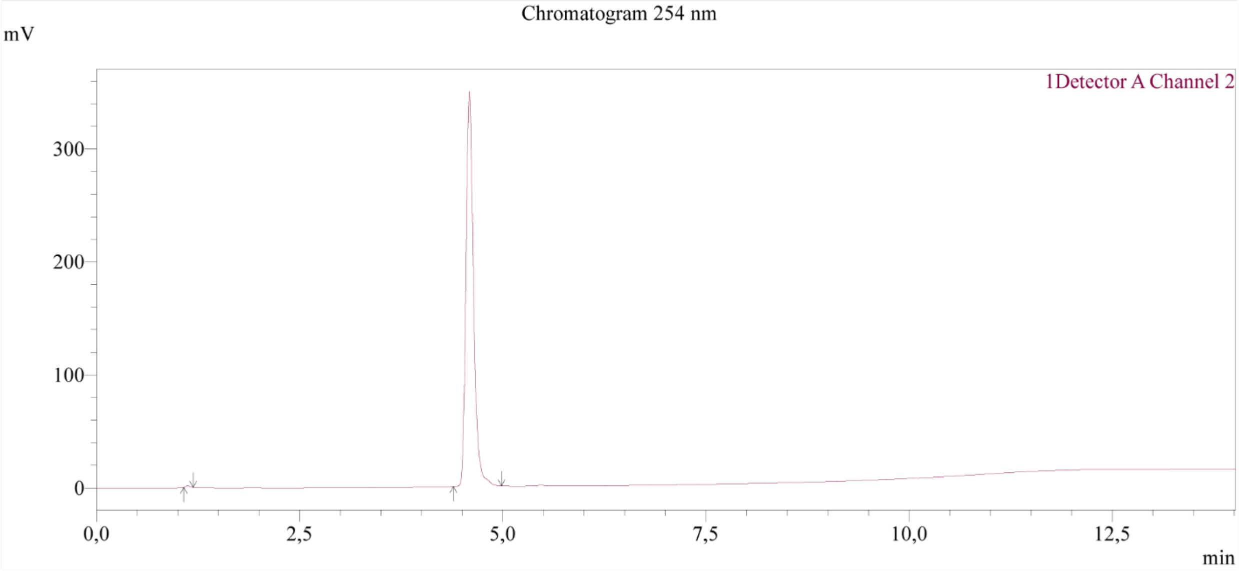

Peak Table

Detector A Channel 2 254nm

Peak#	Ret. Time	Height	Area	Area%
1	1,119	1623	5681	0,250
2	4,584	349363	2266847	99,750
Total		350986	2272528	100,000

HPLC Traces of compound **12d**

SHIMADTZU HPLC ANALYSIS REPORT

Data File Name : EML978.lcd

Chromatogram 220 nm

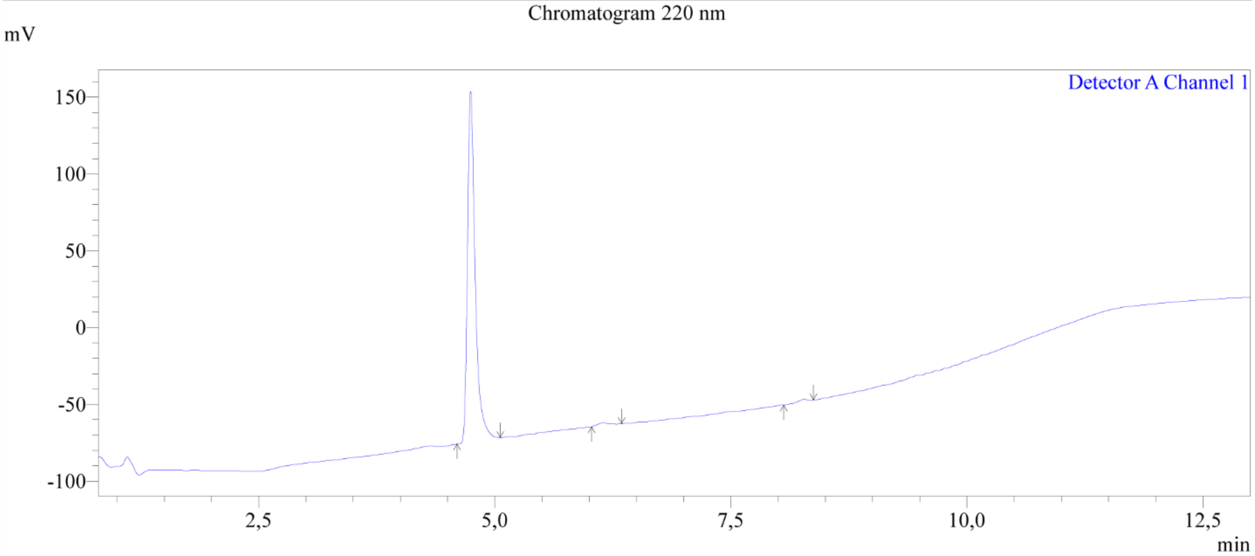

Peak Table

Detector A Channel 1 220nm

Peak#	Ret. Time	Height	Area	Area%
1	4,741	228384	1302258	98,004
2	6,144	2091	15906	1,197
3	8,276	1545	10611	0,799
Total		232020	1328775	100,000

Chromatogram 254 nm

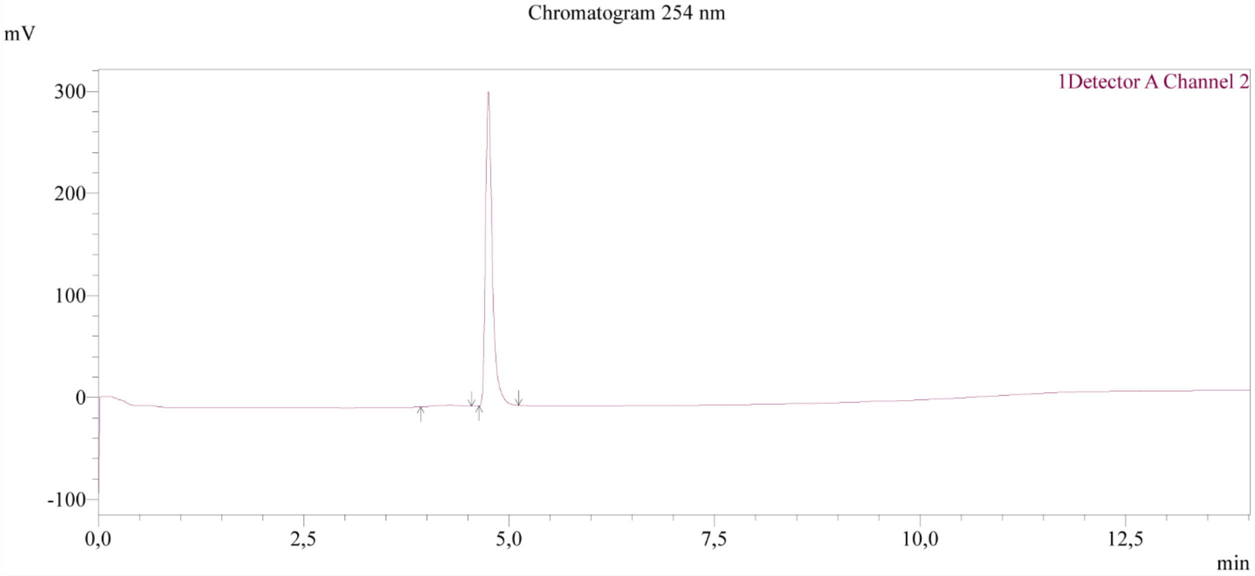

Peak Table

Detector A Channel 2 254nm

Peak#	Ret. Time	Height	Area	Area%
1	4,279	1394	25406	1,420
2	4,742	307849	1764317	98,580
Total		309243	1789724	100,000

HPLC Traces of compound **12e**

SHIMADTZU HPLC ANALYSIS REPORT

Data File Name : EML979.lcd

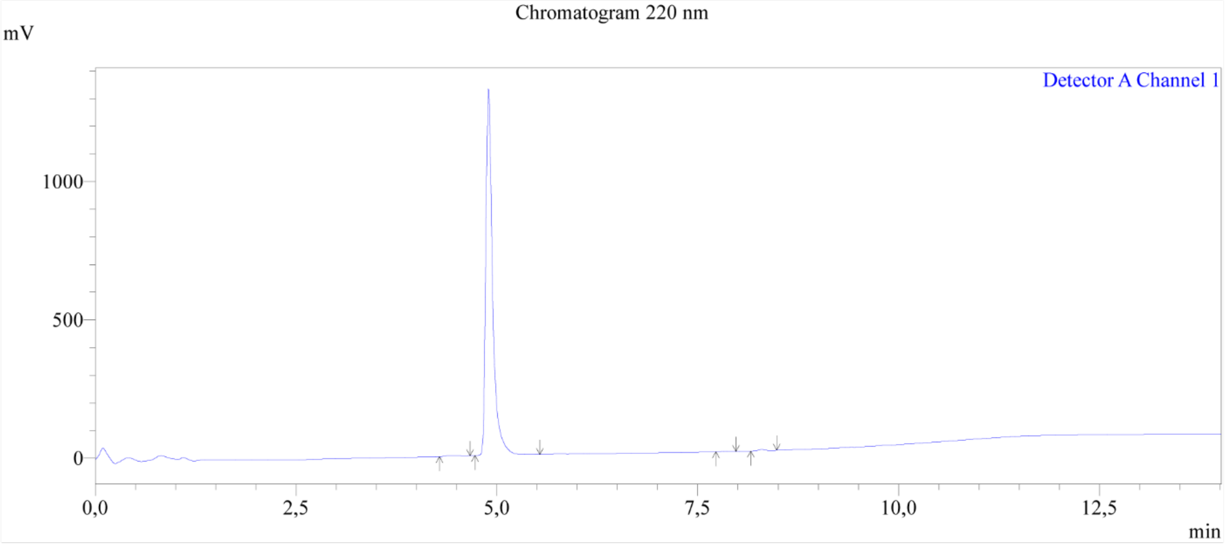

Peak Table

Detector A Channel 1 220nm

Peak#	Ret. Time	Height	Area	Area%
1	4,498	1748	24257	0,307
2	4,891	1323486	7832191	99,284
3	7,837	1580	10070	0,128
4	8,288	4020	22141	0,281
Total		1330834	7888660	100,000

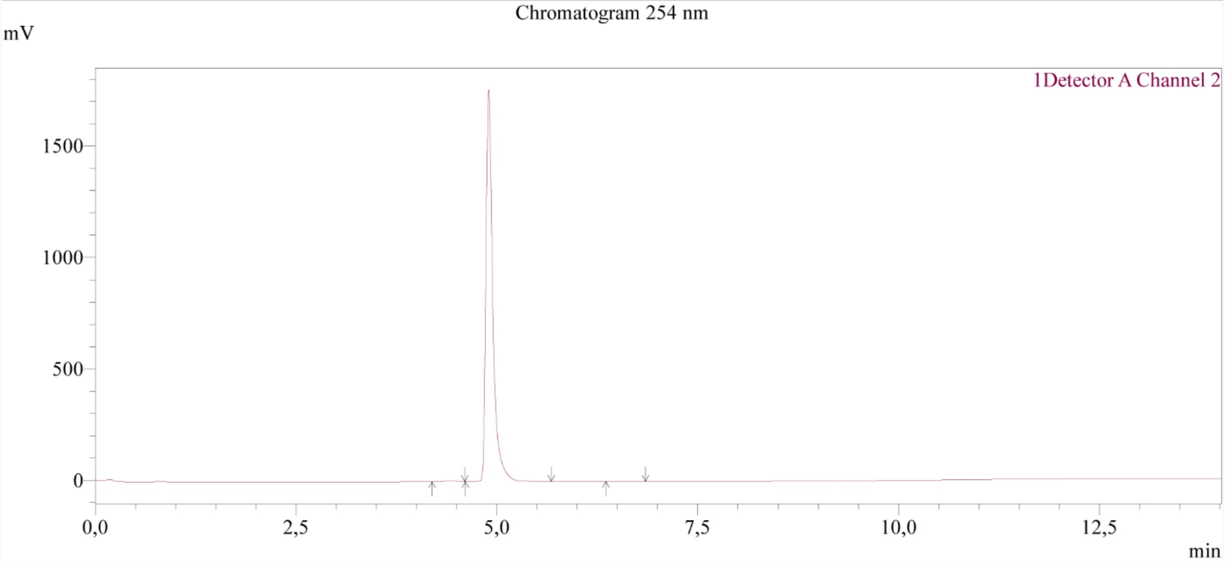

Peak Table

Detector A Channel 2 254nm

Peak#	Ret. Time	Height	Area	Area%
1	4,388	3287	32127	0,305
2	4,893	1756030	10498271	99,596
3	6,666	1015	10413	0,099
Total		1760332	10540811	100,000

HPLC Traces of compound **12f**

SHIMADTZU HPLC ANALYSIS REPORT

Data File Name : EML980.lcd

Chromatogram 220 nm

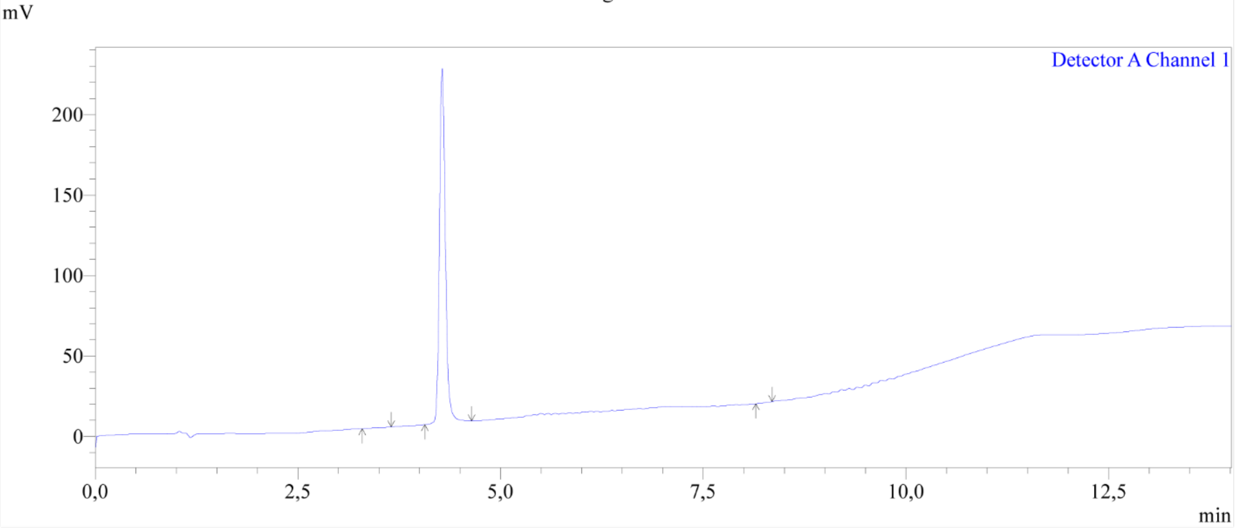

Peak Table

Detector A Channel 1 220nm

Peak#	Ret. Time	Height	Area	Area%
1	3,642	6	1594	0,142
2	4,275	220168	1117711	99,825
3	8,342	-58	366	0,033
Total		220116	1119671	100,000

Chromatogram 254 nm

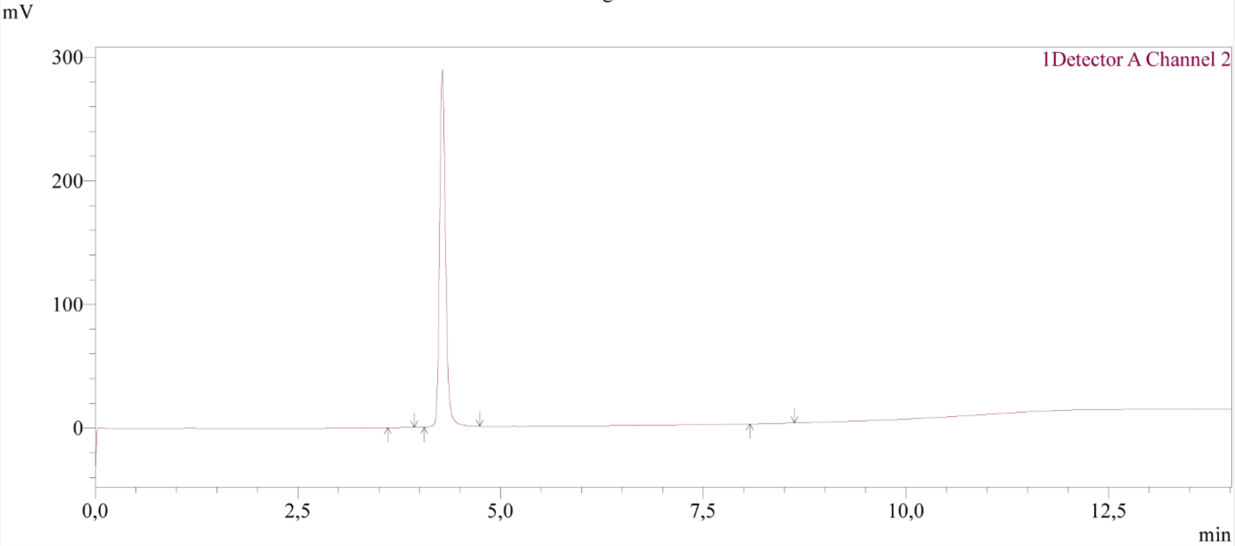

Peak Table

Detector A Channel 2 254nm

Peak#	Ret. Time	Height	Area	Area%
1	3,824	100	549	0,037
2	4,277	289360	1478164	99,992
3	8,617	-1	-431	-0,029
Total		289458	1478281	100,000

HPLC Traces of compound **12g**

SHIMADTZU HPLC ANALYSIS REPORT

Data File Name : EML982.lcd

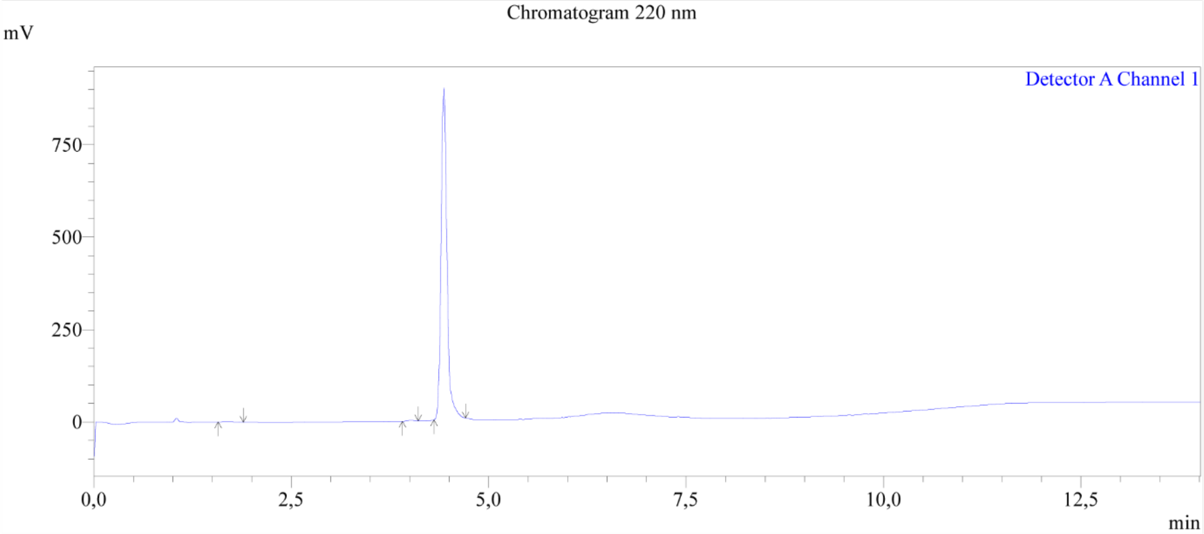

Peak Table

Detector A Channel 1 220nm

Peak#	Ret. Time	Height	Area	Area%
1	1,676	1329	11757	0,251
2	4,014	1923	10936	0,234
3	4,428	897821	4655769	99,515
Total		901073	4678462	100,000

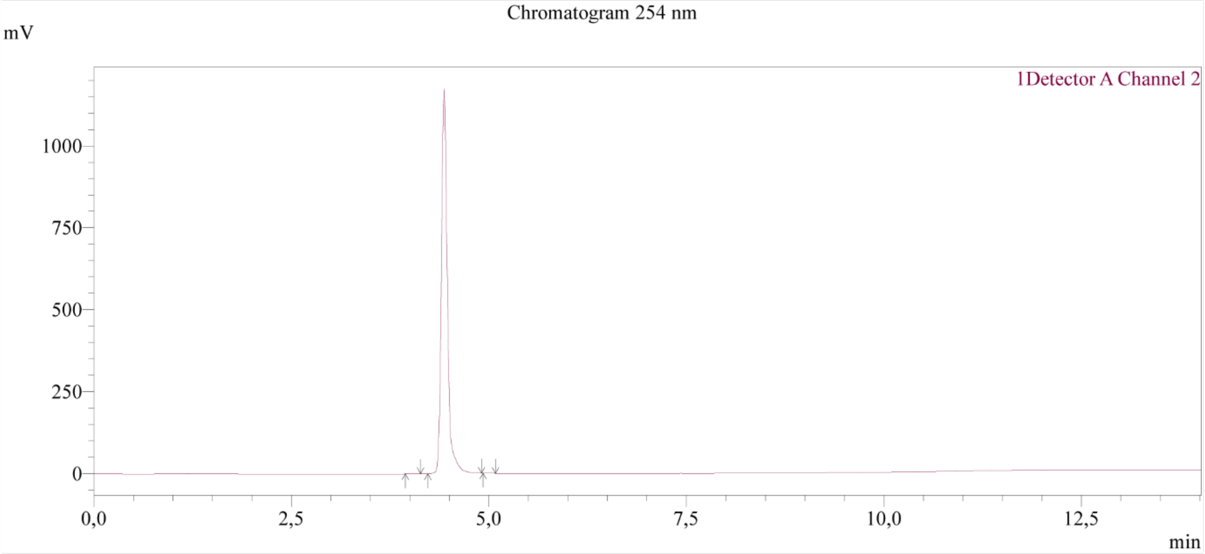

Peak Table

Detector A Channel 2 254nm

Peak#	Ret. Time	Height	Area	Area%
1	4,015	360	1989	0,032
2	4,429	1173656	6199655	99,937
3	4,979	401	1929	0,031
Total		1174417	6203574	100,000

HPLC Traces of compound **12h**

SHIMADTZU HPLC ANALYSIS REPORT

Data File Name : EML981.lcd

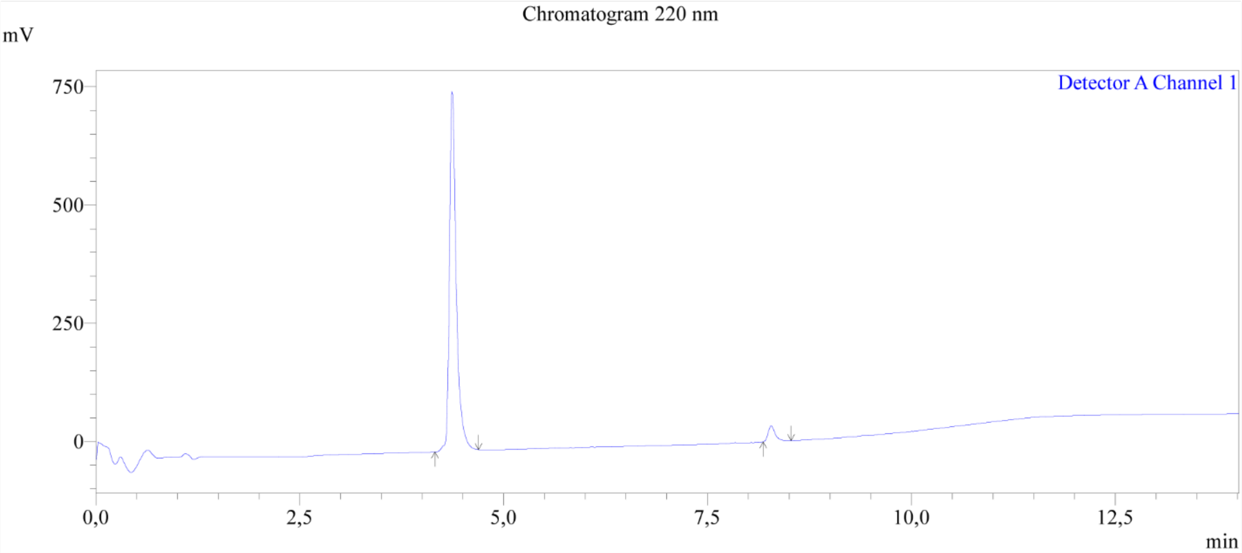

Peak Table

Detector A Channel 1 220nm

Peak#	Ret. Time	Height	Area	Area%
1	4,365	759645	4397472	95,647
2	8,277	33339	200112	4,353
Total		792985	4597584	100,000

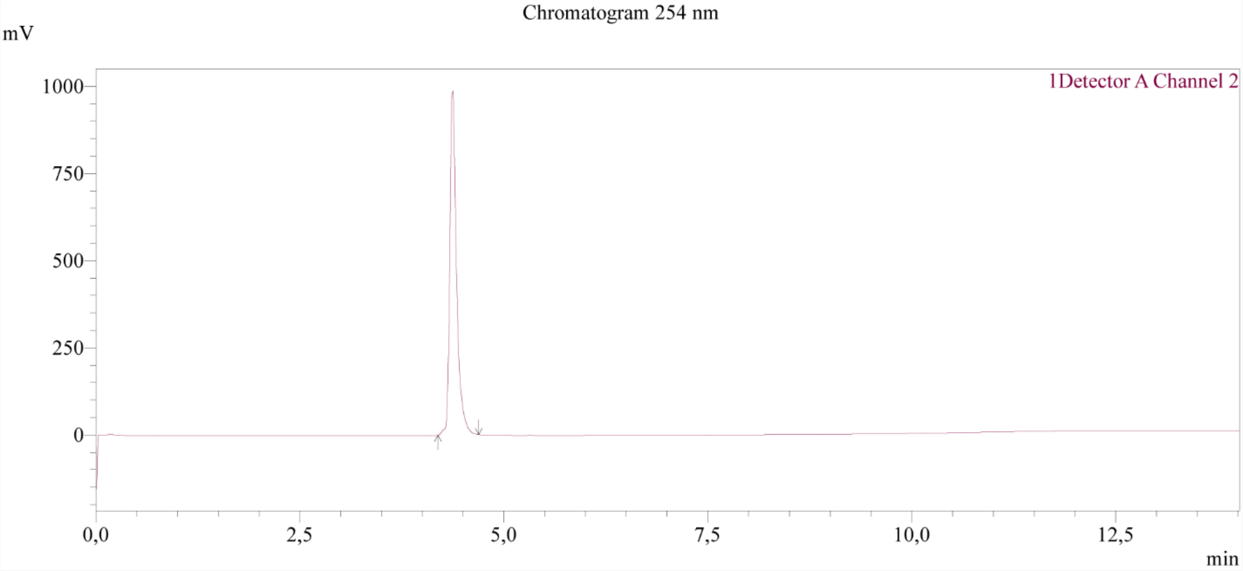

Peak Table

Detector A Channel 2 254nm

Peak#	Ret. Time	Height	Area	Area%
1	4,368	986945	5700448	100,000
Total		986945	5700448	100,000
