# Supplementary material for: Turning Nonselective Inhibitors of Type I Protein Arginine Methyltransferases into Potent and Selective Inhibitors of Protein Arginine Methyltransferase 4 through a Deconstruction–Reconstruction and Fragment-Growing Approach
Source: J Med Chem. 2022 Apr 28;65(17):11574–606. doi: 10.1021/acs.jmedchem.2c00252 (PMC9469100; doi:10.1021/acs.jmedchem.2c00252)
Supplement: Supplementary file 3 — jm2c00252_si_003.pdf [file jm2c00252_si_003.pdf]

Full wwPDB X-ray Structure Validation Report (i)

CONFIDENTIAL VALIDATION REPORT

Oct 4, 2021 – 09:31 am BST

PDB ID	:	7PV6
Title	:	CARM1 in complex with EML734
Deposited on	:	2021-10-01
Resolution	:	2.40 Å(reported)

This is a Full wwPDB X-ray Structure Validation Report.

This report is produced by the wwPDB biocuration pipeline after annotation of the structure.

We welcome your comments at *validation@mail.wwpdb.org*
A user guide is available at
https://www.wwpdb.org/validation/2017/XrayValidationReportHelp
with specific help available everywhere you see the (i) symbol.

The following versions of software and data (see references (i)) were used in the production of this report:

MolProbity	:	4.02b-467
Mogul	:	1.8.5 (274361), CSD as541be (2020)
Xtriage (Phenix)	:	1.13
EDS	:	2.23.2
buster-report	:	1.1.7 (2018)
Percentile statistics	:	20191225.v01 (using entries in the PDB archive December 25th 2019)
Refmac	:	5.8.0267
CCP4	:	7.1.010 (Gargrove)
Ideal geometry (proteins)	:	Engh & Huber (2001)
Ideal geometry (DNA, RNA)	:	Parkinson et al. (1996)
Validation Pipeline (wwPDB-VP)	:	2.23.2

1 Overall quality at a glance (i)

The following experimental techniques were used to determine the structure:
X-RAY DIFFRACTION

The reported resolution of this entry is 2.40 Å.

Percentile scores (ranging between 0-100) for global validation metrics of the entry are shown in the following graphic. The table shows the number of entries on which the scores are based.

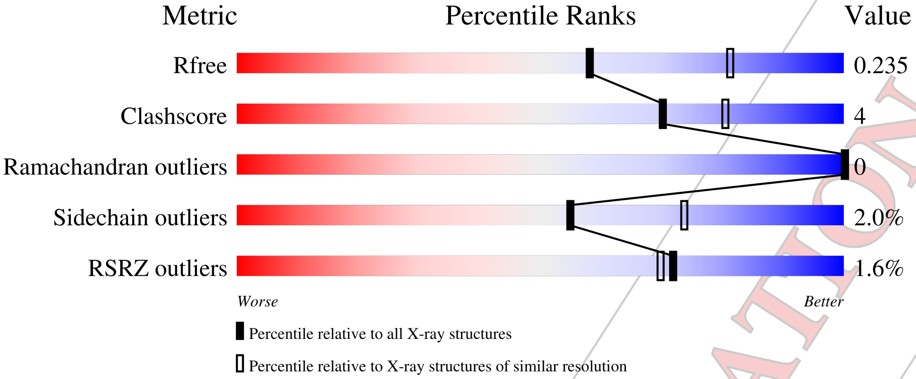

Metric	Whole archive (#Entries)	Similar resolution (#Entries, resolution range(Å))
R_{free}	130704	3907 (2.40-2.40)
Clashscore	141614	4398 (2.40-2.40)
Ramachandran outliers	138981	4318 (2.40-2.40)
Sidechain outliers	138945	4319 (2.40-2.40)
RSRZ outliers	127900	3811 (2.40-2.40)

The table below summarises the geometric issues observed across the polymeric chains and their fit to the electron density. The red, orange, yellow and green segments of the lower bar indicate the fraction of residues that contain outliers for >=3, 2, 1 and 0 types of geometric quality criteria respectively. A grey segment represents the fraction of residues that are not modelled. The numeric value for each fraction is indicated below the corresponding segment, with a dot representing fractions <=5% The upper red bar (where present) indicates the fraction of residues that have poor fit to the electron density. The numeric value is given above the bar.

Mol	Chain	Length	Quality of chain
1	A	368	2%<br>91% 7% •
1	B	368	2%<br>83% 10% • 7%
1	C	368	%<br>88% 10% •
1	D	368	2%<br>82% 10% • 7%

CONFIDENTIAL VALIDATION REPORT

2 Entry composition (i)

There are 5 unique types of molecules in this entry. The entry contains 22948 atoms, of which 11264 are hydrogens and 0 are deuteriums.

In the tables below, the ZeroOcc column contains the number of atoms modelled with zero occupancy, the AltConf column contains the number of residues with at least one atom in alternate conformation and the Trace column contains the number of residues modelled with at most 2 atoms.

- Molecule 1 is a protein called Histone-arginine methyltransferase CARM1.

Mol	Chain	Residues	Atoms						ZeroOcc	AltConf	Trace
			Total	C	H	N	O	S			
1	A	362	5704	1858	2818	476	537	15	0	0	0
1	B	343	5460	1778	2705	457	506	14	0	0	0
1	C	362	5704	1858	2818	476	537	15	0	0	0
1	D	343	5460	1778	2705	457	506	14	0	0	0

- Molecule 2 is methyl 6-[3-[[{N}-[[(2 {R},3 {S},4 {R},5 {R})-5-(6-aminopurin-9-yl)-3,4-bis(oxidanyl)oxolan-2-yl]methyl]carbamimidoyl]amino]propylcarbamoylamino]-4-oxidanyl-naphthalene-2-carboxylate (three-letter code: LRZ) (formula: $C_{27}H_{32}N_{10}O_7$) (labeled as "Ligand of Interest" by depositor).

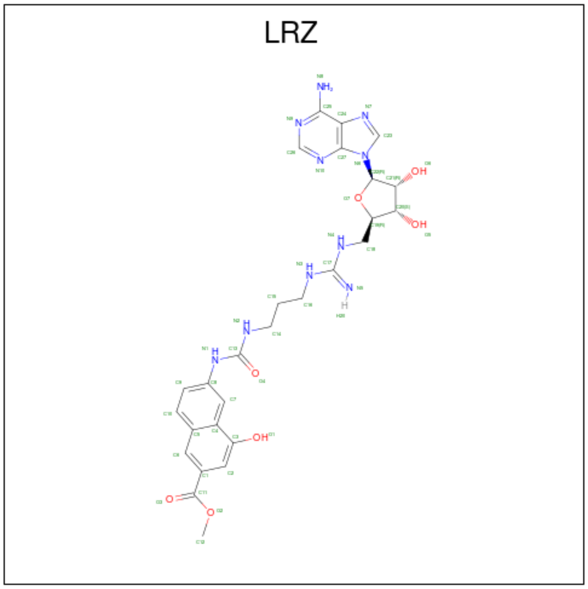

Mol	Chain	Residues	Atoms					ZeroOcc	AltConf
			Total	C	H	N	O		
2	A	1	76	27	32	10	7	0	0

Continued on next page...

VALIDATION REPORT

Continued from previous page...

Mol	Chain	Residues	Atoms	ZeroOcc	AltConf
2	B	1	Total C H N O<br>76 27 32 10 7	0	0
2	C	1	Total C H N O<br>76 27 32 10 7	0	0
2	D	1	Total C H N O<br>76 27 32 10 7	0	0

- Molecule 3 is 1,2-ETHANEDIOL (three-letter code: EDO) (formula: $C_2H_6O_2$).

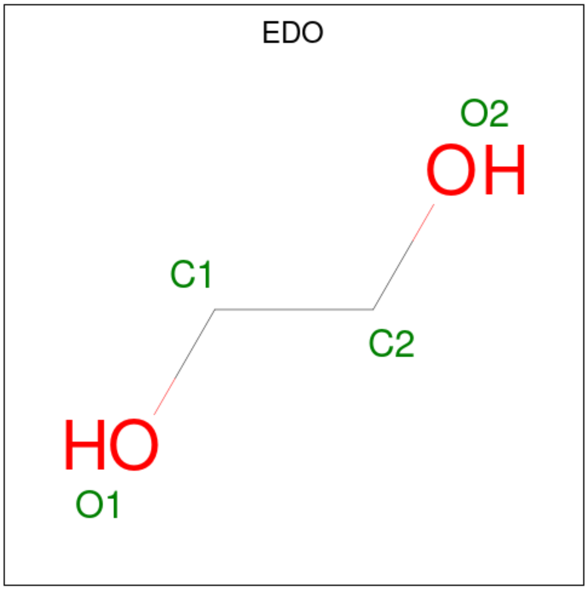

Mol	Chain	Residues	Atoms	ZeroOcc	AltConf
3	A	1	Total C H O<br>10 2 6 2	0	0
3	A	1	Total C H O<br>10 2 6 2	0	0
3	A	1	Total C H O<br>10 2 6 2	0	0
3	A	1	Total C H O<br>10 2 6 2	0	0
3	A	1	Total C H O<br>10 2 6 2	0	0
3	A	1	Total C H O<br>10 2 6 2	0	0
3	B	1	Total C H O<br>10 2 6 2	0	0
3	B	1	Total C H O<br>10 2 6 2	0	0

Continued on next page...

CONFIDENTIAL VALIDATION REPORT

Continued from previous page...

Mol	Chain	Residues	Atoms				ZeroOcc	AltConf
			Total	C	H	O		
3	B	1	10	2	6	2	0	0
3	C	1	10	2	6	2	0	0
3	C	1	10	2	6	2	0	0
3	C	1	10	2	6	2	0	0

- Molecule 4 is TETRAETHYLENE GLYCOL (three-letter code: PG4) (formula: $C_8H_{18}O_5$).

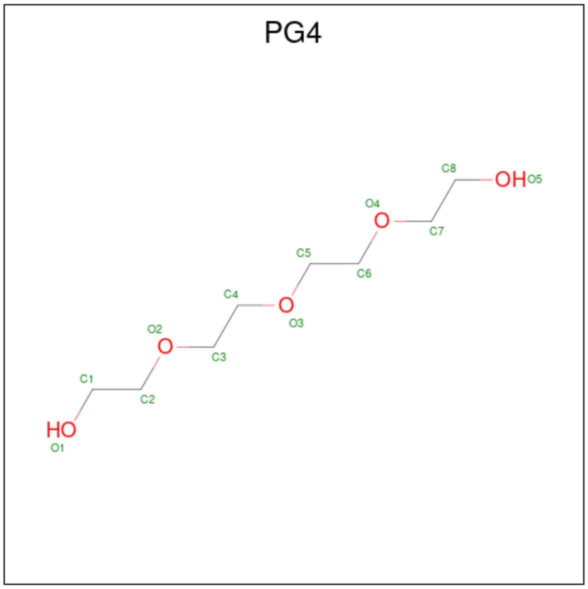

Mol	Chain	Residues	Atoms				ZeroOcc	AltConf
			Total	C	H	O		
4	C	1	31	8	18	5	0	0

- Molecule 5 is water.

Mol	Chain	Residues	Atoms		ZeroOcc	AltConf
			Total	O		
5	A	50	50	50	0	0
5	B	42	42	42	0	0
5	C	36	36	36	0	0
5	D	37	37	37	0	0

CONFIDENTIAL VALIDATION REPORT

3 Residue-property plots ⓘ

These plots are drawn for all protein, RNA, DNA and oligosaccharide chains in the entry. The first graphic for a chain summarises the proportions of the various outlier classes displayed in the second graphic. The second graphic shows the sequence view annotated by issues in geometry and electron density. Residues are color-coded according to the number of geometric quality criteria for which they contain at least one outlier: green = 0, yellow = 1, orange = 2 and red = 3 or more. A red dot above a residue indicates a poor fit to the electron density (RSRZ > 2). Stretches of 2 or more consecutive residues without any outlier are shown as a green connector. Residues present in the sample, but not in the model, are shown in grey.

- Molecule 1: Histone-arginine methyltransferase CARM1

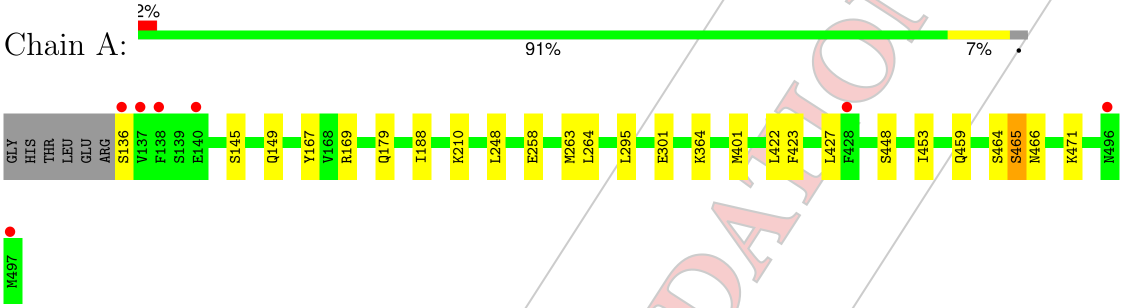

- Molecule 1: Histone-arginine methyltransferase CARM1

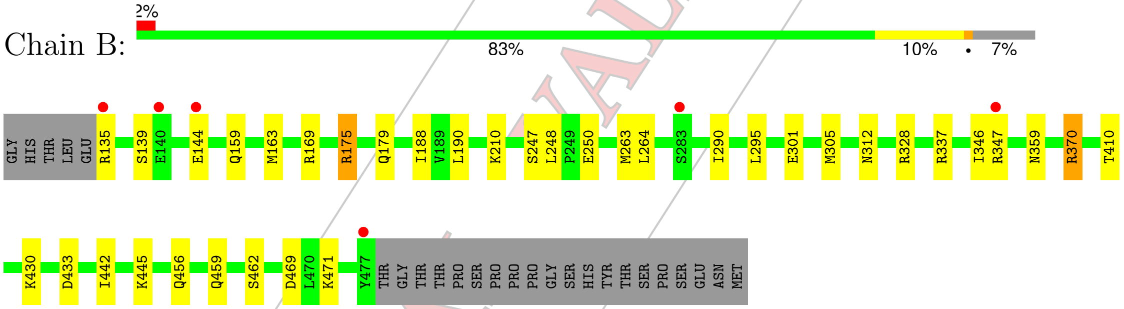

- Molecule 1: Histone-arginine methyltransferase CARM1

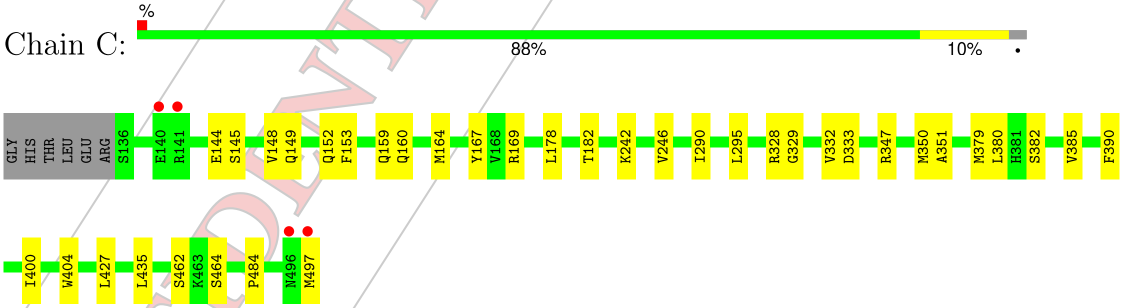

- Molecule 1: Histone-arginine methyltransferase CARM1

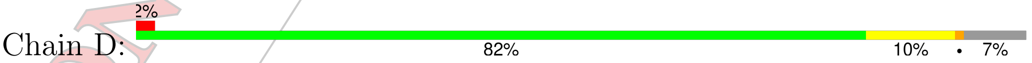

CONFIDENTIAL VALIDATION REPORT

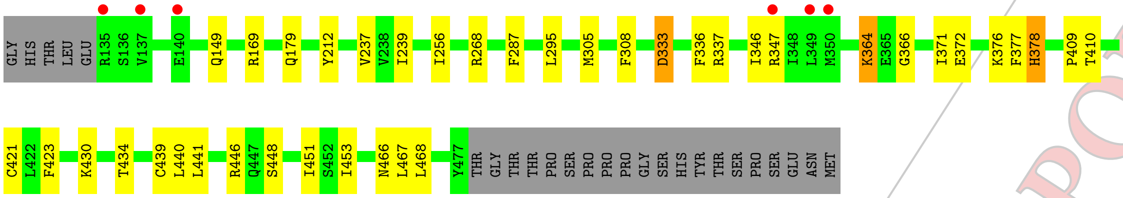

CONFIDENTIAL VALIDATION REPORT

4 Data and refinement statistics (i)

Property	Value	Source
Space group	P 21 21 2	Depositor
Cell constants<br>a, b, c, α, β, γ	75.00Å 99.52Å 208.46Å<br>90.00° 90.00° 90.00°	Depositor
Resolution (Å)	46.17 – 2.40<br>46.17 – 2.40	Depositor<br>EDS
% Data completeness<br>(in resolution range)	99.4 (46.17-2.40)<br>99.6 (46.17-2.40)	Depositor<br>EDS
R_{merge}	0.13	Depositor
R_{sym}	(Not available)	Depositor
$< I/\sigma(I) >$ [1]	1.65 (at 2.39Å)	Xtriage
Refinement program	PHENIX 1.19.2_4158	Depositor
R, R_{free}	0.191 , 0.237<br>0.189 , 0.235	Depositor<br>DCC
R_{free} test set	3092 reflections (4.99%)	wwPDB-VP
Wilson B-factor (Å^2)	47.1	Xtriage
Anisotropy	0.437	Xtriage
Bulk solvent k_{sol}(e/Å^3), B_{sol}(Å^2)	(Not available) , (Not available)	EDS
L-test for twinning[2]	$< \|L\| > = 0.50$, $< L^2 > = 0.33$	Xtriage
Estimated twinning fraction	No twinning to report.	Xtriage
F_o,F_c correlation	0.95	EDS
Total number of atoms	22948	wwPDB-VP
Average B, all atoms (Å^2)	61.0	wwPDB-VP

Xtriage's analysis on translational NCS is as follows: *The analyses of the Patterson function reveals a significant off-origin peak that is 38.42 % of the origin peak, indicating pseudo-translational symmetry. The chance of finding a peak of this or larger height randomly in a structure without pseudo-translational symmetry is equal to 3.6949e-04. The detected translational NCS is most likely also responsible for the elevated intensity ratio.*

[1] Intensities estimated from amplitudes.

[2] Theoretical values of $< |L| >$, $< L^2 >$ for acentric reflections are 0.5, 0.333 respectively for untwinned datasets, and 0.375, 0.2 for perfectly twinned datasets.

CONFIDENTIAL VALIDATION REPORT

5 Model quality (i)

5.1 Standard geometry (i)

Bond lengths and bond angles in the following residue types are not validated in this section: LRZ, EDO, PG4

The Z score for a bond length (or angle) is the number of standard deviations the observed value is removed from the expected value. A bond length (or angle) with $|Z| > 5$ is considered an outlier worth inspection. RMSZ is the root-mean-square of all Z scores of the bond lengths (or angles).

Mol	Chain	Bond lengths		Bond angles	
		RMSZ	#$|Z|$ >5	RMSZ	#$|Z|$ >5
1	A	0.39	0/2963	0.55	0/4021
1	B	0.35	0/2825	0.54	0/3827
1	C	0.35	0/2963	0.53	0/4021
1	D	0.33	0/2825	0.54	0/3827
All	All	0.36	0/11576	0.54	0/15696

There are no bond length outliers.

There are no bond angle outliers.

There are no chirality outliers.

There are no planarity outliers.

5.2 Too-close contacts (i)

In the following table, the Non-H and H(model) columns list the number of non-hydrogen atoms and hydrogen atoms in the chain respectively. The H(added) column lists the number of hydrogen atoms added and optimized by MolProbity. The Clashes column lists the number of clashes within the asymmetric unit, whereas Symm-Clashes lists symmetry-related clashes.

Mol	Chain	Non-H	H(model)	H(added)	Clashes	Symm-Clashes
1	A	2886	2818	2818	16	0
1	B	2755	2705	2705	20	0
1	C	2886	2818	2818	24	1
1	D	2755	2705	2705	29	1
2	A	44	32	0	0	0
2	B	44	32	0	0	0
2	C	44	32	0	0	0
2	D	44	32	0	0	0
3	A	24	36	36	0	0

Continued on next page...

CONFIDENTIAL VALIDATION REPORT

Continued from previous page...

Mol	Chain	Non-H	H(model)	H(added)	Clashes	Symm-Clashes
3	B	12	18	18	0	0
3	C	12	18	18	0	0
4	C	13	18	18	1	0
5	A	50	0	0	2	0
5	B	42	0	0	0	0
5	C	36	0	0	0	0
5	D	37	0	0	1	0
All	All	11684	11264	11136	82	1

The all-atom clashscore is defined as the number of clashes found per 1000 atoms (including hydrogen atoms). The all-atom clashscore for this structure is 4.

All (82) close contacts within the same asymmetric unit are listed below, sorted by their clash magnitude.

Atom-1	Atom-2	Interatomic distance (Å)	Clash overlap (Å)
1:C:350:MET:CE	1:C:385:VAL:HG22	2.20	0.71
1:B:370:ARG:HG2	1:B:442:ILE:HD13	1.72	0.71
1:D:337:ARG:HA	1:D:468:LEU:HD23	1.72	0.70
1:D:364:LYS:HD3	1:D:366:GLY:H	1.60	0.66
1:D:421:CYS:HB3	1:D:468:LEU:HD11	1.81	0.62
1:D:346:ILE:HG21	1:D:410:THR:HG23	1.81	0.61
1:C:350:MET:HE3	1:C:385:VAL:HG22	1.84	0.60
1:B:312:ASN:HD21	1:D:308:PHE:HE2	1.51	0.57
1:A:423:PHE:HE1	1:A:453:ILE:HG21	1.69	0.56
1:C:427:LEU:HD21	1:C:464:SER:HB2	1.88	0.56
1:C:350:MET:HE1	1:C:385:VAL:HG22	1.86	0.55
1:B:430:LYS:HE2	1:C:167:TYR:HE2	1.72	0.55
1:A:169:ARG:HD2	1:A:258:GLU:OE2	2.07	0.55
1:D:421:CYS:HB3	1:D:468:LEU:CD1	2.36	0.55
1:B:469:ASP:OD1	1:B:471:LYS:HG2	2.09	0.53
1:A:167:TYR:CD2	1:D:430:LYS:HD2	2.44	0.53
1:C:149:GLN:HG3	1:D:149:GLN:OE1	2.09	0.53
1:C:329:GLY:O	1:C:333:ASP:OD2	2.27	0.52
1:B:312:ASN:OD1	1:B:328:ARG:NH2	2.43	0.52
1:C:242:LYS:O	1:C:246:VAL:HG13	2.10	0.52
1:B:290:ILE:HG22	1:B:359:ASN:HA	1.91	0.51
1:B:337:ARG:HD3	1:B:469:ASP:HB2	1.92	0.51
1:D:212:TYR:HD1	1:D:239:ILE:HD11	1.76	0.51
1:B:263:MET:O	1:B:264:LEU:HB3	2.12	0.50
1:A:427:LEU:HD21	1:A:464:SER:HB2	1.95	0.49
1:C:153:PHE:CE2	1:C:159:GLN:HG2	2.48	0.49

Continued on next page...

CONFIDENTIAL VALIDATION REPORT

Continued from previous page...

Atom-1	Atom-2	Interatomic distance (Å)	Clash overlap (Å)
1:A:301:GLU:OE1	5:A:601:HOH:O	2.20	0.48
1:B:445:LYS:N	1:B:445:LYS:HD3	2.28	0.48
1:A:448:SER:HB2	1:A:471:LYS:HD3	1.95	0.48
1:B:188:ILE:HD13	1:B:250:GLU:CD	2.34	0.48
1:C:182:THR:HG23	1:C:400:ILE:HD11	1.95	0.47
1:B:433:ASP:OD1	1:B:459:GLN:HG2	2.14	0.47
1:C:148:VAL:O	1:C:152:GLN:HG3	2.14	0.47
1:B:190:LEU:HD13	1:B:248:LEU:HD21	1.96	0.47
1:C:379:MET:HE3	1:C:385:VAL:HG21	1.96	0.47
1:A:136:SER:N	5:A:606:HOH:O	2.47	0.47
1:C:350:MET:HE1	1:C:385:VAL:HA	1.96	0.47
1:D:371:ILE:HB	1:D:441:LEU:HB2	1.96	0.47
1:D:372:GLU:HG3	1:D:440:LEU:HD13	1.97	0.47
1:C:144:GLU:O	1:C:148:VAL:HG23	2.15	0.46
1:D:347:ARG:HB2	1:D:347:ARG:NH1	2.30	0.46
1:D:347:ARG:HB2	1:D:347:ARG:HH11	1.79	0.46
1:C:295:LEU:HD22	1:C:390:PHE:CD2	2.51	0.46
1:C:145:SER:O	1:C:149:GLN:HG2	2.15	0.46
1:A:465:SER:O	1:A:466:ASN:HB2	2.16	0.45
1:D:346:ILE:HG22	1:D:409:PRO:HG2	1.98	0.45
1:D:337:ARG:HG2	1:D:467:LEU:O	2.17	0.45
1:B:346:ILE:HG21	1:B:410:THR:HG23	1.98	0.45
1:C:160:GLN:O	1:C:164:MET:HG3	2.17	0.45
1:A:167:TYR:CE2	1:D:430:LYS:HD2	2.51	0.45
1:A:188:ILE:HG23	1:A:210:LYS:HB3	1.99	0.45
1:A:459:GLN:OE1	1:A:459:GLN:N	2.49	0.45
1:B:159:GLN:HG3	1:B:163:MET:HE2	1.99	0.44
1:D:237:VAL:O	1:D:239:ILE:HD12	2.17	0.44
1:D:423:PHE:HA	1:D:466:ASN:OD1	2.18	0.44
1:A:145:SER:O	1:A:149:GLN:HG2	2.18	0.43
1:C:178:LEU:HD12	4:C:505:PG4:H22	2.00	0.43
1:A:263:MET:O	1:A:264:LEU:HB3	2.18	0.43
1:D:333:ASP:O	1:D:337:ARG:HG3	2.18	0.43
1:D:256:ILE:HG22	1:D:287:PHE:HB2	2.00	0.43
1:D:336:PHE:O	1:D:468:LEU:CD2	2.67	0.42
1:B:370:ARG:HE	1:B:370:ARG:HB3	1.66	0.42
1:C:379:MET:HE1	1:C:435:LEU:HD22	2.01	0.42
1:B:305:MET:HE1	1:D:305:MET:CE	2.49	0.42
1:D:421:CYS:SG	1:D:468:LEU:CD1	3.07	0.42
1:D:441:LEU:HG	1:D:451:ILE:CD1	2.49	0.42
1:A:422:LEU:O	1:A:466:ASN:ND2	2.33	0.42

Continued on next page...

CONFIDENTIAL VALIDATION REPORT

Continued from previous page...

Atom-1	Atom-2	Interatomic distance (Å)	Clash overlap (Å)
1:A:423:PHE:HE1	1:A:453:ILE:CG2	2.33	0.42
1:C:149:GLN:HG2	1:C:149:GLN:H	1.71	0.41
1:C:351:ALA:HA	1:C:380:LEU:HG	2.02	0.41
1:D:376:LYS:HE2	1:D:378:HIS:HE1	1.86	0.41
1:D:377:PHE:O	1:D:434:THR:HA	2.19	0.41
1:B:175:ARG:HG2	1:B:179:GLN:HG3	2.01	0.41
1:B:175:ARG:HG2	1:B:179:GLN:CG	2.51	0.41
1:B:312:ASN:ND2	1:D:308:PHE:CE2	2.89	0.41
1:A:179:GLN:NE2	1:A:401:MET:SD	2.94	0.41
1:B:456:GLN:HA	1:B:462:SER:O	2.20	0.41
1:C:290:ILE:HD11	1:C:404:TRP:CH2	2.56	0.41
1:D:268:ARG:HA	5:D:619:HOH:O	2.21	0.41
1:C:347:ARG:NH2	1:C:484:PRO:HB3	2.36	0.40
1:C:328:ARG:O	1:C:332:VAL:HG23	2.22	0.40
1:D:439:CYS:SG	1:D:453:ILE:CD1	3.10	0.40

All (1) symmetry-related close contacts are listed below. The label for Atom-2 includes the symmetry operator and encoded unit-cell translations to be applied.

Atom-1	Atom-2	Interatomic distance (Å)	Clash overlap (Å)
1:C:382:SER:H	1:D:179:GLN:OE1[2_655]	1.59	0.01

5.3 Torsion angles (i)

5.3.1 Protein backbone (i)

In the following table, the Percentiles column shows the percent Ramachandran outliers of the chain as a percentile score with respect to all X-ray entries followed by that with respect to entries of similar resolution.

The Analysed column shows the number of residues for which the backbone conformation was analysed, and the total number of residues.

Mol	Chain	Analysed	Favoured	Allowed	Outliers	Percentiles	
1	A	360/368 (98%)	348 (97%)	12 (3%)	0	100	100
1	B	341/368 (93%)	333 (98%)	8 (2%)	0	100	100
1	C	360/368 (98%)	349 (97%)	11 (3%)	0	100	100
1	D	341/368 (93%)	331 (97%)	10 (3%)	0	100	100

Continued on next page...

CONFIDENTIAL VALIDATION REPORT

Continued from previous page...

Mol	Chain	Analysed	Favoured	Allowed	Outliers	Percentiles	
All	All	1402/1472 (95%)	1361 (97%)	41 (3%)	0	100	100

There are no Ramachandran outliers to report.

5.3.2 Protein sidechains ⓘ

In the following table, the Percentiles column shows the percent sidechain outliers of the chain as a percentile score with respect to all X-ray entries followed by that with respect to entries of similar resolution.

The Analysed column shows the number of residues for which the sidechain conformation was analysed, and the total number of residues.

Mol	Chain	Analysed	Rotameric	Outliers	Percentiles	
1	A	316/321 (98%)	312 (99%)	4 (1%)	69	84
1	B	299/321 (93%)	288 (96%)	11 (4%)	34	53
1	C	316/321 (98%)	313 (99%)	3 (1%)	78	90
1	D	299/321 (93%)	292 (98%)	7 (2%)	50	70
All	All	1230/1284 (96%)	1205 (98%)	25 (2%)	55	74

All (25) residues with a non-rotameric sidechain are listed below:

Mol	Chain	Res	Type
1	A	248	LEU
1	A	295	LEU
1	A	364	LYS
1	A	465	SER
1	B	135	ARG
1	B	139	SER
1	B	144	GLU
1	B	169	ARG
1	B	175	ARG
1	B	210	LYS
1	B	247	SER
1	B	295	LEU
1	B	301	GLU
1	B	347	ARG
1	B	370	ARG
1	C	169	ARG
1	C	462	SER

Continued on next page...

CONFIDENTIAL VALIDATION REPORT

Continued from previous page...

Mol	Chain	Res	Type
1	C	497	MET
1	D	169	ARG
1	D	295	LEU
1	D	333	ASP
1	D	364	LYS
1	D	378	HIS
1	D	446	ARG
1	D	448	SER

Sometimes sidechains can be flipped to improve hydrogen bonding and reduce clashes. There are no such sidechains identified.

5.3.3 RNA (i)

There are no RNA molecules in this entry.

5.4 Non-standard residues in protein, DNA, RNA chains (i)

There are no non-standard protein/DNA/RNA residues in this entry.

5.5 Carbohydrates (i)

There are no monosaccharides in this entry.

5.6 Ligand geometry (i)

17 ligands are modelled in this entry.

In the following table, the Counts columns list the number of bonds (or angles) for which Mogul statistics could be retrieved, the number of bonds (or angles) that are observed in the model and the number of bonds (or angles) that are defined in the Chemical Component Dictionary. The Link column lists molecule types, if any, to which the group is linked. The Z score for a bond length (or angle) is the number of standard deviations the observed value is removed from the expected value. A bond length (or angle) with $|Z| > 2$ is considered an outlier worth inspection. RMSZ is the root-mean-square of all Z scores of the bond lengths (or angles).

Mol	Type	Chain	Res	Link	Bond lengths			Bond angles		
					Counts	RMSZ	$\#\|Z\| > 2$	Counts	RMSZ	$\#\|Z\| > 2$
3	EDO	B	502	-	3,3,3	0.47	0	2,2,2	0.28	0
3	EDO	C	504	-	3,3,3	0.58	0	2,2,2	0.26	0

CONFIDENTIAL VALIDATION REPORT

Mol	Type	Chain	Res	Link	Bond lengths			Bond angles		
					Counts	RMSZ	#|Z| > 2	Counts	RMSZ	#|Z| > 2
2	LRZ	C	501	-	43,48,48	0.57	1 (2%)	53,68,68	1.19	3 (5%)
3	EDO	A	505	-	3,3,3	0.43	0	2,2,2	0.33	0
2	LRZ	B	501	-	43,48,48	0.59	0	53,68,68	0.96	3 (5%)
4	PG4	C	505	-	12,12,12	0.15	0	11,11,11	0.59	0
3	EDO	C	502	-	3,3,3	0.43	0	2,2,2	0.29	0
3	EDO	A	507	-	3,3,3	0.13	0	2,2,2	0.09	0
3	EDO	A	506	-	3,3,3	0.47	0	2,2,2	0.56	0
3	EDO	B	503	-	3,3,3	0.49	0	2,2,2	0.34	0
2	LRZ	A	501	-	43,48,48	0.54	1 (2%)	53,68,68	0.65	1 (1%)
3	EDO	B	504	-	3,3,3	0.51	0	2,2,2	0.32	0
2	LRZ	D	501	-	43,48,48	0.60	0	53,68,68	0.98	3 (5%)
3	EDO	A	503	-	3,3,3	0.53	0	2,2,2	0.31	0
3	EDO	A	504	-	3,3,3	0.50	0	2,2,2	0.31	0
3	EDO	A	502	-	3,3,3	0.52	0	2,2,2	0.33	0
3	EDO	C	503	-	3,3,3	0.48	0	2,2,2	0.31	0

In the following table, the Chirals column lists the number of chiral outliers, the number of chiral centers analysed, the number of these observed in the model and the number defined in the Chemical Component Dictionary. Similar counts are reported in the Torsion and Rings columns. '-' means no outliers of that kind were identified.

Mol	Type	Chain	Res	Link	Chirals	Torsions	Rings
3	EDO	B	502	-	-	1/1/1/1	-
3	EDO	C	504	-	-	1/1/1/1	-
2	LRZ	C	501	-	-	16/23/43/43	0/5/5/5
3	EDO	A	505	-	-	1/1/1/1	-
2	LRZ	B	501	-	-	14/23/43/43	0/5/5/5
4	PG4	C	505	-	-	4/10/10/10	-
3	EDO	C	502	-	-	0/1/1/1	-
3	EDO	A	507	-	-	1/1/1/1	-
3	EDO	A	506	-	-	0/1/1/1	-
3	EDO	B	503	-	-	0/1/1/1	-
2	LRZ	A	501	-	-	11/23/43/43	0/5/5/5
3	EDO	B	504	-	-	1/1/1/1	-
2	LRZ	D	501	-	-	15/23/43/43	0/5/5/5
3	EDO	A	503	-	-	0/1/1/1	-
3	EDO	A	504	-	-	0/1/1/1	-
3	EDO	A	502	-	-	1/1/1/1	-
3	EDO	C	503	-	-	0/1/1/1	-

All (2) bond length outliers are listed below:

CONFIDENTIAL VALIDATION REPORT

Mol	Chain	Res	Type	Atoms	Z	Observed(Å)	Ideal(Å)
2	C	501	LRZ	C23-N7	-2.04	1.31	1.34
2	A	501	LRZ	C23-N7	-2.01	1.31	1.34

All (10) bond angle outliers are listed below:

Mol	Chain	Res	Type	Atoms	Z	Observed(^{o})	Ideal(^{o})
2	C	501	LRZ	N3-C17-N5	5.51	130.58	120.26
2	C	501	LRZ	N4-C17-N5	-4.48	111.87	120.26
2	B	501	LRZ	N3-C17-N5	3.52	126.86	120.26
2	D	501	LRZ	N3-C17-N5	3.38	126.59	120.26
2	D	501	LRZ	N4-C17-N5	-2.83	114.95	120.26
2	B	501	LRZ	N4-C17-N5	-2.82	114.98	120.26
2	C	501	LRZ	C24-C25-N8	2.41	124.01	120.35
2	D	501	LRZ	C24-C25-N8	2.40	123.99	120.35
2	A	501	LRZ	C24-C25-N8	2.36	123.94	120.35
2	B	501	LRZ	C24-C25-N8	2.34	123.91	120.35

There are no chirality outliers.

All (66) torsion outliers are listed below:

Mol	Chain	Res	Type	Atoms
2	A	501	LRZ	C2-C1-C11-O2
2	A	501	LRZ	C1-C11-O2-C12
2	A	501	LRZ	O3-C11-O2-C12
2	B	501	LRZ	C2-C1-C11-O2
2	B	501	LRZ	C1-C11-O2-C12
2	B	501	LRZ	N4-C17-N3-C16
2	B	501	LRZ	N5-C17-N3-C16
2	B	501	LRZ	N3-C17-N4-C18
2	B	501	LRZ	N5-C17-N4-C18
2	B	501	LRZ	N4-C18-C19-C20
2	B	501	LRZ	N4-C18-C19-O7
2	C	501	LRZ	C1-C11-O2-C12
2	C	501	LRZ	N4-C17-N3-C16
2	C	501	LRZ	N5-C17-N3-C16
2	C	501	LRZ	N3-C17-N4-C18
2	C	501	LRZ	N5-C17-N4-C18
2	D	501	LRZ	C1-C11-O2-C12
2	D	501	LRZ	N4-C17-N3-C16
2	D	501	LRZ	N5-C17-N3-C16
2	D	501	LRZ	N3-C17-N4-C18
2	D	501	LRZ	N5-C17-N4-C18

Continued on next page...

CONFIDENTIAL VALIDATION REPORT

Continued from previous page...

Mol	Chain	Res	Type	Atoms
2	D	501	LRZ	N4-C18-C19-C20
2	D	501	LRZ	N4-C18-C19-O7
2	A	501	LRZ	C6-C1-C11-O2
2	B	501	LRZ	C6-C1-C11-O2
2	C	501	LRZ	C2-C1-C11-O2
2	C	501	LRZ	C2-C1-C11-O3
2	C	501	LRZ	C6-C1-C11-O2
2	C	501	LRZ	C6-C1-C11-O3
2	A	501	LRZ	C2-C1-C11-O3
2	B	501	LRZ	C2-C1-C11-O3
2	A	501	LRZ	N2-C13-N1-C8
2	A	501	LRZ	C6-C1-C11-O3
2	B	501	LRZ	C6-C1-C11-O3
2	B	501	LRZ	O3-C11-O2-C12
2	C	501	LRZ	O3-C11-O2-C12
2	D	501	LRZ	N2-C13-N1-C8
2	A	501	LRZ	O4-C13-N1-C8
2	D	501	LRZ	O4-C13-N1-C8
2	D	501	LRZ	C2-C1-C11-O2
2	D	501	LRZ	C6-C1-C11-O2
2	B	501	LRZ	N2-C13-N1-C8
2	D	501	LRZ	O3-C11-O2-C12
2	B	501	LRZ	O4-C13-N1-C8
2	D	501	LRZ	C6-C1-C11-O3
2	D	501	LRZ	C2-C1-C11-O3
2	A	501	LRZ	N2-C14-C15-C16
2	A	501	LRZ	C7-C8-N1-C13
2	C	501	LRZ	C7-C8-N1-C13
2	A	501	LRZ	C9-C8-N1-C13
3	A	502	EDO	O1-C1-C2-O2
3	A	505	EDO	O1-C1-C2-O2
3	B	502	EDO	O1-C1-C2-O2
2	C	501	LRZ	C9-C8-N1-C13
2	C	501	LRZ	N2-C14-C15-C16
4	C	505	PG4	C4-C3-O2-C2
4	C	505	PG4	C5-C6-O4-C7
2	C	501	LRZ	C15-C16-N3-C17
3	C	504	EDO	O1-C1-C2-O2
4	C	505	PG4	C8-C7-O4-C6
2	C	501	LRZ	C14-C15-C16-N3
4	C	505	PG4	C6-C5-O3-C4
3	B	504	EDO	O1-C1-C2-O2

Continued on next page...

CONFIDENTIAL VALIDATION REPORT

Continued from previous page...

Mol	Chain	Res	Type	Atoms
2	C	501	LRZ	C15-C14-N2-C13
3	A	507	EDO	O1-C1-C2-O2
2	D	501	LRZ	C15-C14-N2-C13

There are no ring outliers.

1 monomer is involved in 1 short contact:

Mol	Chain	Res	Type	Clashes	Symm-Clashes
4	C	505	PG4	1	0

The following is a two-dimensional graphical depiction of Mogul quality analysis of bond lengths, bond angles, torsion angles, and ring geometry for all instances of the Ligand of Interest. In addition, ligands with molecular weight > 250 and outliers as shown on the validation Tables will also be included. For torsion angles, if less then 5% of the Mogul distribution of torsion angles is within 10 degrees of the torsion angle in question, then that torsion angle is considered an outlier. Any bond that is central to one or more torsion angles identified as an outlier by Mogul will be highlighted in the graph. For rings, the root-mean-square deviation (RMSD) between the ring in question and similar rings identified by Mogul is calculated over all ring torsion angles. If the average RMSD is greater than 60 degrees and the minimal RMSD between the ring in question and any Mogul-identified rings is also greater than 60 degrees, then that ring is considered an outlier. The outliers are highlighted in purple. The color gray indicates Mogul did not find sufficient equivalents in the CSD to analyse the geometry.

Ligand LRZ C 501

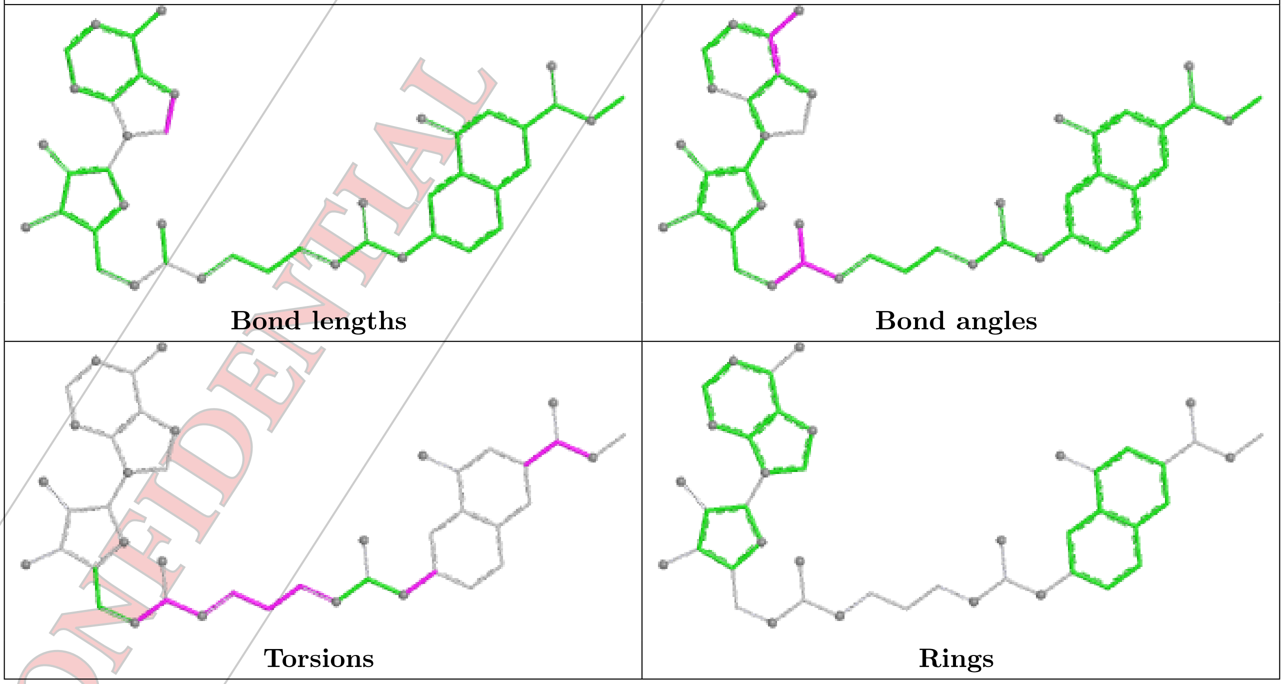

CONFIDENTIAL VALIDATION REPORT

Ligand LRZ B 501

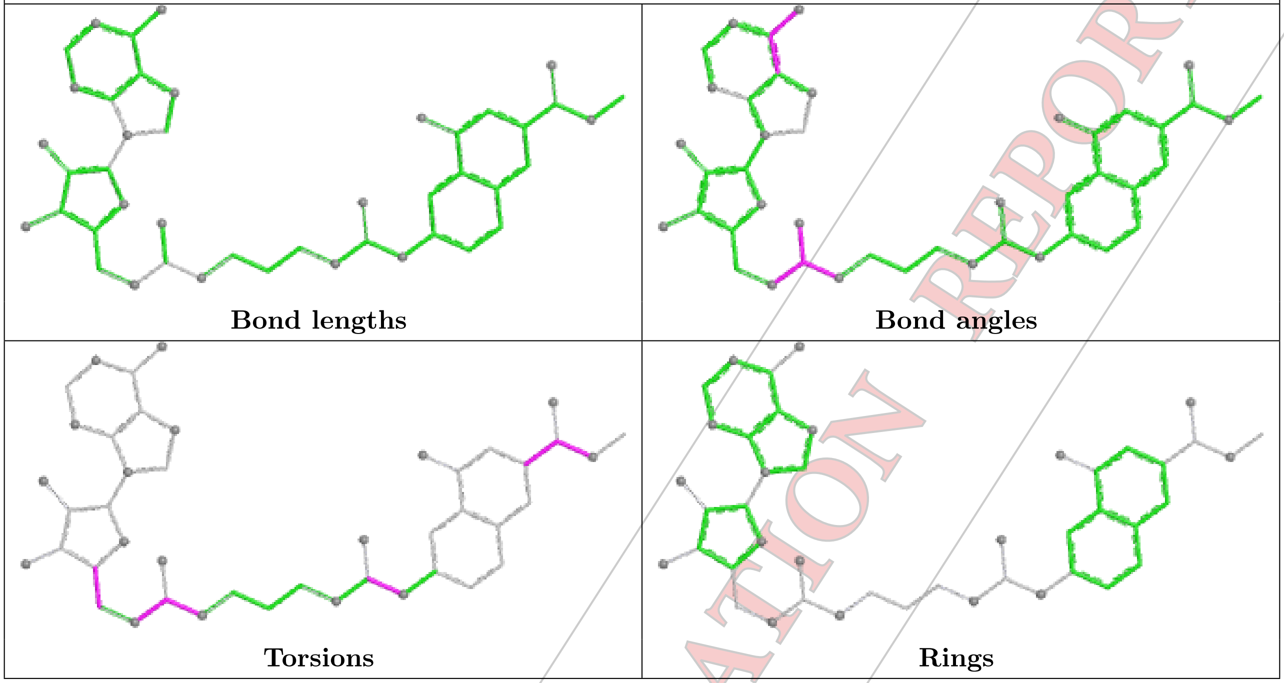

Ligand LRZ A 501

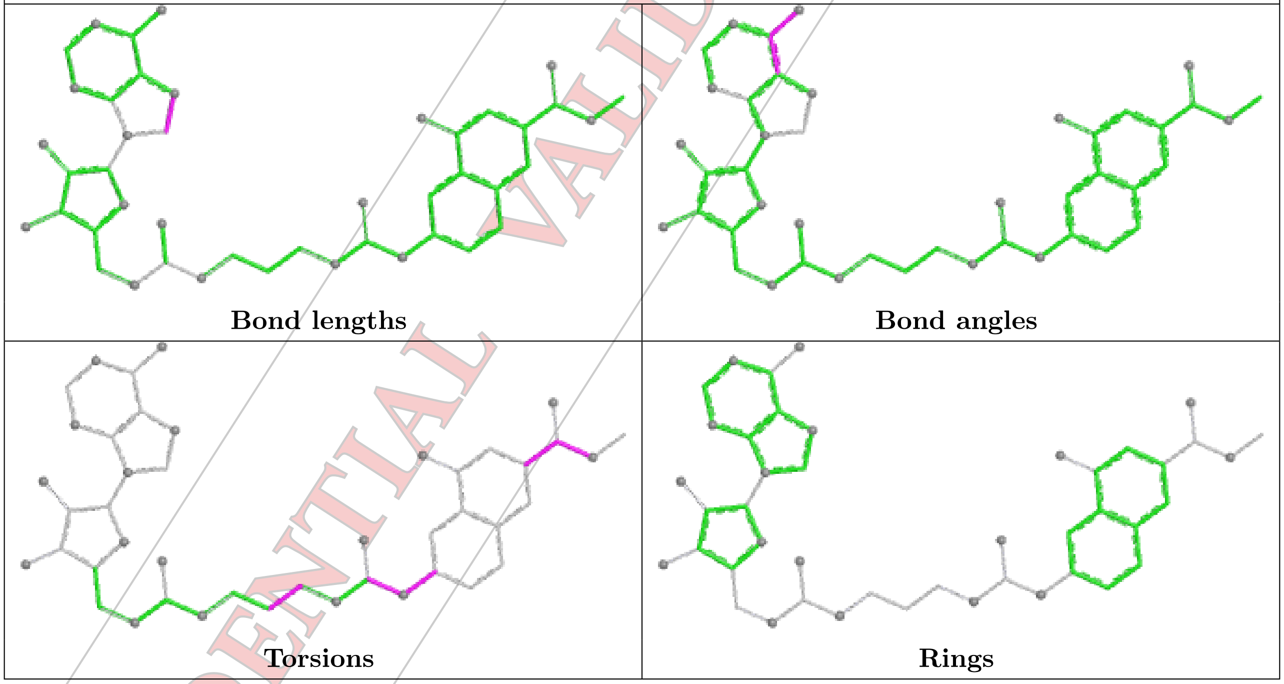

CONFIDENTIAL VALIDATION REPORT

Ligand LRZ D 501	
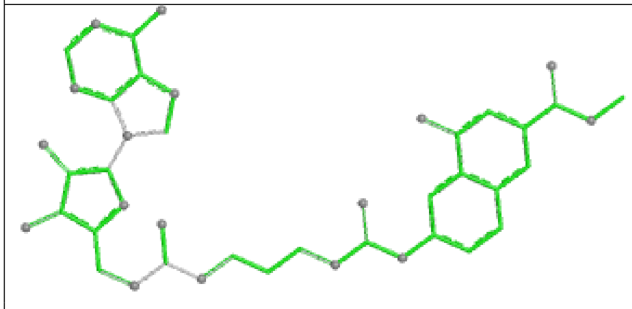 Bond lengths	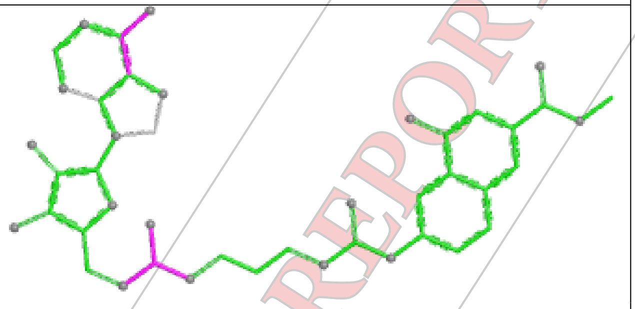 Bond angles
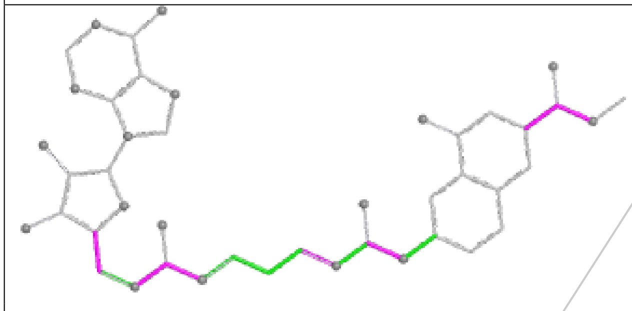 Torsions	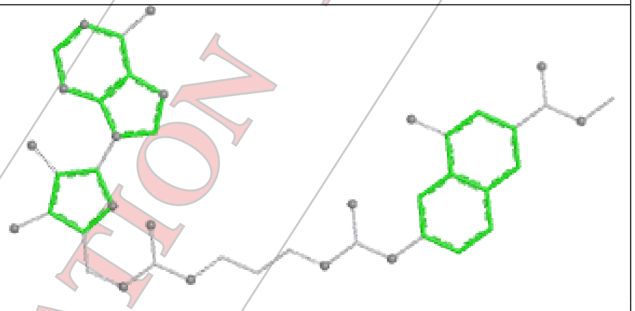 Rings

5.7 Other polymers (i)

There are no such residues in this entry.

5.8 Polymer linkage issues (i)

There are no chain breaks in this entry.

CONFIDENTIAL VALIDATION REPORT

6 Fit of model and data (i)

6.1 Protein, DNA and RNA chains (i)

In the following table, the column labelled '#RSRZ> 2' contains the number (and percentage) of RSRZ outliers, followed by percent RSRZ outliers for the chain as percentile scores relative to all X-ray entries and entries of similar resolution. The OWAB column contains the minimum, median, 95th percentile and maximum values of the occupancy-weighted average B-factor per residue. The column labelled 'Q< 0.9' lists the number of (and percentage) of residues with an average occupancy less than 0.9.

Mol	Chain	Analysed	<RSRZ>	#RSRZ>2			OWAB(Å^2)	Q<0.9
1	A	362/368 (98%)	0.06	7 (1%)	66	64	33, 51, 72, 93	0
1	B	343/368 (93%)	-0.04	6 (1%)	70	68	36, 52, 75, 105	0
1	C	362/368 (98%)	0.06	4 (1%)	80	79	40, 56, 82, 105	0
1	D	343/368 (93%)	-0.07	6 (1%)	70	68	42, 55, 72, 91	0
All	All	1410/1472 (95%)	0.00	23 (1%)	72	70	33, 54, 76, 105	0

All (23) RSRZ outliers are listed below:

Mol	Chain	Res	Type	RSRZ
1	C	140	GLU	4.6
1	C	496	ASN	3.6
1	A	137	VAL	3.3
1	C	497	MET	3.2
1	B	135	ARG	3.1
1	D	135	ARG	2.8
1	A	138	PHE	2.6
1	B	347	ARG	2.5
1	D	140	GLU	2.5
1	A	496	ASN	2.5
1	A	136	SER	2.4
1	D	137	VAL	2.4
1	D	350	MET	2.4
1	B	477	TYR	2.3
1	D	347	ARG	2.3
1	B	144	GLU	2.3
1	D	349	LEU	2.2
1	A	140	GLU	2.1
1	A	428	PHE	2.1
1	B	140	GLU	2.0
1	A	497	MET	2.0

Continued on next page...

CONFIDENTIAL VALIDATION REPORT

Continued from previous page...

Mol	Chain	Res	Type	RSRZ
1	B	283	SER	2.0
1	C	141	ARG	2.0

6.2 Non-standard residues in protein, DNA, RNA chains (i)

There are no non-standard protein/DNA/RNA residues in this entry.

6.3 Carbohydrates (i)

There are no monosaccharides in this entry.

6.4 Ligands (i)

In the following table, the Atoms column lists the number of modelled atoms in the group and the number defined in the chemical component dictionary. The B-factors column lists the minimum, median, 95th percentile and maximum values of B factors of atoms in the group. The column labelled 'Q< 0.9' lists the number of atoms with occupancy less than 0.9.

Mol	Type	Chain	Res	Atoms	RSCC	RSR	B-factors(Å^2)	Q<0.9
3	EDO	C	503	4/4	0.72	0.25	55,73,87,88	0
3	EDO	A	504	4/4	0.75	0.35	65,78,88,92	0
3	EDO	B	504	4/4	0.77	0.21	71,85,91,94	0
3	EDO	C	504	4/4	0.77	0.32	56,69,84,84	0
4	PG4	C	505	13/13	0.79	0.22	73,95,111,112	0
3	EDO	A	502	4/4	0.80	0.13	65,80,96,96	0
3	EDO	A	506	4/4	0.81	0.18	54,74,90,90	0
3	EDO	C	502	4/4	0.82	0.24	64,76,83,83	0
3	EDO	B	503	4/4	0.89	0.21	74,89,105,105	0
2	LRZ	C	501	44/44	0.91	0.17	52,79,103,105	0
2	LRZ	D	501	44/44	0.91	0.21	41,77,103,105	0
2	LRZ	B	501	44/44	0.91	0.20	39,69,98,114	0
3	EDO	A	503	4/4	0.91	0.13	53,71,85,85	0
3	EDO	A	507	4/4	0.92	0.28	56,93,125,125	0
3	EDO	B	502	4/4	0.92	0.30	60,72,87,88	0
3	EDO	A	505	4/4	0.92	0.14	65,78,91,98	0
2	LRZ	A	501	44/44	0.92	0.18	39,76,106,112	0

The following is a graphical depiction of the model fit to experimental electron density of all instances of the Ligand of Interest. In addition, ligands with molecular weight > 250 and outliers as shown on the geometry validation Tables will also be included. Each fit is shown from different orientation to approximate a three-dimensional view.

CONFIDENTIAL VALIDATION REPORT

Electron density around LRZ C 501:

$2mF_o$-DF_c (at 0.7 rmsd) in gray
mF_o-DF_c (at 3 rmsd) in purple (negative)
and green (positive)

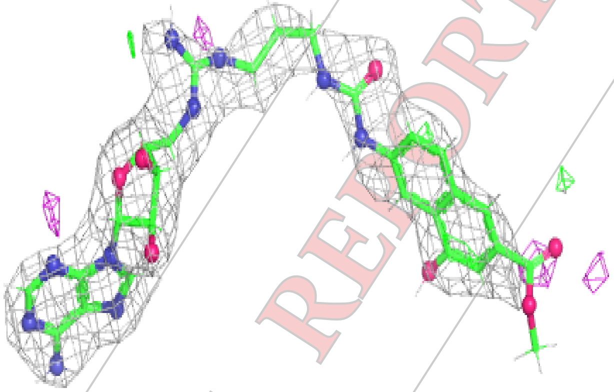

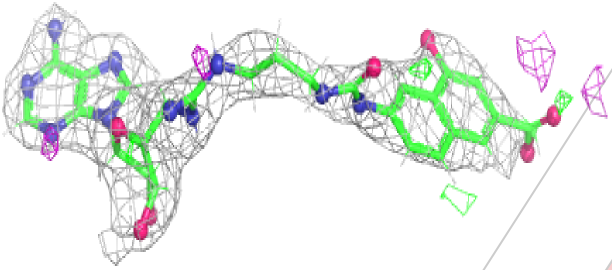

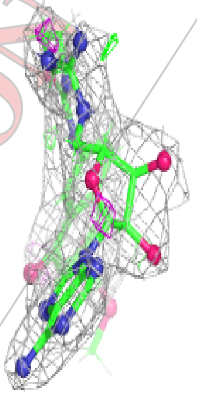

Electron density around LRZ D 501:

$2mF_o$-DF_c (at 0.7 rmsd) in gray
mF_o-DF_c (at 3 rmsd) in purple (negative)
and green (positive)

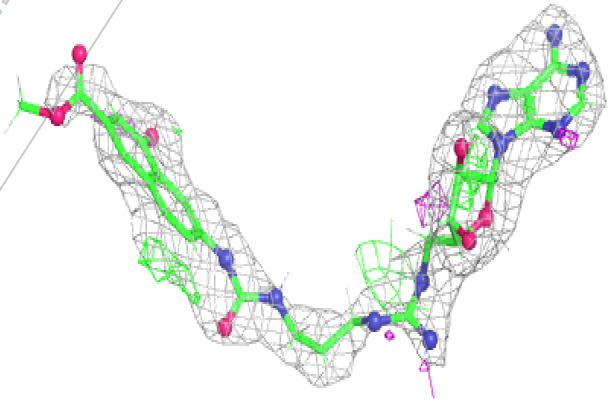

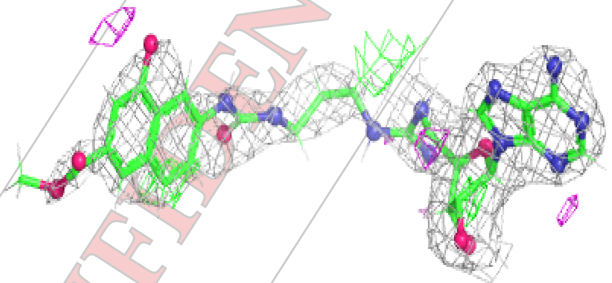

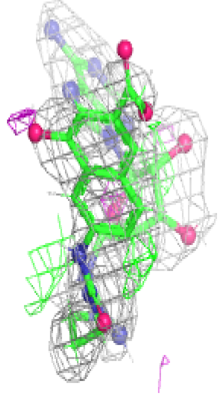

CONFIDENTIAL VALIDATION REPORT

Electron density around LRZ B 501:

$2mF_o$-DF_c (at 0.7 rmsd) in gray
mF_o-DF_c (at 3 rmsd) in purple (negative)
and green (positive)

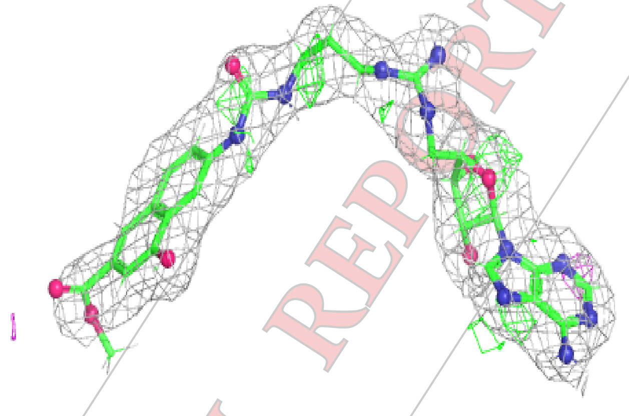

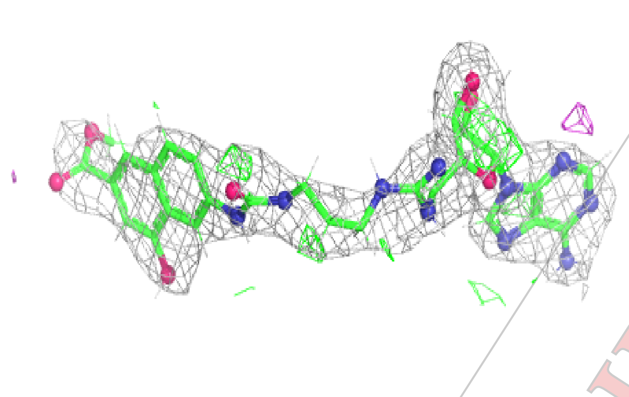

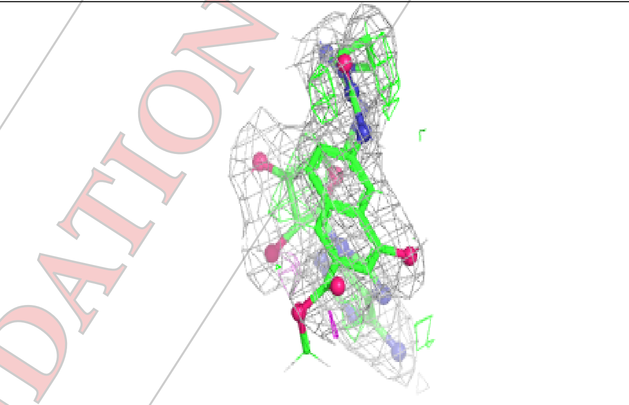

Electron density around LRZ A 501:

$2mF_o$-DF_c (at 0.7 rmsd) in gray
mF_o-DF_c (at 3 rmsd) in purple (negative)
and green (positive)

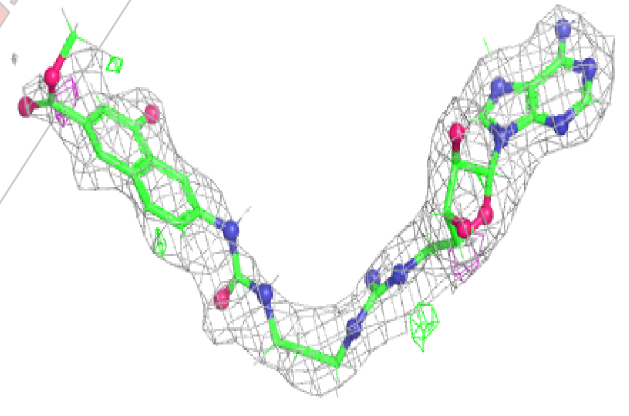

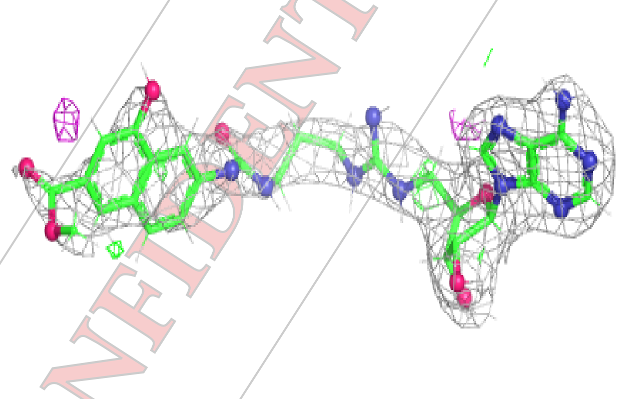

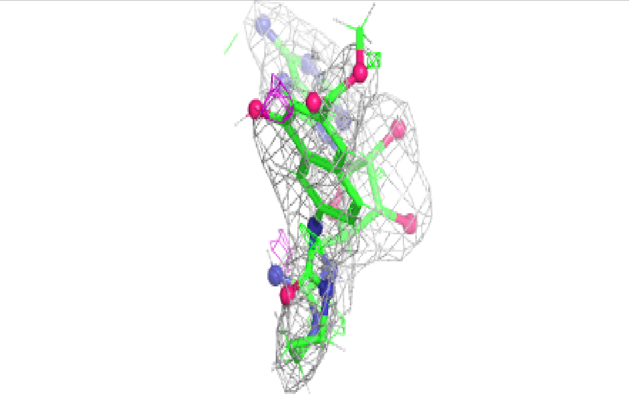

CONFIDENTIAL VALIDATION REPORT

6.5 Other polymers ⓘ

There are no such residues in this entry.

CONFIDENTIAL VALIDATION REPORT
