# Supplementary material for: Turning Nonselective Inhibitors of Type I Protein Arginine Methyltransferases into Potent and Selective Inhibitors of Protein Arginine Methyltransferase 4 through a Deconstruction–Reconstruction and Fragment-Growing Approach
Source: J Med Chem. 2022 Apr 28;65(17):11574–606. doi: 10.1021/acs.jmedchem.2c00252 (PMC9469100; doi:10.1021/acs.jmedchem.2c00252)
Supplement: Supplementary file 4 — jm2c00252_si_004.pdf [file jm2c00252_si_004.pdf]

Full wwPDB X-ray Structure Validation Report (i)

Sep 27, 2021 – 10:49 pm BST

PDB ID	:	7PPQ
Title	:	CARM1 in complex with EML736
Deposited on	:	2021-09-14
Resolution	:	2.10 Å(reported)

This is a Full wwPDB X-ray Structure Validation Report.

This report is produced by the wwPDB biocuration pipeline after annotation of the structure.

We welcome your comments at *validation@mail.wwpdb.org*
A user guide is available at
https://www.wwpdb.org/validation/2017/XrayValidationReportHelp
with specific help available everywhere you see the (i) symbol.

CONFIDENTIAL VALIDATION REPORT

The following versions of software and data (see references (i)) were used in the production of this report:

MolProbity	:	4.02b-467
Mogul	:	1.8.5 (274361), CSD as541be (2020)
Xtriage (Phenix)	:	1.13
EDS	:	2.23.2
buster-report	:	1.1.7 (2018)
Percentile statistics	:	20191225.v01 (using entries in the PDB archive December 25th 2019)
Refmac	:	5.8.0267
CCP4	:	7.1.010 (Gargrove)
Ideal geometry (proteins)	:	Engh & Huber (2001)
Ideal geometry (DNA, RNA)	:	Parkinson et al. (1996)
Validation Pipeline (wwPDB-VP)	:	2.23.2

1 Overall quality at a glance (i)

The following experimental techniques were used to determine the structure:
X-RAY DIFFRACTION

The reported resolution of this entry is 2.10 Å.

Percentile scores (ranging between 0-100) for global validation metrics of the entry are shown in the following graphic. The table shows the number of entries on which the scores are based.

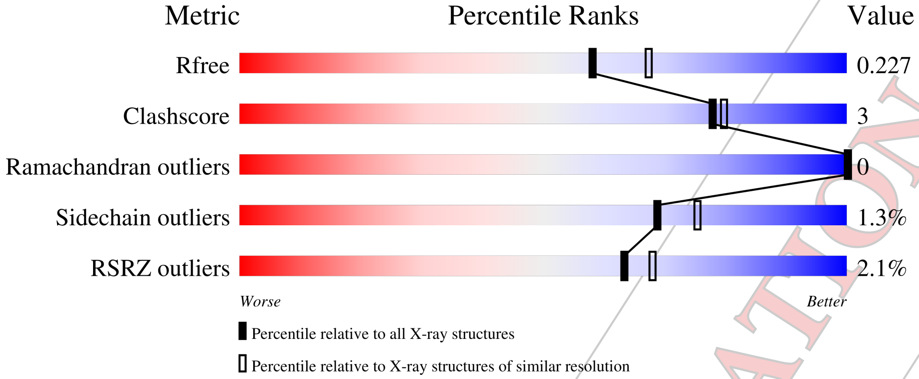

Metric	Whole archive (#Entries)	Similar resolution (#Entries, resolution range(Å))
R_{free}	130704	5197 (2.10-2.10)
Clashscore	141614	5710 (2.10-2.10)
Ramachandran outliers	138981	5647 (2.10-2.10)
Sidechain outliers	138945	5648 (2.10-2.10)
RSRZ outliers	127900	5083 (2.10-2.10)

The table below summarises the geometric issues observed across the polymeric chains and their fit to the electron density. The red, orange, yellow and green segments of the lower bar indicate the fraction of residues that contain outliers for >=3, 2, 1 and 0 types of geometric quality criteria respectively. A grey segment represents the fraction of residues that are not modelled. The numeric value for each fraction is indicated below the corresponding segment, with a dot representing fractions <=5% The upper red bar (where present) indicates the fraction of residues that have poor fit to the electron density. The numeric value is given above the bar.

Mol	Chain	Length	Quality of chain
1	A	361	2%<br>92% • 5%
1	B	361	2%<br>89% 6% • 5%
1	C	361	2%<br>89% 6% 5%
1	D	361	2%<br>85% 9% • 5%

CONFIDENTIAL VALIDATION REPORT

2 Entry composition (i)

There are 4 unique types of molecules in this entry. The entry contains 22808 atoms, of which 10981 are hydrogens and 0 are deuteriums.

In the tables below, the ZeroOcc column contains the number of atoms modelled with zero occupancy, the AltConf column contains the number of residues with at least one atom in alternate conformation and the Trace column contains the number of residues modelled with at most 2 atoms.

- Molecule 1 is a protein called Histone-arginine methyltransferase CARM1.

Mol	Chain	Residues	Atoms						ZeroOcc	AltConf	Trace
			Total	C	H	N	O	S			
1	A	343	5450	1776	2699	454	507	14	0	0	0
1	B	343	5460	1778	2705	457	506	14	0	0	0
1	C	343	5450	1776	2699	454	507	14	0	0	0
1	D	342	5436	1772	2692	453	505	14	0	0	0

There are 12 discrepancies between the modelled and reference sequences:

Chain	Residue	Modelled	Actual	Comment	Reference
A	127	GLY	-	expression tag	UNP Q9WVG6
A	128	HIS	-	expression tag	UNP Q9WVG6
A	129	MET	-	expression tag	UNP Q9WVG6
B	127	GLY	-	expression tag	UNP Q9WVG6
B	128	HIS	-	expression tag	UNP Q9WVG6
B	129	MET	-	expression tag	UNP Q9WVG6
C	127	GLY	-	expression tag	UNP Q9WVG6
C	128	HIS	-	expression tag	UNP Q9WVG6
C	129	MET	-	expression tag	UNP Q9WVG6
D	127	GLY	-	expression tag	UNP Q9WVG6
D	128	HIS	-	expression tag	UNP Q9WVG6
D	129	MET	-	expression tag	UNP Q9WVG6

- Molecule 2 is methyl 6-[5-[[{N}-[[(2 {R},3 {S},4 {R},5 {R})-5-(6-aminopurin-9-yl)-3,4-bis(oxidanyl)oxolan-2-yl]methyl]carbamimidoyl]amino]pentylcarbamoylamino]-4-oxidanyl-naph thalene-2-carboxylate (three-letter code: LSK) (formula: $C_{29}H_{36}N_{10}O_7$) (labeled as "Ligand of Interest" by depositor).

CONFIDENTIAL VALIDATION REPORT

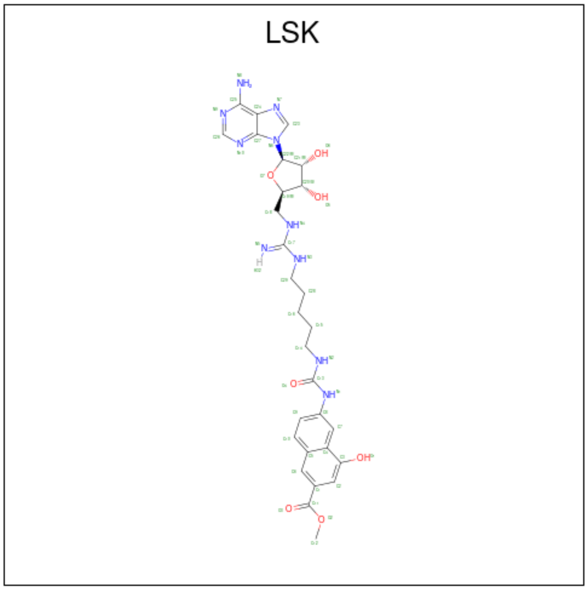

Mol	Chain	Residues	Atoms	ZeroOcc	AltConf
2	A	1	Total C H N O<br>82 29 36 10 7	0	0
2	B	1	Total C H N O<br>82 29 36 10 7	0	0
2	C	1	Total C H N O<br>82 29 36 10 7	0	0
2	D	1	Total C H N O<br>82 29 36 10 7	0	0

- Molecule 3 is 1,2-ETHANEDIOL (three-letter code: EDO) (formula: $C_2H_6O_2$).

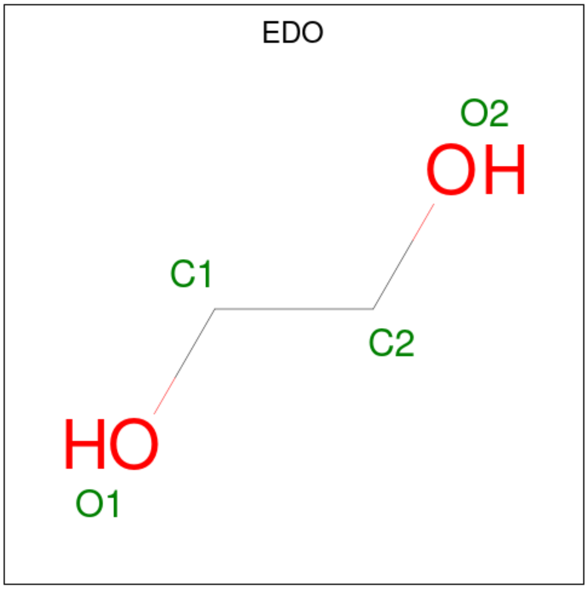

VALIDATION REPORT

Mol	Chain	Residues	Atoms	ZeroOcc	AltConf
3	A	1	Total C H O<br>10 2 6 2	0	0
3	A	1	Total C H O<br>10 2 6 2	0	0
3	B	1	Total C H O<br>10 2 6 2	0	0
3	B	1	Total C H O<br>10 2 6 2	0	0
3	C	1	Total C H O<br>10 2 6 2	0	0
3	C	1	Total C H O<br>10 2 6 2	0	0
3	D	1	Total C H O<br>10 2 6 2	0	0

- Molecule 4 is water.

Mol	Chain	Residues	Atoms	ZeroOcc	AltConf
4	A	175	Total O<br>175 175	0	0
4	B	124	Total O<br>124 124	0	0
4	C	172	Total O<br>172 172	0	0
4	D	143	Total O<br>143 143	0	0

CONFIDENTIAL VALIDATION REPORT

3 Residue-property plots (i)

These plots are drawn for all protein, RNA, DNA and oligosaccharide chains in the entry. The first graphic for a chain summarises the proportions of the various outlier classes displayed in the second graphic. The second graphic shows the sequence view annotated by issues in geometry and electron density. Residues are color-coded according to the number of geometric quality criteria for which they contain at least one outlier: green = 0, yellow = 1, orange = 2 and red = 3 or more. A red dot above a residue indicates a poor fit to the electron density (RSRZ > 2). Stretches of 2 or more consecutive residues without any outlier are shown as a green connector. Residues present in the sample, but not in the model, are shown in grey.

- Molecule 1: Histone-arginine methyltransferase CARM1

Chain A:

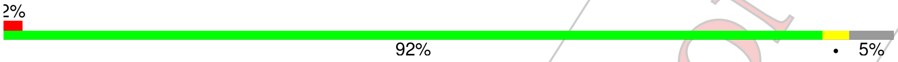

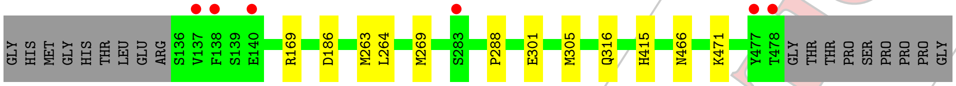

- Molecule 1: Histone-arginine methyltransferase CARM1

Chain B:

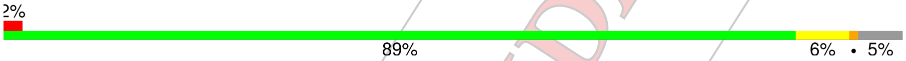

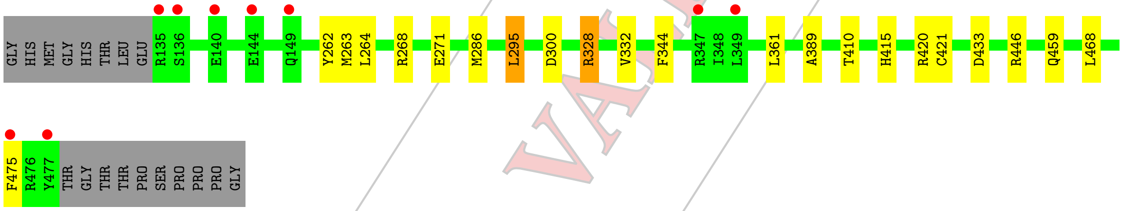

- Molecule 1: Histone-arginine methyltransferase CARM1

Chain C:

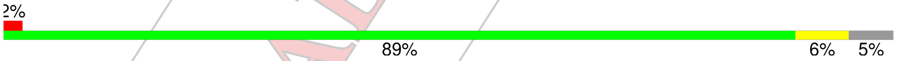

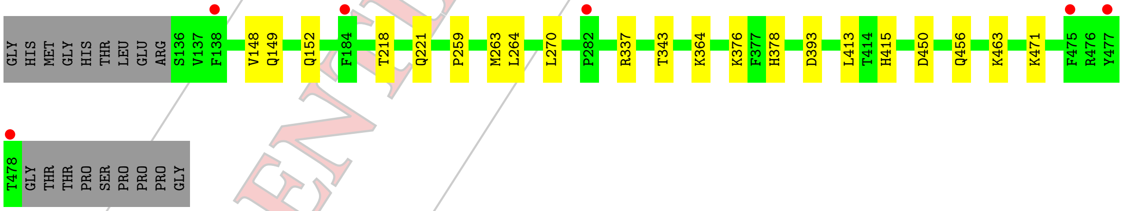

- Molecule 1: Histone-arginine methyltransferase CARM1

Chain D:

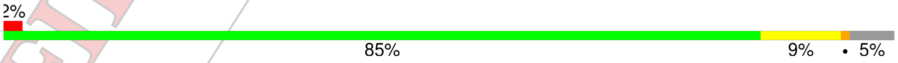

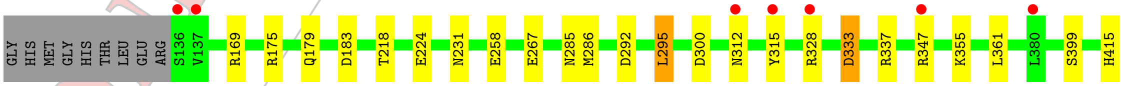

CONFIDENTIAL VALIDATION REPORT

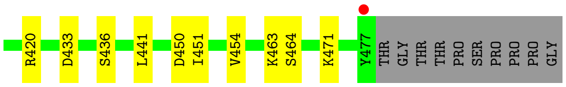

CONFIDENTIAL VALIDATION REPORT

4 Data and refinement statistics (i)

Property	Value	Source
Space group	P 21 21 2	Depositor
Cell constants<br>a, b, c, α, β, γ	74.79Å 98.53Å 206.89Å<br>90.00° 90.00° 90.00°	Depositor
Resolution (Å)	45.80 – 2.10<br>45.80 – 2.10	Depositor<br>EDS
% Data completeness<br>(in resolution range)	98.9 (45.80-2.10)<br>99.0 (45.80-2.10)	Depositor<br>EDS
R_{merge}	0.09	Depositor
R_{sym}	(Not available)	Depositor
$< I/\sigma(I) >$ [1]	1.56 (at 2.10Å)	Xtriage
Refinement program	PHENIX 1.19.2_4158	Depositor
R, R_{free}	0.194 , 0.228<br>0.194 , 0.227	Depositor<br>DCC
R_{free} test set	4457 reflections (4.97%)	wwPDB-VP
Wilson B-factor (Å^2)	36.7	Xtriage
Anisotropy	0.317	Xtriage
Bulk solvent k_{sol}(e/Å^3), B_{sol}(Å^2)	(Not available) , (Not available)	EDS
L-test for twinning[2]	$< \|L\| > = 0.50$, $< L^2 > = 0.33$	Xtriage
Estimated twinning fraction	No twinning to report.	Xtriage
F_o,F_c correlation	0.96	EDS
Total number of atoms	22808	wwPDB-VP
Average B, all atoms (Å^2)	49.0	wwPDB-VP

Xtriage's analysis on translational NCS is as follows: *The analyses of the Patterson function reveals a significant off-origin peak that is 37.40 % of the origin peak, indicating pseudo-translational symmetry. The chance of finding a peak of this or larger height randomly in a structure without pseudo-translational symmetry is equal to 4.3111e-04. The detected translational NCS is most likely also responsible for the elevated intensity ratio.*

[1] Intensities estimated from amplitudes.
[2] Theoretical values of $< |L| >$, $< L^2 >$ for acentric reflections are 0.5, 0.333 respectively for untwinned datasets, and 0.375, 0.2 for perfectly twinned datasets.

CONFIDENTIAL VALIDATION REPORT

5 Model quality (i)

5.1 Standard geometry (i)

Bond lengths and bond angles in the following residue types are not validated in this section: EDO, LSK

The Z score for a bond length (or angle) is the number of standard deviations the observed value is removed from the expected value. A bond length (or angle) with $|Z| > 5$ is considered an outlier worth inspection. RMSZ is the root-mean-square of all Z scores of the bond lengths (or angles).

Mol	Chain	Bond lengths		Bond angles	
		RMSZ	$\#\|Z\| > 5$	RMSZ	$\#\|Z\| > 5$
1	A	0.28	0/2821	0.46	0/3823
1	B	0.28	0/2825	0.45	0/3827
1	C	0.27	0/2821	0.47	0/3823
1	D	0.26	0/2814	0.45	0/3813
All	All	0.27	0/11281	0.46	0/15286

There are no bond length outliers.

There are no bond angle outliers.

There are no chirality outliers.

There are no planarity outliers.

5.2 Too-close contacts (i)

In the following table, the Non-H and H(model) columns list the number of non-hydrogen atoms and hydrogen atoms in the chain respectively. The H(added) column lists the number of hydrogen atoms added and optimized by MolProbity. The Clashes column lists the number of clashes within the asymmetric unit, whereas Symm-Clashes lists symmetry-related clashes.

Mol	Chain	Non-H	H(model)	H(added)	Clashes	Symm-Clashes
1	A	2751	2699	2699	5	0
1	B	2755	2705	2705	18	0
1	C	2751	2699	2699	13	0
1	D	2744	2692	2692	26	0
2	A	46	36	0	1	0
2	B	46	36	0	0	0
2	C	46	36	0	0	0
2	D	46	36	0	0	0
3	A	8	12	12	1	0

Continued on next page...

CONFIDENTIAL VALIDATION REPORT

Continued from previous page...

Mol	Chain	Non-H	H(model)	H(added)	Clashes	Symm-Clashes
3	B	8	12	12	0	0
3	C	8	12	12	0	0
3	D	4	6	6	0	0
4	A	175	0	0	3	0
4	B	124	0	0	1	0
4	C	172	0	0	4	1
4	D	143	0	0	6	0
All	All	11827	10981	10837	63	1

The all-atom clashscore is defined as the number of clashes found per 1000 atoms (including hydrogen atoms). The all-atom clashscore for this structure is 3.

All (63) close contacts within the same asymmetric unit are listed below, sorted by their clash magnitude.

Atom-1	Atom-2	Interatomic distance (Å)	Clash overlap (Å)
1:D:433:ASP:OD2	4:D:601:HOH:O	1.90	0.88
1:B:410:THR:OG1	4:B:601:HOH:O	1.96	0.83
1:C:393:ASP:OD2	4:C:601:HOH:O	2.02	0.77
1:D:267:GLU:OE1	4:D:602:HOH:O	2.03	0.76
1:D:224:GLU:HA	1:D:224:GLU:OE1	1.85	0.74
1:C:148:VAL:O	1:C:152:GLN:HG3	1.89	0.73
1:D:450:ASP:OD1	4:D:603:HOH:O	2.06	0.73
1:D:231:ASN:O	4:D:604:HOH:O	2.08	0.71
1:D:218:THR:OG1	4:D:605:HOH:O	2.09	0.70
1:B:300:ASP:OD2	1:B:420:ARG:NH2	2.29	0.65
1:B:459:GLN:N	1:B:459:GLN:OE1	2.31	0.62
1:B:328:ARG:NH1	1:B:332:VAL:HG21	2.15	0.61
1:D:285:ASN:HB2	4:D:611:HOH:O	2.03	0.59
1:D:175:ARG:HD2	1:D:179:GLN:HE22	1.68	0.57
1:D:300:ASP:OD2	1:D:420:ARG:NH2	2.38	0.57
1:C:218:THR:O	1:C:221:GLN:HG2	2.05	0.57
3:A:502:EDO:O2	4:A:601:HOH:O	2.00	0.56
1:C:450:ASP:OD2	4:C:602:HOH:O	2.18	0.56
1:A:471:LYS:NZ	4:A:607:HOH:O	2.37	0.55
1:C:337:ARG:HD3	4:C:755:HOH:O	2.07	0.53
1:D:312:ASN:HA	1:D:315:TYR:CD1	2.43	0.53
1:B:328:ARG:CZ	1:B:332:VAL:HG21	2.39	0.53
1:D:183:ASP:OD1	1:D:399:SER:OG	2.15	0.52
1:B:328:ARG:O	1:B:328:ARG:HD3	2.09	0.52
1:B:421:CYS:HB3	1:B:468:LEU:HD11	1.92	0.51
1:C:471:LYS:HA	4:C:656:HOH:O	2.11	0.50

Continued on next page...

CONFIDENTIAL VALIDATION REPORT

Continued from previous page...

Atom-1	Atom-2	Interatomic distance (Å)	Clash overlap (Å)
1:B:421:CYS:SG	1:B:468:LEU:HD13	2.52	0.50
1:B:262:TYR:HH	1:B:475:PHE:HZ	1.53	0.49
1:D:292:ASP:CG	1:D:355:LYS:HD2	2.32	0.49
1:D:454:VAL:CG1	1:D:463:LYS:NZ	2.76	0.49
1:B:433:ASP:OD1	1:B:459:GLN:HG2	2.13	0.48
1:D:441:LEU:HD22	1:D:451:ILE:HG12	1.95	0.48
1:D:224:GLU:OE1	1:D:224:GLU:CA	2.55	0.48
1:D:463:LYS:HG2	1:D:464:SER:N	2.28	0.48
1:D:295:LEU:HD23	1:D:295:LEU:N	2.29	0.47
1:D:333:ASP:O	1:D:337:ARG:HG3	2.15	0.47
1:B:421:CYS:HB3	1:B:468:LEU:CD1	2.45	0.47
1:B:446:ARG:HG3	1:B:446:ARG:HH11	1.79	0.47
1:D:312:ASN:HA	1:D:315:TYR:CE1	2.50	0.46
1:D:454:VAL:HG13	1:D:463:LYS:HZ2	1.82	0.45
1:B:295:LEU:HA	1:B:389:ALA:O	2.16	0.45
1:B:263:MET:O	1:B:264:LEU:HB3	2.17	0.45
1:B:328:ARG:HD3	1:B:328:ARG:C	2.38	0.43
1:C:376:LYS:HB3	1:C:376:LYS:HE2	1.73	0.43
1:C:376:LYS:HE3	1:C:378:HIS:CE1	2.54	0.43
1:A:316:GLN:HB2	4:A:698:HOH:O	2.19	0.42
1:D:454:VAL:HG13	1:D:463:LYS:NZ	2.35	0.42
1:C:263:MET:O	1:C:264:LEU:HB3	2.19	0.42
1:C:343:THR:HB	1:C:413:LEU:CD1	2.50	0.42
1:C:456:GLN:HB3	1:C:463:LYS:HG2	2.00	0.42
1:C:259:PRO:HG2	1:C:270:LEU:HD23	2.02	0.42
1:C:343:THR:HB	1:C:413:LEU:HD11	2.01	0.42
1:B:344:PHE:CD2	1:B:420:ARG:HD2	2.54	0.41
1:D:286:MET:HG3	1:D:361:LEU:HD23	2.02	0.41
1:A:263:MET:O	1:A:264:LEU:HB3	2.20	0.41
1:B:286:MET:HG2	1:B:361:LEU:HD23	2.02	0.41
1:D:169:ARG:HD2	1:D:258:GLU:OE2	2.20	0.41
1:D:315:TYR:CE1	1:D:328:ARG:HG2	2.56	0.41
1:A:269:MET:SD	2:A:501:LSK:C23	3.09	0.41
1:A:301:GLU:O	1:A:305:MET:HG3	2.21	0.41
1:D:471:LYS:HD2	1:D:471:LYS:HA	1.92	0.41
1:D:315:TYR:CE1	1:D:328:ARG:CG	3.05	0.40
1:B:268:ARG:HD2	1:B:271:GLU:OE2	2.21	0.40

All (1) symmetry-related close contacts are listed below. The label for Atom-2 includes the symmetry operator and encoded unit-cell translations to be applied.

CONFIDENTIAL VALIDATION REPORT

Atom-1	Atom-2	Interatomic distance (Å)	Clash overlap (Å)
4:C:647:HOH:O	4:C:647:HOH:O[2_655]	2.14	0.06

5.3 Torsion angles (i)

5.3.1 Protein backbone (i)

In the following table, the Percentiles column shows the percent Ramachandran outliers of the chain as a percentile score with respect to all X-ray entries followed by that with respect to entries of similar resolution.

The Analysed column shows the number of residues for which the backbone conformation was analysed, and the total number of residues.

Mol	Chain	Analysed	Favoured	Allowed	Outliers	Percentiles	
1	A	341/361 (94%)	329 (96%)	12 (4%)	0	100	100
1	B	341/361 (94%)	331 (97%)	10 (3%)	0	100	100
1	C	341/361 (94%)	330 (97%)	11 (3%)	0	100	100
1	D	340/361 (94%)	328 (96%)	12 (4%)	0	100	100
All	All	1363/1444 (94%)	1318 (97%)	45 (3%)	0	100	100

There are no Ramachandran outliers to report.

5.3.2 Protein sidechains (i)

In the following table, the Percentiles column shows the percent sidechain outliers of the chain as a percentile score with respect to all X-ray entries followed by that with respect to entries of similar resolution.

The Analysed column shows the number of residues for which the sidechain conformation was analysed, and the total number of residues.

Mol	Chain	Analysed	Rotameric	Outliers	Percentiles	
1	A	299/313 (96%)	294 (98%)	5 (2%)	60	67
1	B	299/313 (96%)	296 (99%)	3 (1%)	76	82
1	C	299/313 (96%)	296 (99%)	3 (1%)	76	82
1	D	298/313 (95%)	293 (98%)	5 (2%)	60	67
All	All	1195/1252 (95%)	1179 (99%)	16 (1%)	69	75

All (16) residues with a non-rotameric sidechain are listed below:

CONFIDENTIAL VALIDATION REPORT

Mol	Chain	Res	Type
1	A	169	ARG
1	A	186	ASP
1	A	288	PRO
1	A	415	HIS
1	A	466	ASN
1	B	295	LEU
1	B	328	ARG
1	B	415	HIS
1	C	149	GLN
1	C	364	LYS
1	C	415	HIS
1	D	295	LEU
1	D	333	ASP
1	D	347	ARG
1	D	415	HIS
1	D	436	SER

Sometimes sidechains can be flipped to improve hydrogen bonding and reduce clashes. All (7) such sidechains are listed below:

Mol	Chain	Res	Type
1	A	466	ASN
1	B	152	GLN
1	B	222	HIS
1	C	221	GLN
1	C	378	HIS
1	C	415	HIS
1	D	179	GLN

5.3.3 RNA (i)

There are no RNA molecules in this entry.

5.4 Non-standard residues in protein, DNA, RNA chains (i)

There are no non-standard protein/DNA/RNA residues in this entry.

5.5 Carbohydrates (i)

There are no monosaccharides in this entry.

CONFIDENTIAL VALIDATION REPORT

5.6 Ligand geometry (i)

11 ligands are modelled in this entry.

In the following table, the Counts columns list the number of bonds (or angles) for which Mogul statistics could be retrieved, the number of bonds (or angles) that are observed in the model and the number of bonds (or angles) that are defined in the Chemical Component Dictionary. The Link column lists molecule types, if any, to which the group is linked. The Z score for a bond length (or angle) is the number of standard deviations the observed value is removed from the expected value. A bond length (or angle) with $|Z| > 2$ is considered an outlier worth inspection. RMSZ is the root-mean-square of all Z scores of the bond lengths (or angles).

Mol	Type	Chain	Res	Link	Bond lengths			Bond angles		
					Counts	RMSZ	$\#\|Z\| > 2$	Counts	RMSZ	$\#\|Z\| > 2$
2	LSK	C	501	-	45,50,50	0.61	1 (2%)	55,70,70	0.97	4 (7%)
2	LSK	B	501	-	45,50,50	0.58	1 (2%)	55,70,70	0.79	1 (1%)
3	EDO	A	503	-	3,3,3	0.44	0	2,2,2	0.34	0
2	LSK	D	501	-	45,50,50	0.64	0	55,70,70	0.85	2 (3%)
3	EDO	D	502	-	3,3,3	0.45	0	2,2,2	0.28	0
3	EDO	A	502	-	3,3,3	0.43	0	2,2,2	0.29	0
3	EDO	B	503	-	3,3,3	0.46	0	2,2,2	0.27	0
3	EDO	B	502	-	3,3,3	0.46	0	2,2,2	0.30	0
3	EDO	C	502	-	3,3,3	0.46	0	2,2,2	0.30	0
2	LSK	A	501	-	45,50,50	0.63	1 (2%)	55,70,70	0.79	2 (3%)
3	EDO	C	503	-	3,3,3	0.44	0	2,2,2	0.28	0

In the following table, the Chirals column lists the number of chiral outliers, the number of chiral centers analysed, the number of these observed in the model and the number defined in the Chemical Component Dictionary. Similar counts are reported in the Torsion and Rings columns. '-' means no outliers of that kind were identified.

Mol	Type	Chain	Res	Link	Chirals	Torsions	Rings
2	LSK	C	501	-	-	17/25/45/45	0/5/5/5
2	LSK	B	501	-	-	16/25/45/45	0/5/5/5
3	EDO	A	503	-	-	0/1/1/1	-
2	LSK	D	501	-	-	12/25/45/45	0/5/5/5
3	EDO	D	502	-	-	1/1/1/1	-
3	EDO	A	502	-	-	0/1/1/1	-
3	EDO	B	503	-	-	1/1/1/1	-
3	EDO	B	502	-	-	0/1/1/1	-
3	EDO	C	502	-	-	0/1/1/1	-
2	LSK	A	501	-	-	13/25/45/45	0/5/5/5
3	EDO	C	503	-	-	0/1/1/1	-

CONFIDENTIAL VALIDATION REPORT

All (3) bond length outliers are listed below:

Mol	Chain	Res	Type	Atoms	Z	Observed(Å)	Ideal(Å)
2	A	501	LSK	C23-N7	-2.09	1.31	1.34
2	B	501	LSK	C23-N7	-2.09	1.31	1.34
2	C	501	LSK	C23-N7	-2.07	1.31	1.34

All (9) bond angle outliers are listed below:

Mol	Chain	Res	Type	Atoms	Z	Observed(^{o})	Ideal(^{o})
2	C	501	LSK	N4-C17-N5	2.89	125.67	120.26
2	C	501	LSK	N3-C17-N5	-2.81	115.00	120.26
2	D	501	LSK	C24-C25-N8	2.40	123.99	120.35
2	A	501	LSK	C24-C25-N8	2.35	123.93	120.35
2	C	501	LSK	C24-C25-N8	2.31	123.87	120.35
2	B	501	LSK	C24-C25-N8	2.30	123.85	120.35
2	A	501	LSK	C19-C18-N4	2.23	117.09	112.69
2	D	501	LSK	O1-C3-C4	2.11	118.95	116.31
2	C	501	LSK	C19-C18-N4	2.03	116.68	112.69

There are no chirality outliers.

All (60) torsion outliers are listed below:

Mol	Chain	Res	Type	Atoms
2	A	501	LSK	N3-C17-N4-C18
2	A	501	LSK	N5-C17-N4-C18
2	A	501	LSK	C1-C11-O2-C12
2	A	501	LSK	O3-C11-O2-C12
2	B	501	LSK	N4-C18-C19-C20
2	B	501	LSK	N4-C18-C19-O7
2	B	501	LSK	N3-C17-N4-C18
2	B	501	LSK	N5-C17-N4-C18
2	B	501	LSK	N2-C13-N1-C8
2	B	501	LSK	O4-C13-N1-C8
2	B	501	LSK	C1-C11-O2-C12
2	C	501	LSK	N4-C17-N3-C29
2	C	501	LSK	N5-C17-N3-C29
2	C	501	LSK	N3-C17-N4-C18
2	C	501	LSK	N5-C17-N4-C18
2	C	501	LSK	C6-C1-C11-O2
2	D	501	LSK	N4-C18-C19-O7
2	D	501	LSK	N4-C17-N3-C29
2	D	501	LSK	N5-C17-N3-C29
2	D	501	LSK	C2-C1-C11-O2

Continued on next page...

CONFIDENTIAL VALIDATION REPORT

Continued from previous page...

Mol	Chain	Res	Type	Atoms
2	D	501	LSK	C1-C11-O2-C12
2	A	501	LSK	C2-C1-C11-O3
2	A	501	LSK	C6-C1-C11-O2
2	A	501	LSK	C6-C1-C11-O3
2	B	501	LSK	C2-C1-C11-O2
2	B	501	LSK	C2-C1-C11-O3
2	C	501	LSK	C2-C1-C11-O2
2	D	501	LSK	C6-C1-C11-O2
2	C	501	LSK	C1-C11-O2-C12
2	A	501	LSK	C2-C1-C11-O2
2	B	501	LSK	C6-C1-C11-O2
2	B	501	LSK	C6-C1-C11-O3
2	D	501	LSK	C2-C1-C11-O3
2	D	501	LSK	C6-C1-C11-O3
2	A	501	LSK	N2-C13-N1-C8
2	C	501	LSK	N2-C13-N1-C8
2	D	501	LSK	N2-C13-N1-C8
2	C	501	LSK	C6-C1-C11-O3
2	B	501	LSK	O3-C11-O2-C12
2	C	501	LSK	O3-C11-O2-C12
2	D	501	LSK	O3-C11-O2-C12
2	C	501	LSK	C2-C1-C11-O3
2	A	501	LSK	O4-C13-N1-C8
2	C	501	LSK	O4-C13-N1-C8
2	D	501	LSK	O4-C13-N1-C8
2	C	501	LSK	C28-C29-N3-C17
2	B	501	LSK	N2-C14-C15-C16
2	C	501	LSK	C7-C8-N1-C13
2	B	501	LSK	C9-C8-N1-C13
2	B	501	LSK	C7-C8-N1-C13
2	C	501	LSK	C9-C8-N1-C13
2	A	501	LSK	C7-C8-N1-C13
3	B	503	EDO	O1-C1-C2-O2
2	A	501	LSK	C9-C8-N1-C13
2	A	501	LSK	C15-C16-C28-C29
2	C	501	LSK	C14-C15-C16-C28
2	B	501	LSK	C14-C15-C16-C28
2	D	501	LSK	N4-C18-C19-C20
2	C	501	LSK	N2-C14-C15-C16
3	D	502	EDO	O1-C1-C2-O2

There are no ring outliers.

CONFIDENTIAL VALIDATION REPORT

2 monomers are involved in 2 short contacts:

Mol	Chain	Res	Type	Clashes	Symm-Clashes
3	A	502	EDO	1	0
2	A	501	LSK	1	0

The following is a two-dimensional graphical depiction of Mogul quality analysis of bond lengths, bond angles, torsion angles, and ring geometry for all instances of the Ligand of Interest. In addition, ligands with molecular weight > 250 and outliers as shown on the validation Tables will also be included. For torsion angles, if less then 5% of the Mogul distribution of torsion angles is within 10 degrees of the torsion angle in question, then that torsion angle is considered an outlier. Any bond that is central to one or more torsion angles identified as an outlier by Mogul will be highlighted in the graph. For rings, the root-mean-square deviation (RMSD) between the ring in question and similar rings identified by Mogul is calculated over all ring torsion angles. If the average RMSD is greater than 60 degrees and the minimal RMSD between the ring in question and any Mogul-identified rings is also greater than 60 degrees, then that ring is considered an outlier. The outliers are highlighted in purple. The color gray indicates Mogul did not find sufficient equivalents in the CSD to analyse the geometry.

Ligand LSK C 501	
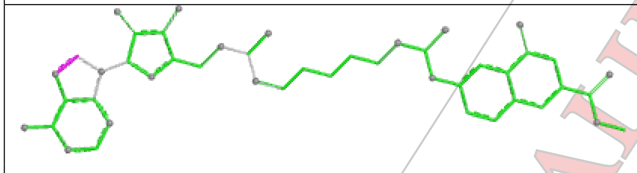 Bond lengths	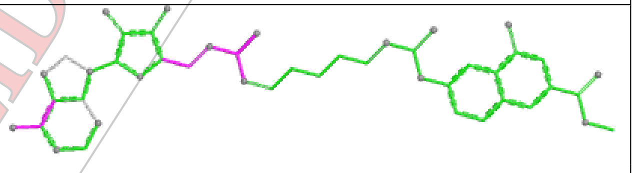 Bond angles
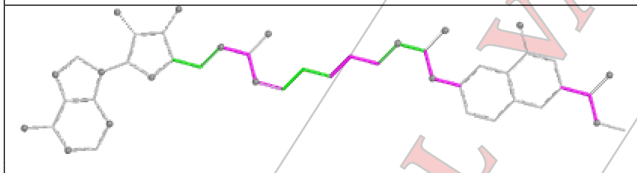 Torsions	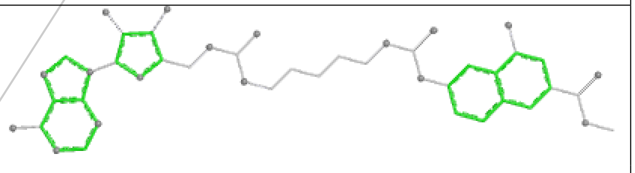 Rings

Ligand LSK B 501	
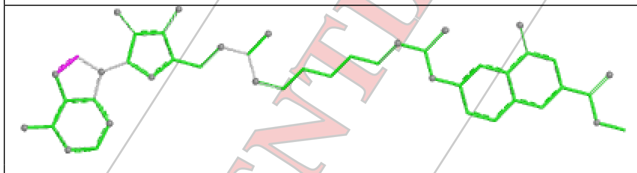 Bond lengths	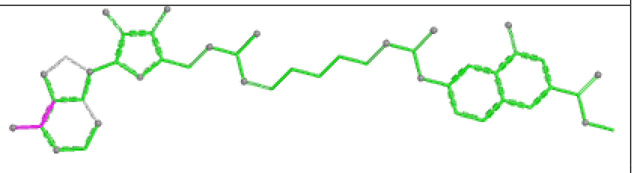 Bond angles
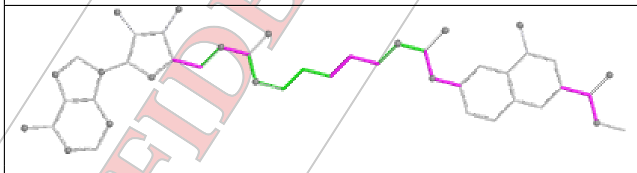 Torsions	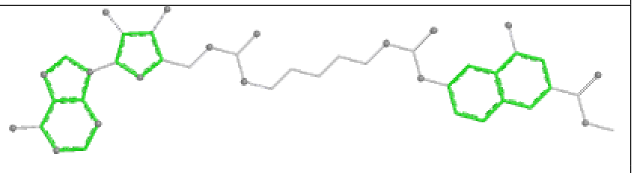 Rings

CONFIDENTIAL VALIDATION REPORT

Ligand LSK D 501	
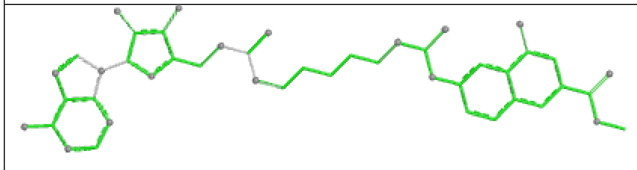 Bond lengths	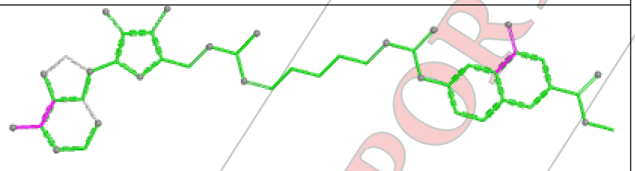 Bond angles
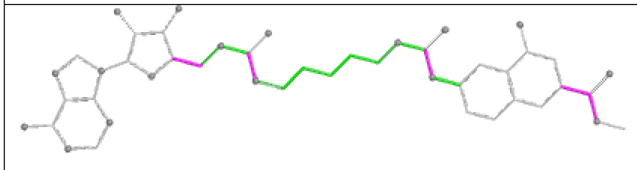 Torsions	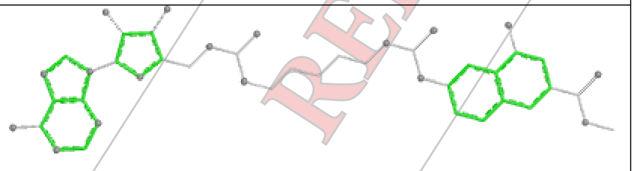 Rings

Ligand LSK A 501	
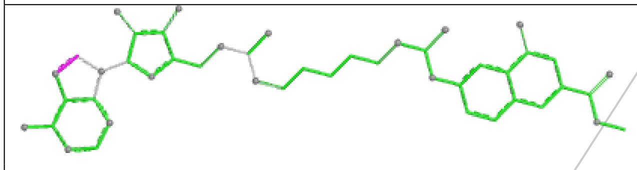 Bond lengths	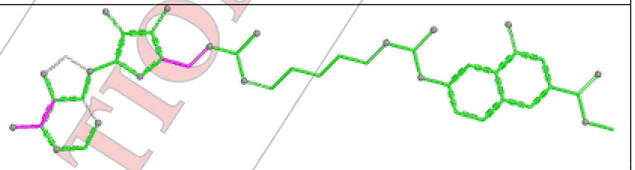 Bond angles
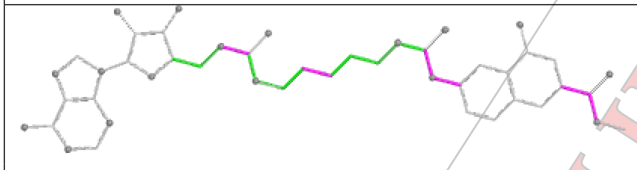 Torsions	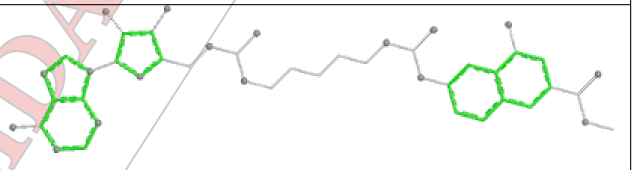 Rings

5.7 Other polymers

There are no such residues in this entry.

5.8 Polymer linkage issues

There are no chain breaks in this entry.

CONFIDENTIAL VALIDATION REPORT

6 Fit of model and data (i)

6.1 Protein, DNA and RNA chains (i)

In the following table, the column labelled '#RSRZ> 2' contains the number (and percentage) of RSRZ outliers, followed by percent RSRZ outliers for the chain as percentile scores relative to all X-ray entries and entries of similar resolution. The OWAB column contains the minimum, median, 95th percentile and maximum values of the occupancy-weighted average B-factor per residue. The column labelled 'Q< 0.9' lists the number of (and percentage) of residues with an average occupancy less than 0.9.

Mol	Chain	Analysed	<RSRZ>	#RSRZ>2			OWAB(Å^2)	Q<0.9
1	A	343/361 (95%)	0.26	6 (1%)	70	74	26, 40, 60, 95	0
1	B	343/361 (95%)	0.29	9 (2%)	56	61	31, 45, 66, 85	0
1	C	343/361 (95%)	0.20	6 (1%)	70	74	27, 42, 63, 91	0
1	D	342/361 (94%)	0.22	8 (2%)	60	65	29, 43, 64, 86	0
All	All	1371/1444 (94%)	0.24	29 (2%)	63	68	26, 42, 65, 95	0

All (29) RSRZ outliers are listed below:

Mol	Chain	Res	Type	RSRZ
1	B	135	ARG	5.9
1	B	144	GLU	4.2
1	A	140	GLU	4.0
1	D	347	ARG	3.6
1	B	136	SER	3.1
1	C	477	TYR	3.1
1	A	138	PHE	3.0
1	D	312	ASN	2.9
1	A	477	TYR	2.8
1	A	137	VAL	2.7
1	B	149	GLN	2.7
1	B	475	PHE	2.6
1	C	184	PHE	2.6
1	C	478	THR	2.6
1	D	315	TYR	2.6
1	D	380	LEU	2.4
1	A	283	SER	2.4
1	C	475	PHE	2.3
1	B	349	LEU	2.3
1	B	477	TYR	2.3
1	C	138	PHE	2.3

Continued on next page...

CONFIDENTIAL VALIDATION REPORT

Continued from previous page...

Mol	Chain	Res	Type	RSRZ
1	C	282	PRO	2.3
1	B	347	ARG	2.3
1	D	136	SER	2.2
1	D	328	ARG	2.2
1	B	140	GLU	2.2
1	D	137	VAL	2.1
1	A	478	THR	2.1
1	D	477	TYR	2.0

6.2 Non-standard residues in protein, DNA, RNA chains ⓘ

There are no non-standard protein/DNA/RNA residues in this entry.

6.3 Carbohydrates ⓘ

There are no monosaccharides in this entry.

6.4 Ligands ⓘ

In the following table, the Atoms column lists the number of modelled atoms in the group and the number defined in the chemical component dictionary. The B-factors column lists the minimum, median, 95th percentile and maximum values of B factors of atoms in the group. The column labelled 'Q< 0.9' lists the number of atoms with occupancy less than 0.9.

Mol	Type	Chain	Res	Atoms	RSCC	RSR	B-factors($Å^2$)	Q<0.9
3	EDO	C	503	4/4	0.74	0.24	67,81,81,90	0
3	EDO	C	502	4/4	0.78	0.29	57,68,77,77	0
3	EDO	A	503	4/4	0.83	0.30	58,70,81,81	0
3	EDO	B	503	4/4	0.83	0.16	56,68,73,78	0
3	EDO	D	502	4/4	0.86	0.11	51,62,64,75	0
3	EDO	B	502	4/4	0.87	0.15	41,50,60,72	0
2	LSK	B	501	46/46	0.89	0.21	33,61,88,100	0
2	LSK	C	501	46/46	0.89	0.23	36,61,99,102	0
2	LSK	A	501	46/46	0.90	0.18	32,56,93,102	0
2	LSK	D	501	46/46	0.91	0.20	28,65,92,102	0
3	EDO	A	502	4/4	0.93	0.14	46,60,73,83	0

The following is a graphical depiction of the model fit to experimental electron density of all instances of the Ligand of Interest. In addition, ligands with molecular weight > 250 and outliers as shown on the geometry validation Tables will also be included. Each fit is shown from different orientation to approximate a three-dimensional view.

CONFIDENTIAL VALIDATION REPORT

Electron density around LSK B 501:

$2mF_o$-DF_c (at 0.7 rmsd) in gray
mF_o-DF_c (at 3 rmsd) in purple (negative)
and green (positive)

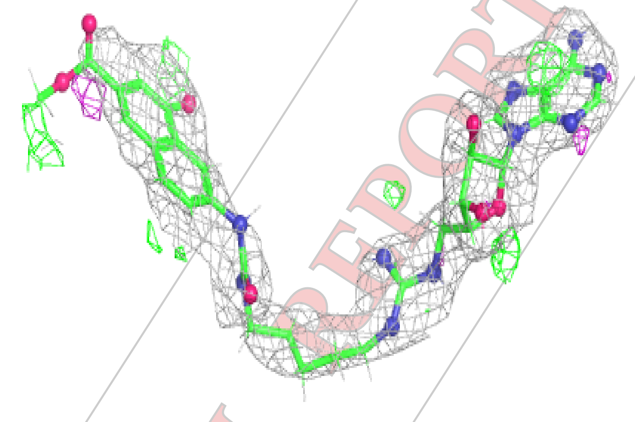

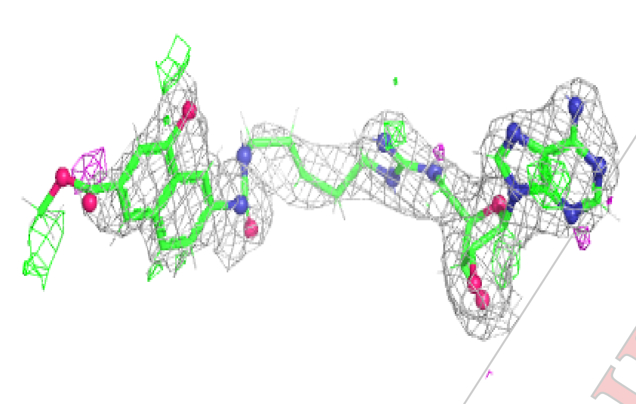

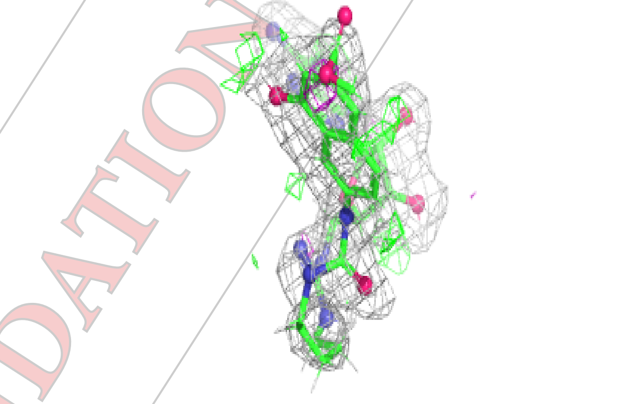

Electron density around LSK C 501:

$2mF_o$-DF_c (at 0.7 rmsd) in gray
mF_o-DF_c (at 3 rmsd) in purple (negative)
and green (positive)

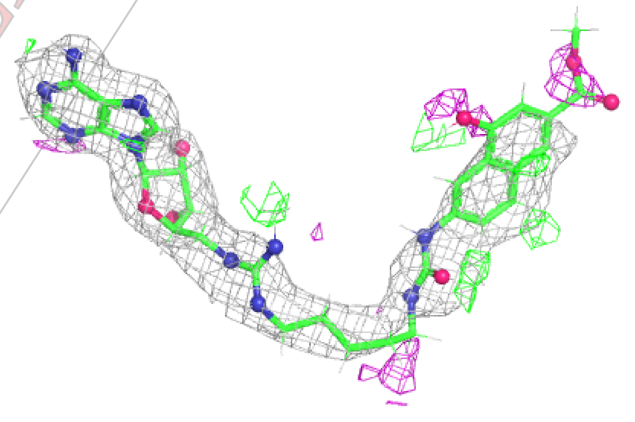

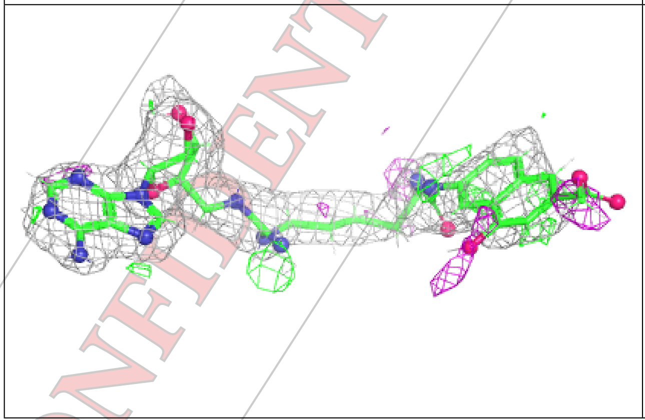

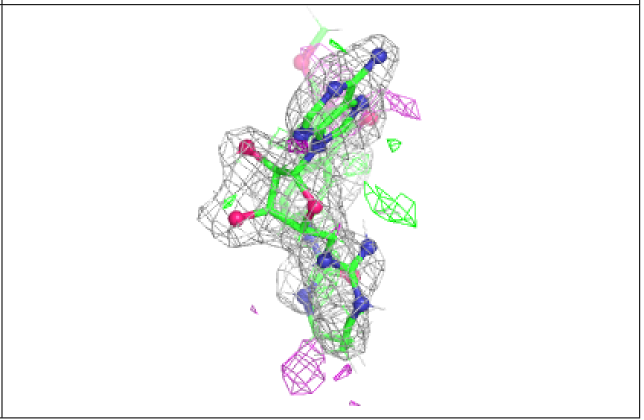

CONFIDENTIAL VALIDATION REPORT

Electron density around LSK A 501:

$2mF_o$-DF_c (at 0.7 rmsd) in gray
mF_o-DF_c (at 3 rmsd) in purple (negative)
and green (positive)

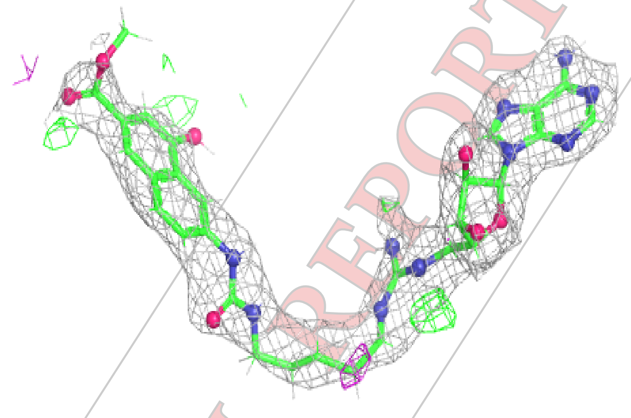

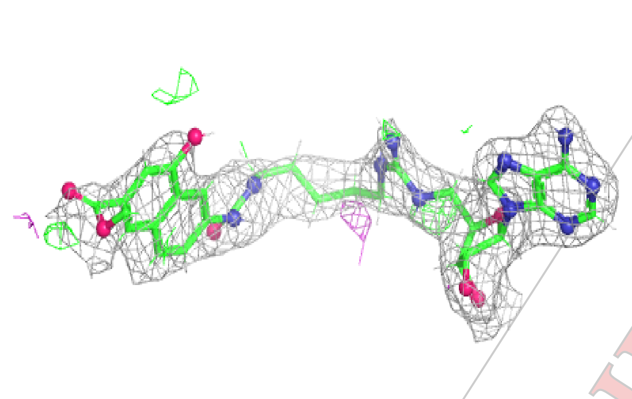

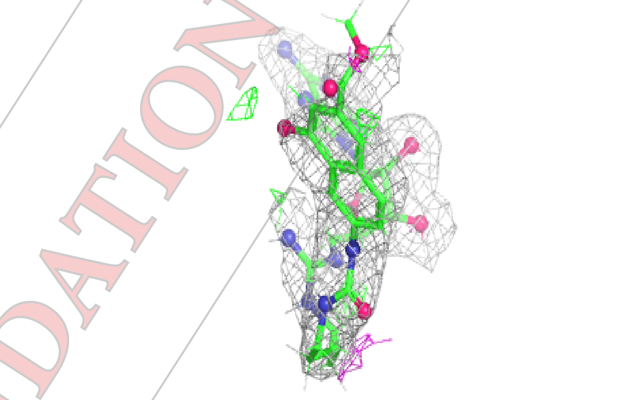

Electron density around LSK D 501:

$2mF_o$-DF_c (at 0.7 rmsd) in gray
mF_o-DF_c (at 3 rmsd) in purple (negative)
and green (positive)

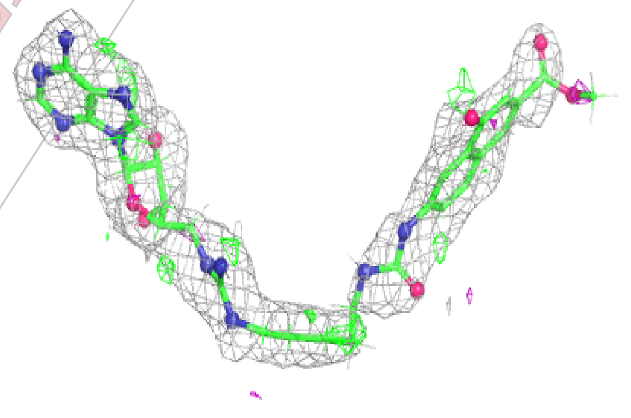

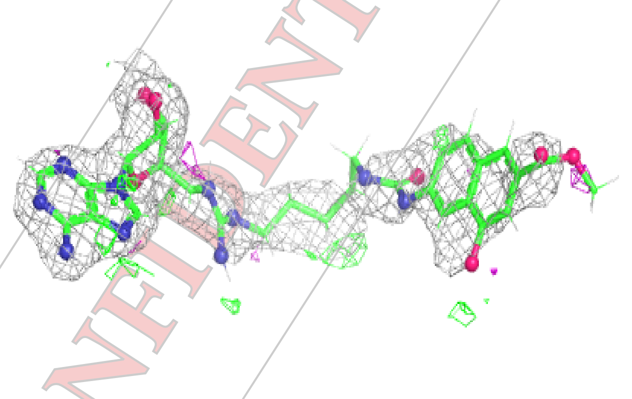

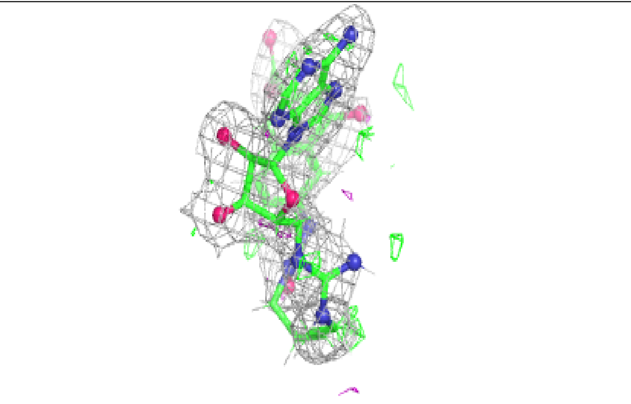

CONFIDENTIAL VALIDATION REPORT

6.5 Other polymers (i)

There are no such residues in this entry.

CONFIDENTIAL VALIDATION REPORT
