# Supplementary material for: Turning Nonselective Inhibitors of Type I Protein Arginine Methyltransferases into Potent and Selective Inhibitors of Protein Arginine Methyltransferase 4 through a Deconstruction–Reconstruction and Fragment-Growing Approach
Source: J Med Chem. 2022 Apr 28;65(17):11574–606. doi: 10.1021/acs.jmedchem.2c00252 (PMC9469100; doi:10.1021/acs.jmedchem.2c00252)
Supplement: Supplementary file 5 — jm2c00252_si_005.pdf [file jm2c00252_si_005.pdf]

Full wwPDB X-ray Structure Validation Report (i)

Oct 4, 2021 – 10:29 am BST

PDB ID	:	7PU8
Title	:	CARM1 in complex with EML980
Deposited on	:	2021-09-28
Resolution	:	2.19 Å(reported)

This is a Full wwPDB X-ray Structure Validation Report.

This report is produced by the wwPDB biocuration pipeline after annotation of the structure.

We welcome your comments at *validation@mail.wwpdb.org*
A user guide is available at
https://www.wwpdb.org/validation/2017/XrayValidationReportHelp
with specific help available everywhere you see the (i) symbol.

CONFIDENTIAL VALIDATION REPORT

The following versions of software and data (see references (i)) were used in the production of this report:

MolProbity	:	4.02b-467
Mogul	:	1.8.5 (274361), CSD as541be (2020)
Xtriage (Phenix)	:	1.13
EDS	:	2.23.2
buster-report	:	1.1.7 (2018)
Percentile statistics	:	20191225.v01 (using entries in the PDB archive December 25th 2019)
Refmac	:	5.8.0267
CCP4	:	7.1.010 (Gargrove)
Ideal geometry (proteins)	:	Engh & Huber (2001)
Ideal geometry (DNA, RNA)	:	Parkinson et al. (1996)
Validation Pipeline (wwPDB-VP)	:	2.23.2

1 Overall quality at a glance (i)

The following experimental techniques were used to determine the structure:
X-RAY DIFFRACTION

The reported resolution of this entry is 2.19 Å.

Percentile scores (ranging between 0-100) for global validation metrics of the entry are shown in the following graphic. The table shows the number of entries on which the scores are based.

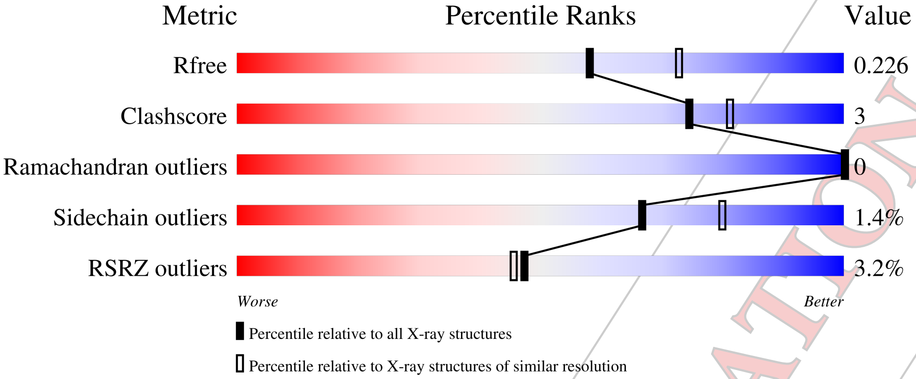

Metric	Whole archive (#Entries)	Similar resolution (#Entries, resolution range(Å))
R_{free}	130704	4898 (2.20-2.20)
Clashscore	141614	5594 (2.20-2.20)
Ramachandran outliers	138981	5503 (2.20-2.20)
Sidechain outliers	138945	5504 (2.20-2.20)
RSRZ outliers	127900	4800 (2.20-2.20)

The table below summarises the geometric issues observed across the polymeric chains and their fit to the electron density. The red, orange, yellow and green segments of the lower bar indicate the fraction of residues that contain outliers for >=3, 2, 1 and 0 types of geometric quality criteria respectively. A grey segment represents the fraction of residues that are not modelled. The numeric value for each fraction is indicated below the corresponding segment, with a dot representing fractions <=5% The upper red bar (where present) indicates the fraction of residues that have poor fit to the electron density. The numeric value is given above the bar.

Mol	Chain	Length	Quality of chain
1	A	361	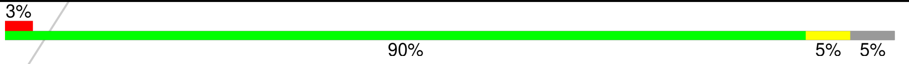 3% 90% 5% 5%
1	B	361	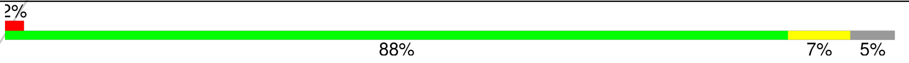 2% 88% 7% 5%
1	C	361	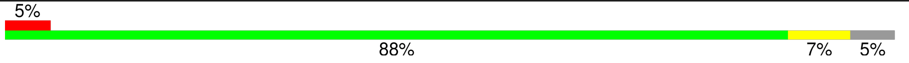 5% 88% 7% 5%
1	D	361	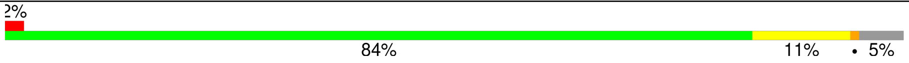 2% 84% 11% • 5%

The following table lists non-polymeric compounds, carbohydrate monomers and non-standard

CONFIDENTIAL VALIDATION REPORT

residues in protein, DNA, RNA chains that are outliers for geometric or electron-density-fit criteria:

Mol	Type	Chain	Res	Chirality	Geometry	Clashes	Electron density
3	EDO	A	503	-	-	-	X
3	EDO	D	502	-	-	-	X

CONFIDENTIAL VALIDATION REPORT

2 Entry composition (i)

There are 4 unique types of molecules in this entry. The entry contains 22448 atoms, of which 10965 are hydrogens and 0 are deuteriums.

In the tables below, the ZeroOcc column contains the number of atoms modelled with zero occupancy, the AltConf column contains the number of residues with at least one atom in alternate conformation and the Trace column contains the number of residues modelled with at most 2 atoms.

- Molecule 1 is a protein called Histone-arginine methyltransferase CARM1.

Mol	Chain	Residues	Atoms						ZeroOcc	AltConf	Trace
1	A	343	Total 5450	C 1776	H 2699	N 454	O 507	S 14	0	0	0
1	B	343	Total 5460	C 1778	H 2705	N 457	O 506	S 14	0	0	0
1	C	343	Total 5448	C 1776	H 2697	N 454	O 507	S 14	0	0	0
1	D	342	Total 5439	C 1769	H 2696	N 456	O 504	S 14	0	0	0

There are 12 discrepancies between the modelled and reference sequences:

Chain	Residue	Modelled	Actual	Comment	Reference
A	127	GLY	-	expression tag	UNP Q9WVG6
A	128	HIS	-	expression tag	UNP Q9WVG6
A	129	MET	-	expression tag	UNP Q9WVG6
B	127	GLY	-	expression tag	UNP Q9WVG6
B	128	HIS	-	expression tag	UNP Q9WVG6
B	129	MET	-	expression tag	UNP Q9WVG6
C	127	GLY	-	expression tag	UNP Q9WVG6
C	128	HIS	-	expression tag	UNP Q9WVG6
C	129	MET	-	expression tag	UNP Q9WVG6
D	127	GLY	-	expression tag	UNP Q9WVG6
D	128	HIS	-	expression tag	UNP Q9WVG6
D	129	MET	-	expression tag	UNP Q9WVG6

- Molecule 2 is methyl 6-[4-[[N-[2-[(2R,3S,4R,5R)-5-(6-aminopurin-9-yl)-3,4-bis(oxidanyl)oxolan-2-yl]ethyl]carbamimidoyl]amino]butylcarbamoylamino]-4-oxidanyl-naphthalene-2-carboxylate (three-letter code: 4R7) (formula: $C_{29}H_{36}N_{10}O_7$) (labeled as "Ligand of Interest" by depositor).

CONFIDENTIAL VALIDATION REPORT

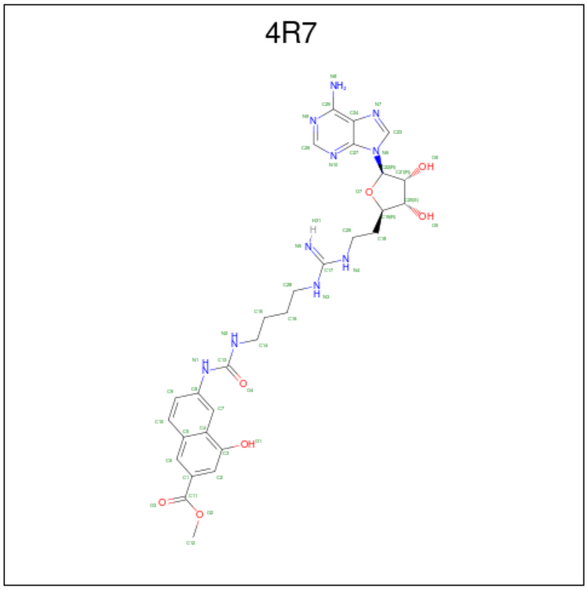

Mol	Chain	Residues	Atoms					ZeroOcc	AltConf
2	A	1	Total 82	C 29	H 36	N 10	O 7	0	0
2	B	1	Total 82	C 29	H 36	N 10	O 7	0	0
2	C	1	Total 82	C 29	H 36	N 10	O 7	0	0
2	D	1	Total 82	C 29	H 36	N 10	O 7	0	0

- Molecule 3 is 1,2-ETHANEDIOL (three-letter code: EDO) (formula: $C_2H_6O_2$).

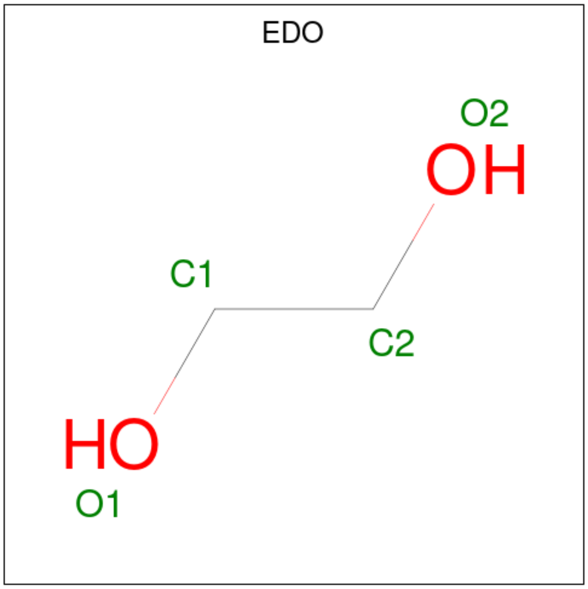

CONTINUATION VALIDATION REPORT

Mol	Chain	Residues	Atoms	ZeroOcc	AltConf
3	A	1	Total C H O<br>10 2 6 2	0	0
3	A	1	Total C H O<br>10 2 6 2	0	0
3	C	1	Total C H O<br>10 2 6 2	0	0
3	D	1	Total C H O<br>10 2 6 2	0	0

- Molecule 4 is water.

Mol	Chain	Residues	Atoms	ZeroOcc	AltConf
4	A	93	Total O<br>93 93	0	0
4	B	72	Total O<br>72 72	0	0
4	C	73	Total O<br>73 73	0	0
4	D	45	Total O<br>45 45	0	0

CONFIDENTIAL VALIDATION REPORT

3 Residue-property plots (i)

These plots are drawn for all protein, RNA, DNA and oligosaccharide chains in the entry. The first graphic for a chain summarises the proportions of the various outlier classes displayed in the second graphic. The second graphic shows the sequence view annotated by issues in geometry and electron density. Residues are color-coded according to the number of geometric quality criteria for which they contain at least one outlier: green = 0, yellow = 1, orange = 2 and red = 3 or more. A red dot above a residue indicates a poor fit to the electron density (RSRZ > 2). Stretches of 2 or more consecutive residues without any outlier are shown as a green connector. Residues present in the sample, but not in the model, are shown in grey.

- Molecule 1: Histone-arginine methyltransferase CARM1

Chain A:
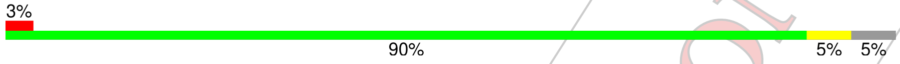

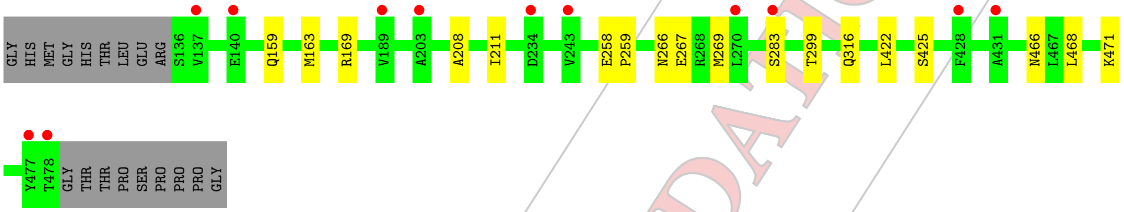

- Molecule 1: Histone-arginine methyltransferase CARM1

Chain B:
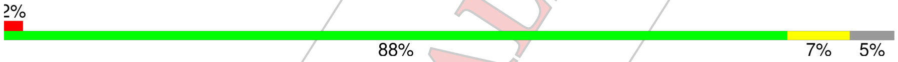

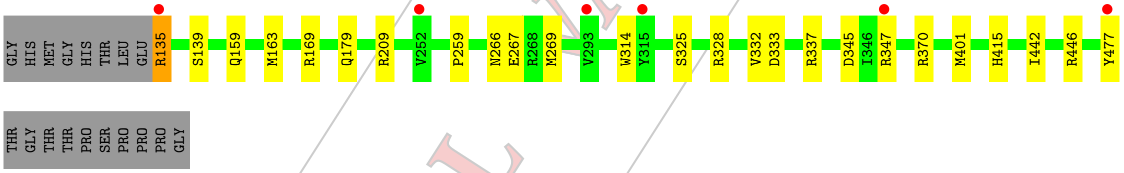

- Molecule 1: Histone-arginine methyltransferase CARM1

Chain C:
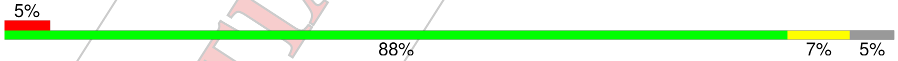

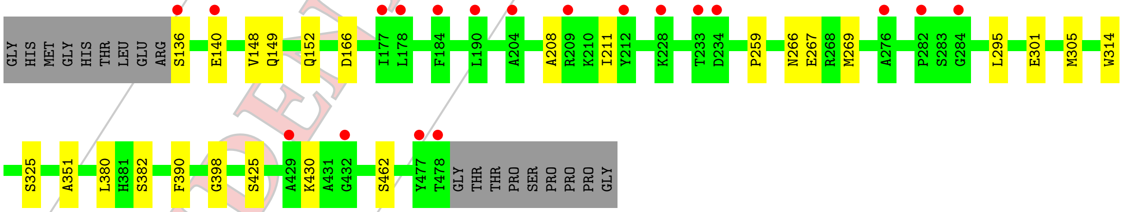

- Molecule 1: Histone-arginine methyltransferase CARM1

Chain D:
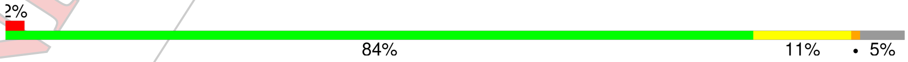

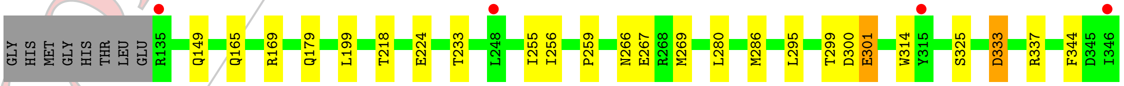

CONFIDENTIAL VALIDATION REPORT

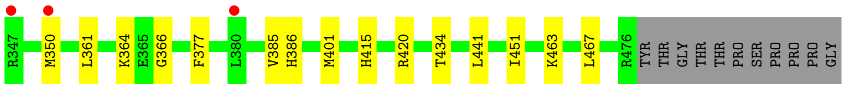

CONFIDENTIAL VALIDATION REPORT

4 Data and refinement statistics ⓘ

Property	Value	Source
Space group	P 21 21 2	Depositor
Cell constants<br>a, b, c, α, β, γ	75.21Å 98.81Å 208.40Å<br>90.00° 90.00° 90.00°	Depositor
Resolution (Å)	48.07 – 2.19<br>48.07 – 2.19	Depositor<br>EDS
% Data completeness<br>(in resolution range)	98.6 (48.07-2.19)<br>98.6 (48.07-2.19)	Depositor<br>EDS
R_{merge}	0.10	Depositor
R_{sym}	(Not available)	Depositor
$< I/\sigma(I) >$ [1]	1.60 (at 2.18Å)	Xtriage
Refinement program	PHENIX 1.19.2_4158	Depositor
R, R_{free}	0.194 , 0.229<br>0.192 , 0.226	Depositor<br>DCC
R_{free} test set	3984 reflections (4.97%)	wwPDB-VP
Wilson B-factor (Å^2)	45.8	Xtriage
Anisotropy	0.295	Xtriage
Bulk solvent k_{sol}(e/Å^3), B_{sol}(Å^2)	(Not available) , (Not available)	EDS
L-test for twinning[2]	$< \|L\| > = 0.49$, $< L^2 > = 0.32$	Xtriage
Estimated twinning fraction	No twinning to report.	Xtriage
F_o,F_c correlation	0.96	EDS
Total number of atoms	22448	wwPDB-VP
Average B, all atoms (Å^2)	58.0	wwPDB-VP

Xtriage's analysis on translational NCS is as follows: *The analyses of the Patterson function reveals a significant off-origin peak that is 46.67 % of the origin peak, indicating pseudo-translational symmetry. The chance of finding a peak of this or larger height randomly in a structure without pseudo-translational symmetry is equal to 1.1072e-04. The detected translational NCS is most likely also responsible for the elevated intensity ratio.*

[1] Intensities estimated from amplitudes.

[2] Theoretical values of $< |L| >$, $< L^2 >$ for acentric reflections are 0.5, 0.333 respectively for untwinned datasets, and 0.375, 0.2 for perfectly twinned datasets.

CONFIDENTIAL VALIDATION REPORT

5 Model quality (i)

5.1 Standard geometry (i)

Bond lengths and bond angles in the following residue types are not validated in this section: 4R7, EDO

The Z score for a bond length (or angle) is the number of standard deviations the observed value is removed from the expected value. A bond length (or angle) with $|Z| > 5$ is considered an outlier worth inspection. RMSZ is the root-mean-square of all Z scores of the bond lengths (or angles).

Mol	Chain	Bond lengths		Bond angles	
		RMSZ	$\#\|Z\| >5$	RMSZ	$\#\|Z\| >5$
1	A	0.37	0/2821	0.57	0/3823
1	B	0.34	0/2825	0.56	0/3827
1	C	0.34	0/2821	0.56	0/3823
1	D	0.34	0/2812	0.55	0/3809
All	All	0.35	0/11279	0.56	0/15282

There are no bond length outliers.

There are no bond angle outliers.

There are no chirality outliers.

There are no planarity outliers.

5.2 Too-close contacts (i)

In the following table, the Non-H and H(model) columns list the number of non-hydrogen atoms and hydrogen atoms in the chain respectively. The H(added) column lists the number of hydrogen atoms added and optimized by MolProbity. The Clashes column lists the number of clashes within the asymmetric unit, whereas Symm-Clashes lists symmetry-related clashes.

Mol	Chain	Non-H	H(model)	H(added)	Clashes	Symm-Clashes
1	A	2751	2699	2699	15	0
1	B	2755	2705	2705	13	0
1	C	2751	2697	2699	17	0
1	D	2743	2696	2696	29	0
2	A	46	36	0	0	0
2	B	46	36	0	0	0
2	C	46	36	0	0	0
2	D	46	36	0	0	0
3	A	8	12	12	0	0

Continued on next page...

CONFIDENTIAL VALIDATION REPORT

Continued from previous page...

Mol	Chain	Non-H	H(model)	H(added)	Clashes	Symm-Clashes
3	C	4	6	6	0	0
3	D	4	6	6	0	0
4	A	93	0	0	5	0
4	B	72	0	0	0	0
4	C	73	0	0	2	0
4	D	45	0	0	6	0
All	All	11483	10965	10823	70	0

The all-atom clashscore is defined as the number of clashes found per 1000 atoms (including hydrogen atoms). The all-atom clashscore for this structure is 3.

All (70) close contacts within the same asymmetric unit are listed below, sorted by their clash magnitude.

Atom-1	Atom-2	Interatomic distance (Å)	Clash overlap (Å)
1:A:316:GLN:CB	4:A:601:HOH:O	2.24	0.85
1:A:316:GLN:HB3	4:A:601:HOH:O	1.80	0.82
1:D:165:GLN:OE1	4:D:601:HOH:O	2.00	0.79
1:D:301:GLU:OE1	1:D:386:HIS:NE2	2.19	0.74
1:C:149:GLN:NE2	1:D:149:GLN:OE1	2.22	0.72
1:A:316:GLN:OE1	4:A:601:HOH:O	2.11	0.68
1:C:398:GLY:O	4:C:602:HOH:O	2.13	0.66
1:A:169:ARG:NE	1:A:258:GLU:OE1	2.29	0.64
1:A:316:GLN:HB2	4:A:601:HOH:O	1.95	0.63
1:D:179:GLN:NE2	1:D:401:MET:SD	2.71	0.62
1:B:135:ARG:HG3	1:B:139:SER:OG	2.02	0.59
1:C:149:GLN:CD	1:D:149:GLN:CD	2.62	0.58
1:B:209:ARG:HH11	1:B:209:ARG:HG3	1.70	0.56
1:A:266:ASN:O	1:A:267:GLU:HB2	2.05	0.56
1:D:441:LEU:HD22	1:D:451:ILE:HG12	1.87	0.56
1:C:136:SER:O	1:C:140:GLU:HG3	2.06	0.55
1:B:328:ARG:CZ	1:B:332:VAL:HG21	2.37	0.55
1:C:266:ASN:O	1:C:267:GLU:HB2	2.07	0.54
1:D:300:ASP:OD2	1:D:420:ARG:NH2	2.41	0.53
1:C:259:PRO:HA	1:C:269:MET:HG2	1.90	0.53
1:B:333:ASP:O	1:B:337:ARG:HG3	2.10	0.52
1:B:266:ASN:O	1:B:267:GLU:HB2	2.10	0.52
1:D:266:ASN:O	1:D:267:GLU:HB2	2.10	0.51
1:D:218:THR:HG23	4:D:620:HOH:O	2.10	0.51
1:D:233:THR:HG22	4:D:641:HOH:O	2.11	0.50
1:A:316:GLN:HB2	4:A:677:HOH:O	2.10	0.50
1:B:370:ARG:HG3	1:B:442:ILE:CD1	2.41	0.50

Continued on next page...

CONFIDENTIAL VALIDATION REPORT

Continued from previous page...

Atom-1	Atom-2	Interatomic distance (Å)	Clash overlap (Å)
1:D:286:MET:HG3	1:D:361:LEU:HD23	1.93	0.49
1:D:314:TRP:O	1:D:325:SER:HA	2.11	0.49
1:A:422:LEU:O	1:A:466:ASN:ND2	2.47	0.48
1:D:259:PRO:HA	1:D:269:MET:HG2	1.96	0.47
1:B:328:ARG:HD3	1:B:328:ARG:C	2.35	0.47
1:B:345:ASP:OD1	1:B:347:ARG:HG2	2.14	0.47
1:D:301:GLU:OE1	1:D:386:HIS:CE1	2.67	0.47
1:B:209:ARG:HG3	1:B:209:ARG:NH1	2.29	0.47
1:C:149:GLN:OE1	1:D:149:GLN:CD	2.53	0.47
1:D:233:THR:HB	4:D:641:HOH:O	2.15	0.47
1:C:166:ASP:OD1	4:C:603:HOH:O	2.20	0.47
1:B:314:TRP:O	1:B:325:SER:HA	2.15	0.46
1:C:301:GLU:O	1:C:305:MET:HG3	2.16	0.45
1:D:364:LYS:HD3	1:D:366:GLY:H	1.81	0.45
1:A:259:PRO:HA	1:A:269:MET:HG2	1.97	0.45
1:D:337:ARG:HG2	1:D:467:LEU:O	2.16	0.45
1:C:149:GLN:CD	1:D:149:GLN:OE1	2.55	0.45
1:D:350:MET:SD	1:D:385:VAL:HG22	2.56	0.45
1:B:179:GLN:NE2	1:B:401:MET:SD	2.88	0.44
1:D:224:GLU:HA	1:D:224:GLU:OE1	2.17	0.43
1:D:299:THR:HG23	1:D:299:THR:O	2.18	0.43
1:A:159:GLN:O	1:A:163:MET:HG3	2.19	0.43
1:C:295:LEU:HG	1:C:390:PHE:CE2	2.53	0.43
1:D:344:PHE:CD2	1:D:420:ARG:HD2	2.54	0.42
1:A:266:ASN:O	1:A:267:GLU:CB	2.67	0.42
1:B:159:GLN:O	1:B:163:MET:HG3	2.20	0.42
1:C:351:ALA:HA	1:C:380:LEU:HD12	2.02	0.42
1:D:255:ILE:HG13	1:D:280:LEU:HD13	2.00	0.42
1:A:466:ASN:HD22	1:A:468:LEU:HD11	1.84	0.42
1:A:466:ASN:ND2	1:A:468:LEU:HD11	2.34	0.42
1:C:148:VAL:O	1:C:152:GLN:HG3	2.20	0.42
1:A:208:ALA:HB3	1:A:211:ILE:HD11	2.02	0.41
1:D:377:PHE:O	1:D:434:THR:HA	2.19	0.41
1:C:266:ASN:O	1:C:267:GLU:CB	2.68	0.41
1:C:208:ALA:HB3	1:C:211:ILE:HD11	2.03	0.41
1:D:233:THR:CB	4:D:641:HOH:O	2.68	0.41
1:B:259:PRO:HA	1:B:269:MET:HG2	2.02	0.41
1:C:314:TRP:O	1:C:325:SER:HA	2.21	0.41
1:A:299:THR:O	1:A:299:THR:HG23	2.20	0.40
1:D:333:ASP:O	1:D:337:ARG:HG3	2.21	0.40
1:C:430:LYS:HB2	1:C:430:LYS:HE2	1.92	0.40

Continued on next page...

CONFIDENTIAL VALIDATION REPORT

Continued from previous page...

Atom-1	Atom-2	Interatomic distance (Å)	Clash overlap (Å)
1:D:233:THR:CG2	4:D:641:HOH:O	2.68	0.40
1:D:199:LEU:HB3	1:D:256:ILE:HD12	2.03	0.40

There are no symmetry-related clashes.

5.3 Torsion angles (i)

5.3.1 Protein backbone (i)

In the following table, the Percentiles column shows the percent Ramachandran outliers of the chain as a percentile score with respect to all X-ray entries followed by that with respect to entries of similar resolution.

The Analysed column shows the number of residues for which the backbone conformation was analysed, and the total number of residues.

Mol	Chain	Analysed	Favoured	Allowed	Outliers	Percentiles	
1	A	341/361 (94%)	331 (97%)	10 (3%)	0	100	100
1	B	341/361 (94%)	331 (97%)	10 (3%)	0	100	100
1	C	341/361 (94%)	331 (97%)	10 (3%)	0	100	100
1	D	340/361 (94%)	331 (97%)	9 (3%)	0	100	100
All	All	1363/1444 (94%)	1324 (97%)	39 (3%)	0	100	100

There are no Ramachandran outliers to report.

5.3.2 Protein sidechains (i)

In the following table, the Percentiles column shows the percent sidechain outliers of the chain as a percentile score with respect to all X-ray entries followed by that with respect to entries of similar resolution.

The Analysed column shows the number of residues for which the sidechain conformation was analysed, and the total number of residues.

Mol	Chain	Analysed	Rotameric	Outliers	Percentiles	
1	A	299/313 (96%)	296 (99%)	3 (1%)	76	86
1	B	299/313 (96%)	294 (98%)	5 (2%)	60	74
1	C	299/313 (96%)	296 (99%)	3 (1%)	76	86

Continued on next page...

CONFIDENTIAL VALIDATION REPORT

Continued from previous page...

Mol	Chain	Analysed	Rotameric	Outliers	Percentiles	
1	D	298/313 (95%)	292 (98%)	6 (2%)	55	69
All	All	1195/1252 (95%)	1178 (99%)	17 (1%)	67	80

All (17) residues with a non-rotameric sidechain are listed below:

Mol	Chain	Res	Type
1	A	283	SER
1	A	425	SER
1	A	471	LYS
1	B	135	ARG
1	B	169	ARG
1	B	415	HIS
1	B	446	ARG
1	B	477	TYR
1	C	382	SER
1	C	425	SER
1	C	462	SER
1	D	169	ARG
1	D	295	LEU
1	D	301	GLU
1	D	333	ASP
1	D	415	HIS
1	D	463	LYS

Sometimes sidechains can be flipped to improve hydrogen bonding and reduce clashes. All (1) such sidechains are listed below:

Mol	Chain	Res	Type
1	A	466	ASN

5.3.3 RNA (i)

There are no RNA molecules in this entry.

5.4 Non-standard residues in protein, DNA, RNA chains (i)

There are no non-standard protein/DNA/RNA residues in this entry.

CONFIDENTIAL VALIDATION REPORT

5.5 Carbohydrates (i)

There are no monosaccharides in this entry.

5.6 Ligand geometry (i)

8 ligands are modelled in this entry.

In the following table, the Counts columns list the number of bonds (or angles) for which Mogul statistics could be retrieved, the number of bonds (or angles) that are observed in the model and the number of bonds (or angles) that are defined in the Chemical Component Dictionary. The Link column lists molecule types, if any, to which the group is linked. The Z score for a bond length (or angle) is the number of standard deviations the observed value is removed from the expected value. A bond length (or angle) with $|Z| > 2$ is considered an outlier worth inspection. RMSZ is the root-mean-square of all Z scores of the bond lengths (or angles).

Mol	Type	Chain	Res	Link	Bond lengths			Bond angles		
					Counts	RMSZ	$\#\|Z\| > 2$	Counts	RMSZ	$\#\|Z\| > 2$
2	4R7	C	501	-	45,50,50	0.58	0	56,70,70	1.00	4 (7%)
3	EDO	C	502	-	3,3,3	0.85	0	2,2,2	0.40	0
3	EDO	D	502	-	3,3,3	0.84	0	2,2,2	0.39	0
3	EDO	A	503	-	3,3,3	0.06	0	2,2,2	0.16	0
2	4R7	A	501	-	45,50,50	0.60	1 (2%)	56,70,70	0.72	1 (1%)
3	EDO	A	502	-	3,3,3	0.79	0	2,2,2	0.54	0
2	4R7	D	501	-	45,50,50	0.62	1 (2%)	56,70,70	1.02	3 (5%)
2	4R7	B	501	-	45,50,50	0.62	1 (2%)	56,70,70	1.11	5 (8%)

In the following table, the Chirals column lists the number of chiral outliers, the number of chiral centers analysed, the number of these observed in the model and the number defined in the Chemical Component Dictionary. Similar counts are reported in the Torsion and Rings columns. '-' means no outliers of that kind were identified.

Mol	Type	Chain	Res	Link	Chirals	Torsions	Rings
2	4R7	C	501	-	-	12/25/45/45	0/5/5/5
3	EDO	C	502	-	-	0/1/1/1	-
3	EDO	D	502	-	-	0/1/1/1	-
3	EDO	A	503	-	-	0/1/1/1	-
2	4R7	A	501	-	-	14/25/45/45	0/5/5/5
3	EDO	A	502	-	-	0/1/1/1	-
2	4R7	D	501	-	-	12/25/45/45	0/5/5/5
2	4R7	B	501	-	-	10/25/45/45	0/5/5/5

All (3) bond length outliers are listed below:

CONFIDENTIAL VALIDATION REPORT

Mol	Chain	Res	Type	Atoms	Z	Observed(Å)	Ideal(Å)
2	A	501	4R7	C23-N7	-2.09	1.31	1.34
2	B	501	4R7	C23-N7	-2.02	1.31	1.34
2	D	501	4R7	C23-N7	-2.01	1.31	1.34

All (13) bond angle outliers are listed below:

Mol	Chain	Res	Type	Atoms	Z	Observed(^{o})	Ideal(^{o})
2	B	501	4R7	N3-C17-N5	3.97	127.69	120.26
2	B	501	4R7	N4-C17-N5	-3.93	112.89	120.26
2	D	501	4R7	N4-C17-N5	-3.34	114.01	120.26
2	D	501	4R7	N3-C17-N5	3.30	126.45	120.26
2	C	501	4R7	N4-C17-N5	-3.19	114.28	120.26
2	C	501	4R7	N3-C17-N5	3.16	126.18	120.26
2	B	501	4R7	C24-C25-N8	2.46	124.08	120.35
2	C	501	4R7	C20-C21-C22	-2.43	97.32	100.98
2	D	501	4R7	C24-C25-N8	2.42	124.03	120.35
2	C	501	4R7	C24-C25-N8	2.33	123.89	120.35
2	A	501	4R7	C24-C25-N8	2.33	123.89	120.35
2	B	501	4R7	O5-C20-C19	2.25	117.55	111.05
2	B	501	4R7	C20-C21-C22	-2.03	97.92	100.98

There are no chirality outliers.

All (48) torsion outliers are listed below:

Mol	Chain	Res	Type	Atoms
2	A	501	4R7	N4-C17-N3-C28
2	A	501	4R7	N5-C17-N3-C28
2	A	501	4R7	N3-C17-N4-C29
2	A	501	4R7	N5-C17-N4-C29
2	A	501	4R7	C19-C18-C29-N4
2	A	501	4R7	C29-C18-C19-C20
2	A	501	4R7	C29-C18-C19-O7
2	A	501	4R7	C1-C11-O2-C12
2	B	501	4R7	C1-C11-O2-C12
2	C	501	4R7	C2-C1-C11-O2
2	C	501	4R7	C19-C18-C29-N4
2	C	501	4R7	C18-C29-N4-C17
2	D	501	4R7	C2-C1-C11-O2
2	D	501	4R7	C1-C11-O2-C12
2	B	501	4R7	C6-C1-C11-O2
2	C	501	4R7	C6-C1-C11-O2
2	D	501	4R7	C6-C1-C11-O2

Continued on next page...

CONFIDENTIAL VALIDATION REPORT

Continued from previous page...

Mol	Chain	Res	Type	Atoms
2	C	501	4R7	C1-C11-O2-C12
2	B	501	4R7	C2-C1-C11-O2
2	C	501	4R7	C2-C1-C11-O3
2	D	501	4R7	C2-C1-C11-O3
2	D	501	4R7	C6-C1-C11-O3
2	C	501	4R7	C6-C1-C11-O3
2	A	501	4R7	O3-C11-O2-C12
2	B	501	4R7	O3-C11-O2-C12
2	C	501	4R7	O3-C11-O2-C12
2	D	501	4R7	O3-C11-O2-C12
2	B	501	4R7	C2-C1-C11-O3
2	B	501	4R7	C6-C1-C11-O3
2	B	501	4R7	C18-C29-N4-C17
2	D	501	4R7	C18-C29-N4-C17
2	A	501	4R7	N2-C14-C15-C16
2	B	501	4R7	N2-C14-C15-C16
2	D	501	4R7	C19-C18-C29-N4
2	B	501	4R7	C14-C15-C16-C28
2	A	501	4R7	C6-C1-C11-O2
2	A	501	4R7	C2-C1-C11-O2
2	C	501	4R7	N5-C17-N3-C28
2	C	501	4R7	N5-C17-N4-C29
2	D	501	4R7	N5-C17-N3-C28
2	D	501	4R7	N5-C17-N4-C29
2	A	501	4R7	C6-C1-C11-O3
2	C	501	4R7	N4-C17-N3-C28
2	C	501	4R7	N3-C17-N4-C29
2	D	501	4R7	N4-C17-N3-C28
2	D	501	4R7	N3-C17-N4-C29
2	A	501	4R7	C2-C1-C11-O3
2	B	501	4R7	C19-C18-C29-N4

There are no ring outliers.

No monomer is involved in short contacts.

The following is a two-dimensional graphical depiction of Mogul quality analysis of bond lengths, bond angles, torsion angles, and ring geometry for all instances of the Ligand of Interest. In addition, ligands with molecular weight > 250 and outliers as shown on the validation Tables will also be included. For torsion angles, if less then 5% of the Mogul distribution of torsion angles is within 10 degrees of the torsion angle in question, then that torsion angle is considered an outlier. Any bond that is central to one or more torsion angles identified as an outlier by Mogul will be highlighted in the graph. For rings, the root-mean-square deviation (RMSD) between the ring in question and similar rings identified by Mogul is calculated over all ring torsion angles. If the

CONFIDENTIAL VALIDATION REPORT

average RMSD is greater than 60 degrees and the minimal RMSD between the ring in question and any Mogul-identified rings is also greater than 60 degrees, then that ring is considered an outlier. The outliers are highlighted in purple. The color gray indicates Mogul did not find sufficient equivalents in the CSD to analyse the geometry.

Ligand 4R7 C 501

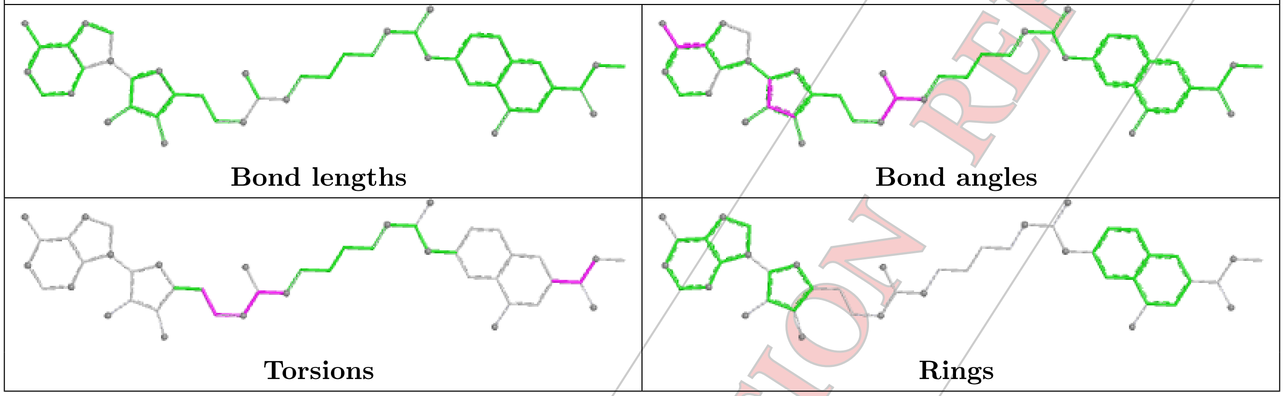

Ligand 4R7 A 501

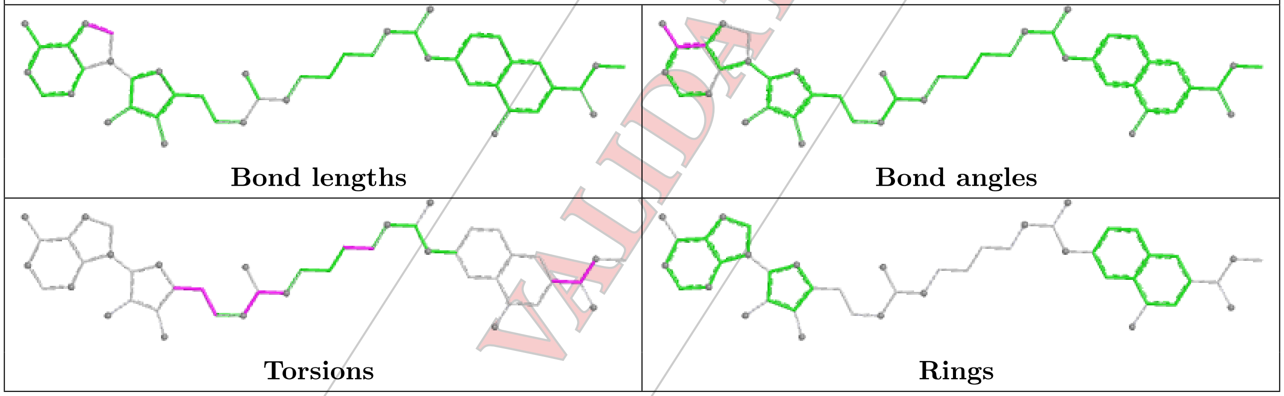

Ligand 4R7 D 501

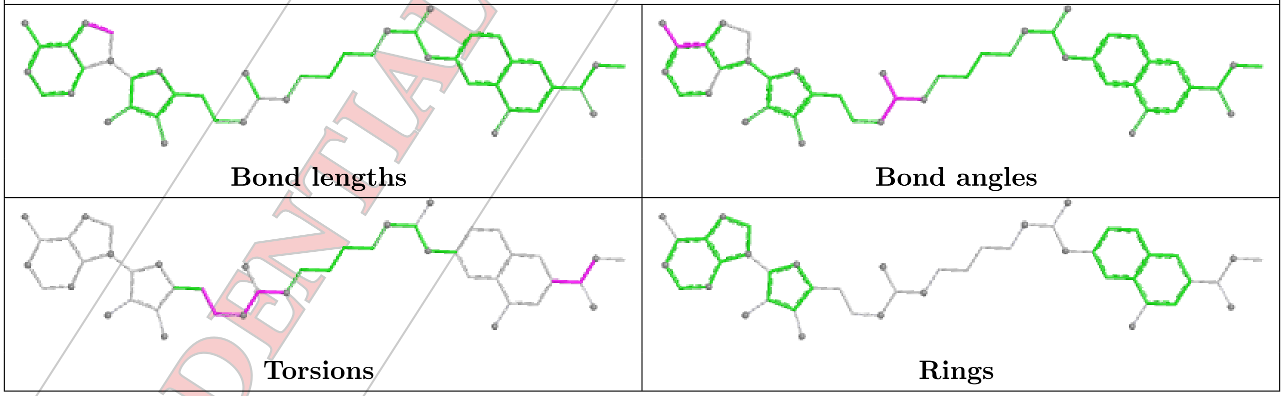

CONFIDENTIAL VALIDATION REPORT

Ligand 4R7 B 501	
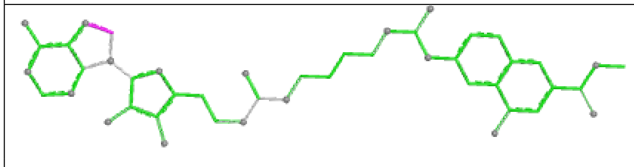 Bond lengths	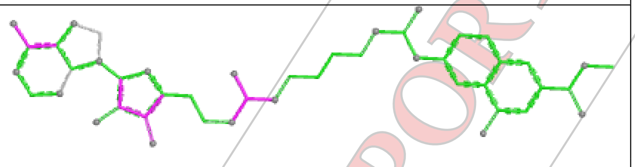 Bond angles
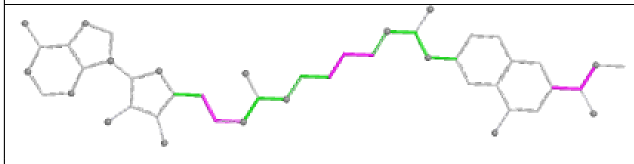 Torsions	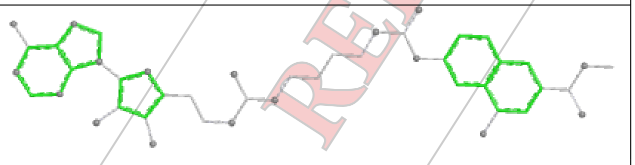 Rings

5.7 Other polymers (i)

There are no such residues in this entry.

5.8 Polymer linkage issues (i)

There are no chain breaks in this entry.

CONFIDENTIAL VALIDATION REPORT

6 Fit of model and data (i)

6.1 Protein, DNA and RNA chains (i)

In the following table, the column labelled '#RSRZ> 2' contains the number (and percentage) of RSRZ outliers, followed by percent RSRZ outliers for the chain as percentile scores relative to all X-ray entries and entries of similar resolution. The OWAB column contains the minimum, median, 95th percentile and maximum values of the occupancy-weighted average B-factor per residue. The column labelled 'Q< 0.9' lists the number of (and percentage) of residues with an average occupancy less than 0.9.

Mol	Chain	Analysed	<RSRZ>	#RSRZ>2			OWAB(Å^2)	Q<0.9
1	A	343/361 (95%)	0.47	12 (3%)	44	42	32, 47, 63, 91	0
1	B	343/361 (95%)	0.38	6 (1%)	70	68	36, 51, 66, 81	0
1	C	343/361 (95%)	0.61	19 (5%)	25	24	35, 54, 71, 91	0
1	D	342/361 (94%)	0.43	7 (2%)	65	63	41, 55, 70, 83	0
All	All	1371/1444 (94%)	0.47	44 (3%)	47	45	32, 52, 69, 91	0

All (44) RSRZ outliers are listed below:

Mol	Chain	Res	Type	RSRZ
1	C	204	ALA	6.5
1	C	478	THR	6.1
1	C	136	SER	5.4
1	A	234	ASP	4.6
1	C	477	TYR	4.5
1	C	276	ALA	4.5
1	D	135	ARG	4.3
1	B	135	ARG	4.3
1	B	315	TYR	4.2
1	C	234	ASP	4.1
1	D	347	ARG	3.9
1	C	209	ARG	3.7
1	C	228	LYS	3.7
1	A	137	VAL	3.7
1	B	347	ARG	3.4
1	C	233	THR	3.3
1	D	315	TYR	3.2
1	C	178	LEU	3.2
1	B	477	TYR	3.2
1	C	284	GLY	3.0
1	C	429	ALA	2.9

Continued on next page...

CONFIDENTIAL VALIDATION REPORT

Continued from previous page...

Mol	Chain	Res	Type	RSRZ
1	A	428	PHE	2.8
1	C	212	TYR	2.7
1	C	184	PHE	2.7
1	C	432	GLY	2.7
1	A	477	TYR	2.6
1	D	346	ILE	2.6
1	C	190	LEU	2.6
1	A	283	SER	2.5
1	B	293	VAL	2.4
1	A	140	GLU	2.4
1	C	282	PRO	2.4
1	A	270	LEU	2.4
1	A	243	VAL	2.4
1	A	203	ALA	2.4
1	D	350	MET	2.3
1	C	177	ILE	2.3
1	B	252	VAL	2.3
1	D	380	LEU	2.2
1	A	431	ALA	2.2
1	A	189	VAL	2.2
1	D	248	LEU	2.1
1	C	140	GLU	2.1
1	A	478	THR	2.1

6.2 Non-standard residues in protein, DNA, RNA chains (i)

There are no non-standard protein/DNA/RNA residues in this entry.

6.3 Carbohydrates (i)

There are no monosaccharides in this entry.

6.4 Ligands (i)

In the following table, the Atoms column lists the number of modelled atoms in the group and the number defined in the chemical component dictionary. The B-factors column lists the minimum, median, 95th percentile and maximum values of B factors of atoms in the group. The column labelled 'Q< 0.9' lists the number of atoms with occupancy less than 0.9.

Continued on next page...

CONFIDENTIAL VALIDATION REPORT

Continued from previous page...

Mol	Type	Chain	Res	Atoms	RSCC	RSR	B-factors($Å^2$)	Q<0.9
3	EDO	A	503	4/4	0.38	0.45	66,79,92,104	0
3	EDO	A	502	4/4	0.50	0.27	65,79,88,88	0
3	EDO	D	502	4/4	0.61	0.47	70,85,88,88	0
3	EDO	C	502	4/4	0.62	0.36	70,84,97,97	0
2	4R7	A	501	46/46	0.85	0.19	38,62,91,102	0
2	4R7	D	501	46/46	0.87	0.18	40,60,79,81	0
2	4R7	C	501	46/46	0.89	0.17	45,64,87,98	0
2	4R7	B	501	46/46	0.91	0.19	36,53,74,95	0

The following is a graphical depiction of the model fit to experimental electron density of all instances of the Ligand of Interest. In addition, ligands with molecular weight > 250 and outliers as shown on the geometry validation Tables will also be included. Each fit is shown from different orientation to approximate a three-dimensional view.

Electron density around 4R7 A 501:

$2mF_o$-DF_c (at 0.7 rmsd) in gray
mF_o-DF_c (at 3 rmsd) in purple (negative)
and green (positive)

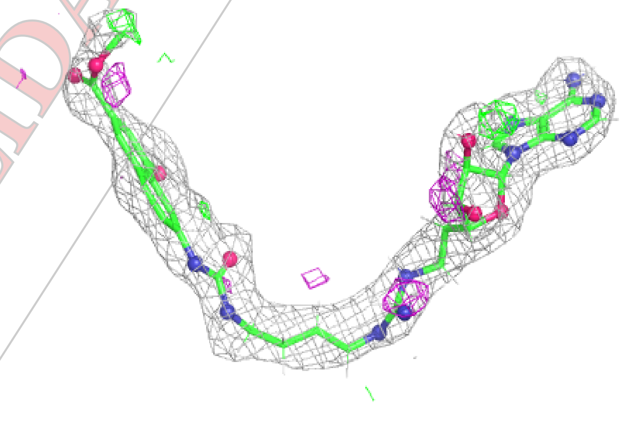

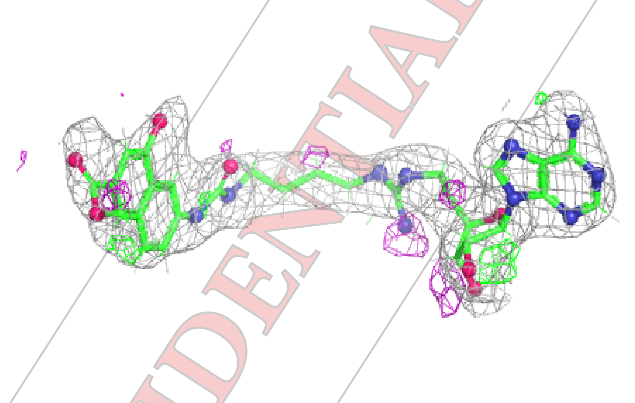

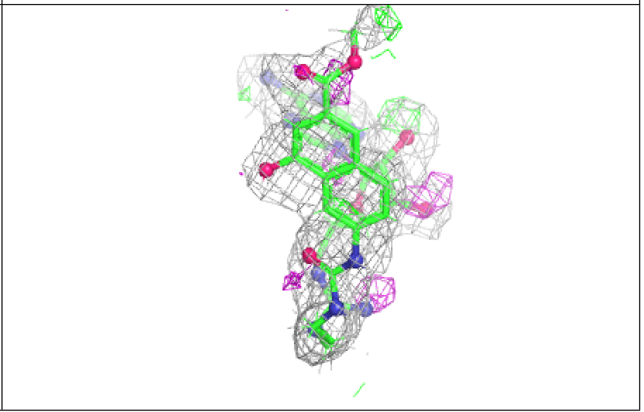

CONFIDENTIAL VALIDATION REPORT

Electron density around 4R7 D 501:

$2mF_o$-DF_c (at 0.7 rmsd) in gray
mF_o-DF_c (at 3 rmsd) in purple (negative) and green (positive)

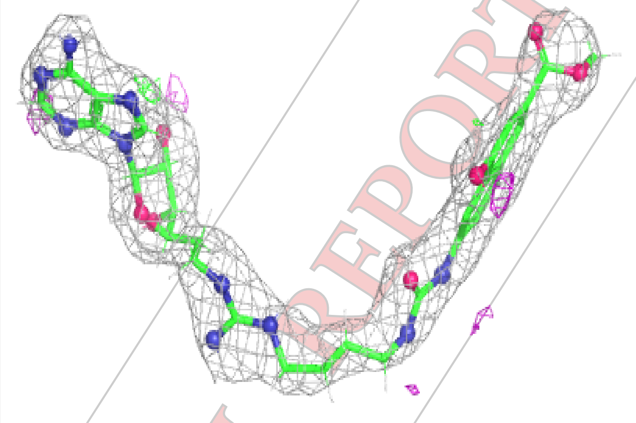

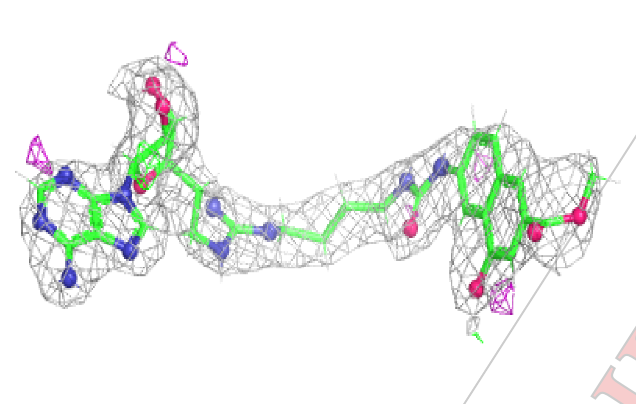

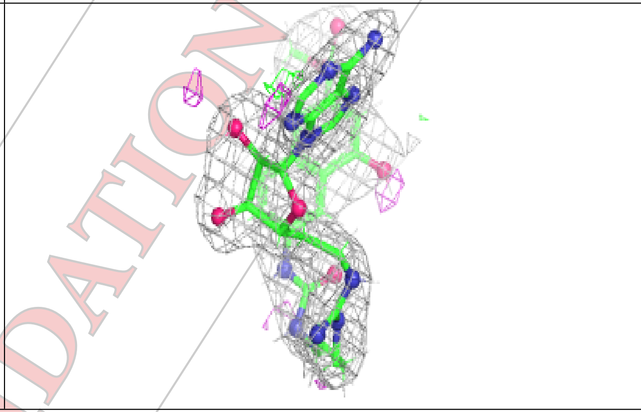

Electron density around 4R7 C 501:

$2mF_o$-DF_c (at 0.7 rmsd) in gray
mF_o-DF_c (at 3 rmsd) in purple (negative) and green (positive)

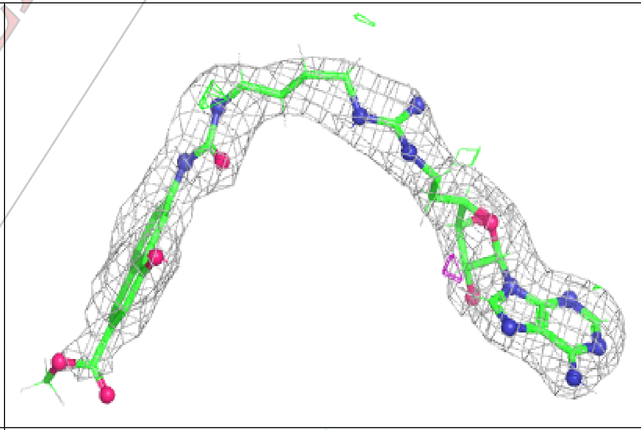

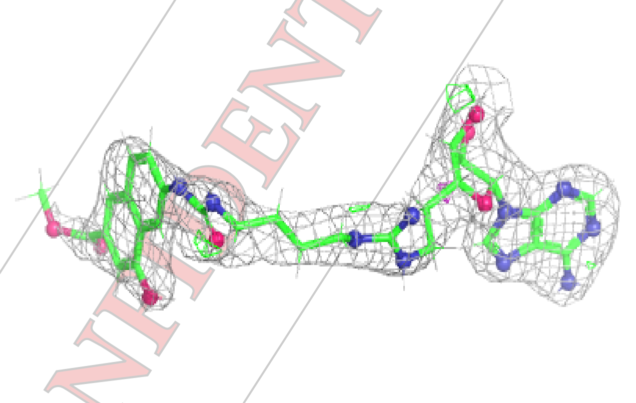

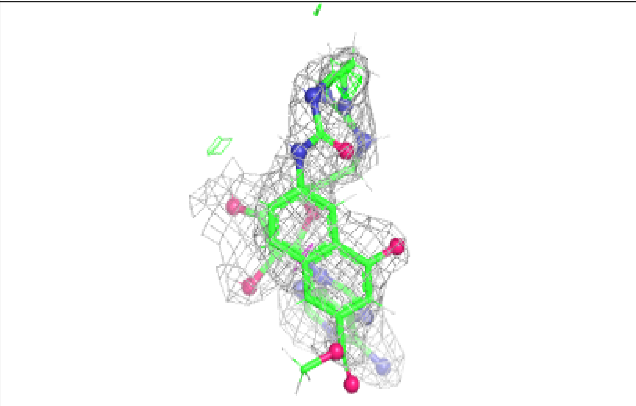

Electron density around 4R7 B 501:

$2mF_o$-DF_c (at 0.7 rmsd) in gray
mF_o-DF_c (at 3 rmsd) in purple (negative)
and green (positive)

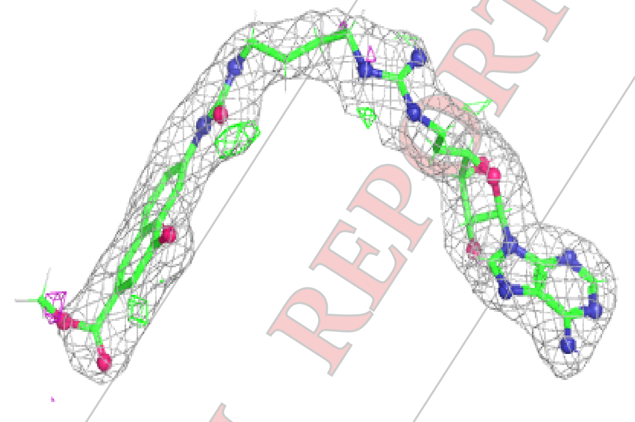

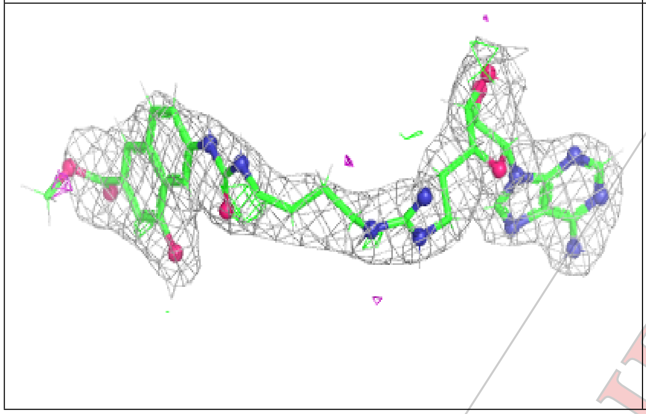

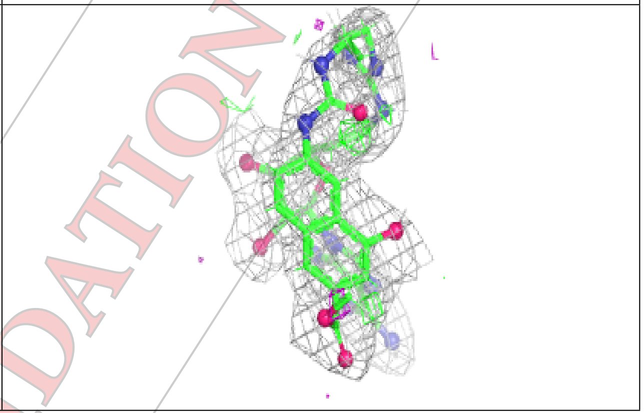

6.5 Other polymers (i)

There are no such residues in this entry.

CONFIDENTIAL VALIDATION REPORT
